# Supplementary material for: Can we accurately forecast non-elective bed occupancy and admissions in the NHS? A time-series MSARIMA analysis of longitudinal data from an NHS Trust
Source: BMJ Open. 2022 Apr 19;12(4):e056523. doi: 10.1136/bmjopen-2021-056523 (PMC9021768; doi:10.1136/bmjopen-2021-056523)
Supplement: Supplementary data [file bmjopen-2021-056523supp001.pdf]

**Appendix 1:** Subforecast figures

**Figure A1: Model 2 (medicine admissions)**

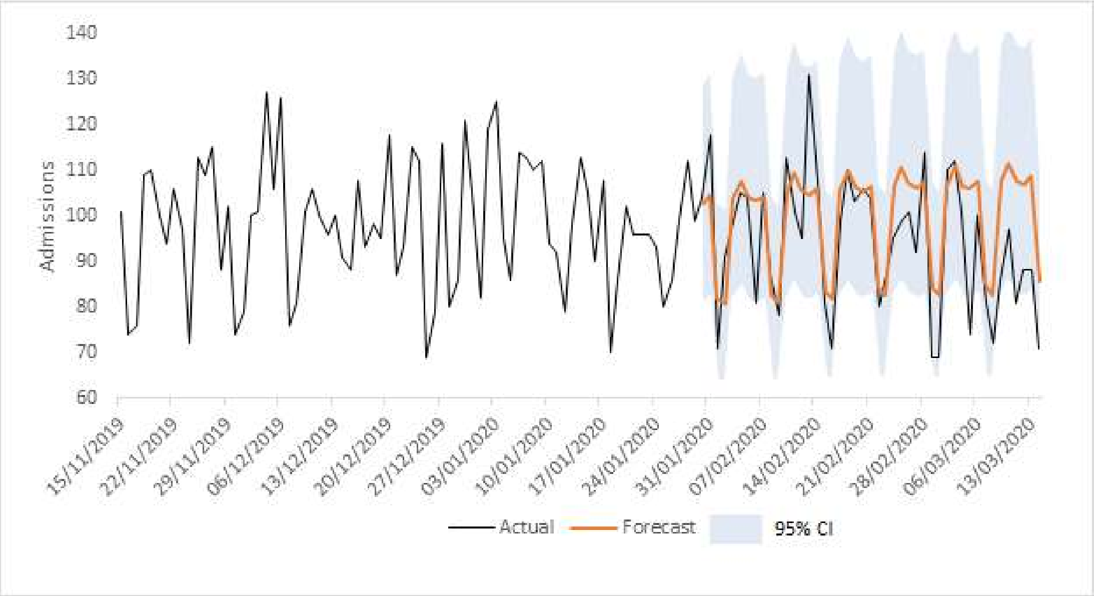


**Figure A2: Model 3 (surgery admissions)**

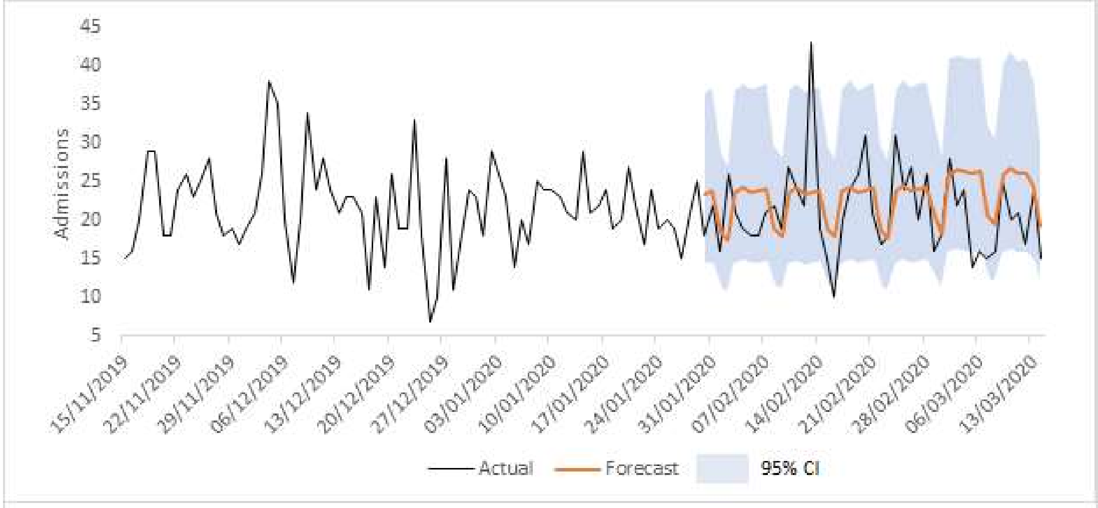

**Figure A3: Model 4 (under 48 hour length of stay admissions)**

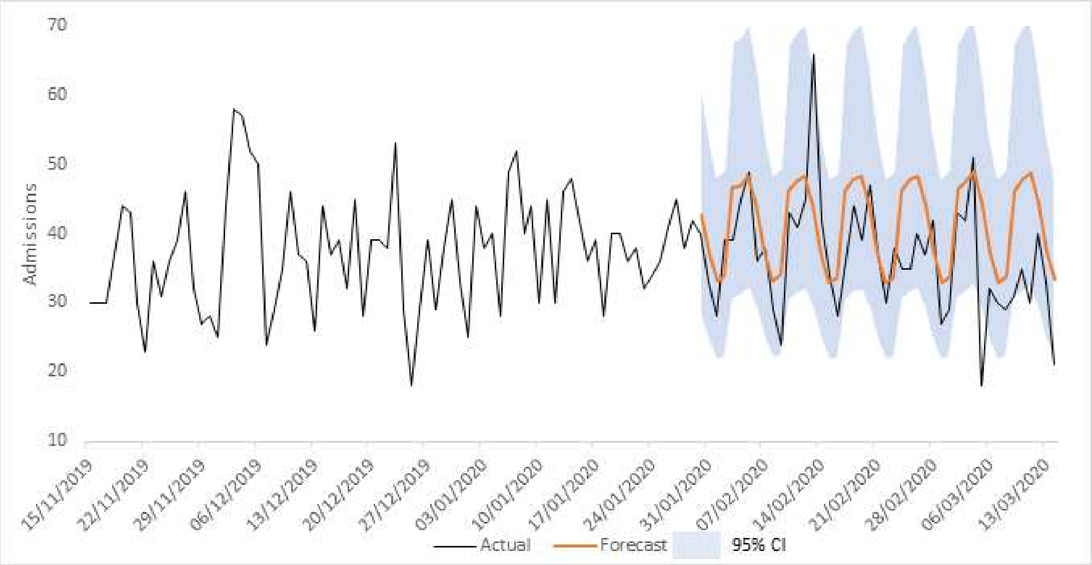


**Figure A4: Model 5 (over 48 hour length of stay admissions)**

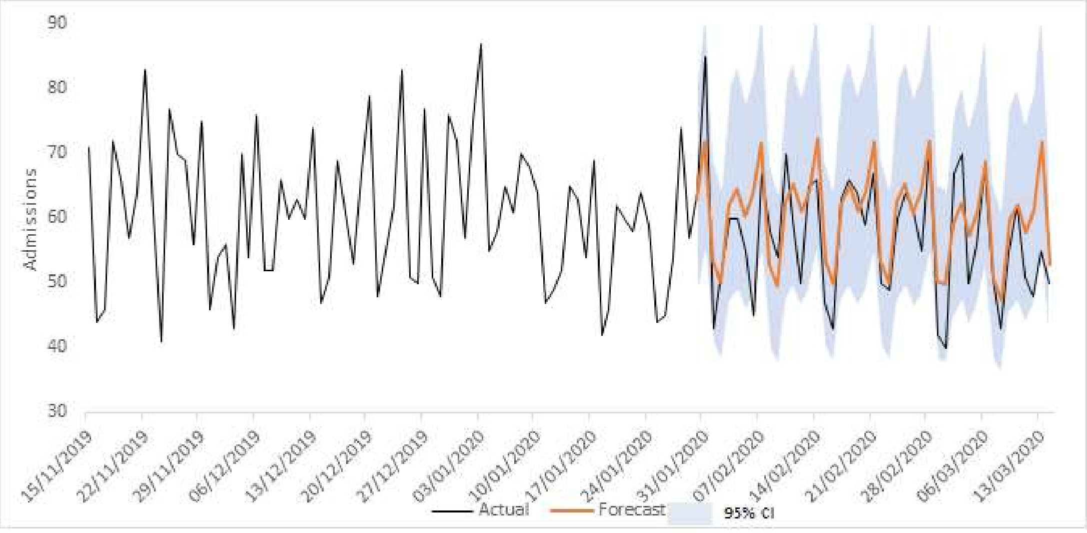


**Figure A5: Model 6 (under 48 hour length of stay medicine admissions)**

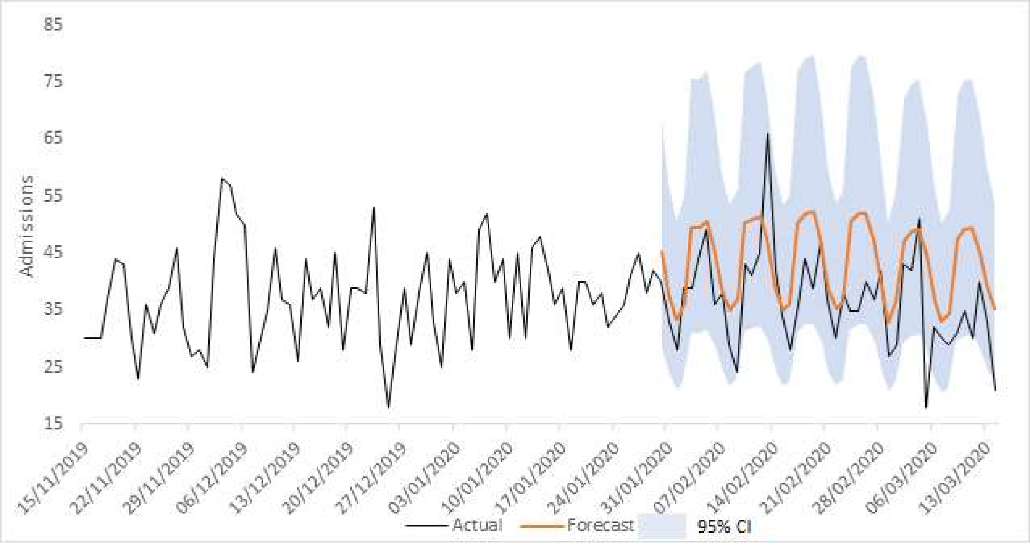


**Figure A6: Model 7 (under 48 hour length of stay surgery admissions)**

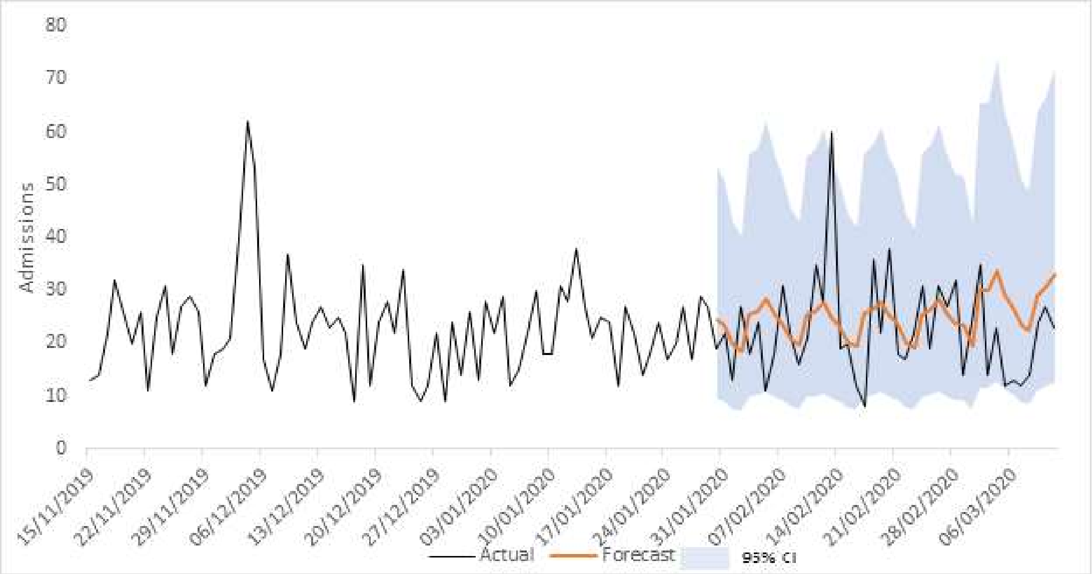


**Figure A7: Model 8 (over 48 hour length of stay medicine admissions)**

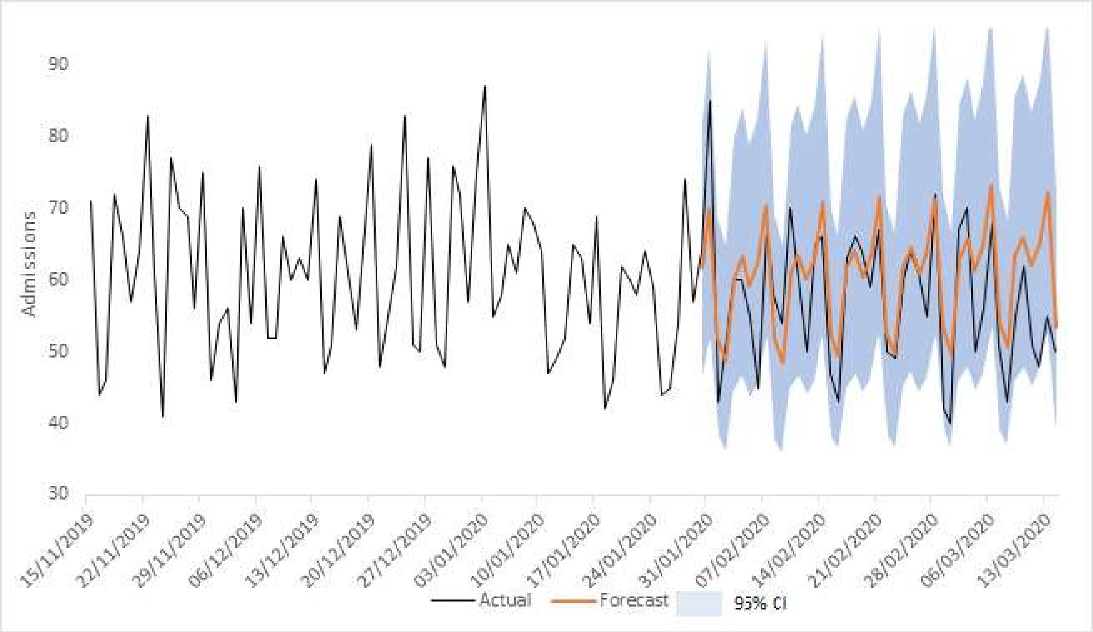


**Figure A8: Model 9 (over 48 hour length of stay surgery admissions)**

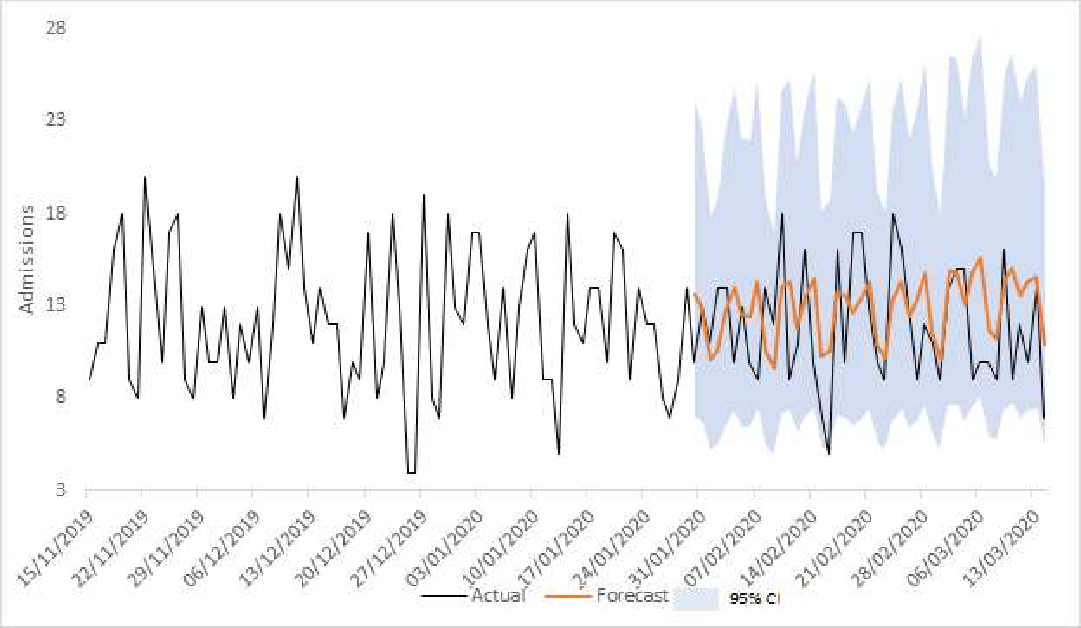


**Figure A9: Model 10 (all bed occupancy)**

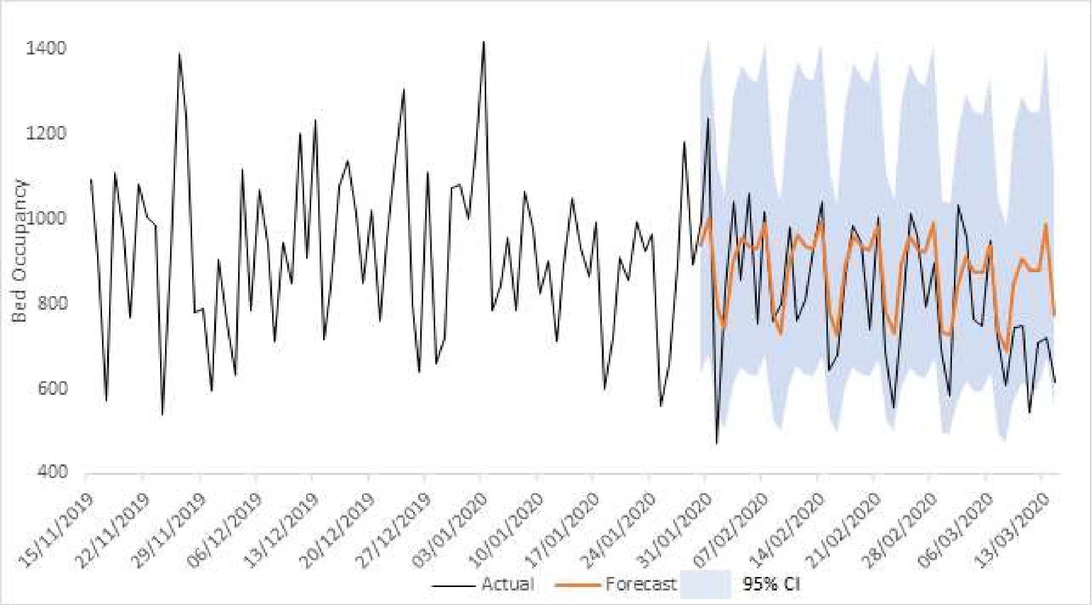


**Figure A10: Model 11 (medicine bed occupancy)**

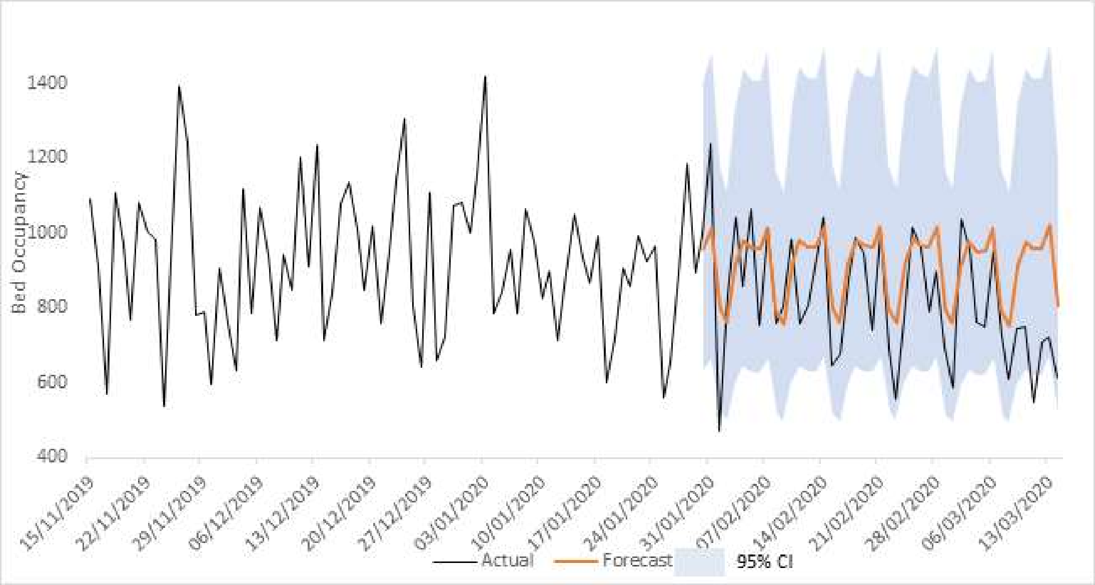


**Figure A11: Model 12 (surgery bed occupancy)**

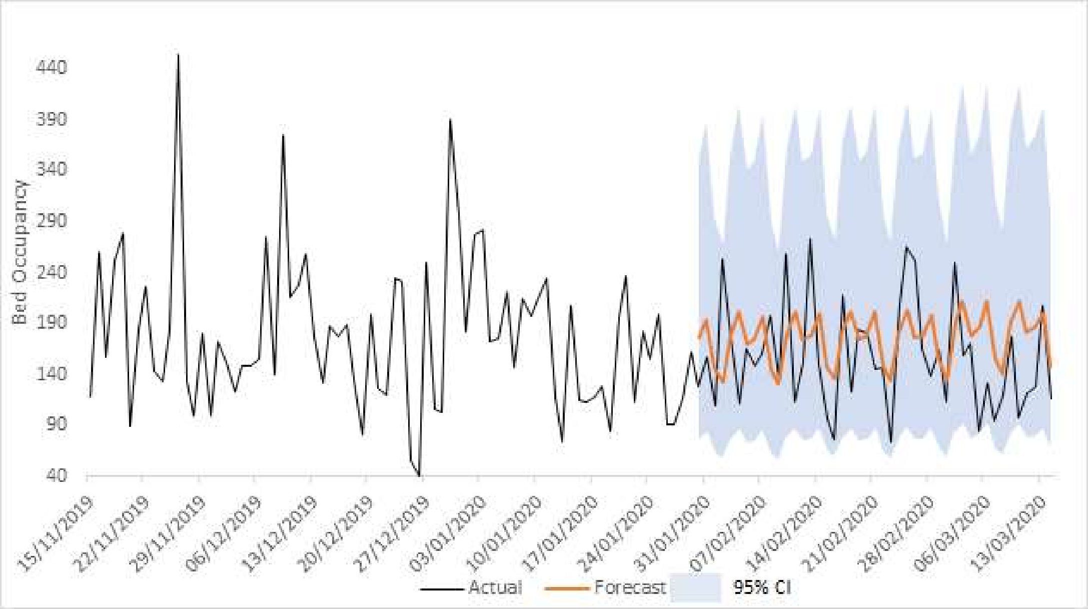


**Figure A12: Model 13 (under 48 hour length of stay bed occupancy)**

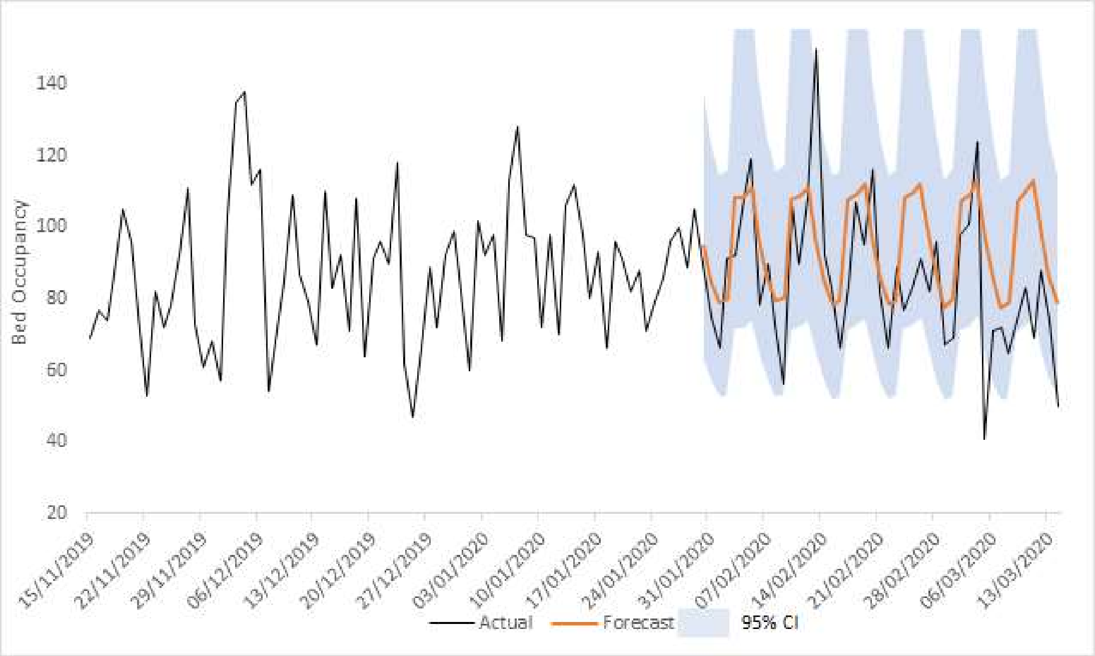


**Figure A13: Model 14 (over 48 hour length of stay bed occupancy)**

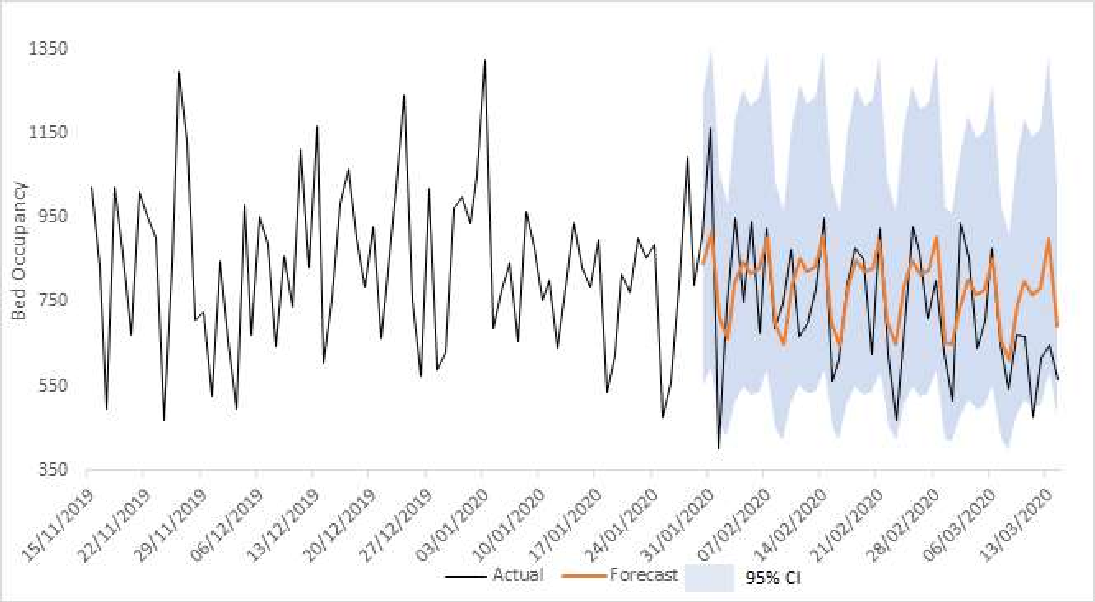


**Figure A14: Model 15 (under 48 hour length of stay medicine bed occupancy)**

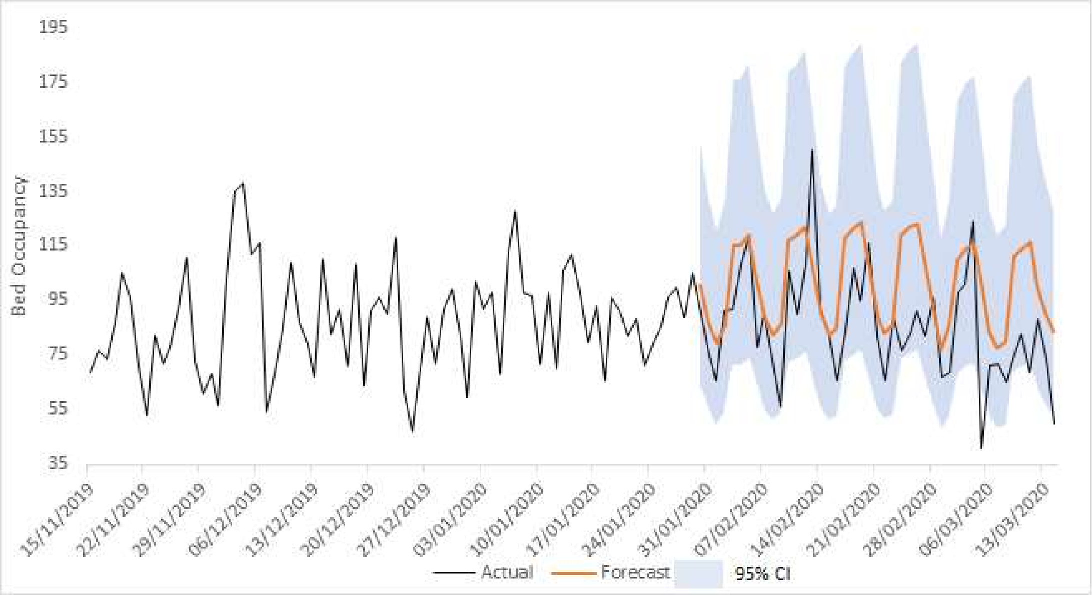


**Figure A15: Model 16 (under 48 hour length of stay surgery bed occupancy)**

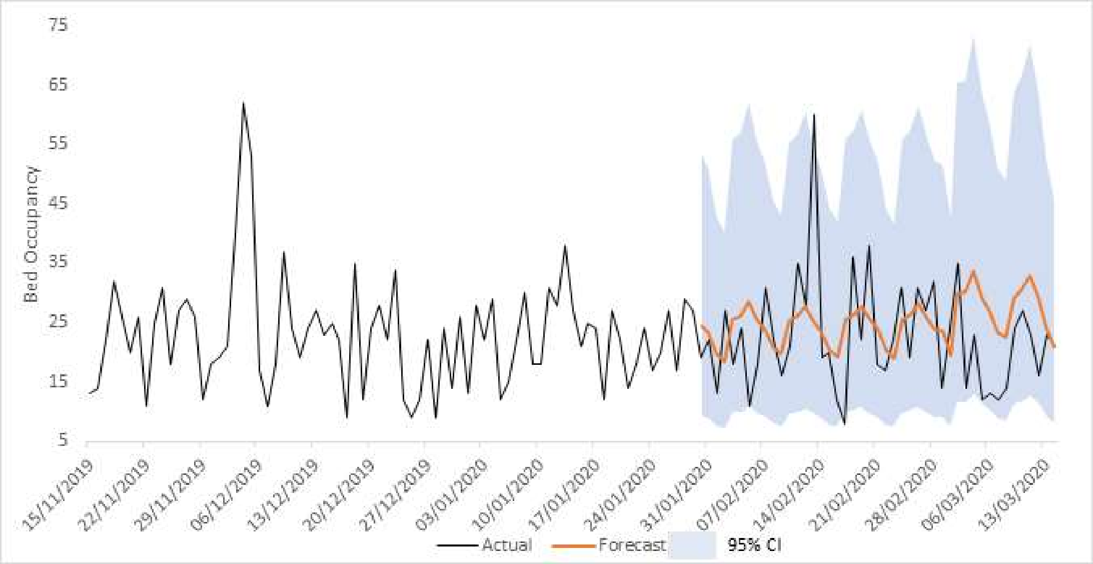


**Figure A16: Model 17 (over 48 hour length of stay medicine bed occupancy)**

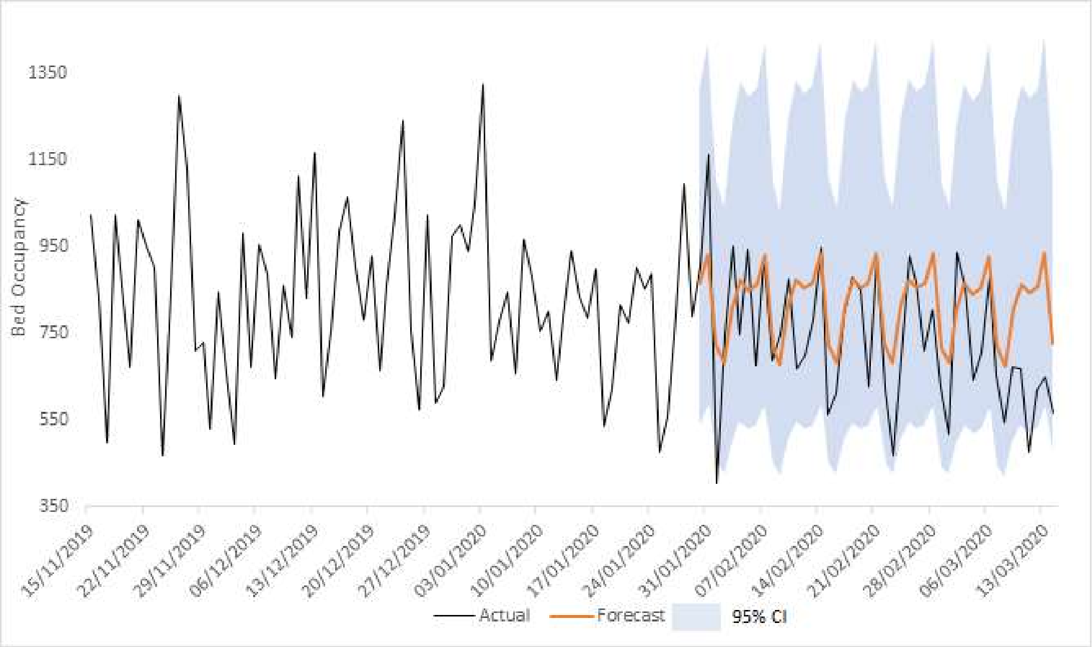


**Figure A17: Model 18 (over 48 hour length of stay surgery bed occupancy)**

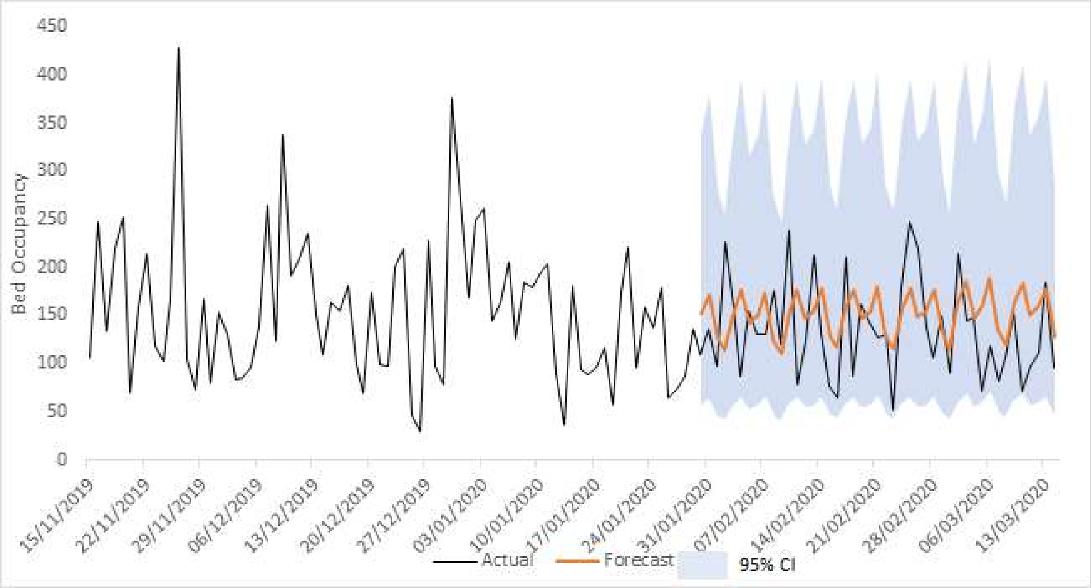
450
400
350
300
250
200
150
100
50
0
Bed Occupancy
15/11/2019
22/11/2019
29/11/2019
06/12/2019
13/12/2019
20/12/2019
27/12/2019
03/01/2020
10/01/2020
17/01/2020
24/01/2020
31/01/2020
07/02/2020
14/02/2020
21/02/2020
28/02/2020
06/03/2020
13/03/2020
Actual
Forecast
95% CI
