# Supplementary material for: Can we accurately forecast non-elective bed occupancy and admissions in the NHS? A time-series MSARIMA analysis of longitudinal data from an NHS Trust
Source: BMJ Open. 2022 Apr 19;12(4):e056523. doi: 10.1136/bmjopen-2021-056523 (PMC9021768; doi:10.1136/bmjopen-2021-056523)
Supplement: Supplementary data [file bmjopen-2021-056523supp002.pdf]

```
#clear the environment of all data
rm(list = ls())

## install necessary packages (Can skip this step if you have them)
install.packages("readstata13", "Hmisc", "DataCombine", "lmtest","tidyverse",
"scales", "forecast", "zoo", "tempdisagg", "imputeTS", "lubridate", "aTSA",
"tseries", "gridExtra", "broom", "knitr", "dplyr", "pixedust")


#tidy regression output libraries
library(broom)
library(knitr)
require(broom)
require(knitr)
library(dplyr) ## this is the library with 'pipes' %>%
library(pixiedust)



## Packages needed
## If you don't have a package, use 'install.packages("NAME OF PACKAGE HERE")
#bring in stata data
library(readstata13)

#lag function
library(Hmisc)

#Row insertion library for missing data problem
library(DataCombine)

#Some tests are in these libraries
library(lmtest)


#tidyverse includes many other useful libraries for formatting data, scales
includes date formatting
library(tidyverse)
library(scales)

#forecasting and time series libraries
library(forecast)
library(zoo)
library(tempdisagg)
library(imputeTS)
library(lubridate)
library(aTSA)
library(tseries)

#laying out graph library
library(gridExtra)
```

```
#tidy regression output libraries
library(broom)
library(knitr)
require(broom)
require(knitr)
library(dplyr) ## this is the library with 'pipes' %>%
library(pixiedust)



# Set file path for forecasting data

setwd("YOUR WORKING DIRECTORY HERE")


# Transfer Stata dataset into R datasets. Use the relevant dataset in 'hosp_data'
hosp_data = read.dta13("COLLAPSED HOSPITAL DATA HERE", convert.factors = TRUE,
generate.factors = TRUE, nonint.factors = TRUE)
temp = read.csv("TEMPERATURE WITH SUFFICIENT LAG (ONE YEAR BEFORE HOSPITAL DATA
START) HERE")
precip = read.dta13("PRECIPITATION WITH SUFFICIENT LAG (ONE YEAR BEFORE HOSPITAL
DATA START) HERE", convert.factors = TRUE, generate.factors = TRUE, nonint.factors
= TRUE)

#Use below if temperature/precipitation data is in dta format from stata, but new
code to combine in R is much more efficient.
#temp = read.dta13("temperatures_lag.dta", convert.factors = TRUE, generate.factors
= TRUE, nonint.factors = TRUE)
#precip = read.dta13("temperatures_lag.dta", convert.factors = TRUE,
generate.factors = TRUE, nonint.factors = TRUE)

#make a full date variable for the temp and precip data to match hosp_data
temp = temp %>%
  mutate(admidate=make_date(year, month, day))

precip = precip %>%
  mutate(admidate=make_date(admiyear, admimonth, admiday))


#check the plot to make sure it looks like you would expect (time series data, with
a count per day)

plot(hosp_data$id)


#pull out the length and the end date of the hospital data, for data manipulation
purposes

leng = nrow(hosp_data)
```

```
end_date=hosp_data$dateindex[leng]
init_date=hosp_data$dateindex[1]


#check the data if there's a huge outlier (often for some of the bed occupancy
subforecasts)

plot(hosp_data$id)

#find the position of the outlier, then make it an average of the previous week's
occupancy/admissions.
#CHECK: max admissions/max occupancy with NBT

outval = which.max(hosp_data$id) #if the outlier is higher
#outval = which.min(hosp_data$id) #if the outlier is lower (this one is commented
out)
hosp_data$id[outval] #displays the value
hosp_data$id[outval] = as.integer(sum(hosp_data$id[(outval-7):(outval-1)])/7)
#makes the outlier an average of the previous 7 days' occupancy/admissions


#sort out the lag temperature - the temperature and precipitation files should have
one year more data in the past, so that you can do this part
#if we didn't do it this way, if we did last week's temperature, we'd have to
predict the weather accurately if our forecast horizon exceeded one week
#in conclusion, since we're not meteorologists, it's probably better to just use
last year's, so we can use whatever horizon we'd like up to a year

temp$last_year = Lag(temp$avg_temp, 365)

#lag precipitation

precip$last_year = Lag(precip$rainfall, 365)

#overwrite the dataframes so that they're the same length as the hospital
admissions data
#the models won't work if the inputs have different lengths

precip= subset(precip, admidate>=init_date & admidate<=end_date)
temp = subset(temp, admidate>=init_date & admidate<=end_date)

#Looking at missingness in the temperature and precipitation data
#Some days may be missing, (NOT IN CURRENT TRANCHE) and missing values need to be
imputed
#If there are no missing values, it will say 'integer (empty)' in the environment

missing_t = which(is.na(temp$last_year))
missing_p = which(is.na(precip$last_year))

#A quick fix for NAs in the temperature and precipitation data, if there
```

```
#are missing values, but don't run if there aren't.

temp$last_year[missing_t] = na.spline(temp$last_year)
precip$last_year[missing_t] = na.spline(precip$last_year)

#Check for missing dates - for subforecasts this might be an issue for those with
small numbers
#Make sure to put 0s in when preparing subforecast data in Stata rather than
allowing missingness
#If there are missing dates, go back to the preparation stage.
#It will say 'Date' num(0) in the global environment if there are no missing dates

## THIS SECTION IS ONLY FOR IF THERE ARE MISSING COUNT DATA ON ANY DAY ##

missing_d = as.Date(setdiff(precip$admidate,hosp_data$dateindex))

#If they are missing, add them in as '0' values (missingness means there was
#no occupancy on those dates, as far as I understand).

#First create a new dataframe with the information that's missing
#Name the columns Add the new dataframe to the end of the old one, then sort
("arrange")

add_miss = data.frame(missing_d, 1, month(missing_d), year(missing_d),
day(missing_d), wday(missing_d), 0)
names(add_miss)= c("dateindex", "id", "month", "year", "day", "dow", "yearmonth")
hosp_data=rbind(hosp_data, add_miss)
hosp_data=hosp_data %>% arrange(dateindex)
rm(add_miss)


leng=nrow(hosp_data)

#tidy up a bit
rm(missing_d,missing_t,missing_p)

#check and record the mean and standard deviation of the data
mean(hosp_data$id)
sd(hosp_data$id)


#Transform the dataframes into time series objects, with a daily 'seasonality'

hospts <- msts(hosp_data, seasonal.periods = c(7,365.25), ts.frequency = 7)
tempts <- msts(temp, seasonal.periods = c(7,365.25), ts.frequency = 7)
precipts <- msts(precip, seasonal.periods = c(7,365.25), ts.frequency = 7)



#Check ACF plot of the count to confirm the presence of autocorrelation.
```

```
#If the values fall between the blue lines (p<0.05), then the data are not
#likely to be autocorrelated. If they do fall outside the lines, they are likely to
be.
#This uses a lag of 21 days
# "The ACF of stationary data drops to zero relatively quickly"
# "The ACF of non-starionary data decreases slowly"

acf(hospts[,"id"], lag = 21)

#Check Partial ACF plot to confirm that the autocorrelation is or isn't a result of
the
#autocorrelation of the first lag carrying through (if it is, the spikes on the
PACF will
#peak at the first lag, then be in between the blue lines (p <0.05))
# "A partial autocorrelation is the amount of correlation between a variable
#and a lag of itself that is not explained by correlations at all
lower-order-lags."
#This uses a lag of 21 days

pacf(hospts[,"id"], lag = 21)

#Fit a regression model to formally test for a lagged autocorrelation in the
residuals

#create a time variable
timef <- seq_along(hospts[,"id"])

#fit a linear regression to model the series as a function of time
lin_reg = tslm(hospts[,"id"] ~ timef)

#Durbin-Watson test - check for lag-1 autocorrelated residuals
#H0 = residuals do not have lag-1 auotocorrelation
#small p -> there is evidence for lag-1 autocorrelation

dwtest(lin_reg)

#tidy up
rm(timef, lin_reg)


#Determine stationarity of the data (it is non-stationary if it has a trend,
seasonal
#effect, or any systematic change in mean and variance


#Seasonal decomposition graphs (including weekly and annual seasonality)
#These are decomposed to the full sample and also financial years

## You may need to adjust these to the years of your data (the label), but the
actual end of the window
```

```
##is one year long##

hospts[,"id"] %>% mstl(lambda = 0) %>% autoplot() + xlab("week") +
  ggtitle("Estimated components (full sample)")

#2015-2016 (check the weeks of the year for these)

hospts[,"id"] %>% mstl(lambda = 0) %>% window(end = 52) %>% autoplot(facet=TRUE) +
xlab("week") +
  ggtitle("Estimated components (2015-2016)")

#2016-2017 (check the weeks of the year for these)

hospts[,"id"] %>% mstl(lambda = 0) %>% window(start = 53, end = 105) %>%
autoplot(facet=TRUE) +
  xlab("week") + ggtitle("Estimated components (2016-2017)")

#2017-2018 (check the weeks of the year for these)

hospts[,"id"] %>% mstl(lambda = 0) %>% window(start = 106, end = 158) %>%
autoplot(facet=TRUE) +
  xlab("week") + ggtitle("Estimated components (2017-2018)")

#2018-2019 (check the weeks of the year for these)

hospts[,"id"] %>% mstl(lambda = 0) %>% window(start = 159, end = 211) %>%
autoplot(facet=TRUE) +
  xlab("week") +ggtitle("Estimated components (2018-2019)")

#2019-'2020' (check the weeks of the year for these -
#keep in mind that we currently don't have all the data for this financial year

hospts[,"id"] %>% mstl(lambda = 0) %>% window(start = 212, end = 250) %>%
autoplot(facet=TRUE) +
  xlab("week") +ggtitle("Estimated components (2019-2020)")

# Check the seasonality of the data - annual seasonality often includes Christmas
(push to discharge before Christmas) and at times Easter
# There is also usually weekly seasonality. Check the trend of the data as well.

#Conduct an augmented Dickey-Fuller test (ADF) test to formally test for
stationarity
#H0 = non-stationary, H1 = stationary. low p value = reject the null

adf.test(hospts[,"id"])

#If the data are not stationary (i.e. high p), transform them to be stationary
(differencing)
#if the adf test rejects the null, then don't difference as they are stationary.
#if you difference once, and it still isn't stationary, consider differencing the
```

```
differenced data
#or, log the data (if the variance is unstable). Currently commented out.

#hospts = diff(hospts)

#Onto modelling, creating the data. Please note if you differenced the data, you
will need to change the variable names:

# Create deterministic trend and log deterministic trend

trend <- seq_along(hospts[,"id"])

# Create dummy daily variables

mon <- ifelse(hospts[,"dow"] == 1, 1, 0) # create dummy variable equal to 1 if
Monday and 0 otherwise
tue <- ifelse(hospts[,"dow"] == 2, 1, 0)
wed <- ifelse(hospts[,"dow"] == 3, 1, 0)
thu <- ifelse(hospts[,"dow"] == 4, 1, 0)
fri <- ifelse(hospts[,"dow"] == 5, 1, 0)
sat <- ifelse(hospts[,"dow"] == 6, 1, 0)

# Create dummmy(ies) for Christmas effect

chris <- ifelse(hospts[,"day"] == 25 & hospts[,"month"] == 12, 1, 0)
chrisweek <- ifelse(hospts[,"day"] %in% c(23, 24, 25, 26, 27) & hospts[,"month"] ==
12, 1, 0)
easter2015 <- ifelse(hospts[,"day"] %in% c(3,4,5,6) & hospts[,"month"] == 4, 1, 0
& hospts[,"year"] == 2015)
easter2016 <- ifelse(hospts[,"day"] %in% c(25,26,27,28) & hospts[,"month"] == 3, 1,
0  & hospts[,"year"] == 2016)
easter2017 <- ifelse(hospts[,"day"] %in% c(14,15,16,17) & hospts[,"month"] == 4, 1,
0  & hospts[,"year"] == 2017)
easter2018a <- ifelse(hospts[,"day"] %in% c(30,31) & hospts[,"month"] == 3, 1, 0  &
hospts[,"year"] == 2018)
easter2018b <- ifelse(hospts[,"day"] %in% c(1,2) & hospts[,"month"] == 4, 1, 0  &
hospts[,"year"] == 2018)
easter2018 <- easter2018a + easter2018b
easter2019 <- ifelse(hospts[,"day"] %in% c(19,20,21,22) & hospts[,"month"] == 4, 1,
0  & hospts[,"year"] == 2019)

easterall <- easter2015 + easter2016 + easter2017 + easter2018 + easter2019

bhm2015 <- ifelse(hospts[,"day"] %in% c(4,25) & hospts[,"month"] == 5, 1, 0  &
hospts[,"year"] == 2015)
bha2015 <- ifelse(hospts[,"day"] %in% c(31) & hospts[,"month"] == 8, 1, 0  &
hospts[,"year"] == 2015)
bh2015 <- bhm2015 + bha2015
bhj2016 <- ifelse(hospts[,"day"] %in% c(1) & hospts[,"month"] == 1, 1, 0  &
hospts[,"year"] == 2016)
```

```
bhm2016 <- ifelse(hospts[,"day"] %in% c(2,30) & hospts[,"month"] == 5, 1, 0  &
hospts[,"year"] == 2016)
bha2016 <- ifelse(hospts[,"day"] %in% c(29) & hospts[,"month"] == 8, 1, 0  &
hospts[,"year"] == 2016)
bh2016 <- bhj2016 + bhm2016 + bha2016
bhj2017 <- ifelse(hospts[,"day"] %in% c(2) & hospts[,"month"] == 1, 1, 0  &
hospts[,"year"] == 2017)
bhm2017 <- ifelse(hospts[,"day"] %in% c(1,29) & hospts[,"month"] == 5, 1, 0  &
hospts[,"year"] == 2017)
bha2017 <- ifelse(hospts[,"day"] %in% c(28) & hospts[,"month"] == 8, 1, 0  &
hospts[,"year"] == 2017)
bh2017 <- bhj2017 + bhm2017 + bha2017
bhj2018 <- ifelse(hospts[,"day"] %in% c(1) & hospts[,"month"] == 1, 1, 0  &
hospts[,"year"] == 2018)
bhm2018 <- ifelse(hospts[,"day"] %in% c(7,28) & hospts[,"month"] == 5, 1, 0  &
hospts[,"year"] == 2018)
bha2018 <- ifelse(hospts[,"day"] %in% c(27) & hospts[,"month"] == 8, 1, 0  &
hospts[,"year"] == 2018)
bh2018 <- bhj2018 + bhm2018 + bha2018
bhj2019 <- ifelse(hospts[,"day"] %in% c(1) & hospts[,"month"] == 1, 1, 0  &
hospts[,"year"] == 2019)
bhm2019 <- ifelse(hospts[,"day"] %in% c(6,27) & hospts[,"month"] == 5, 1, 0  &
hospts[,"year"] == 2019)
bha2019 <- ifelse(hospts[,"day"] %in% c(26) & hospts[,"month"] == 8, 1, 0  &
hospts[,"year"] == 2019)
bh2019 <- bhj2019 + bhm2019 + bha2019

bhall <- bh2015 + bh2016 + bh2017 + bh2018 + bh2019

hollall <- easterall + bhall +chrisweek

#clean up the extra variables we don't need

rm(bhm2015,bha2015,bh2015,bhj2016,bhm2016,bha2016,bhj2017,bhm2017,bha2017,bhj2018,b
hm2018,bha2018,bhj2019,bhm2019,bha2019,chris)
rm(easter2015,easter2016,easter2017,easter2018,easter2018a,easter2018b,easter2019)


# Create a matrix of regressors for trend and daily dummies

trend_days <- tibble(trend, mon, tue, wed, thu, fri, sat, chrisweek, easterall,
bhall, tempts[,"last_year"], precipts[,"last_year"]) # create matrix of regressors:
trend + daily dummies
trend_days_noCLIM <- tibble(trend, mon, tue, wed, thu, fri, sat, chrisweek,
hollall) # create matrix of regressors: trend + daily dummies


#create the time series with the regressors included

seasonality <- msts(trend_days, seasonal.periods = c(7,365), ts.frequency = 7)
```

```
seasonality_noCLIM <-  msts(trend_days_noCLIM, seasonal.periods = c(7,365),
ts.frequency = 7)


# Estimate models with and without climatic data - other model types can be found
in the code graveyard

fit_trend <- auto.arima(hospts[,"id"], d=0, xreg=cbind(seasonality), lambda=0,
biasadj=TRUE)
fit_trend_noCLIM <- auto.arima(hospts[,"id"], d=0, xreg=cbind(seasonality_noCLIM),
lambda=0, biasadj=TRUE)

#Checks for the deterministic trend

summary(fit_trend) #to get model fit parameters manually
res_trend <- residuals(fit_trend)
checkresiduals(fit_trend, points = FALSE)
ggPacf(res_trend, lag=70)
adf.test(res_trend)
trend_nonseas = c(fit_trend$arma[1], fit_trend$arma[6], fit_trend$arma[2]) #pull
out the nonseasonal (first part) ARIMA parameters
trend_seas = c(fit_trend$arma[3], fit_trend$arma[7], fit_trend$arma[4]) #pull out
the seasonal (second part) ARIMA parameters

#Checks for the deterministic trend without climate data
summary(fit_trend_noCLIM) #to get model fit parameters manually
res_trend_noCLIM <- residuals(fit_trend_noCLIM)
checkresiduals(fit_trend_noCLIM, points = FALSE)
ggPacf(res_trend_noCLIM, lag=70)
adf.test(res_trend)
trend_noCLIM_nonseas = c(fit_trend_noCLIM$arma[1], fit_trend_noCLIM$arma[6],
fit_trend_noCLIM$arma[2]) #pull out the nonseasonal (first part) ARIMA parameters
trend_noCLIM_seas = c(fit_trend_noCLIM$arma[3], fit_trend_noCLIM$arma[7],
fit_trend_noCLIM$arma[4]) #pull out the seasonal (second part) ARIMA parameters


# Forecasting on training and test sets - 6 week horizon

train.hospts <- subset(hospts[,"id"], end = length(hospts[,"id"]) - 45)
test.hospts <- subset(hospts[,"id"], start=length(hospts[,"id"])-44)

lentrain=length(train.hospts)


#xlim=c(230,241) to examine the training/test split
train.hospts %>% autoplot()+
  autolayer(test.hospts, color = "red")


#deterministic trend
```

```
trend_model <- Arima(train.hospts, order=trend_nonseas, seasonal=trend_seas,
include.constant = TRUE,
                     xreg = cbind(seasonality[1:lentrain,]),
                     lambda=0, biasadj=TRUE)

trend_fcast <- forecast(trend_model, level = c(95), xreg =
cbind(seasonality[(lentrain+1):leng,]))

trend_fcast %>% autoplot() +
  autolayer(test.hospts, series="Actual value", color = 'darkorange') +
  guides(colour=guide_legend(title="")) +
  ggtitle("Deterministic trend model") +
  ylab("Daily admissions")


#deterministic trend no climate data

trend_model_noCLIM <- Arima(train.hospts, order=trend_noCLIM_nonseas,
seasonal=trend_noCLIM_seas, include.constant = TRUE,
                     xreg = cbind(seasonality_noCLIM[1:lentrain,]),
                     lambda=0, biasadj=TRUE)

trend_fcast_noCLIM <- forecast(trend_model_noCLIM, level = c(95), xreg =
cbind(seasonality_noCLIM[(lentrain+1):leng,]))

trend_fcast_noCLIM %>% autoplot() +
  autolayer(test.hospts, series="Actual value", color = 'darkorange') +
  guides(colour=guide_legend(title="")) +
  ggtitle("Deterministic trend model no climate") +
  ylab("Daily admissions")




# assess the accuracy - for AIC/BIC check model summaries - summary(trend_model)
etc

accuracy(trend_model$fitted, hospts[,"id"])
accuracy(trend_model_noCLIM$fitted, hospts[,"id"])


#residual differences tables
diff_trend = hospts[(lentrain+1):leng,"id"] - trend_fcast$mean
date = as.character(paste(hospts[(lentrain+1):leng,"year"],
hospts[(lentrain+1):leng,"month"],  hospts[(lentrain+1):leng,"day"], sep="/"))
dow = factor(as.character(hospts[(lentrain+1):leng,"dow"]), levels =
c(0,1,2,3,4,5,6), labels = c("Sun", "Mon", "Tues", "Weds", "Thurs", "Fri", "Sat"))

#admissions values
```

```
acc_trend = abs(diff_trend) <= (0.2*mean(hosp_data$id))
acc_trend_s = abs(diff_trend) < (0.1*mean(hosp_data$id))


##put it into a nice format
diff_trend_df=data.frame("date" = date, "dow" = dow , "actual" =
hospts[(lentrain+1):leng,"id"], "predicted" = trend_fcast$mean, "difference"=
diff_trend, "lower95" = trend_fcast$lower, "upper95" = trend_fcast$upper,
"accuracybig" = acc_trend,  "accuracybetter" = acc_trend_s)

##write to a csv (easier to copy)- when you open it in Excel the date column will
look weird - just make sure to format it as a date in Excel and pick a format that
shows up properly - also you can reduce the df in excel
write.csv(diff_trend_df, file="Trend_diff.csv", row.names=FALSE)

##write all the data to a csv for making graphs
write.csv(hosp_data, file="all_data.csv", row.names=FALSE)
```
