# Supplementary material for: Can we accurately forecast non-elective bed occupancy and admissions in the NHS? A time-series MSARIMA analysis of longitudinal data from an NHS Trust
Source: BMJ Open. 2022 Apr 19;12(4):e056523. doi: 10.1136/bmjopen-2021-056523 (PMC9021768; doi:10.1136/bmjopen-2021-056523)
Supplement: Supplementary data [file bmjopen-2021-056523supp003.pdf]

Appendix 3: Model specifications and AICs for models with and without climatic data

Table A3.1: Model specifications. Guide to acronyms: a- admissions, bo- bed occupancy, o48 – over 48 hour length of stay, u48 – under 48 hour length of stay, med – medicine, surg - surgery

A/BO	Time	Climate	Speci alty	SARIMA	RMSE	MAPE	mean	sd	AIC
a	all	y	all	(3,0,0)(1,0,0)	9.59	8.66	90.52	15.02	-2069.49
a	all	n	all	(2,0,2)(1,0,0)	9.50	8.59	90.52	15.02	-2043.83
a	all	y	med	(3,0,2)(2,0,2)	8.98	9.44	79.14	19.32	-1817.45
a	all	n	med	(3,0,2)(2,0,2)	8.98	9.43	79.14	19.32	-1813.09
a	o48	y	all	(3,0,1)(1,0,1)	7.22	10.78	55.14	10.04	-1496.64
a	o48	n	all	(2,0,3)(1,0,0)	7.25	10.83	55.14	10.04	-1495.55
a	o48	y	med	(5,0,1)(2,0,0)	6.88	11.97	48.55	12.06	-1242.32
a	o48	n	med	(5,0,1)(2,0,0)	6.88	11.97	48.55	12.06	-1237.50
a	o48	y	surg	(3,0,3)(1,0,2)	3.40	27.45	11.87	3.74	678.12
a	o48	n	surg	(4,0,4)(0,0,2)	3.41	27.47	11.87	3.74	681.20
a	all	y	surg	(1,0,2)(0,0,0)	4.59	19.86	20.65	5.31	-7.37
a	all	n	surg	(2,0,1)(2,0,0)	4.58	19.87	20.65	5.31	-1.96
a	u48	y	all	(1,0,0)(1,0,0)	6.81	16.53	35.38	9.06	-471.22
a	u48	n	all	(2,0,3)(1,0,0)	6.78	16.47	35.38	9.06	-463.39
a	u48	y	med	(4,0,0)(2,0,0)	6.45	18.64	30.59	10.21	-177.19
a	u48	n	med	(4,0,0)(2,0,0)	6.46	18.67	30.59	10.21	-176.50
a	u48	y	surg	(0,0,1)(0,0,0)	7.82	44.19	20.99	8.39	1583.52
a	u48	n	surg	(0,0,2)(0,0,0)	7.85	44.51	20.99	8.39	1583.97
bo	all	y	all	(0,0,1)(1,0,0)	158.24	15.57	849.71	179.77	-620.10
bo	all	n	all	(1,0,3)(1,0,0)	158.17	15.57	849.71	179.77	-618.59
bo	all	y	med	(2,0,1)(0,0,0)	151.69	17.06	760.03	189.22	-423.18
bo	all	n	med	(2,0,1)(0,0,0)	151.75	17.06	760.03	189.22	-418.86
bo	o48	y	all	(0,0,1)(1,0,0)	159.55	17.60	767.41	177.20	-330.94
bo	o48	n	all	(1,0,3)(1,0,0)	159.42	17.62	767.41	177.20	-330.48
bo	o48	y	med	(2,0,1)(0,0,0)	152.70	19.15	689.20	181.24	-153.63
bo	o48	n	med	(2,0,1)(0,0,0)	152.76	19.15	689.20	181.24	-149.13
bo	o48	y	surg	(0,0,1)(0,0,0)	64.95	44.22	141.63	67.49	1661.21
bo	o48	n	surg	(0,0,1)(0,0,0)	65.09	44.36	141.63	67.49	1662.07
bo	all	y	surg	(0,0,1)(0,0,0)	64.50	35.40	162.62	67.71	1248.15
bo	all	n	surg	(0,0,1)(0,0,0)	64.63	35.45	162.62	67.71	1248.44
bo	u48	y	all	(3,0,0)(2,0,1)	15.72	16.41	82.30	21.06	-468.39
bo	u48	n	all	(3,0,0)(2,0,1)	15.73	16.42	82.30	21.06	-465.24
bo	u48	y	med	(4,0,0)(2,0,0)	14.95	18.63	70.83	23.95	-170.75
bo	u48	n	med	(4,0,0)(2,0,1)	14.88	18.61	70.83	23.95	-164.97
bo	u48	y	surg	(0,0,1)(0,0,0)	7.82	44.19	21.00	8.38	1583.52
bo	u48	n	surg	(0,0,2)(0,0,0)	7.85	44.51	21.00	8.38	1583.97
