# Supplementary material for: Can we accurately forecast non-elective bed occupancy and admissions in the NHS? A time-series MSARIMA analysis of longitudinal data from an NHS Trust
Source: BMJ Open. 2022 Apr 19;12(4):e056523. doi: 10.1136/bmjopen-2021-056523 (PMC9021768; doi:10.1136/bmjopen-2021-056523)

Appendix 4: Sensitivity Analyses

The results of the sensitivity analyses are presented here. Two other horizons were tested: One from November to December of 2019, and one from May to June of 2020. These analyses were performed on the overall admissions data.

Table A4.1: Model specifications and accuracy

Model	SARIMA (p,d,q)(P,D,Q)	Model accuracy (strict/moderate thresholds)	MAPE	RMSE	Mean of Outcome	SD of outcome	AIC
Dec 2019	(2,0,2)(1,0,0)	75.6%/93.3%	8.57	9.43	90.09	14.97	-1933.76
June 2020	(3,0,1)(1,0,1)	42.2%/77.8%	8.88	9.67	89.28396	15.79	-2063.21

Figure A4.1: Forecast versus Actual values for December 2019 Horizon.

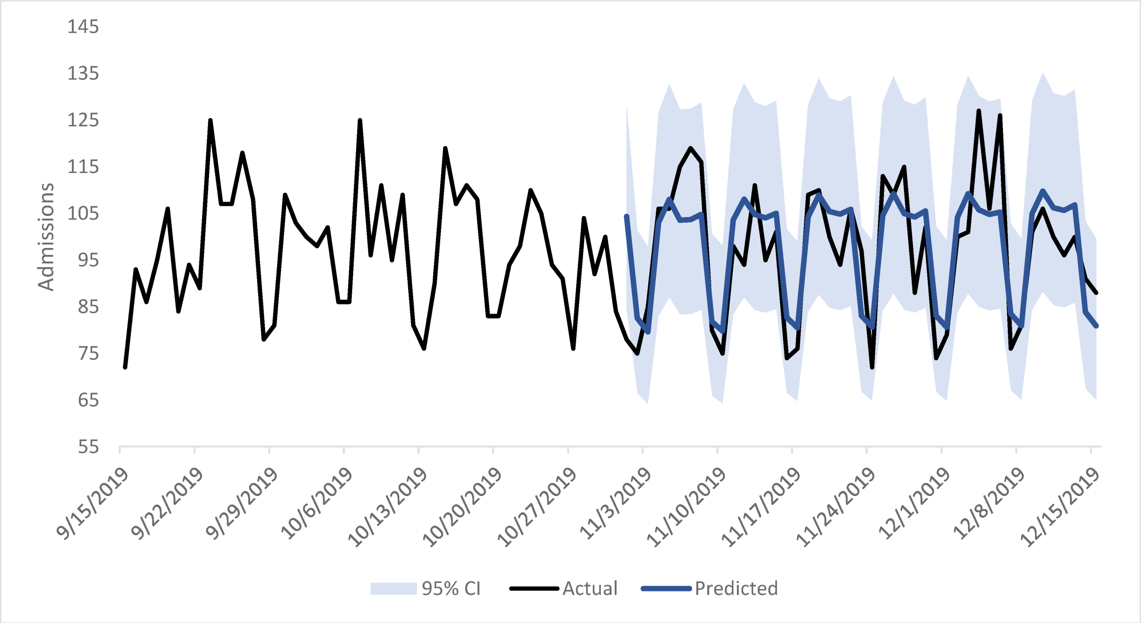

Figure A4.2: Forecast versus Actual values for June 2020 Horizon

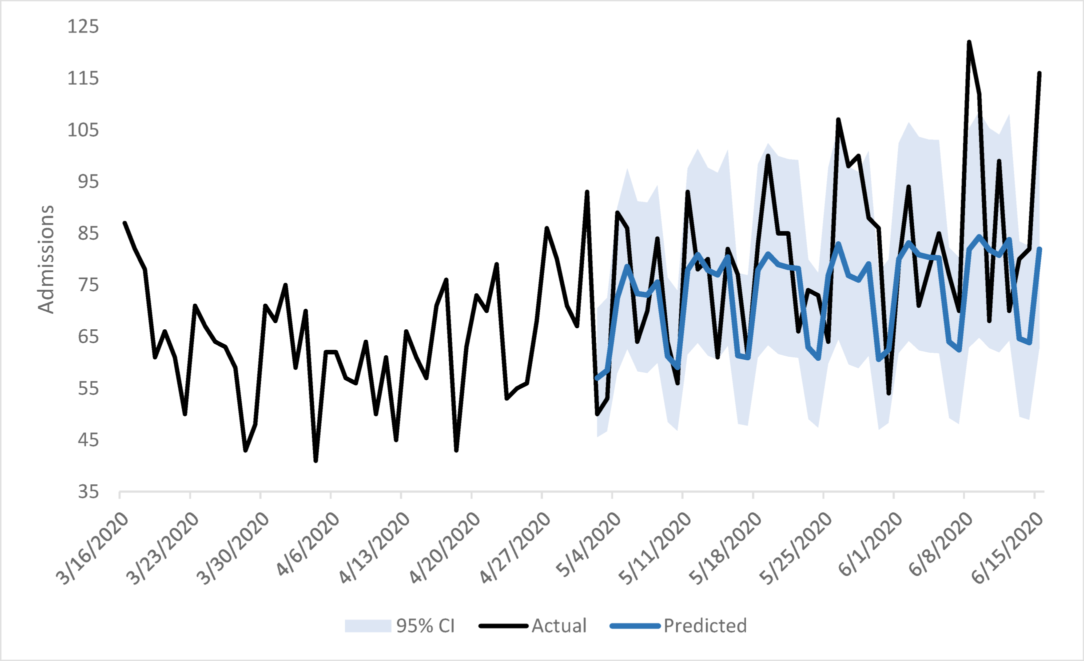

Supplement: Supplementary data [file bmjopen-2021-056523supp004.pdf]
