# Supplementary material for: Can we accurately forecast non-elective bed occupancy and admissions in the NHS? A time-series MSARIMA analysis of longitudinal data from an NHS Trust
Source: BMJ Open. 2022 Apr 19;12(4):e056523. doi: 10.1136/bmjopen-2021-056523 (PMC9021768; doi:10.1136/bmjopen-2021-056523)
Supplement: Author's manuscript [file bmjopen-2021-056523.draft_revisions.pdf]

BMJ Open is committed to open peer review. As part of this commitment we make the peer review history of every article we publish publicly available.

When an article is published we post the peer reviewers' comments and the authors' responses online. We also post the versions of the paper that were used during peer review. These are the versions that the peer review comments apply to.

The versions of the paper that follow are the versions that were submitted during the peer review process. They are not the versions of record or the final published versions. They should not be cited or distributed as the published version of this manuscript.

BMJ Open is an open access journal and the full, final, typeset and author-corrected version of record of the manuscript is available on our site with no access controls, subscription charges or pay-per-view fees (http://bmjopen.bmj.com).

If you have any questions on BMJ Open's open peer review process please email info.bmjopen@bmj.com

BMJ Open

Modelling nonelective bed occupancy and admissions in the North Bristol NHS Trust, England: Demand forecasting study

Journal:	*BMJ Open*
Manuscript ID	bmjopen-2021-056523
Article Type:	Original research
Date Submitted by the Author:	03-Sep-2021

Complete List of Authors:	Eyles, Emily; NIHR ARC West; University of Bristol School of Medical Sciences, Population Health Sciences<br>Redaniel, Maria Theresa; NIHR ARC West; University of Bristol School of Medical Sciences, Population Health Sciences<br>Jones, Tim; NIHR ARC West, NIHR ARC West; University of Bristol School of Medical Sciences, Population Health Sciences<br>Prat, Marion; University of Bristol Faculty of Social Sciences and Law, School of Economics<br>Keen, Tim; North Bristol NHS Trust

Keywords:	STATISTICS & RESEARCH METHODS, HEALTH SERVICES ADMINISTRATION & MANAGEMENT, EPIDEMIOLOGY, ACCIDENT & EMERGENCY MEDICINE

SCHOLARONE™
Manuscripts

I, the Submitting Author has the right to grant and does grant on behalf of all authors of the Work (as defined in the below author licence), an exclusive licence and/or a non-exclusive licence for contributions from authors who are: i) UK Crown employees; ii) where BMJ has agreed a CC-BY licence shall apply, and/or iii) in accordance with the terms applicable for US Federal Government officers or employees acting as part of their official duties; on a worldwide, perpetual, irrevocable, royalty-free basis to BMJ Publishing Group Ltd ("BMJ") its licensees and where the relevant Journal is co-owned by BMJ to the co-owners of the Journal, to publish the Work in this journal and any other BMJ products and to exploit all rights, as set out in our licence.

The Submitting Author accepts and understands that any supply made under these terms is made by BMJ to the Submitting Author unless you are acting as an employee on behalf of your employer or a postgraduate student of an affiliated institution which is paying any applicable article publishing charge ("APC") for Open Access articles. Where the Submitting Author wishes to make the Work available on an Open Access basis (and intends to pay the relevant APC), the terms of reuse of such Open Access shall be governed by a Creative Commons licence – details of these licences and which Creative Commons licence will apply to this Work are set out in our licence referred to above.

Other than as permitted in any relevant BMJ Author's Self Archiving Policies, I confirm this Work has not been accepted for publication elsewhere, is not being considered for publication elsewhere and does not duplicate material already published. I confirm all authors consent to publication of this Work and authorise the granting of this licence.

For peer review only

Modelling nonelective bed occupancy and admissions in the North Bristol NHS Trust, England: Demand forecasting study

Emily Eyles [1,2*] (emily.eyles@bristol.ac.uk) ; Maria Theresa Redaniel [1,2] (theresa.redaniel@bristol.ac.uk); Tim Jones [1,2] (timothy.jones@bristol.ac.uk); Marion Prat [3] (marion.prat@bristol.ac.uk); Tim Keen [4] (tim.keen@nbt.nhs.uk)

1. The National Institute for Health Research Applied Research Collaboration West (NIHR ARC West) at University Hospitals Bristol and Weston NHS Foundation Trust, UK
2. Population Health Sciences, Bristol Medical School, University of Bristol, UK
3. School of Economics, University of Bristol, UK
4. North Bristol NHS Trust, UK

*corresponding author: 9th Floor, Whitefriars, Lewins Mead, Bristol BS1 2NT. emily.eyles@bristol.ac.uk ; 0117 342 1262

For peer review only

For peer review only - http://bmjopen.bmj.com/site/about/guidelines.xhtml

Abstract

Objectives: The main objective of the study was to develop more accurate and precise short-term forecasting models for admissions and bed occupancy for an NHS Trust located in Bristol, England. Subforecasts for the medical and surgical specialties, and for different lengths of stay were realised

Design: Autoregressive integrated moving average models were specified on a training dataset of daily count data, then tested on a six-week forecast horizon. Explanatory variables were included in the models: day of the week, holiday days, lagged temperature and precipitation.

Setting: A secondary care hospital in an NHS Trust in South West England.

Participants: Hospital admissions between September 2016 and March 2020, comprising 1291 days.

Primary and secondary outcome measures: The accuracy of the forecasts was assessed through standard measures, as well as compared to the actual data using accuracy thresholds of 10% and 20% of the mean number of admissions or occupied beds.

Results: The overall ARIMA admissions forecast was compared to the Trust's forecast, and found to be more accurate, namely, being closer to the actual value 95.6% of the time. Furthermore, it was more precise than the Trust's. The subforecasts, as well as those for bed occupancy, tended to be less accurate compared to the overall forecasts. All of the explanatory variables improved the forecasts.

Conclusions: ARIMA models can forecast nonelective admissions in an NHS Trust accurately on a 6-week horizon, which is an improvement on the current predictive modelling in the Trust. These models can be readily applied to other contexts, improving patient flow.

Article Summary

Strengths and limitations of this study:

- The use of ARIMA models, which are simple to set up using *R*, and can be used to improve short term forecasts given enough input data are a strength of this study.
- Limitations include poorer accuracy in subforecasts, possibly due to using a simpler method.
- Data from only one NHS Trust was used, so the models are not validated for other contexts.

Section 1: What is already known on this topic

- Nonelective admissions are rising, and thought to be difficult to predict
- Current models of forecasting nonelective admissions in NHS Trusts can be improved
- Using a variety of model types, short-term forecasts for nonelective admissions and bed occupancy can be produced, but they are not always reliable.

Section 2: What this study adds

- ARIMA models using day of the week, holiday days, and lagged climatic data improve on the predictive models for nonelective admissions for an NHS Trust.

Word Count: 3260

For peer review only - http://bmjopen.bmj.com/site/about/guidelines.xhtml

Introduction

Hospitals are increasingly busy, and there is more demand for resources such as hospital beds than can be easily met. Resource management is a challenge for many hospitals, and with an increase in the number of unplanned admissions, the issue of efficient resource allocation has become more urgent. The annual growth rate in England of emergency admissions is 3.2% on average over the last ten years [2]. Inaccurate estimates of unplanned admissions, and therefore, unpredictable emergency admissions, can lead to the cancellation of planned or routine operations[3]. This leads to subsequent need for more capacity to cope with routine operation backlog. [4 5]. Granular models with daily and weekly forecasts predicted accurately can help strategically plan short term and long term resource management, particularly in order to cope with surges in demand.

In the past three and a half years, emergency admissions in the North Bristol NHS Trust (NBT) increased by 26% while average length of stay was reduced from 7.4 days to 6.1 days over the same period[6]. The North Bristol Trust is situated in Bristol and South Gloucestershire, England. The non-elective activity in NBT, particularly emergency admissions and bed occupancy, is higher than in other hospitals in the Bristol, North Somerset, South Gloucestershire Clinical Commissioning Group area. The consequences of this increase in emergency activity are a difficulty in meeting planned care, and, for the Trust, receiving less pay than expected, as unplanned procedures are paid less than planned ones[6]. As the increase in demand is rising faster than demographic growth, the need to efficiently forecast non-elective activity and understand its predictors has been identified. If the Trust is better able to forecast emergency admissions and understand the factors driving their increase, then it will be able to confidently plan over the longer term. Understanding demand allows the Trust to plan what capacity it will need to meet that demand, including the beds required, the workforce and the theatre time. This in turn influences what capacity is then available for elective work.

The NBT has forecasted using two main methods: a scenario-based model, combining the previous year's growth and demographic growth, and a crude average growth model[6]. These techniques have been found to be insufficient for fine-grained, accurate operational planning, especially at the specialty level[6]. Previous work has used horizons of up to one to two weeks, as forecasting benefit was seen to be limited for longer horizons [3]. Reliable hourly models have been established[7], but the daily horizon over a period of several weeks has been underexplored. Further, most research does not divide by length of stay and specialty, but rather by urgency, or how severe a case is[8 9]. As Medicine and Surgery may have different drivers in terms of admission to hospital, and longer lengths of stay tend to have different characteristics, it makes sense to model these as separate subforecasts as well. Several techniques have been used to predict unplanned admissions, including multiple linear regression[10 11], generalised estimating equations[12], exponential smoothing[8], and the widely-used family of Autoregressive Integrated Moving Average (ARIMA) models[13]. We used Multivariate Seasonal AutoRegressive Integrated Moving Average (MSARIMA) models to forecast emergency admissions and bed occupancy, following the ARIMA forecasting procedure detailed by Jebb et al[14]. MSARIMA models are a special type of regression for time series, which use information from previous days to inform them, ,while adjusting for other factors in the model, such as the weather, or day of the week[8]. SARIMA and ARIMA models have previously been used to forecast emergency department admissions and occupancy[3 8], emergency department crowding (hourly forecasting)[7], and infectious disease bed occupancy[15].

For peer review only - http://bmjopen.bmj.com/site/about/guidelines.xhtml

In this paper, we aim to present a method of forecasting admissions and bed occupancy, using a 45 day (6 week) horizon. We also present subforecasts, divided by length of stay and specialty. The daily horizon over several weeks and the separation by length of stay and specialty are unique to this study, and we further use climatic information to inform the forecast as well. The subforecasts will be assessed using several measures of accuracy, including the mean absolute percentage error, and accuracy thresholds determined by the mean of that particular subforecast. This will demonstrate the possibility of conducting more detailed, specific forecasts, which will allow for better, more granular planning.

Methods

NBT provided operational data on non-elective, i.e. unplanned, admissions to Southmead Hospital, the largest of the three hospitals in the Trust, from September 2016 to March 2020. These data are the precursor to what NBT provides to NHS Digital as part of the Hospital Episode Statistics[16]. The data were split into two: a training dataset of 1246 days to develop the model, and a test dataset of 45 days to test the model. All days had at least one admission or occupied bed, so there were no missing values. The forecast horizon was decided by operational demand. Temperature data were obtained from the Horfield and Filton weather station[17], and precipitation data were obtained from the Met Office daily values for South West England[18].

We made forecasts for admissions and bed occupancy (see table 1). Admissions are the start of a hospital spell[19]. For lengths of stay longer than 'zero', on admission, a patient will occupy a bed, and the number of patients in beds forms bed occupancy. Bed occupancy was calculated by counting a bed as being occupied by a patient between their admission and discharge dates. 'Zero' length of stay patients, i.e. patients who did not stay for a full day, were excluded from the analysis[3]. Zero length of stay is defined operationally as a stay of under 24 hours, which does not overlap midnight. Zero length of stay patients were excluded, due to operational inconsistencies in recording on admission. A stay overlapping midnight becomes an overnight stay, regardless of time of admission.

We controlled for temperature and precipitation (both lagged, the same day last year) the day of the week, holidays (bank, Christmas, and Easter holidays). The temperature and precipitation were lagged to the previous year, as weather prediction is not sufficiently accurate for longer forecast horizons. Weather data were included as they have been tested in a number of other contexts, to mixed results[8 9 20 21]. Atherton et al[22] found that adult trauma admissions were not influenced by the weather, but only used temperature in 5 Celsius increments over a single year, whereas this study uses temperature in Celsius to a single decimal point over a longer time period. In contrast, Macgregor[23] found an association between daily temperature and trauma admissions in Aberdeen, and Rising et al[24] found similar results for temperature and precipitation on trauma admissions in an American hospital. Note that these solely examine trauma admissions, rather than other types of nonelective secondary admissions, which our study includes. Finally, Sahu et al[25] developed a Bayesian model for forecasting hospital admissions in Cardiff and Southampton, two nearby localities to Bristol, the setting of this study, and emphasised the importance and relevance of including weather data.

Day of the week, as well as holidays, inclusive of bank holidays, were found across several studies to be highly relevant in predicting secondary care admissions[20-22 25]. Finally, a simple trend variable was generated, to account for the upward direction of admissions and bed occupancy over time, calculated as the days since the start of the dataset [14]. Table 1 details the variables included in the

For peer review only - http://bmjopen.bmj.com/site/about/guidelines.xhtml

models. Separate subforecasts were also conducted divided by specialty (Surgery or Medicine) and length of stay (over or under 48 hours), detailed in table 2.

Table 1: Outcome variables and explanatory variables

**Variable**	**Definition**
**Outcomes**	
Admissions	Count of admissions to hospital, for a non-zero length of stay, i.e. overnight stays
Bed Occupancy	Count of hospital beds occupied, calculated as a count between admission and discharge dates, for non-zero lengths of stay
**Explanatory variables**	
Day of the week	Sunday, Monday, Tuesday, Wednesday, Thursday, Friday, Saturday
Temperature last year	The average temperature in Celsius on the same day last year
Precipitation last year	The precipitation in millimetres on the same day last year
Holiday	Whether a day was a bank holiday, over Christmas week, or during the Easter holiday weekend

For peer review only

Table 2: Model specifications

**Outcome**	**Treatment Specialty**	**Length of Stay**
**Admissions**	All	All
		<= 48 hours
		> 48 hours
	Medicine	All

For peer review only - http://bmjopen.bmj.com/site/about/guidelines.xhtml

		<= 48 hours
		> 48 hours
	Surgery	All
		<= 48 hours
		> 48 hours
**Bed Occupancy**	All	All
		<= 48 hours
		> 48 hours
	Medicine	All
		<= 48 hours
		> 48 hours
	Surgery	All
		<= 48 hours
		> 48 hours

Seasonal ARIMA models allow for seasonality, or regularly repeating cycles or trends in the data[26], and takes into account the autoregressive order p, d is the differencing order, and q is the moving average order. The second set of brackets represents the seasonal versions of these components[12]. The autoregressive part of the model regresses the forecast variable against its own past values, to the order of the number of time lags (p)[26]. The moving average part of the model uses the past regression errors terms to the order of the time lag of the error (q)[26]. The differencing part of the model (d) refers to how many times past values have been subtracted from the data [26].

The modelling component consists of an examination of the time series to ensure its stationarity, i.e. whether or not observations and their lags are autocorrelated. Unlike other time series modelling techniques, the ARIMA family of models allow for nonstationarity and the inclusion of explanatory variables[14]. In the case of this data, the subsets all featured lag-1 autocorrelation. An augmented Dickey-Fuller test showed no further autocorrelation, negating the need to transform any of the datasets.

The mean absolute percentage error (MAPE) and the root mean square error (RMSE) are presented for each model [3,7-9,13,15,26]. MAPE measures model fit with closer to 0% indicating better fit[9]. For the RMSE, lower values indicate better model performance, though its value is dependent on the mean value of the variable, which are presented in table 3 along with the standard deviations[13]. The model was tested against the test dataset, providing the actual out-of-sample accuracy. The model accuracy was determined by whether the residual of the forecast vs the observed was over or under a given threshold. We used 2 thresholds, representing moderate and strict accuracy. This was defined as 20% of the sample mean for moderate accurate and 10% of the sample mean for strict accuracy. These thresholds match the ad-hoc thresholds determined in meetings with NBT, namely a 10/20 bed threshold for all admissions, which is matched by a 9.4/18.8 bed threshold as mean percentages. Accuracy is thus represented as a percentage of accurate daily forecasts within those thresholds (see table 3). A short-term 6 week (45 day) forecast horizon was chosen, as long term daily forecasts require different methods, as eventually the autoregressive component of the model will converge to the mean [26].

For peer review only

For peer review only - http://bmjopen.bmj.com/site/about/guidelines.xhtml

The data were prepared using Stata 15.1, and analysed using the *forecast* package in R. Following the data checks, the *auto.arima* function in R was used to determine the model specifications[27]. To determine the relevance of the explanatory variables, once the model specifications were determined, the models were specified with and without the explanatory variables. Models with the explanatory variable of interest were compared to null models and models containing the other explanatory variables on the basis of AIC, similar to how *auto.arima* determines model specifications[27]. Admissions and bed occupancy were modelled separately. The first two models were all admissions and all bed occupancy data. Then, subforecasts were modelled, split by specialty or length of stay, and finally specialty and length of stay (see table 2).

Patient and public involvement statement: Members of the public were consulted in the development of this study. The research idea was presented to them in a workshop and suggestions and comments were incorporated in the protocol. Feedback during the workshop was positive, with participants agreeing with the research objectives and the identified need for forecasting models. The use of generic and generalisable models were highlighted in the discussion, and that the audience and end users perspectives should be considered in the development of the model and tool.

Results

18 separate ARIMA models were forecast for the variable specifications described in Table 1, and the subsets described in table 2. The final ARIMA specification, accuracy, RMSE, and MAPE of the models can be seen in table 3. Day of the week, holiday days, temperature, and precipitation were all found to improve the accuracy of the forecasts and subforecasts. The subsets' mean and standard deviation are also provided. Models with more training data, i.e. higher sample size, were more accurate.

The overall admissions model was accurate at the moderate threshold 88.9% of the time, and at the strict threshold 60.0% of the time. Bar the surgical and under 48 hour length of stay subforecasts, 82.2% of daily predictions of admissions were within the moderate admission threshold; 51.1% were within the strict accuracy admission threshold. Models for surgical admissions performed less well, with the under 48 hour length of stay surgical admissions having accuracy of 31.1% on the moderate level, and 11.1% on the strict level. This is likely due to sample size, and high variation in daily admissions.

For bed occupancy, the models generally performed more poorly. For the moderate bed threshold, all models were at least 28.9% accurate, and for the strict bed threshold, the lowest accuracy was 15.6%. However, this, similar to admissions, was dependent on specialty and length of stay. The bed occupancy model overall (model 10) performed rather poorly, and was accurate within the moderate threshold77.8% of the time, and within the strict threshold 40.0 % of the time. Surgery performed better as a specialty, and under 48 hour length of stay tended to outperform over 48 hour length of stay as well. Appendix 1 shows the subforecasts for each model in table 3, with 95% confidence intervals.

Table 3: Model specifications and accuracy

Model	Outcome	(Sub)for-ecast	SARIMA (p,d,q)(P,D,Q)	Model accuracy (strict/moderate thresholds)	MAPE	RMSE	Mean of Outcome	SD of outcome

For peer review only - http://bmjopen.bmj.com/site/about/guidelines.xhtml

1	Admissions	All data overall	(3,0,0)(1,0,0)	60.0%/88.9%	9.59	8.66	94.04	14.98
2	Admissions	All medical	(3,0,2)(,2,0,2)	51.1%/84.4%	8.98	9.44	79.14	19.32
3	Admissions	All surgical	(1,0,2)(0,0,0)	28.9%/57.8%	4.59	19.86	20.65	5.31
4	Admissions	All under 48 hour length of stay	(1,0,0)(1,0,0)	28.9%/71.1%	6.81	16.53	35.38	9.06
5	Admissions	All over 48 hour length of stay	(3,0,1)(1,0,1)	62.2%/88.9%	7.22	10.78	55.14	10.04
6	Admissions	Under 48 hour length of stay, medical	(4,0,0)(2,0,0)	24.4%/48.9%	6.45	18.64	30.59	10.21
7	Admissions	Under 48 hour length of stay, surgical	(0,01)(0,0,0)	11.1%/31.1%	7.82	44.19	20.99	8.39
8	Admissions	Over 48 hour length of stay, medical	(5,0,1)(2,0,0)	53.3%/82.2%	6.88	11.97	48.55	12.06
9	Admissions	Over 48 hour length of stay, surgical	(3,0,3)(1,0,2)	25.8%/46.7%	3.4	27.45	11.87	3.74
10	Bed Occupancy	All data overall	(0,0,1)(1,0,0)	40.0%/77.8%	158.24	15.57	823.36	165.22
11	Bed Occupancy	All medical	(4,0,0)(2,0,0)	37.8%/60.0%	151.69	17.06	760.03	189.22
12	Bed Occupancy	All surgical	(0,0,1)(0,0,0)	24.4%/37.8%	64.5	35.4	162.62	67.71
13	Bed Occupancy	All under 48 hour length of stay	(3,0,0)(2,0,1)	28.9%/66.7%	15.72	16.41	82.3	21.06

For peer review only - http://bmjopen.bmj.com/site/about/guidelines.xhtml

14	Bed Occupancy	All over 48 hour length of stay	(0,0,1) (1,00)	40.0%/75.6 %	159.55	17.6	767.41	177.2
15	Bed Occupancy	Under 48 hour length of stay, medical	(4,0,0) (2,0,0)	22.2%/48.9 %	14.95	18.63	70.83	23.95
16	Bed Occupancy	Under 48 hour length of stay, surgical	(0,0,1) (0,0,0)	15.6%/28.9 %	7.82	44.19	21	8.38
17	Bed Occupancy	Over 48 hour length of stay, medical	(2,0,1) (0,0,0)	35.6%/57.8 %	152.7	19.15	673.24	181.24
18	Bed Occupancy	Over 48 hour length of stay, surgical	(0,0,1) (0,0,0)	20.0%/35.6 %	64.95	44.22	141.63	67.49

Our forecasts were fairly close to the actual values observed in the test data. Our prediction for admissions overall, for example, which can be seen in table 4, was within the moderate threshold (18.8), excepting 5 days on our 45-day horizon. 15 days were between the moderate and strict (9.4) thresholds. 25 days were under the strict accuracy threshold. The model was less accurate the farther forward in time from the beginning of the horizon. Further, possibly due to the impact of the coronavirus pandemic, the latter part of the horizon may have different patterning with respect to hospital admissions. This can be seen in figure 1 (Model 1 predictions), with the actual value of admissions dropping at the end of the horizon. The 95% confidence intervals range from about 67 to 135 beds wide.

Figure 1: ARIMA forecast for overall admissions to NBT

figure 1 goes here

Compared to NBT's in-house prediction modelling for overall admissions, the ARIMA model outperformed them in terms of accuracy apart from two days, one which the NBT model was closer by just 1.5 beds, and another where the NBT model was closer by 10 beds. The mean difference between the ARIMA models and the actual admissions value was 7.3 beds, with a standard deviation of 9.9 beds. The median difference for the ARIMA models was 6 beds. For the NBT prediction models, the mean difference from the actual value was 21 beds, with a standard deviation of 11.1 beds, and a median difference of 21 beds. Finally, the correlation between the ARIMA prediction and the actual values was 0.75, whereas for the NBT prediction, it was -0.45. Therefore, the ARIMA models are more accurate and more precise than the NBT models.

For peer review only - http://bmjopen.bmj.com/site/about/guidelines.xhtml

Table 4: Forecast predictions for overall admissions (model 1) Unshaded is ARIMA predicted within 9.4 (strict threshold) beds of the actual value, light grey is ARIMA predicted within 18.8 (moderate threshold) beds of the actual, grey is ARIMA predicted outside of 18.8 beds of the actual, and dark grey is where the NBT prediction was better than the ARIMA one.

Date	Day of the week	Actual	NBT prediction	Difference NBT – actual	ARIMA Predicted	Difference ARIMA - actual	95% CI	
2020-01-30	Thurs	104	116	12	106.99	2.99	85.99	131.56
2020-01-31	Fri	118	128	10	109.29	-8.71	87.79	134.45
2020-02-01	Sat	71	93	22	85.61	14.61	68.76	105.34
2020-02-02	Sun	91	101	10	83.99	-7.01	67.39	103.45
2020-02-03	Mon	99	122	23	108.29	9.29	86.88	133.37
2020-02-04	Tues	105	132	27	112.48	7.48	90.25	138.54
2020-02-05	Weds	104	107	3	108.52	4.52	87.07	133.66
2020-02-06	Thurs	81	114	33	107.87	26.87	86.54	132.86
2020-02-07	Fri	105	130	25	109.33	4.33	87.71	134.67
2020-02-08	Sat	87	93	6	85.65	-1.35	68.71	105.49
2020-02-09	Sun	78	97	19	83.39	5.39	66.9	102.72
2020-02-10	Mon	113	120	7	108.52	-4.48	87.06	133.67
2020-02-11	Tues	101	132	31	112.84	11.84	90.53	138.99
2020-02-12	Weds	95	115	20	109.12	14.12	87.54	134.4
2020-02-13	Thurs	131	118	-13	108.18	-22.82	86.79	133.25
2020-02-14	Fri	108	128	20	109.77	1.77	88.07	135.21
2020-02-15	Sat	81	97	16	86.07	5.07	69.05	106.01
2020-02-16	Sun	71	97	26	83.28	12.28	66.81	102.57
2020-02-17	Mon	99	120	21	108.25	9.25	86.85	133.34
2020-02-18	Tues	110	132	22	112.74	2.74	90.45	138.87
2020-02-19	Weds	103	121	18	109.17	6.17	87.59	134.47

For peer review only - http://bmjopen.bmj.com/site/about/guidelines.xhtml

2020-02-20	Thurs	106	121	15	108.02	2.02	86.66	133.05
2020-02-21	Fri	104	135	31	109.14	5.14	87.56	134.42
2020-02-22	Sat	80	94	14	86.23	6.23	69.18	106.22
2020-02-23	Sun	87	98	11	83.69	-3.31	67.14	103.09
2020-02-24	Mon	95	125	30	108.5	13.5	87.04	133.64
2020-02-25	Tues	99	136	37	112.86	13.86	90.54	139.01
2020-02-26	Weds	101	117	16	108.85	7.85	87.32	134.07
2020-02-27	Thurs	92	121	29	107.81	15.81	86.5	132.8
2020-02-28	Fri	114	127	13	109.57	-4.43	87.9	134.96
2020-02-29	Sat	69	94	25	82.8	13.8	66.42	101.98
2020-03-01	Sun	69	92	23	83.52	14.52	67.01	102.88
2020-03-02	Mon	110	121	11	104.16	-5.84	83.56	128.29
2020-03-03	Tues	112	124	12	108.97	-3.03	87.42	134.22
2020-03-04	Weds	101	116	15	104.71	3.71	84	128.97
2020-03-05	Thurs	74	123	49	104.15	30.15	83.55	128.28
2020-03-06	Fri	100	132	32	105.82	5.82	84.9	130.34
2020-03-07	Sat	81	94	13	83.13	2.13	66.69	102.39
2020-03-08	Sun	72	93	21	80.56	8.56	64.63	99.23
2020-03-09	Mon	86	119	33	104.79	18.79	84.07	129.07
2020-03-10	Tues	97	119	22	108.8	11.8	87.29	134.01
2020-03-11	Weds	81	116	35	105.1	24.1	84.32	129.46
2020-03-12	Thurs	88	121	33	104.45	16.45	83.8	128.65
2020-03-13	Fri	88	129	41	109.66	21.66	87.97	135.07
2020-03-14	Sat	71	93	22	86.03	15.03	69.02	105.97

For peer review only - http://bmjopen.bmj.com/site/about/guidelines.xhtml

Discussion

We have extended the literature by producing forecasts for a variety of combinations of specialty and length of stay, for both emergency admissions and bed occupancy, as well as for nonelective admissions beyond those for trauma[20 22 24]. We have found that these models work very well for some but not as well for other types of subsets, specifically shorter lengths of stay and for surgery. The models appear to work better in general for admissions rather than bed occupancy, as well as for shorter lengths of stay. The differences in accuracy can possibly be explained by the beginning of the coronavirus pandemic in the United Kingdom, as can be seen in the test data for several of the subsets, particularly for over 48 hour length of stay, and bed occupancy: a sharp drop can be seen at the beginning of February 2020. In the all data categories, and under 48 hour length of stay this sharp rise and fall can be seen around mid February 2020 (see appendix 1).

The strengths of this study are the use of ARIMA models, which are simple to set up using *R*, and can be used to improve short term forecasts given enough input data. The dataset used is the hospital level precursor to the Hospital Episode Statistics (HES), available through *NHS Digital*[16]. It is possible to improve short-term models from NBT with a relatively simple procedure, which should be transferable to other contexts, due to this similarity. The models can be incorporated easily into practice by sharing the *R* code on Github or a similar platform, and tested on data from other Trusts, which should follow a standard structure, again due to the reporting requirements of HES[16]. The code used to generate the models in this paper is available, with commentary, as a supplementary file (see appendix 2). Weaknesses of this study include the poorer accuracy in subforecasts, which may require more complex methods to mitigate, and the use of only one NHS Trust context. Using only one Trust means that the models are not necessarily validated for different contexts and settings. This means that it is necessary to test them in other Trusts.

Abraham and colleagues [3] found that admissions for patients spending at least one night were predominantly random and difficult to predict, using 3 years worth of training data. However, we found that, in general, over all subsets admissions forecasts specified better than bed occupancy ones, and that forecast horizons of over a week can be produced. It would appear that bed occupancy data are more volatile than admissions. Bed occupancy may be harder to forecast, in that it is driven by admissions, and then length of stay is at times difficult to predict[28]. It is likely that, for subsets of bed occupancy, due to the smaller amount of observations (for example, under 48 hour surgical bed occupancy has a mean of 21.00 compared to 849.71 on the whole), and higher variability of the subsets, that the models for bed occupancy produce less accurate forecasts with higher MAPEs. The MAPE for daily admissions found by Boyle and colleagues[29] is 11%: several of the subforecasts, such as the medicine, surgery, and over 48 hour length of stay ones reach this threshold, but as the amount of observations decreases, the inaccuracy seems to increase. A possible avenue of improvement would be to forecast bed occupancy by levels of severity.

Day of the week has been shown to have reasonably strong predictive capacity in previous work[20 21 25], though the inclusion of climate variables is debated, with no clear answer[8 9 12 21-25]. Calegari and colleagues[8] found that SARIMA models including day of the week, but not climatic variables were the most accurate, especially in complex departments. However, the test period did not comprise the whole year, and the study was located in one hospital in southern Brazil, a different climate to Bristol, England[8]. Similarly, in Brazil, the ambient temperature effect was not found to improve daily visit

For peer review only - http://bmjopen.bmj.com/site/about/guidelines.xhtml

forecasts, though again, the data only were for one season[12]. Sun et al[9] also found that admissions were not predicted by weather, though caution that Singapore, the study setting, has little variation in its weather throughout the year. Therefore context is important, as in climatic data marginally improved our models, similar to other UK studies[23 25], and we suggest its inclusion be considered in future work, echoing recommendations provided by Sahu et al[25].

By improving short term forecasts, care can be better planned, limiting cancellations due to capacity or patient flow issues. Better care can be provided as hospitals will be less crowded. In future, other methods more appropriate for longer horizons can be tested, and medium to long term forecasts can be undertaken with more appropriate methods. We have developed ARIMA models that can forecast emergency admissions over a six week horizon with very good accuracy, which are more precise and more accurate than the current models used by the NBT. More work is needed to inform further model development, as the accuracy of subforecasts for length of stay and specialty varies significantly. This is important work because improved modelling capability will have a direct impact on the business planning of acute NHS Trusts. Improving patient flow through the hospitals greatly improves the working and care environment, reduces A&E crowding and enables hospitals to provide the most appropriate care for patients.

Acknowledgements

This research was supported by the National Institute for Health Research (NIHR) Applied Research Collaboration West (NIHR ARC West). The views expressed in this article are those of the author(s) and not necessarily those of the NIHR or the Department of Health and Social Care. The authors are grateful to the members of the public who contributed through the patient and public involvement process.

Author Statement: EE drafted the manuscript. EE and TJ analysed the data. MTR and TK supervised the project. TJ managed the data. MP conceptualised the methodology and conducted preliminary analysis of the data. MTR is the guarantor of this paper. The public contributed through the patient and public involvement process in the development of the study.

Copyright: I, the Submitting Author has the right to grant and does grant on behalf of all authors of the Work (as defined in the author licence), an exclusive licence and/or a non-exclusive licence for contributions from authors who are: i) UK Crown employees; ii) where BMJ has agreed a CC-BY licence shall apply, and/or iii) in accordance with the terms applicable for US Federal Government officers or employees acting as part of their official duties; on a worldwide, perpetual, irrevocable, royalty-free basis to BMJ Publishing Group Ltd ("BMJ") its licensees.

The Submitting Author accepts and understands that any supply made under these terms is made by BMJ to the Submitting Author unless you are acting as an employee on behalf of your employer or a postgraduate student of an affiliated institution which is paying any applicable article publishing charge ("APC") for Open Access articles. Where the Submitting Author wishes to make the Work available on an Open Access basis (and intends to pay the relevant APC), the terms of reuse of such Open Access shall be governed by a Creative Commons licence – details of these licences and which licence will apply to this Work are set out in our licence referred to above.

Competing interest statement: All authors have completed the ICMJE uniform disclosure form at www.icmje.org/coi_disclosure.pdf and declare: support from HDRUK Better Care Partnership and NIHR ARC West for the submitted work; no financial relationships with any organisations that might have an

For peer review only - http://bmjopen.bmj.com/site/about/guidelines.xhtml

interest in the submitted work in the previous three years; no other relationships or activities that could appear to have influenced the submitted work.

Transparency declaration: The lead author affirms that this manuscript is an honest, accurate, and transparent account of the study being reported; that no important aspects of the study have been omitted; and that any discrepancies from the study as planned (and, if relevant, registered) have been explained[1].

Ethical approval: University of Bristol Faculty Research Ethics Approval was obtained. (ID #109,024)

Funding statement: This study was funded by the HDRUK Better Care Partnership (#6.12). This research was supported by the National Institute for Health Research (NIHR) Applied Research Collaboration West (NIHR ARC West). The views expressed in this article are those of the author(s) and not necessarily those of the NIHR or the Department of Health and Social Care.

Role of sponsor/ Statement of independence of researchers from funders: The funding source and sponsors had no role in the design of this study and will not have any role during its execution, analyses, interpretation of the data, or decision to submit results.

Dissemination declaration: The results of the study are to be shared to the North Bristol NHS Trust.

Data sharing statement: The data used in the study is collected by the NBT as part of their care and support. Sharing of anonymised data with the University of Bristol was underpinned by a data sharing agreement and solely covers the purposes of this study. Data requests can be made through the NBT. Statistical code is available as a supplementary file.

References

1. Altman DG, Moher D. Declaration of transparency for each research article. *BMJ : British Medical Journal* 2013;347
2. Steventon A, Deeny S, Friebel R, et al. Briefing: Emergency hospital admissions in England: which may be avoidable and how? London: The Health Foundation, 2018.
3. Abraham G, Byrnes GB, Bain CA. Short-Term Forecasting of Emergency Inpatient Flow. *IEEE Transactions on Information Technology in Biomedicine* 2009;13(3):380-88.
4. Association BM. NHS pressures – winter analysis. London: British Medical Association, 2018.
5. Office NA. Report by the Comptroller and Auditor General: Reducing emergency admissions. London: National Audit Office, 2018.
6. Falconer C, Keen T. Forecasting and demand in the North Bristol Trust, 2018.
7. Schweigler LM, Desmond JS, McCarthy ML, et al. Forecasting Models of Emergency Department Crowding. *Academic Emergency Medicine* 2009;16(4):301-08.
8. Calegari R, Fogliatto FS, Lucini FR, et al. Forecasting Daily Volume and Acuity of Patients in the Emergency Department. *Computational and Mathematical Methods in Medicine* 2016;2016:1-8.
9. Sun Y, Heng BH, Seow YT, et al. Forecasting daily attendances at an emergency department to aid resource planning. *BMC Emergency Medicine* 2009;9:1.
10. Bilings J, Georghiou T, Blunt I, et al. Choosing a model to predict hospital admission: an observational study of new variants of predictive models for case finding. *BMJ Open* 2013;3:e003352.
11. Boyle J, Wallis M, Jessup M, et al. Regression Forecasting of Patient Admission Data. *30th Annual International Conference of the IEEE Engineering in Medicine and Biology Society* 2008:3819-22.

For peer review only - http://bmjopen.bmj.com/site/about/guidelines.xhtml

12. Marcilio I, Hajat S, Gouveia N. Forecasting Daily Emergency Department Visits Using Calendar Variables and Ambient Temperature Readings. *Academic Emergency Medicine* 2013;20(8):769-77.
13. Wargon M, Guidet B, Hoang TD, et al. A systematic review of models for forecasting the number of emergency department visits. *Emerg Med J* 2009;26:395-99.
14. Jebb A, Tay L, Wang W, et al. Time series analysis for psychological research: Examining and forecasting change. *Frontiers in Psychology* 2015;6:727.
15. Earnest A, Chen MI, Ng D, et al. Using autoregressive integrated moving average (ARIMA) models to predict and monitor the number of beds occupied during a SARS outbreak in a tertiary hospital in Singapore. *BMC Health Services Research* 2005;5:36.
16. Digital N. The HES Processing Cycle and Data Quality. London: Health and Social Care Information Centre, 2016.
17. Hicks M. Horfield and Filton, Bristol, UK Weather Station 2020 [Available from: http://www.martynhicks.uk/weather/data.php accessed 04 Apr 2020.
18. Office M. Daily Southwest England & Wales Precipitation (mm) In: Office M, ed. London, 2020.
19. NHS. Hospital Provider Spell 2020 [Available from: https://www.datadictionary.nhs.uk/data_dictionary/nhs_business_definitions/h/hospital_provider_spell_de.asp#:~:text=An%20admission%20is%20the%20start,Nursing%20Episode%20within%20the%20spell. accessed 20/08/2020 2020.
20. Bhattacharyya T, Millham F. Relationship between Weather and Seasonal Factors and Trauma Admission Volume at a Level I Trauma Center. *The Journal of Trauma and Acute Care Surgery* 2001;51(1):118-22.
21. Holleman Jr DR, Bowling RL, Gathy C. Predicting daily visits to a walk-in clinic and emergency department using calendar and weather data. *J Gen Intern Med* 1996;11(4):237-39.
22. Atherton W, Harper W, Abrams K. A year's trauma admissions and the effect of the weather. *Injury* 2005;36(1):40-46.
23. Macgregor D. Effect of weather on attendance with injury at a paediatric emergency department. *Emerg Med J* 2003;20:204-05.
24. Rising W, O'Daniel J, Roberts C. Correlating Weather and Trauma Admissions at a Level I Trauma Center. *The Journal of Trauma and Acute Care Surgery* 2006;60(5):1096-100.
25. Sahu S, Baffour B, Harper P, et al. A hierarchical Bayesian model for improving short-term forecasting of hospital demand by including meteorological information. *Journal of the Royal Statistical Society Series A (Statistics in Society)* 2014;177(1):39-61.
26. Hyndman RJ, Athanasopoulos G. Forecasting: principles and practice. Melbourne: OTexts, 2018.
27. Hyndman RJ. auto.arima 2020 [Available from: https://www.rdocumentation.org/packages/forecast/versions/8.12/topics/auto.arima accessed 20/08/2020 2020.
28. Predicting Hospital Length of Stay (PHLOS): A Multi-tiered Data Mining Approach. 2012 IEEE 12th International Conference on Data Mining Workshops; 2012 10-10 Dec. 2012.
29. Boyle J, Jessup M, Crilly J, et al. Predicting emergency department admissions. *Emerg Med J* 2012;29:358-65.

For peer review only - http://bmjopen.bmj.com/site/about/guidelines.xhtml

For peer review only

For peer review only - http://bmjopen.bmj.com/site/about/guidelines.xhtml

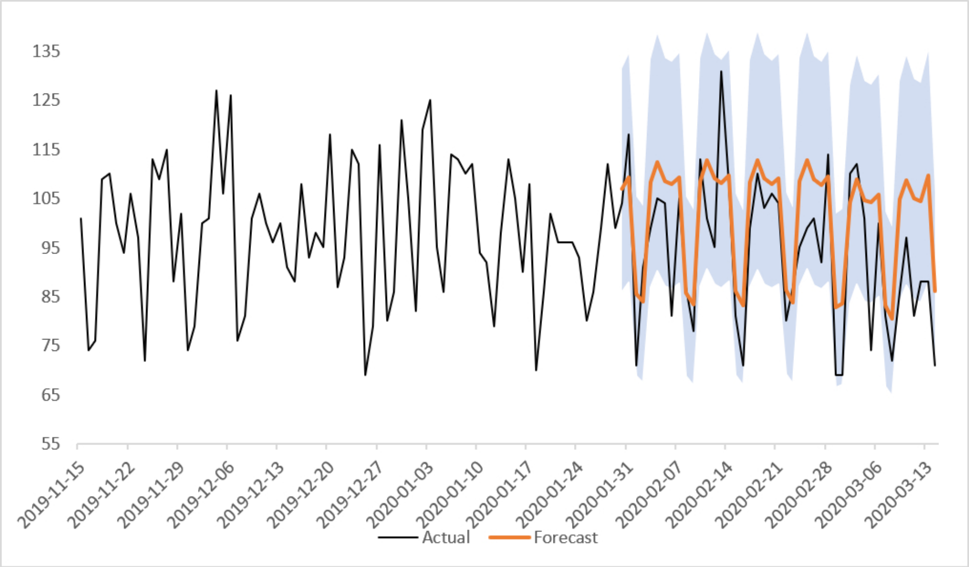

566x330mm (47 x 47 DPI)

Appendix 1: Subforecast figures

Figure A1: Model 2 (medicine admissions)

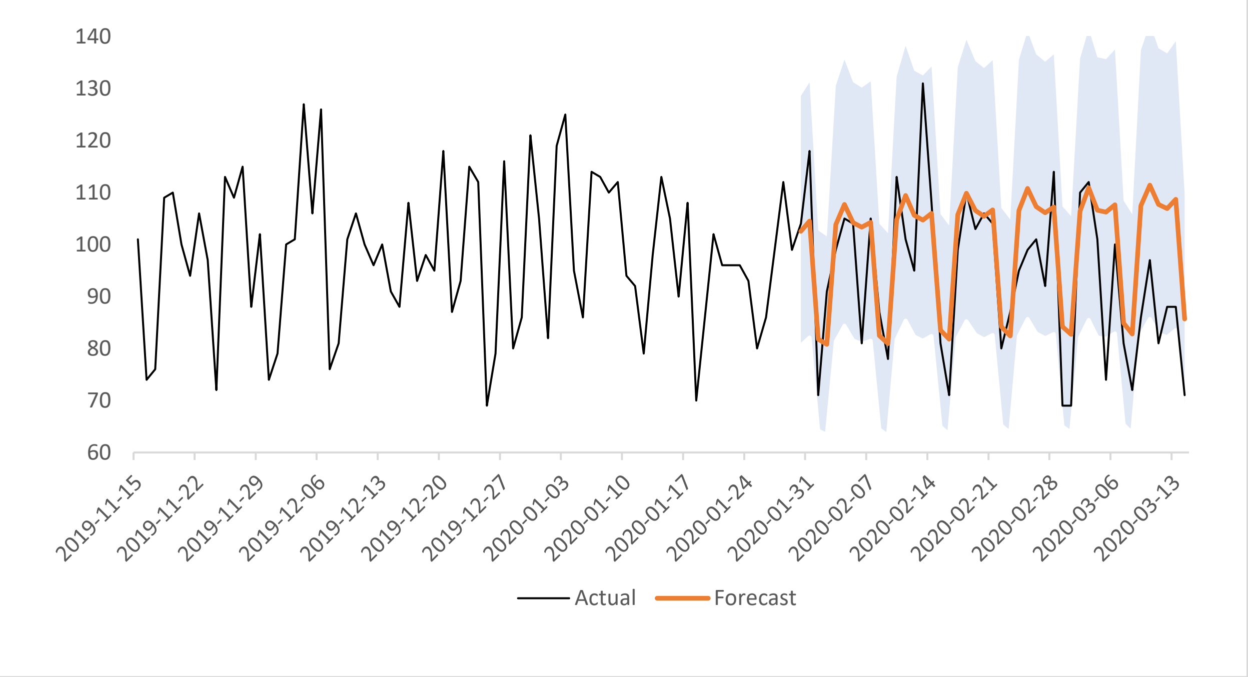

Figure A2: Model 3 (surgery admissions)

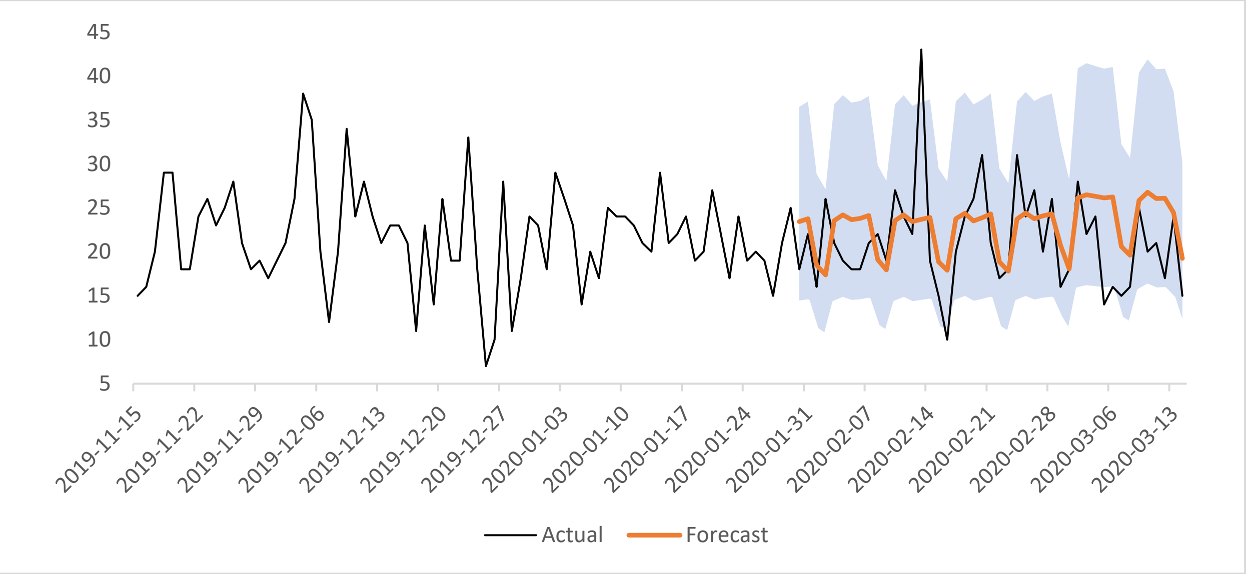

Figure A3: Model 4 (under 48 hour length of stay admissions)

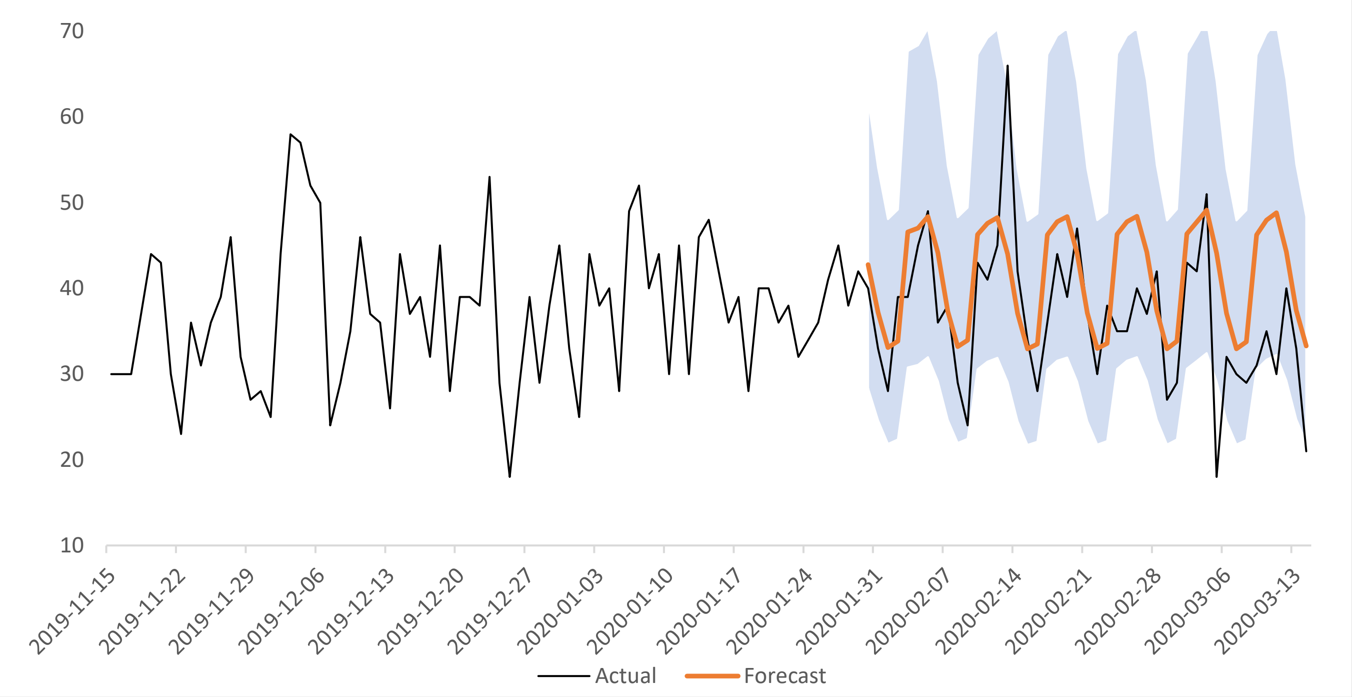

Figure A4: Model 5 (over 48 hour length of stay admissions)

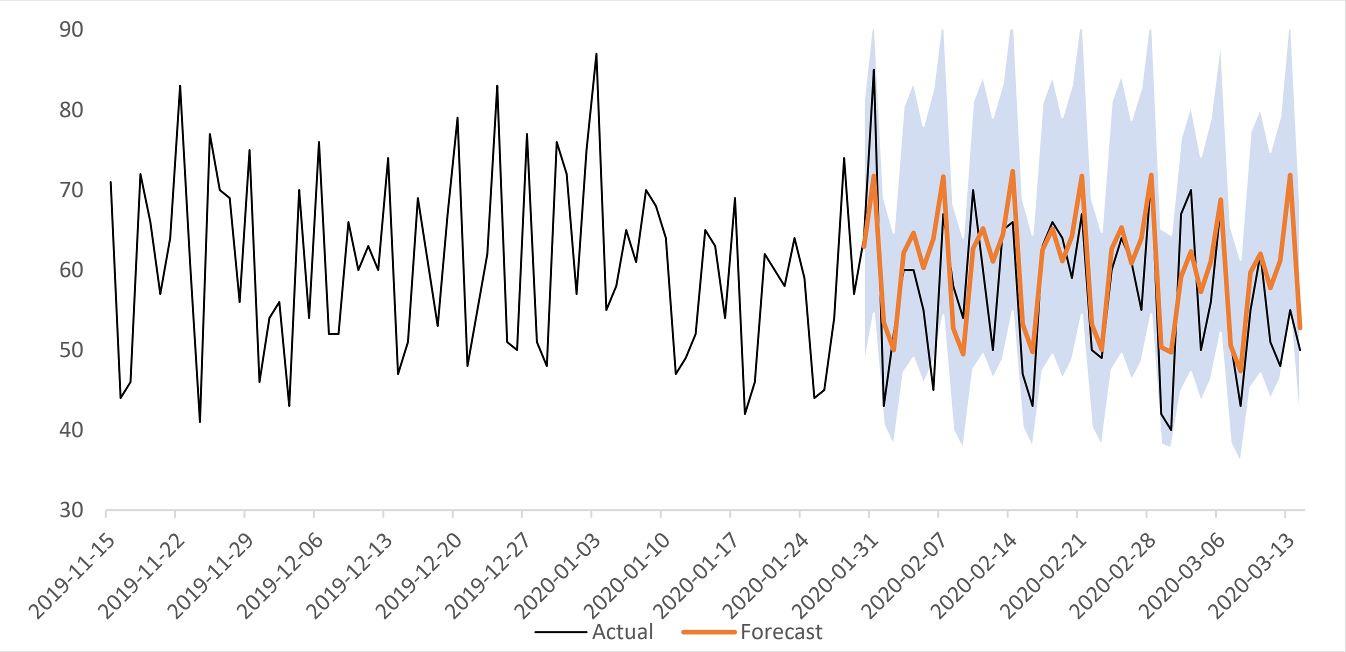

Figure A5: Model 6 (under 48 hour length of stay medicine admissions)

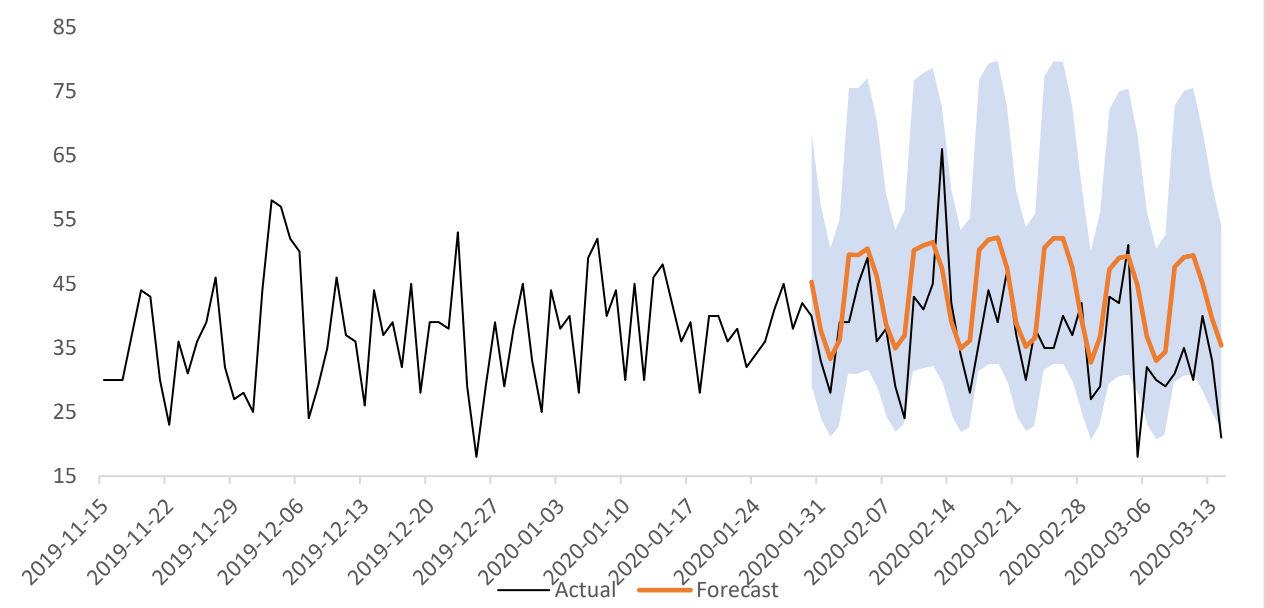

Figure A6: Model 7 (under 48 hour length of stay surgery admissions)

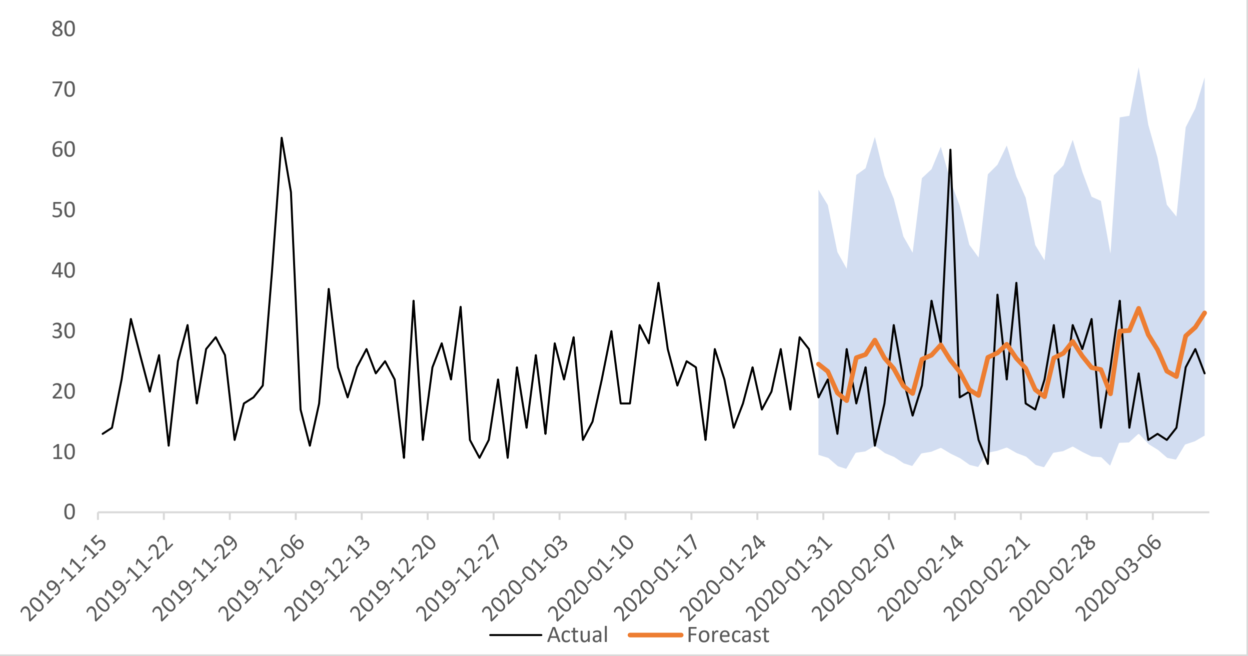

Figure A7: Model 8 (over 48 hour length of stay medicine admissions)

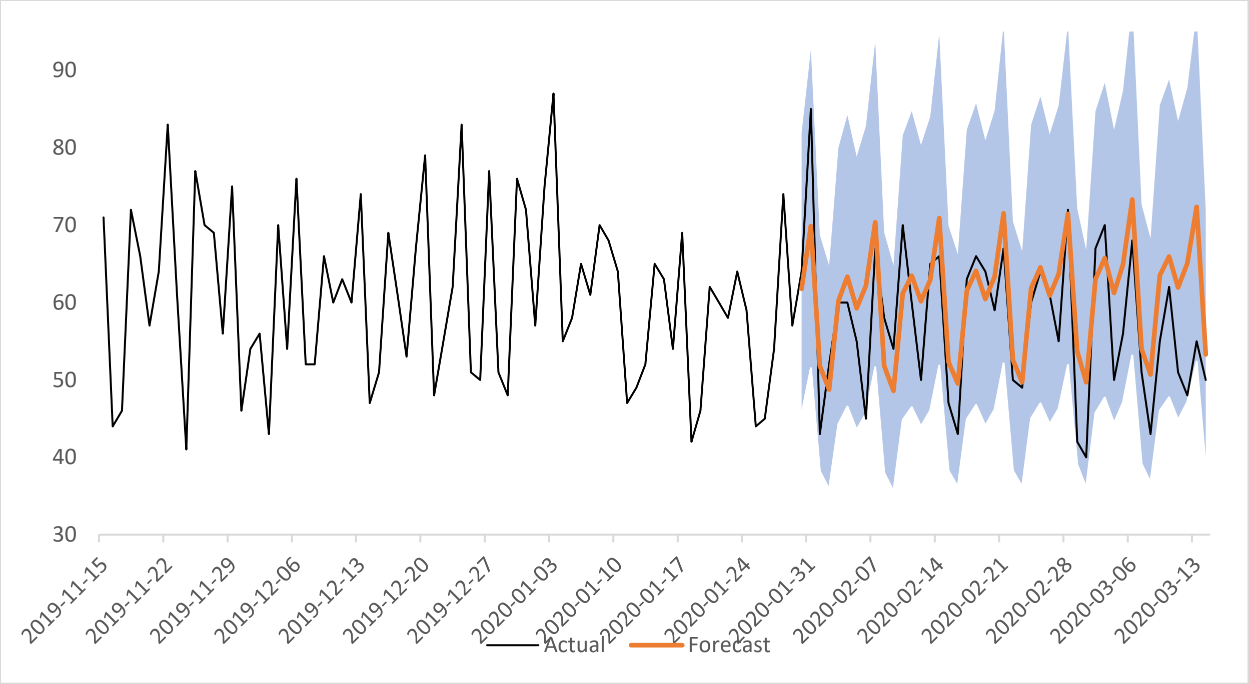

Figure A8: Model 9 (over 48 hour length of stay surgery admissions)

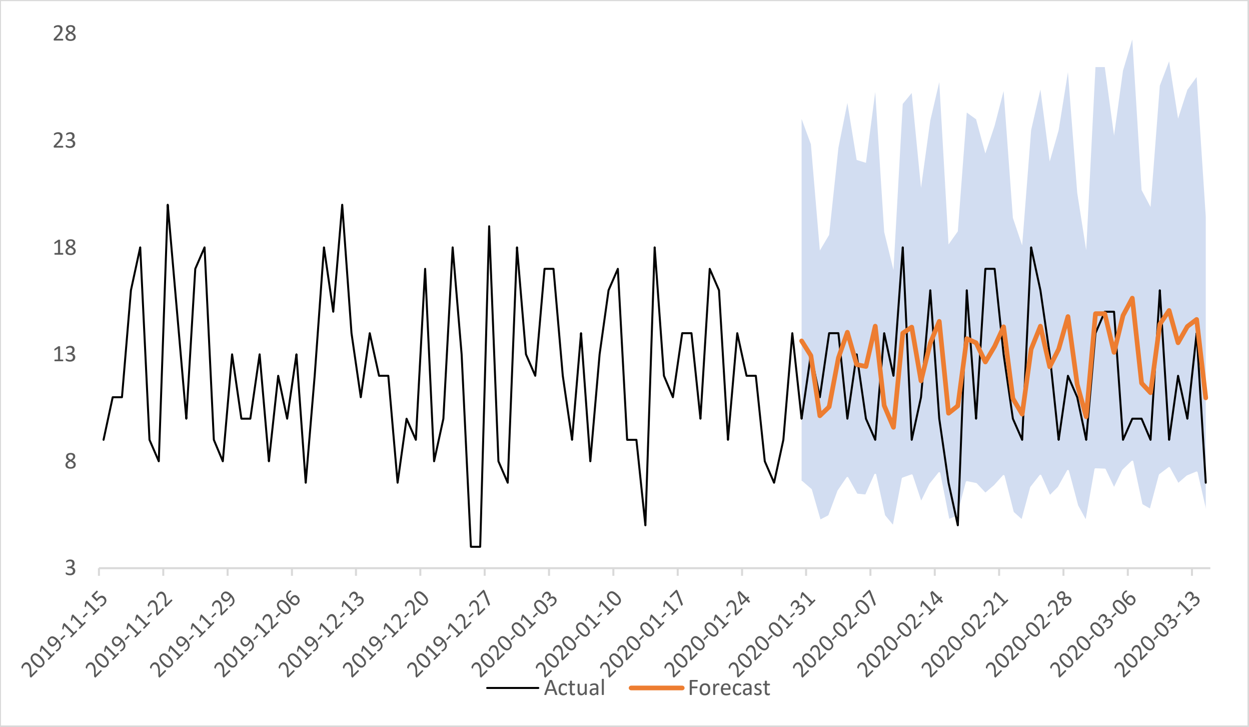

Figure A9: Model 10 (all bed occupancy)

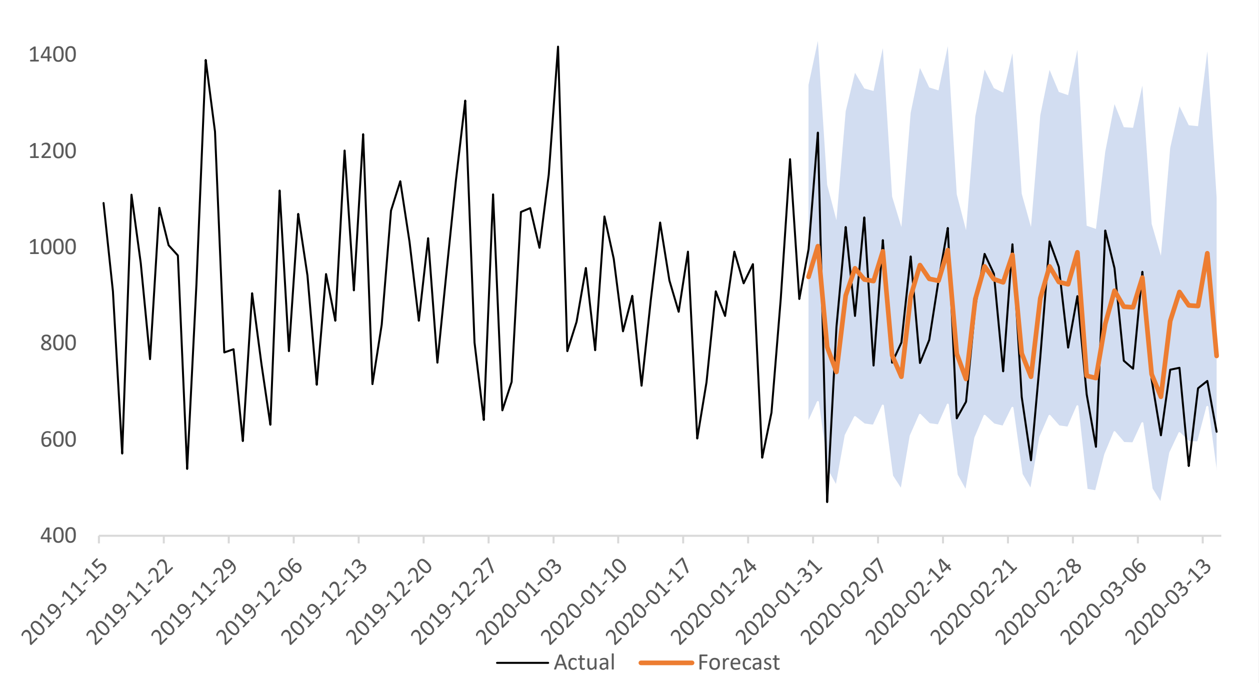

Figure A10: Model 11 (medicine bed occupancy)

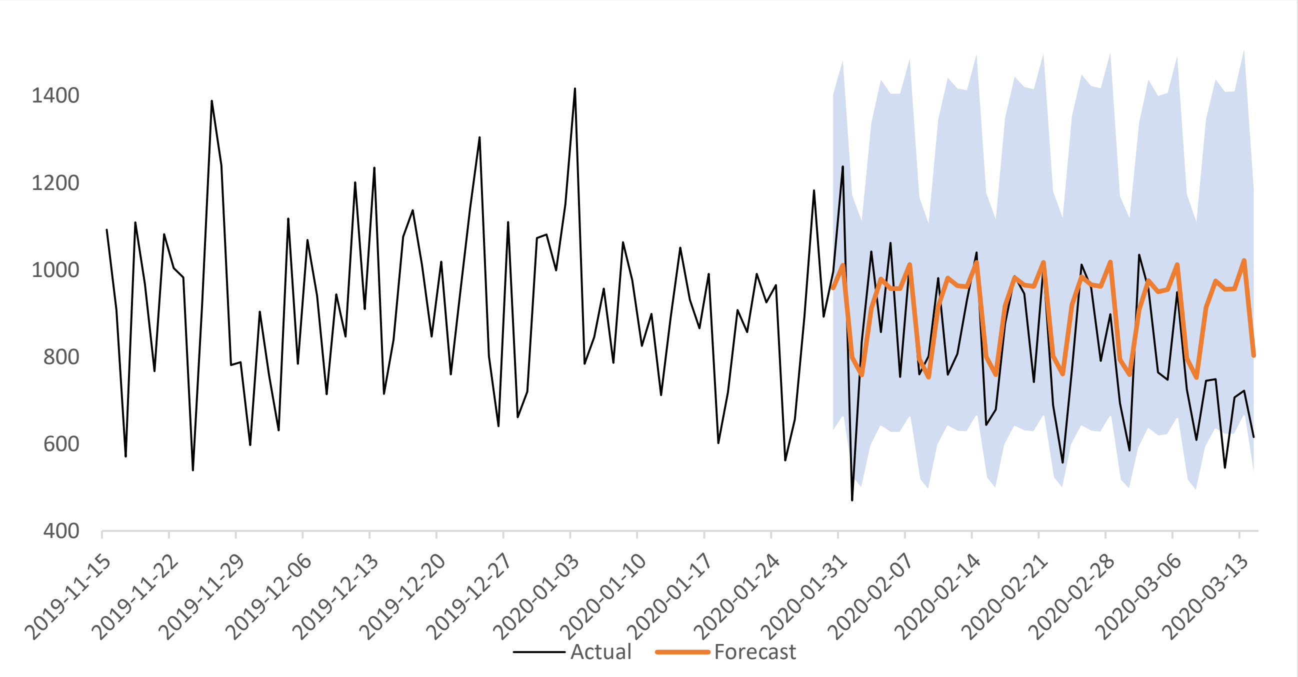

Figure A11: Model 12 (surgery bed occupancy)

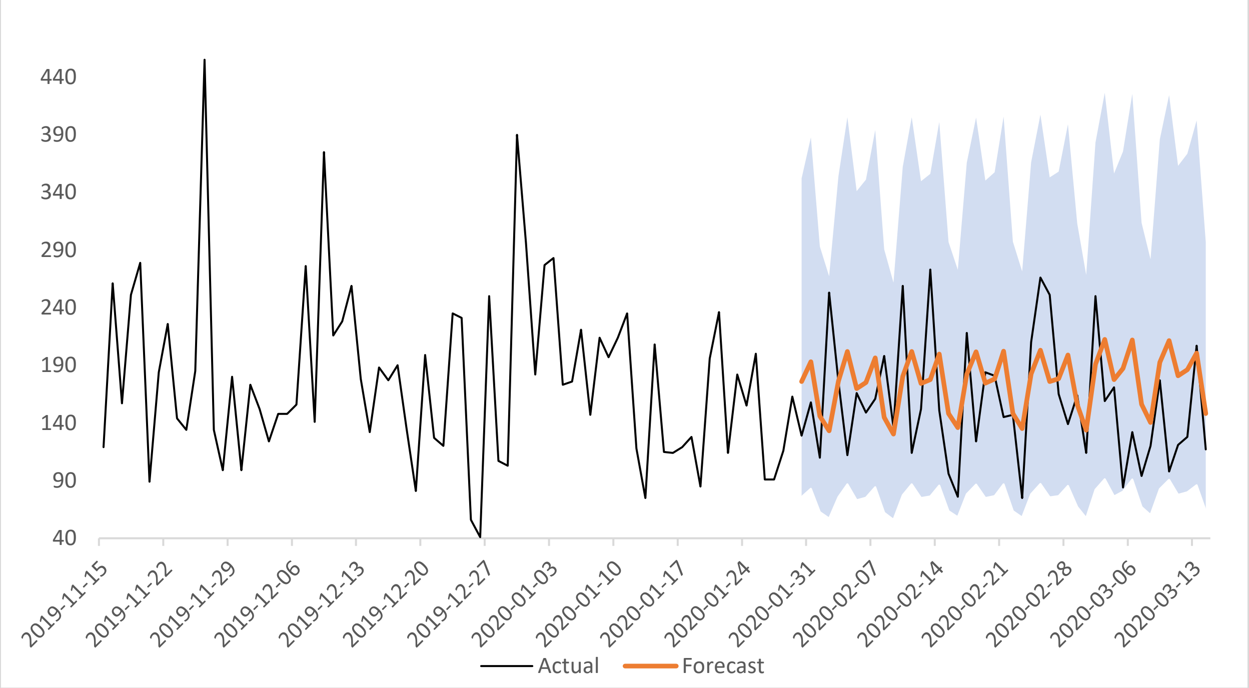

Figure A12: Model 13 (under 48 hour length of stay bed occupancy)

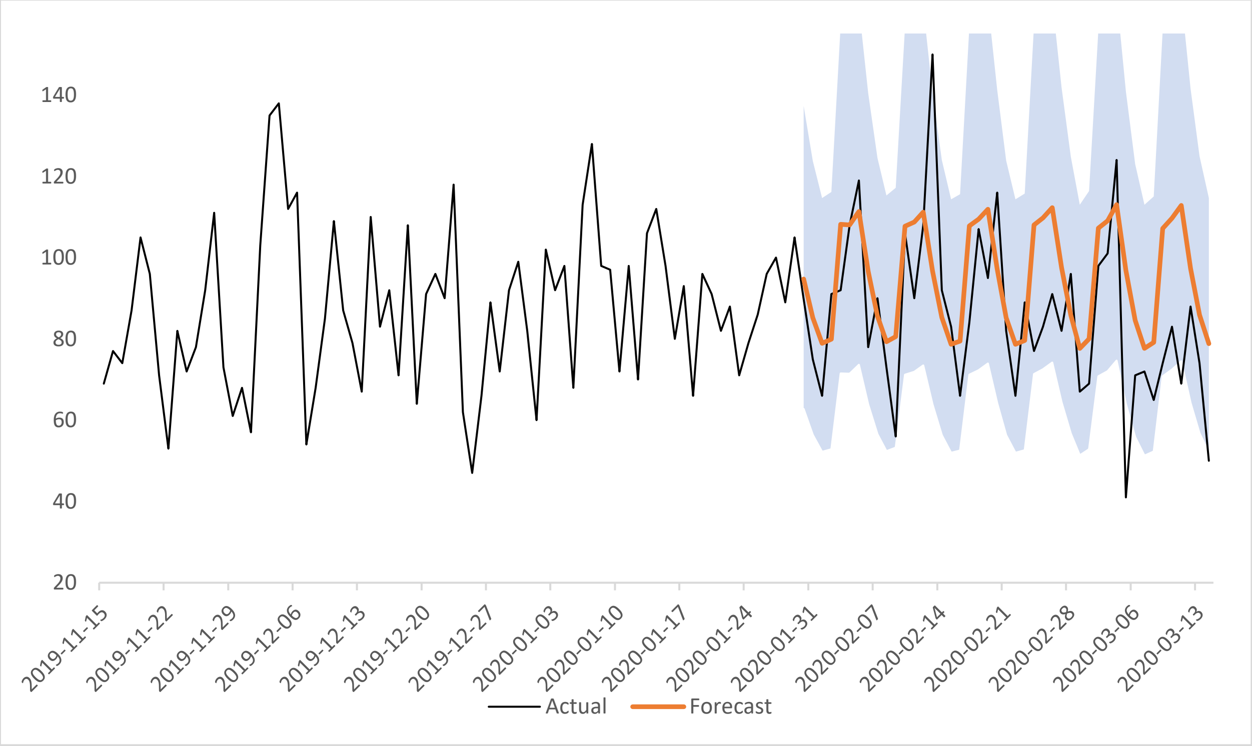

Figure A13: Model 14 (over 48 hour length of stay bed occupancy)

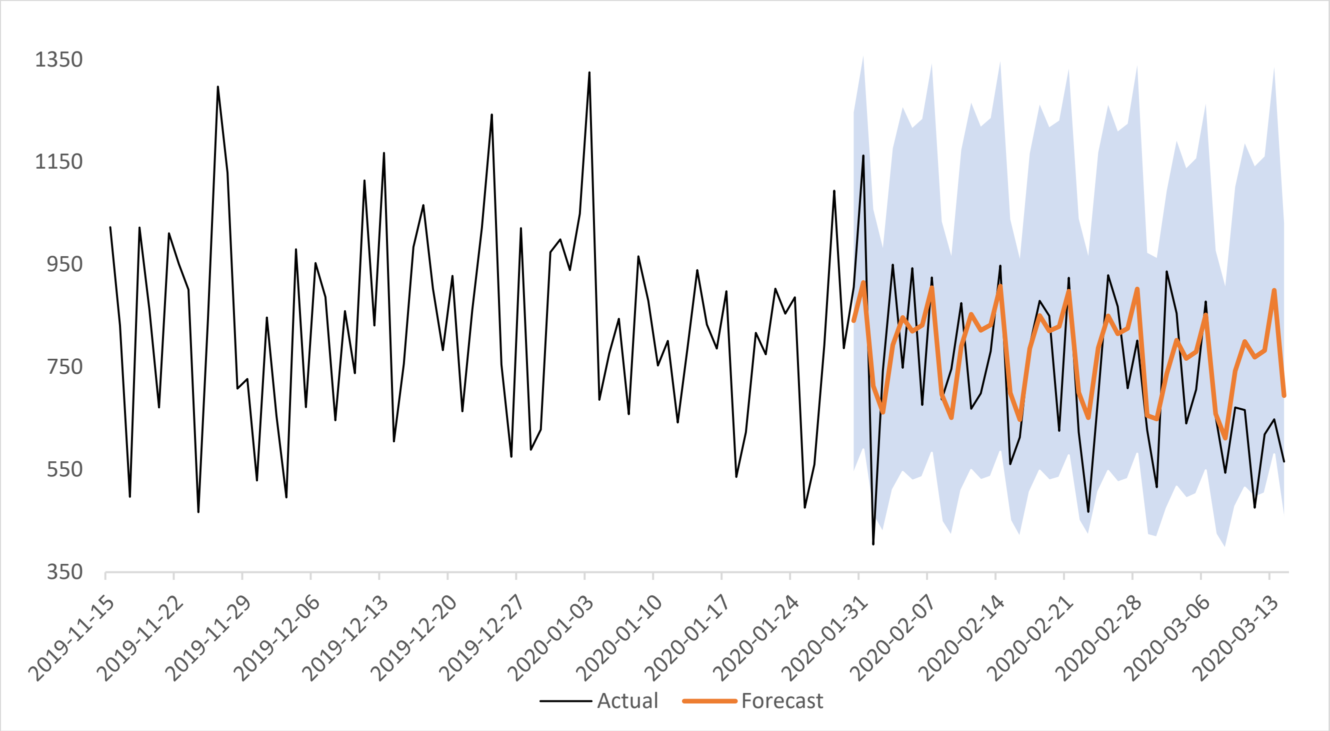

Figure A14: Model 15 (under 48 hour length of stay medicine bed occupancy)

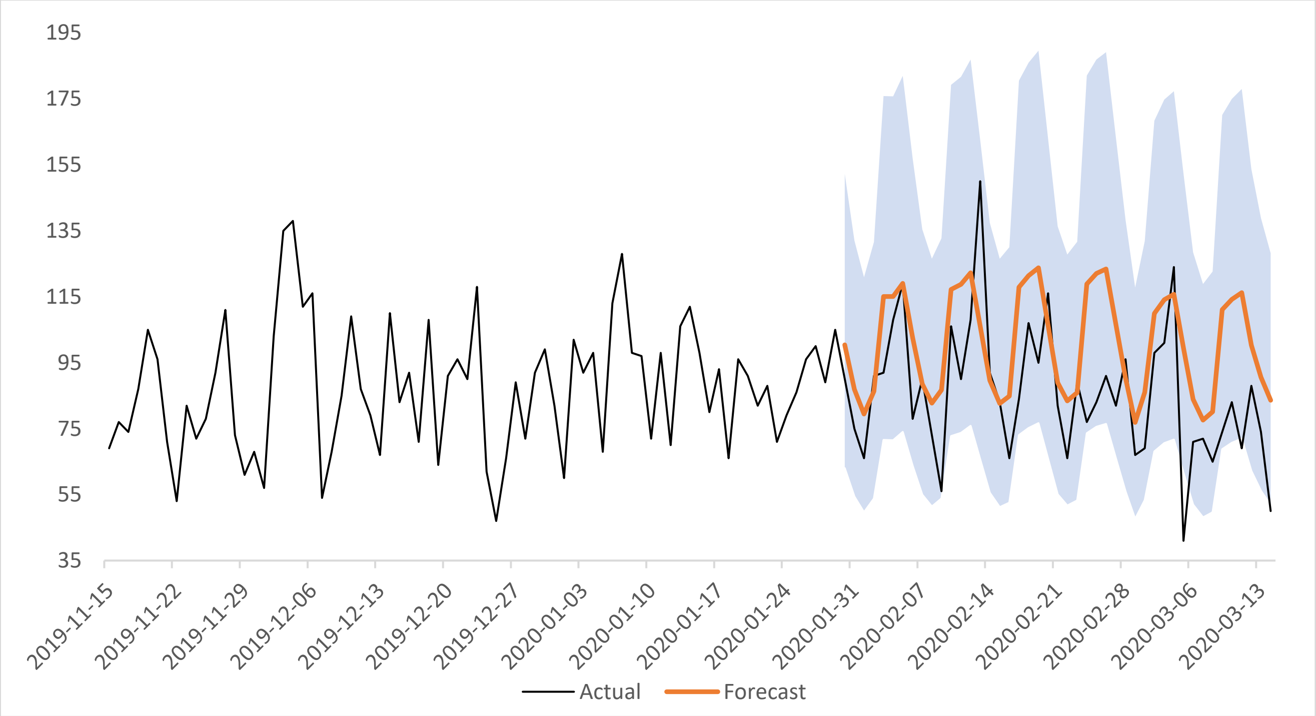

Figure A15: Model 16 (under 48 hour length of stay surgery bed occupancy)

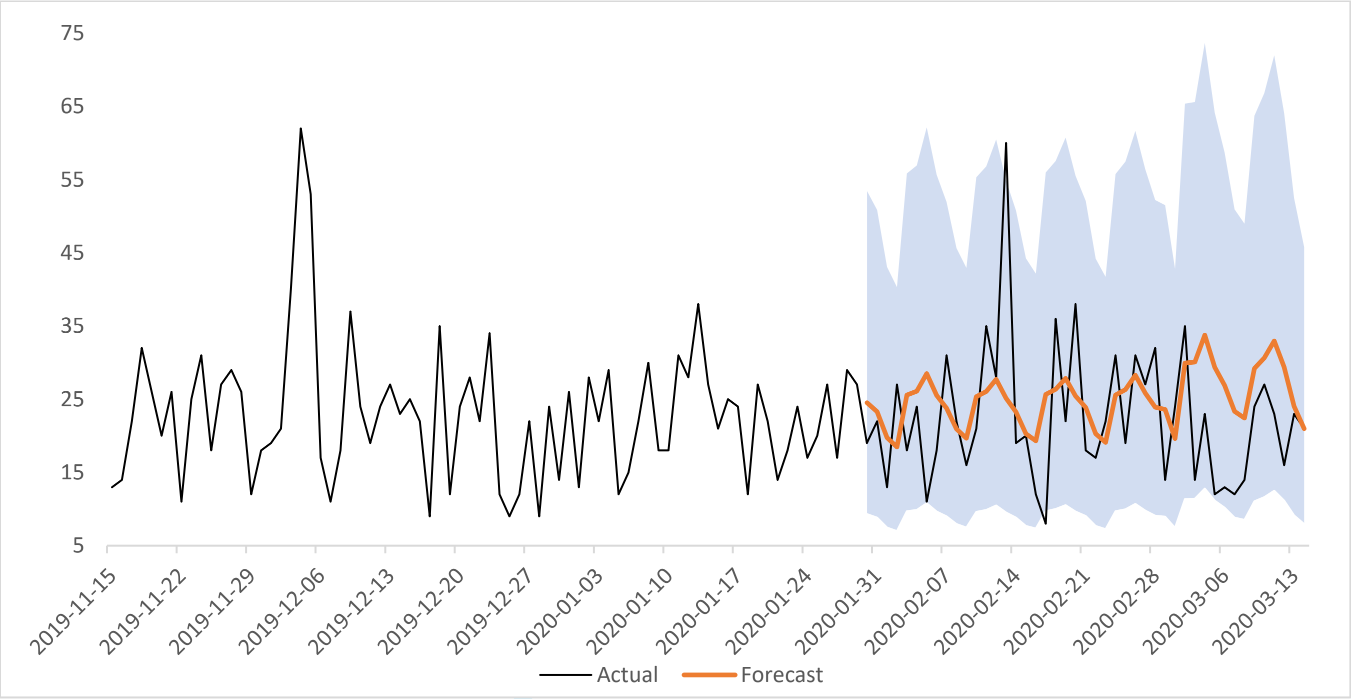

Figure A16: Model 17 (over 48 hour length of stay medicine bed occupancy)

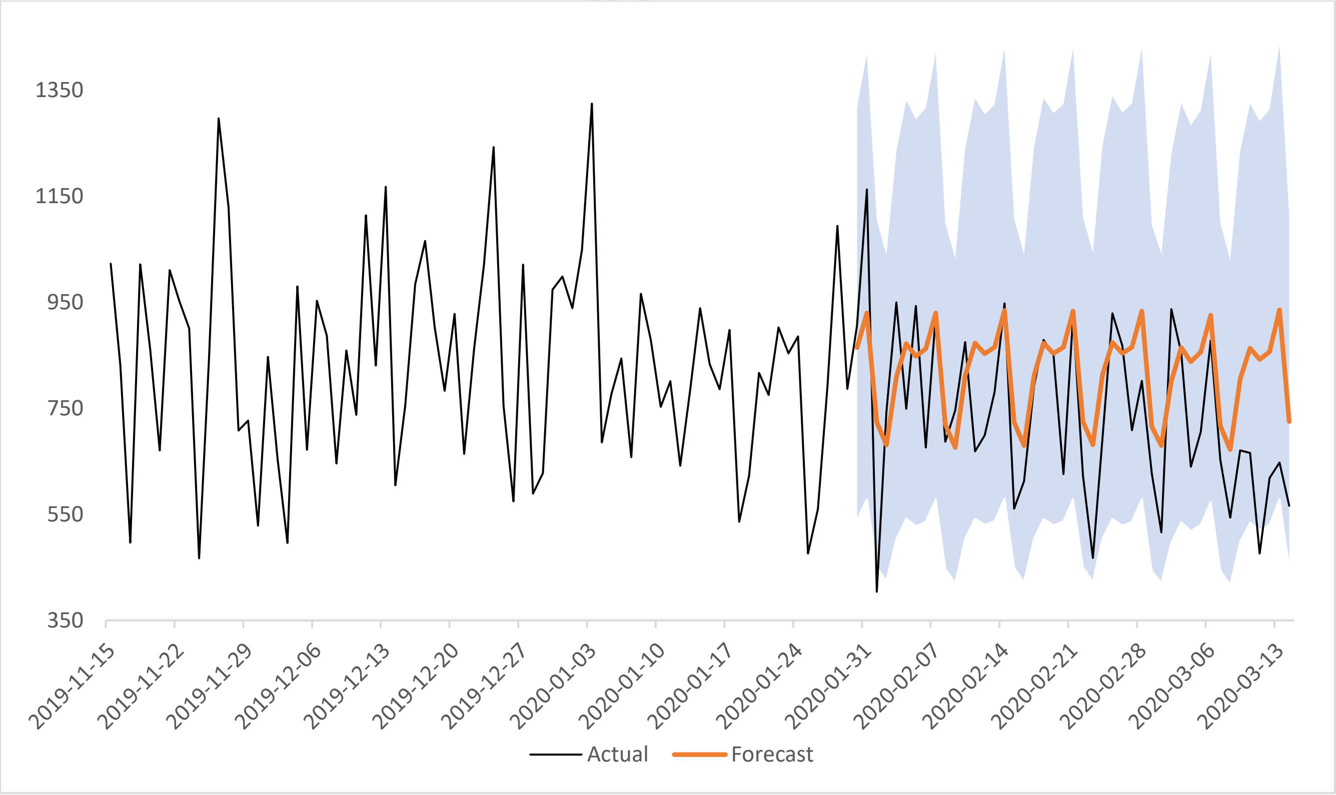

Figure A17: Model 18 (over 48 hour length of stay surgery bed occupancy)

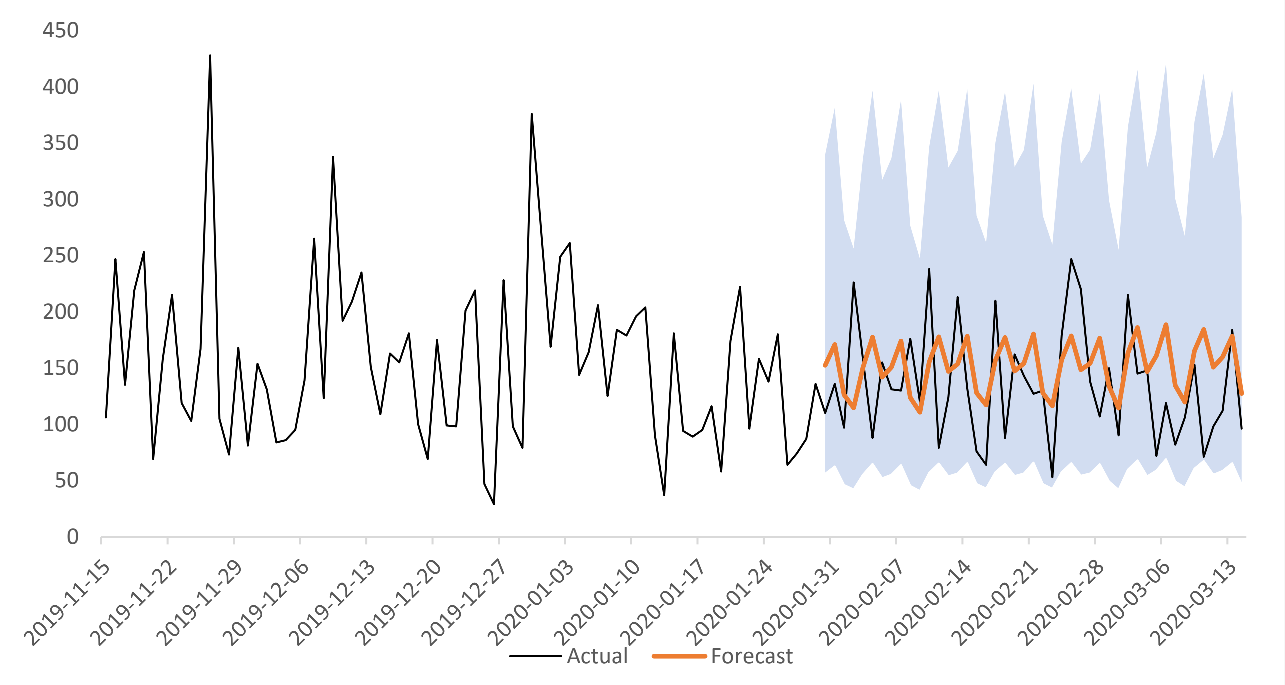

```
#clear the environment of all data
rm(list = ls())

## install necessary packages (Can skip this step if you have them)
install.packages("readstata13", "Hmisc", "DataCombine", "lmtest","tidyverse",
"scales", "forecast", "zoo", "tempdisagg", "imputeTS", "lubridate", "aTSA",
"tseries", "gridExtra", "broom", "knitr", "dplyr", "pixedust")


#tidy regression output libraries
library(broom)
library(knitr)
require(broom)
require(knitr)
library(dplyr) ## this is the library with 'pipes' %>%
library(pixiedust)



## Packages needed
## If you don't have a package, use 'install.packages("NAME OF PACKAGE HERE")
#bring in stata data
library(readstata13)

#lag function
library(Hmisc)

#Row insertion library for missing data problem
library(DataCombine)

#Some tests are in these libraries
library(lmtest)


#tidyverse includes many other useful libraries for formatting data, scales
includes date formatting
library(tidyverse)
library(scales)

#forecasting and time series libraries
library(forecast)
library(zoo)
library(tempdisagg)
library(imputeTS)
library(lubridate)
library(aTSA)
library(tseries)

#laying out graph library
library(gridExtra)
```

For peer review only

```
#tidy regression output libraries
library(broom)
library(knitr)
require(broom)
require(knitr)
library(dplyr) ## this is the library with 'pipes' %>%
library(pixiedust)


# Set file path for forecasting data

setwd("YOUR WORKING DIRECTORY HERE")


# Transfer Stata dataset into R datasets. Use the relevant dataset in 'hosp_data'
hosp_data = read.dta13("COLLAPSED HOSPITAL DATA HERE", convert.factors = TRUE,
generate.factors = TRUE, nonint.factors = TRUE)
temp = read.csv("TEMPERATURE WITH SUFFICIENT LAG (ONE YEAR BEFORE HOSPITAL DATA
START) HERE")
precip = read.dta13("PRECIPITATION WITH SUFFICIENT LAG (ONE YEAR BEFORE HOSPITAL
DATA START) HERE", convert.factors = TRUE, generate.factors = TRUE, nonint.factors
= TRUE)

#Use below if temperature/precipitation data is in dta format from stata, but new
code to combine in R is much more efficient.
#temp = read.dta13("temperatures_lag.dta", convert.factors = TRUE, generate.factors
= TRUE, nonint.factors = TRUE)
#precip = read.dta13("temperatures_lag.dta", convert.factors = TRUE,
generate.factors = TRUE, nonint.factors = TRUE)

#make a full date variable for the temp and precip data to match hosp_data
temp = temp %>%
  mutate(admidate=make_date(year, month, day))

precip = precip %>%
  mutate(admidate=make_date(admiyear, admimonth, admiday))


#check the plot to make sure it looks like you would expect (time series data, with
a count per day)

plot(hosp_data$id)


#pull out the length and the end date of the hospital data, for data manipulation
purposes

leng = nrow(hosp_data)
```

For peer review only

```
end_date=hosp_data$dateindex[leng]
init_date=hosp_data$dateindex[1]


#check the data if there's a huge outlier (often for some of the bed occupancy
subforecasts)

plot(hosp_data$id)

#find the position of the outlier, then make it an average of the previous week's
occupancy/admissions.
#CHECK: max admissions/max occupancy with NBT

outval = which.max(hosp_data$id) #if the outlier is higher
#outval = which.min(hosp_data$id) #if the outlier is lower (this one is commented
out)
hosp_data$id[outval] #displays the value
hosp_data$id[outval] = as.integer(sum(hosp_data$id[(outval-7):(outval-1)])/7)
#makes the outlier an average of the previous 7 days' occupancy/admissions


#sort out the lag temperature - the temperature and precipitation files should have
one year more data in the past, so that you can do this part
#if we didn't do it this way, if we did last week's temperature, we'd have to
predict the weather accurately if our forecast horizon exceeded one week
#in conclusion, since we're not meteorologists, it's probably better to just use
last year's, so we can use whatever horizon we'd like up to a year

temp$last_year = Lag(temp$avg_temp, 365)

#lag precipitation

precip$last_year = Lag(precip$rainfall, 365)

#overwrite the dataframes so that they're the same length as the hospital
admissions data
#the models won't work if the inputs have different lengths

precip= subset(precip, admidate>=init_date & admidate<=end_date)
temp = subset(temp, admidate>=init_date & admidate<=end_date)

#Looking at missingness in the temperature and precipitation data
#Some days may be missing, (NOT IN CURRENT TRANCHE) and missing values need to be
imputed
#If there are no missing values, it will say 'integer (empty)' in the environment

missing_t = which(is.na(temp$last_year))
missing_p = which(is.na(precip$last_year))

#A quick fix for NAs in the temperature and precipitation data, if there
```

For peer review only

```
#are missing values, but don't run if there aren't.

temp$last_year[missing_t] = na.spline(temp$last_year)
precip$last_year[missing_t] = na.spline(precip$last_year)

#Check for missing dates - for subforecasts this might be an issue for those with
small numbers
#Make sure to put 0s in when preparing subforecast data in Stata rather than
allowing missingness
#If there are missing dates, go back to the preparation stage.
#It will say 'Date' num(0) in the global environment if there are no missing dates

## THIS SECTION IS ONLY FOR IF THERE ARE MISSING COUNT DATA ON ANY DAY ##

missing_d = as.Date(setdiff(precip$admidate,hosp_data$dateindex))

#If they are missing, add them in as '0' values (missingness means there was
#no occupancy on those dates, as far as I understand).

#First create a new dataframe with the information that's missing
#Name the columns Add the new dataframe to the end of the old one, then sort
("arrange")

add_miss = data.frame(missing_d, 1, month(missing_d), year(missing_d),
day(missing_d), wday(missing_d), 0)
names(add_miss)= c("dateindex", "id", "month", "year", "day", "dow", "yearmonth")
hosp_data=rbind(hosp_data, add_miss)
hosp_data=hosp_data %>% arrange(dateindex)
rm(add_miss)


leng=nrow(hosp_data)

#tidy up a bit
rm(missing_d,missing_t,missing_p)

#check and record the mean and standard deviation of the data
mean(hosp_data$id)
sd(hosp_data$id)


#Transform the dataframes into time series objects, with a daily 'seasonality'

hospts <- msts(hosp_data, seasonal.periods = c(7,365.25), ts.frequency = 7)
tempts <- msts(temp, seasonal.periods = c(7,365.25), ts.frequency = 7)
precipts <- msts(precip, seasonal.periods = c(7,365.25), ts.frequency = 7)



#Check ACF plot of the count to confirm the presence of autocorrelation.
```

For peer review only

```
#If the values fall between the blue lines (p<0.05), then the data are not
#likely to be autocorrelated. If they do fall outside the lines, they are likely to
be.
#This uses a lag of 21 days
# "The ACF of stationary data drops to zero relatively quickly"
# "The ACF of non-starionary data decreases slowly"

acf(hospts[,"id"], lag = 21)

#Check Partial ACF plot to confirm that the autocorrelation is or isn't a result of
the
#autocorrelation of the first lag carrying through (if it is, the spikes on the
PACF will
#peak at the first lag, then be in between the blue lines (p <0.05))
# "A partial autocorrelation is the amount of correlation between a variable
#and a lag of itself that is not explained by correlations at all
lower-order-lags."
#This uses a lag of 21 days

pacf(hospts[,"id"], lag = 21)

#Fit a regression model to formally test for a lagged autocorrelation in the
residuals

#create a time variable
timef <- seq_along(hospts[,"id"])

#fit a linear regression to model the series as a function of time
lin_reg = tslm(hospts[,"id"] ~ timef)

#Durbin-Watson test - check for lag-1 autocorrelated residuals
#H0 = residuals do not have lag-1 auotocorrelation
#small p -> there is evidence for lag-1 autocorrelation

dwtest(lin_reg)

#tidy up
rm(timef, lin_reg)


#Determine stationarity of the data (it is non-stationary if it has a trend,
seasonal
#effect, or any systematic change in mean and variance


#Seasonal decomposition graphs (including weekly and annual seasonality)
#These are decomposed to the full sample and also financial years

## You may need to adjust these to the years of your data (the label), but the
actual end of the window
```

For peer review only

```
##is one year long##

hospts[,"id"] %>% mstl(lambda = 0) %>% autoplot() + xlab("week") +
  ggtitle("Estimated components (full sample)")

#2015-2016 (check the weeks of the year for these)

hospts[,"id"] %>% mstl(lambda = 0) %>% window(end = 52) %>% autoplot(facet=TRUE) +
xlab("week") +
  ggtitle("Estimated components (2015-2016)")

#2016-2017 (check the weeks of the year for these)

hospts[,"id"] %>% mstl(lambda = 0) %>% window(start = 53, end = 105) %>%
autoplot(facet=TRUE) +
  xlab("week") + ggtitle("Estimated components (2016-2017)")

#2017-2018 (check the weeks of the year for these)

hospts[,"id"] %>% mstl(lambda = 0) %>% window(start = 106, end = 158) %>%
autoplot(facet=TRUE) +
  xlab("week") + ggtitle("Estimated components (2017-2018)")

#2018-2019 (check the weeks of the year for these)

hospts[,"id"] %>% mstl(lambda = 0) %>% window(start = 159, end = 211) %>%
autoplot(facet=TRUE) +
  xlab("week") +ggtitle("Estimated components (2018-2019)")

#2019-'2020' (check the weeks of the year for these -
#keep in mind that we currently don't have all the data for this financial year

hospts[,"id"] %>% mstl(lambda = 0) %>% window(start = 212, end = 250) %>%
autoplot(facet=TRUE) +
  xlab("week") +ggtitle("Estimated components (2019-2020)")

# Check the seasonality of the data - annual seasonality often includes Christmas
(push to discharge before Christmas) and at times Easter
# There is also usually weekly seasonality. Check the trend of the data as well.

#Conduct an augmented Dickey-Fuller test (ADF) test to formally test for
stationarity
#H0 = non-stationary, H1 = stationary. low p value = reject the null

adf.test(hospts[,"id"])

#If the data are not stationary (i.e. high p), transform them to be stationary
(differencing)
#if the adf test rejects the null, then don't difference as they are stationary.
#if you difference once, and it still isn't stationary, consider differencing the
```

For peer review only

```
differenced data
#or, log the data (if the variance is unstable). Currently commented out.

#hospts = diff(hospts)

#Onto modelling, creating the data. Please note if you differenced the data, you
will need to change the variable names:

# Create deterministic trend and log deterministic trend

trend <- seq_along(hospts[,"id"])

# Create dummy daily variables

mon <- ifelse(hospts[,"dow"] == 1, 1, 0) # create dummy variable equal to 1 if
Monday and 0 otherwise
tue <- ifelse(hospts[,"dow"] == 2, 1, 0)
wed <- ifelse(hospts[,"dow"] == 3, 1, 0)
thu <- ifelse(hospts[,"dow"] == 4, 1, 0)
fri <- ifelse(hospts[,"dow"] == 5, 1, 0)
sat <- ifelse(hospts[,"dow"] == 6, 1, 0)

# Create dummmy(ies) for Christmas effect

chris <- ifelse(hospts[,"day"] == 25 & hospts[,"month"] == 12, 1, 0)
chrisweek <- ifelse(hospts[,"day"] %in% c(23, 24, 25, 26, 27) & hospts[,"month"] ==
12, 1, 0)
easter2015 <- ifelse(hospts[,"day"] %in% c(3,4,5,6) & hospts[,"month"] == 4, 1, 0
& hospts[,"year"] == 2015)
easter2016 <- ifelse(hospts[,"day"] %in% c(25,26,27,28) & hospts[,"month"] == 3, 1,
0  & hospts[,"year"] == 2016)
easter2017 <- ifelse(hospts[,"day"] %in% c(14,15,16,17) & hospts[,"month"] == 4, 1,
0  & hospts[,"year"] == 2017)
easter2018a <- ifelse(hospts[,"day"] %in% c(30,31) & hospts[,"month"] == 3, 1, 0  &
hospts[,"year"] == 2018)
easter2018b <- ifelse(hospts[,"day"] %in% c(1,2) & hospts[,"month"] == 4, 1, 0  &
hospts[,"year"] == 2018)
easter2018 <- easter2018a + easter2018b
easter2019 <- ifelse(hospts[,"day"] %in% c(19,20,21,22) & hospts[,"month"] == 4, 1,
0  & hospts[,"year"] == 2019)

easterall <- easter2015 + easter2016 + easter2017 + easter2018 + easter2019

bhm2015 <- ifelse(hospts[,"day"] %in% c(4,25) & hospts[,"month"] == 5, 1, 0  &
hospts[,"year"] == 2015)
bha2015 <- ifelse(hospts[,"day"] %in% c(31) & hospts[,"month"] == 8, 1, 0  &
hospts[,"year"] == 2015)
bh2015 <- bhm2015 + bha2015
bhj2016 <- ifelse(hospts[,"day"] %in% c(1) & hospts[,"month"] == 1, 1, 0  &
hospts[,"year"] == 2016)
```

For peer review only

```
bhm2016 <- ifelse(hospts[,"day"] %in% c(2,30) & hospts[,"month"] == 5, 1, 0  &
hospts[,"year"] == 2016)
bha2016 <- ifelse(hospts[,"day"] %in% c(29) & hospts[,"month"] == 8, 1, 0  &
hospts[,"year"] == 2016)
bh2016 <- bhj2016 + bhm2016 + bha2016
bhj2017 <- ifelse(hospts[,"day"] %in% c(2) & hospts[,"month"] == 1, 1, 0  &
hospts[,"year"] == 2017)
bhm2017 <- ifelse(hospts[,"day"] %in% c(1,29) & hospts[,"month"] == 5, 1, 0  &
hospts[,"year"] == 2017)
bha2017 <- ifelse(hospts[,"day"] %in% c(28) & hospts[,"month"] == 8, 1, 0  &
hospts[,"year"] == 2017)
bh2017 <- bhj2017 + bhm2017 + bha2017
bhj2018 <- ifelse(hospts[,"day"] %in% c(1) & hospts[,"month"] == 1, 1, 0  &
hospts[,"year"] == 2018)
bhm2018 <- ifelse(hospts[,"day"] %in% c(7,28) & hospts[,"month"] == 5, 1, 0  &
hospts[,"year"] == 2018)
bha2018 <- ifelse(hospts[,"day"] %in% c(27) & hospts[,"month"] == 8, 1, 0  &
hospts[,"year"] == 2018)
bh2018 <- bhj2018 + bhm2018 + bha2018
bhj2019 <- ifelse(hospts[,"day"] %in% c(1) & hospts[,"month"] == 1, 1, 0  &
hospts[,"year"] == 2019)
bhm2019 <- ifelse(hospts[,"day"] %in% c(6,27) & hospts[,"month"] == 5, 1, 0  &
hospts[,"year"] == 2019)
bha2019 <- ifelse(hospts[,"day"] %in% c(26) & hospts[,"month"] == 8, 1, 0  &
hospts[,"year"] == 2019)
bh2019 <- bhj2019 + bhm2019 + bha2019

bhall <- bh2015 + bh2016 + bh2017 + bh2018 + bh2019

hollall <- easterall + bhall +chrisweek

#clean up the extra variables we don't need

rm(bhm2015,bha2015,bh2015,bhj2016,bhm2016,bha2016,bhj2017,bhm2017,bha2017,bhj2018,b
hm2018,bha2018,bhj2019,bhm2019,bha2019,chris)
rm(easter2015,easter2016,easter2017,easter2018,easter2018a,easter2018b,easter2019)


# Create a matrix of regressors for trend and daily dummies

trend_days <- tibble(trend, mon, tue, wed, thu, fri, sat, chrisweek, easterall,
bhall, tempts[,"last_year"], precipts[,"last_year"]) # create matrix of regressors:
trend + daily dummies
trend_days_noCLIM <- tibble(trend, mon, tue, wed, thu, fri, sat, chrisweek,
hollall) # create matrix of regressors: trend + daily dummies


#create the time series with the regressors included

seasonality <- msts(trend_days, seasonal.periods = c(7,365), ts.frequency = 7)
```

For peer review only

```
seasonality_noCLIM <-  msts(trend_days_noCLIM, seasonal.periods = c(7,365),
ts.frequency = 7)


# Estimate models with and without climatic data - other model types can be found
in the code graveyard

fit_trend <- auto.arima(hospts[,"id"], d=0, xreg=cbind(seasonality), lambda=0,
biasadj=TRUE)
fit_trend_noCLIM <- auto.arima(hospts[,"id"], d=0, xreg=cbind(seasonality_noCLIM),
lambda=0, biasadj=TRUE)

#Checks for the deterministic trend

summary(fit_trend) #to get model fit parameters manually
res_trend <- residuals(fit_trend)
checkresiduals(fit_trend, points = FALSE)
ggPacf(res_trend, lag=70)
adf.test(res_trend)
trend_nonseas = c(fit_trend$arma[1], fit_trend$arma[6], fit_trend$arma[2]) #pull
out the nonseasonal (first part) ARIMA parameters
trend_seas = c(fit_trend$arma[3], fit_trend$arma[7], fit_trend$arma[4]) #pull out
the seasonal (second part) ARIMA parameters

#Checks for the deterministic trend without climate data
summary(fit_trend_noCLIM) #to get model fit parameters manually
res_trend_noCLIM <- residuals(fit_trend_noCLIM)
checkresiduals(fit_trend_noCLIM, points = FALSE)
ggPacf(res_trend_noCLIM, lag=70)
adf.test(res_trend)
trend_noCLIM_nonseas = c(fit_trend_noCLIM$arma[1], fit_trend_noCLIM$arma[6],
fit_trend_noCLIM$arma[2]) #pull out the nonseasonal (first part) ARIMA parameters
trend_noCLIM_seas = c(fit_trend_noCLIM$arma[3], fit_trend_noCLIM$arma[7],
fit_trend_noCLIM$arma[4]) #pull out the seasonal (second part) ARIMA parameters


# Forecasting on training and test sets - 6 week horizon

train.hospts <- subset(hospts[,"id"], end = length(hospts[,"id"]) - 45)
test.hospts <- subset(hospts[,"id"], start=length(hospts[,"id"])-44)

lentrain=length(train.hospts)


#xlim=c(230,241) to examine the training/test split
train.hospts %>% autoplot()+
  autolayer(test.hospts, color = "red")


#deterministic trend
```

For peer review only

```
trend_model <- Arima(train.hospts, order=trend_nonseas, seasonal=trend_seas, 
include.constant = TRUE,
                     xreg = cbind(seasonality[1:lentrain,]),
                     lambda=0, biasadj=TRUE)

trend_fcast <- forecast(trend_model, level = c(95), xreg = 
cbind(seasonality[(lentrain+1):leng,]))

trend_fcast %>% autoplot() +
  autolayer(test.hospts, series="Actual value", color = 'darkorange') +
  guides(colour=guide_legend(title="")) +
  ggtitle("Deterministic trend model") +
  ylab("Daily admissions")


#deterministic trend no climate data

trend_model_noCLIM <- Arima(train.hospts, order=trend_noCLIM_nonseas, 
seasonal=trend_noCLIM_seas, include.constant = TRUE,
                     xreg = cbind(seasonality_noCLIM[1:lentrain,]),
                     lambda=0, biasadj=TRUE)

trend_fcast_noCLIM <- forecast(trend_model_noCLIM, level = c(95), xreg = 
cbind(seasonality_noCLIM[(lentrain+1):leng,]))

trend_fcast_noCLIM %>% autoplot() +
  autolayer(test.hospts, series="Actual value", color = 'darkorange') +
  guides(colour=guide_legend(title="")) +
  ggtitle("Deterministic trend model no climate") +
  ylab("Daily admissions")



# assess the accuracy - for AIC/BIC check model summaries - summary(trend_model) 
etc

accuracy(trend_model$fitted, hospts[,"id"])
accuracy(trend_model_noCLIM$fitted, hospts[,"id"])


#residual differences tables
diff_trend = hospts[(lentrain+1):leng,"id"] - trend_fcast$mean
date = as.character(paste(hospts[(lentrain+1):leng,"year"], 
hospts[(lentrain+1):leng,"month"],  hospts[(lentrain+1):leng,"day"], sep="/"))
dow = factor(as.character(hospts[(lentrain+1):leng,"dow"]), levels = 
c(0,1,2,3,4,5,6), labels = c("Sun", "Mon", "Tues", "Weds", "Thurs", "Fri", "Sat"))

#admissions values
```

For peer review only

```
acc_trend = abs(diff_trend) <= (0.2*mean(hosp_data$id))
acc_trend_s = abs(diff_trend) < (0.1*mean(hosp_data$id))


##put it into a nice format
diff_trend_df=data.frame("date" = date, "dow" = dow , "actual" =
hospts[(lentrain+1):leng,"id"], "predicted" = trend_fcast$mean, "difference"=
diff_trend, "lower95" = trend_fcast$lower, "upper95" = trend_fcast$upper,
"accuracybig" = acc_trend,  "accuracybetter" = acc_trend_s)

##write to a csv (easier to copy)- when you open it in Excel the date column will
look weird - just make sure to format it as a date in Excel and pick a format that
shows up properly - also you can reduce the df in excel
write.csv(diff_trend_df, file="Trend_diff.csv", row.names=FALSE)

##write all the data to a csv for making graphs
write.csv(hosp_data, file="all_data.csv", row.names=FALSE)
```

For peer review only

TRIPOD Checklist: Prediction Model Development

1Section/Topic	Item	Checklist Item	Page
**Title and abstract**			
Title	1	Identify the study as developing and/or validating a multivariable prediction model, the target population, and the outcome to be predicted.	1
Abstract	2	Provide a summary of objectives, study design, setting, participants, sample size, predictors, outcome, statistical analysis, results, and conclusions.	3
**Introduction**			
Background and objectives	3a	Explain the medical context (including whether diagnostic or prognostic) and rationale for developing or validating the multivariable prediction model, including references to existing models.	4,5
	3b	Specify the objectives, including whether the study describes the development or validation of the model or both.	5
**Methods**			
Source of data	4a	Describe the study design or source of data (e.g., randomized trial, cohort, or registry data), separately for the development and validation data sets, if applicable.	5
	4b	Specify the key study dates, including start of accrual; end of accrual; and, if applicable, end of follow-up.	5
Participants	5a	Specify key elements of the study setting (e.g., primary care, secondary care, general population) including number and location of centres.	5
	5b	Describe eligibility criteria for participants.	5
	5c	Give details of treatments received, if relevant.	N/A
Outcome	6a	Clearly define the outcome that is predicted by the prediction model, including how and when assessed.	5,6
	6b	Report any actions to blind assessment of the outcome to be predicted.	N/A
Predictors	7a	Clearly define all predictors used in developing or validating the multivariable prediction model, including how and when they were measured.	5,6
	7b	Report any actions to blind assessment of predictors for the outcome and other predictors.	N/A
Sample size	8	Explain how the study size was arrived at.	5
Missing data	9	Describe how missing data were handled (e.g., complete-case analysis, single imputation, multiple imputation) with details of any imputation method.	5
Statistical analysis methods	10a	Describe how predictors were handled in the analyses.	7
	10b	Specify type of model, all model-building procedures (including any predictor selection), and method for internal validation.	7
	10d	Specify all measures used to assess model performance and, if relevant, to compare multiple models.	7
Risk groups	11	Provide details on how risk groups were created, if done.	N/A
**Results**			
Participants	13a	Describe the flow of participants through the study, including the number of participants with and without the outcome and, if applicable, a summary of the follow-up time. A diagram may be helpful.	N/A
	13b	Describe the characteristics of the participants (basic demographics, clinical features, available predictors), including the number of participants with missing data for predictors and outcome.	8-9
Model development	14a	Specify the number of participants and outcome events in each analysis.	8-9
	14b	If done, report the unadjusted association between each candidate predictor and outcome.	N/A
Model specification	15a	Present the full prediction model to allow predictions for individuals (i.e., all regression coefficients, and model intercept or baseline survival at a given time point).	N/A
	15b	Explain how to the use the prediction model.	N/A
Model performance	16	Report performance measures (with CIs) for the prediction model.	8-9,
**Discussion**			
Limitations	18	Discuss any limitations of the study (such as nonrepresentative sample, few events per predictor, missing data).	13
Interpretation	19b	Give an overall interpretation of the results, considering objectives, limitations, and results from similar studies, and other relevant evidence.	12,13
Implications	20	Discuss the potential clinical use of the model and implications for future research.	13,14
**Other information**			
Supplementary information	21	Provide information about the availability of supplementary resources, such as study protocol, Web calculator, and data sets.	2,13
Funding	22	Give the source of funding and the role of the funders for the present study.	2

We recommend using the TRIPOD Checklist in conjunction with the TRIPOD Explanation and Elaboration document.

BMJ Open

Can we accurately forecast non-elective bed occupancy and admissions in the NHS? A time-series MSARIMA analysis of longitudinal data from an NHS Trust.

Journal:	*BMJ Open*
Manuscript ID	bmjopen-2021-056523.R1
Article Type:	Original research
Date Submitted by the Author:	24-Feb-2022
Complete List of Authors:	Eyles, Emily; NIHR ARC West; University of Bristol School of Medical Sciences, Population Health Sciences<br>Redaniel, Maria Theresa; NIHR ARC West; University of Bristol School of Medical Sciences, Population Health Sciences<br>Jones, Tim; NIHR ARC West, NIHR ARC West; University of Bristol School of Medical Sciences, Population Health Sciences<br>Prat, Marion; University of Bristol Faculty of Social Sciences and Law, School of Economics<br>Keen, Tim; North Bristol NHS Trust
**Primary Subject Heading**:	Epidemiology
Secondary Subject Heading:	Emergency medicine, Health services research
Keywords:	STATISTICS & RESEARCH METHODS, HEALTH SERVICES ADMINISTRATION & MANAGEMENT, EPIDEMIOLOGY, ACCIDENT & EMERGENCY MEDICINE

SCHOLARONE™
Manuscripts

I, the Submitting Author has the right to grant and does grant on behalf of all authors of the Work (as defined in the below author licence), an exclusive licence and/or a non-exclusive licence for contributions from authors who are: i) UK Crown employees; ii) where BMJ has agreed a CC-BY licence shall apply, and/or iii) in accordance with the terms applicable for US Federal Government officers or employees acting as part of their official duties; on a worldwide, perpetual, irrevocable, royalty-free basis to BMJ Publishing Group Ltd ("BMJ") its licensees and where the relevant Journal is co-owned by BMJ to the co-owners of the Journal, to publish the Work in this journal and any other BMJ products and to exploit all rights, as set out in our licence.

The Submitting Author accepts and understands that any supply made under these terms is made by BMJ to the Submitting Author unless you are acting as an employee on behalf of your employer or a postgraduate student of an affiliated institution which is paying any applicable article publishing charge ("APC") for Open Access articles. Where the Submitting Author wishes to make the Work available on an Open Access basis (and intends to pay the relevant APC), the terms of reuse of such Open Access shall be governed by a Creative Commons licence – details of these licences and which Creative Commons licence will apply to this Work are set out in our licence referred to above.

Other than as permitted in any relevant BMJ Author's Self Archiving Policies, I confirm this Work has not been accepted for publication elsewhere, is not being considered for publication elsewhere and does not duplicate material already published. I confirm all authors consent to publication of this Work and authorise the granting of this licence.

For peer review only

Can we accurately forecast non-elective bed occupancy and admissions in the NHS? A time-series MSARIMA analysis of longitudinal data from an NHS Trust.

Emily Eyles [1,2*] (emily.eyles@bristol.ac.uk) ; Maria Theresa Redaniel [1,2] (theresa.redaniel@bristol.ac.uk); Tim Jones [1,2] (timothy.jones@bristol.ac.uk); Marion Prat [3] (marion.prat@bristol.ac.uk); Tim Keen [4] (tim.keen@nbt.nhs.uk)

1. The National Institute for Health Research Applied Research Collaboration West (NIHR ARC West) at University Hospitals Bristol and Weston NHS Foundation Trust, UK
2. Population Health Sciences, Bristol Medical School, University of Bristol, UK
3. School of Economics, University of Bristol, UK
4. North Bristol NHS Trust, UK

*corresponding author: 9th Floor, Whitefriars, Lewins Mead, Bristol BS1 2NT. emily.eyles@bristol.ac.uk ; 0117 342 1262

For peer review only

Abstract

Objectives: The main objective of the study was to develop more accurate and precise short-term forecasting models for admissions and bed occupancy for an NHS Trust located in Bristol, England. Subforecasts for the medical and surgical specialties, and for different lengths of stay were realised

Design: Autoregressive integrated moving average models were specified on a training dataset of daily count data, then tested on a six-week forecast horizon. Explanatory variables were included in the models: day of the week, holiday days, lagged temperature and precipitation.

Setting: A secondary care hospital in an NHS Trust in South West England.

Participants: Hospital admissions between September 2016 and March 2020, comprising 1291 days.

Primary and secondary outcome measures: The accuracy of the forecasts was assessed through standard measures, as well as compared to the actual data using accuracy thresholds of 10% and 20% of the mean number of admissions or occupied beds.

Results: The overall ARIMA admissions forecast was compared to the Trust's forecast, and found to be more accurate, namely, being closer to the actual value 95.6% of the time. Furthermore, it was more precise than the Trust's. The subforecasts, as well as those for bed occupancy, tended to be less accurate compared to the overall forecasts. All of the explanatory variables improved the forecasts.

Conclusions: ARIMA models can forecast nonelective admissions in an NHS Trust accurately on a 6-week horizon, which is an improvement on the current predictive modelling in the Trust. These models can be readily applied to other contexts, improving patient flow.

Strengths and limitations of this study:

- The use of ARIMA models, which are simple to set up using *R*, and can be used to improve short term forecasts given enough input data are a strength of this study.Limitations include poorer accuracy in subforecasts, possibly due to using a simpler method.
- The coronavirus pandemic has an impact on the accuracy of forecasts, as shown by the sensitivity analysis
- Data from only one NHS Trust was used, so the models are not validated for other contexts.

Word Count: 4742

Introduction

Hospitals are increasingly busy, and there is more demand for resources such as hospital beds than can be easily met. Resource management is a challenge for many hospitals, and with an increase in the number of unplanned admissions, the issue of efficient resource allocation has become more urgent. The annual growth rate in England of emergency admissions is 3.2% on average over the last ten years [1]. Inaccurate estimates of unplanned admissions, and therefore, unpredictable emergency admissions, can lead to the cancellation of planned or routine operations[2]. This leads to subsequent need for more capacity to cope with routine operation backlog. [3 4]. Granular models with daily and

For peer review only

weekly forecasts predicted accurately can help strategically plan short term and long term resource management, particularly in order to cope with surges in demand.

In the past three and a half years, emergency admissions in the North Bristol NHS Trust (NBT) increased by 26% while average length of stay was reduced from 7.4 days to 6.1 days over the same period[5]. The North Bristol Trust is situated in Bristol and South Gloucestershire, England. The non-elective activity in NBT, particularly emergency admissions and bed occupancy, is higher than in other hospitals in the Bristol, North Somerset, South Gloucestershire Clinical Commissioning Group area. The consequences of this increase in emergency activity are a difficulty in meeting planned care, and, for the Trust, receiving less pay than expected, as unplanned procedures are paid less than planned ones[5]. As the increase in demand is rising faster than demographic growth, the need to efficiently forecast non-elective activity and understand its predictors has been identified. If the Trust is better able to forecast emergency admissions and understand the factors driving their increase, then it will be able to confidently plan over the longer term. Understanding demand allows the Trust to plan what capacity it will need to meet that demand, including the beds required, the workforce and the theatre time. This in turn influences what capacity is then available for elective work.

The NBT used two main methods to forecast non-elective admissions: a scenario-based model, combining the previous year's growth in demand and demographic growth, and a crude average growth model, which only includes demographic growth[5]. These techniques have been found to be insufficient for fine-grained, accurate operational planning, especially at the specialty level[5]. Several techniques have been used to predict unplanned admissions, including multiple linear regression[6-8], generalised estimating equations[9], exponential smoothing[8 10], and the widely-used family of Autoregressive Integrated Moving Average (ARIMA) models[8 11]. Other work has used hybrid models, including forecasting-simulation-optimisation SARIMA and ARIMA models have previously been used to forecast emergency department admissions and occupancy[2 8 10], emergency department crowding (hourly forecasting)[12], and infectious disease bed occupancy[13].

Previous work has mostly used horizons of up to one to two weeks, as forecasting benefit was seen to be limited for longer horizons [2]. Reliable hourly models have been established[12], but the daily horizon over a period of several weeks has been largely underexplored. Shah et al[14] developed a national model of daily midnight bed occupancy using 121 NHS Trusts, across a six week horizon, but this approach may not reflect local context, a priority in the NHS Long Term Plan[15].Our study will explore the six week horizon in a site-specific context. Further, most previous research did not divide by length of stay and specialty, but rather by urgency, or how severe a case is[10 16]. Medicine and Surgery may have different drivers in terms of admission to hospital, and longer lengths of stay tend to have different characteristics. Other work[17 18] has modelled not only beds but nurse and physician availability in A&E, inpatient and outpatient services.

In this paper, we aim to present a method of forecasting admissions and bed occupancy in a local context, using a 45 day (6 week) horizon. We also present subforecasts, divided by length of stay and specialty. The daily horizon over several weeks and the separation by length of stay and specialty are unique to this study, and we further use climatic information to inform the forecast as well. The subforecasts will be assessed using several measures of accuracy, including the mean absolute percentage error, and accuracy thresholds determined by the mean of that particular subforecast. This

For peer review only - http://bmjopen.bmj.com/site/about/guidelines.xhtml

will demonstrate the possibility of conducting more detailed, specific forecasts, which will allow for better, more granular planning.

Methods

NBT provided operational data on non-elective, i.e. unplanned, admissions to Southmead Hospital, the largest of the three hospitals in the Trust, from September 2016 to March 2020. These data are the precursor to what NBT provides to NHS Digital as part of the Hospital Episode Statistics (HES). On average, NBT reported having 868 General and Acute beds available[19]. Figure 1 shows the ratio of monthly elective to non-elective admissions up to February 2020, which was generated from HES data. The data from Hospital Episode Statistics (HES) was obtained under licence (DARS-NIC-17875-X7K1V) from NHS Digital (previously the Health and Social Care Information Centre); Copyright © 2021, re-used with the permission of The Health & Social Care Information Centre. All rights reserved. The data are provided by patients and collected by the NHS as part of their care and support. HES data can be accessed via NHS Digital[20].

The NBT data were split into two: a training dataset of 1246 days to develop the model, and a test dataset of 45 days to test the model. The six week forecast horizon was from the 29 January 2020 to the 14 March 2020. All days had at least one admission or occupied bed, so there were no missing values. The forecast horizon was decided by operational demand. Temperature data were obtained from the Horfield and Filton weather station, and precipitation data were obtained from the Met Office daily values for South West England[21].

Figure 1: Monthly ratio of elective to non-elective admissions, September 2016 to February 2020

Figure 1 goes here

We made forecasts for admissions and bed occupancy (see table 1). Admissions are the start of a hospital spell[22]. Admissions therefore are a count of the number of patients admitted to hospital on a particular day. Bed occupancy was calculated from the admissions data by counting a bed as being occupied by a patient between their admission and discharge dates. Bed occupancy is thus a cumulative measure of how many patients are in hospital on a particular day, even if they were admitted on a previous day. Zero length of stay is defined operationally as a stay of under 24 hours, which does not overlap midnight. Patients who stay over midnight, but for under 24 hours are counted as a non zero length of stay: e.g. if the patient was admitted at 9pm and was discharged at 3am the next day, this would be recorded as a length of stay of one rather than zero. Zero length of stay patients, i.e. patients who did not stay over at least one midnight, were excluded from the analysis[2]. This is because zero length of stay patients are managed through a separate bed-base at NBT. The acuity of these patients means they are rarely admitted into the main bed-base and may not occupy a physical bed (e.g., they may be under observation in a chair for a few hours).

For peer review only - http://bmjopen.bmj.com/site/about/guidelines.xhtml

We controlled for temperature and precipitation (both lagged, the same day last year) the day of the week, holidays (bank, Christmas week, and the Easter holiday weekend). The temperature and precipitation were lagged to the previous year, as weather prediction is not sufficiently accurate for longer forecast horizons. Weather data have been included in other forecasting models and contexts with, to mixed results[10 16 23 24].

Day of the week, as well as holidays, inclusive of bank holidays, were found across several studies to be highly relevant in predicting secondary care admissions[23-26]. Finally, a simple trend variable was generated, to account for the upward direction of admissions and bed occupancy over time, calculated as the days since the start of the dataset [27]. Table 1 details the variables included in the models. Separate subforecasts were also conducted divided by specialty (Surgery or Medicine) and length of stay (over or under 48 hours), detailed in table 2. The threshold of 48 hours was chosen as it is the acute medicine phase[28]. In the UK, as a part of good clinical care leading to successful outcomes, many patients can and should be diagnosed, treated, and even discharged within the 48 hour timeline[28]. Specialty was determined by a classification of Treatment Function Codes[29] provided by the Trust.

Table 1: Outcome variables and explanatory variables

**Variable**	**Definition**
**Outcomes**	
Admissions	Count of admissions to hospital, for a non-zero length of stay, i.e. overnight stays
Bed Occupancy	Count of hospital beds occupied, calculated as a count between admission and discharge dates, for non-zero lengths of stay
**Explanatory variables**	
Day of the week	Sunday, Monday, Tuesday, Wednesday, Thursday, Friday, Saturday
Temperature last year	The average temperature in Celsius on the same day last year
Precipitation last year	The precipitation in millimetres on the same day last year
Holiday	Whether a day was a bank holiday, over Christmas week, or during the Easter holiday weekend

For peer review only - http://bmjopen.bmj.com/site/about/guidelines.xhtml

Table 2: Model specifications

Outcome	Treatment Specialty	Length of Stay
**Admissions**	All	All
		<= 48 hours
		> 48 hours
	Medicine	All
		<= 48 hours
		> 48 hours
	Surgery	All
		<= 48 hours
		> 48 hours
**Bed Occupancy**	All	All
		<= 48 hours
		> 48 hours
	Medicine	All
		<= 48 hours
		> 48 hours
	Surgery	All
		<= 48 hours
		> 48 hours

For peer review only

We used Multivariate Seasonal AutoRegressive Integrated Moving Average (MSARIMA) models to forecast emergency admissions and bed occupancy, following the ARIMA forecasting procedure detailed by Jebb et al[27]. MSARIMA models allow for seasonality, or regularly repeating cycles or trends in the data, and takes into account the autoregressive order p, d is the differencing order, and q is the moving average order. The second set of brackets represents the seasonal versions of these components[9]. The autoregressive part of the model regresses the forecast variable against its own past values, to the order of the number of time lags (p)[30]. The moving average part of the model uses the past regression errors terms to the order of the time lag of the error (q)[30]. The differencing part of the model (d) refers to how many times lagged values have been subtracted from the data [30].

The modelling component consists first of an examination of the time series to check whether it is stationary, ie whether its mean, variance and autocorrelation do not vary over time, a necessary requirement if we want to use information about the past behaviour of the series to learn something about its future values. Unlike other time series modelling techniques, the ARIMA family of models allow for nonstationary data and the inclusion of explanatory variables[27]. Augmented Dickey-Fuller tests indicated that all data series were stationary or integrated of order 0 (d=0). MSARIMA models are a special type of regression for time series, which use information from previous days to inform them, while adjusting for other factors in the model, such as the weather, or day of the week[10]. MSARIMA modelling techniques were chosen as they are one of the recommended advanced forecasting techniques by NHS England[22], due to the complexity and seasonality of A&E settings. Further, according to NHS England[22], these techniques provided the most consistent estimate of daily A&E patient volumes; other methods such as artificial neural network models were said to provide less accurate forecasts of these volumes.

For peer review only - http://bmjopen.bmj.com/site/about/guidelines.xhtml

The mean absolute percentage error (MAPE) and the root mean square error (RMSE) are presented for each model [2 10-13 16 30]. MAPE measures model fit with closer to 0% indicating better fit[16]. For the RMSE, lower values indicate better model performance, though its value is dependent on the mean value of the variable, which are presented in table 3 along with the standard deviations[11]. The model was tested against the test dataset, providing the actual out-of-sample accuracy. The model accuracy was determined by whether the residual of the forecast vs the observed was over or under a given threshold. We used 2 thresholds, representing moderate and strict accuracy. This was defined as 20% of the sample mean for moderate accurate and 10% of the sample mean for strict accuracy. These thresholds match the ad-hoc thresholds determined in meetings with NBT, namely a 10/20 bed threshold for all admissions, which is matched by a 9.4/18.8 bed threshold as mean percentages. Accuracy is thus represented as a percentage of accurate daily forecasts within those thresholds (see table 3). A short-term 6 week (45 day) forecast horizon was chosen, as long term daily forecasts require different methods, as eventually the autoregressive component of the model will converge to the mean [30].

The data were prepared using Stata 15.1, and analysed using the *forecast* package in R. Following the data checks, the *auto.arima* function in R was used to determine the model specifications[31]. To determine the relevance of the explanatory variables, once the model specifications were determined, the models were specified with and without the explanatory variables. Models with the explanatory variable of interest were compared to null models and models containing the other explanatory variables on the basis of AIC, similar to how *auto.arima* determines model specifications[31]. Admissions and bed occupancy were modelled separately. The first two models were all admissions and all bed occupancy data. Then, subforecasts were modelled, split by specialty or length of stay, and finally specialty and length of stay (see table 2). Detailed results for the subforecasts are presented in Appendix 1. The code for the models is provided in Appendix 2.

Sensitivity analyses were performed for two other time periods on the overall admissions dataset. The first was using the data from September 2016 to mid December 2019, for a horizon of six weeks between the 1 November 2019 and the 15 December 2019. The second sensitivity analysis used the data from September 2016 to mid June 2020, for a horizon between the 2 May 2020 and the 15 June 2020. These horizons were selected to either omit the impact of the coronavirus pandemic (the earlier horizon) or to examine the forecast within the coronavirus pandemic (the later horizon). A further sensitivity analysis was performed by excluding the climatic data from the models (see Appendix 3).

Patient and public involvement statement: Members of the public were consulted in the development of this study. The research idea was presented to them in a workshop and suggestions and comments were incorporated in the protocol. Feedback during the workshop was positive, with participants agreeing with the research objectives and the identified need for forecasting models. The use of generic and generalisable models were highlighted in the discussion, and that the audience and end users perspectives should be considered in the development of the model and tool.

Results

18 separate ARIMA models were forecast for the variable specifications described in Table 1, and the subsets described in table 2. The final ARIMA specification, accuracy, RMSE, and MAPE of the models can be seen in table 3. Day of the week, holiday days, temperature, and precipitation were all found to improve the accuracy of the forecasts and subforecasts. The model AICs and the AICs of

For peer review only - http://bmjopen.bmj.com/site/about/guidelines.xhtml

comparable models without temperature and precipitation can be found in Appendix 3. The subsets' mean and standard deviation are also provided. Models with more training data, i.e. higher sample size, were more accurate.

The overall admissions model was accurate at the moderate threshold 88.9% of the time, and at the strict threshold 60.0% of the time. Bar the surgical and under 48 hour length of stay subforecasts, 82.2% of daily predictions of admissions were within the moderate admission threshold; 51.1% were within the strict accuracy admission threshold. Models for surgical admissions performed less well, with the under 48 hour length of stay surgical admissions having accuracy of 31.1% on the moderate level, and 11.1% on the strict level. This is likely due to sample size, and high variation in daily admissions.

For bed occupancy, the models generally performed more poorly. For the moderate bed threshold, all models were at least 28.9% accurate, and for the strict bed threshold, the lowest accuracy was 15.6%. However, this, similar to admissions, was dependent on specialty and length of stay. The bed occupancy model overall (model 10) performed rather poorly, and was accurate within the moderate threshold77.8% of the time, and within the strict threshold 40.0 % of the time. Surgery performed better as a specialty, and under 48 hour length of stay tended to outperform over 48 hour length of stay as well. Appendix 1 shows the subforecasts for each model in table 3, with 95% confidence intervals.

Table 3: Model specifications and accuracy

Model	Outcome	(Sub)for-ecast	SARIMA (p,d,q)(P,D,Q)	Model accuracy (strict/moderate thresholds)	RMSE	MAPE	Mean of Outcome	SD of outcome
1	Admissions	All data overall	(3,0,0)(1,0,0)	60.0%/88.9%	9.59	8.66	94.04	14.98
2	Admissions	All medical	(3,0,2)(,2,0,2)	51.1%/84.4%	8.98	9.44	79.14	19.32
3	Admissions	All surgical	(1,0,2)(0,0,0)	28.9%/57.8%	4.59	19.86	20.65	5.31
4	Admissions	All under 48 hour length of stay	(1,0,0)(1,0,0)	28.9%/71.1%	6.81	16.53	35.38	9.06
5	Admissions	All over 48 hour length of stay	(3,0,1)(1,0,1)	62.2%/88.9%	7.22	10.78	55.14	10.04
6	Admissions	Under 48 hour length of stay, medical	(4,0,0)(2,0,0)	24.4%/48.9%	6.45	18.64	30.59	10.21
7	Admissions	Under 48 hour	(0,01)(0,0,0)	11.1%/31.1%	7.82	44.19	20.99	8.39

For peer review only - http://bmjopen.bmj.com/site/about/guidelines.xhtml

		length of stay, surgical						
8	Admissions	Over 48 hour length of stay, medical	(5,0,1) (2,0,0)	53.3%/82.2 %	6.88	11.97	48.55	12.06
9	Admissions	Over 48 hour length of stay, surgical	(3,0,3) (1,0,2)	25.8%/46.7 %	3.40	27.45	11.87	3.74
10	Bed Occupancy	All data overall	(0,0,1) (1,0,0)	40.0%/77.8 %	158.24	15.57	823.36	165.22
11	Bed Occupancy	All medical	(4,0,0) (2,0,0)	37.8%/60.0 %	151.69	17.06	760.03	189.22
12	Bed Occupancy	All surgical	(0,0,1) (0,0,0)	24.4%/37.8 %	64.50	35.40	162.62	67.71
13	Bed Occupancy	All under 48 hour length of stay	(3,0,0) (2,0,1)	28.9%/66.7 %	15.72	16.41	82.3	21.06
14	Bed Occupancy	All over 48 hour length of stay	(0,0,1) (1,00)	40.0%/75.6 %	159.55	17.6	767.41	177.2
15	Bed Occupancy	Under 48 hour length of stay, medical	(4,0,0) (2,0,0)	22.2%/48.9 %	14.95	18.63	70.83	23.95
16	Bed Occupancy	Under 48 hour length of stay, surgical	(0,0,1) (0,0,0)	15.6%/28.9 %	7.82	44.19	21	8.38
17	Bed Occupancy	Over 48 hour length of stay, medical	(2,0,1) (0,0,0)	35.6%/57.8 %	152.7	19.15	673.24	181.24
18	Bed Occupancy	Over 48 hour length	(0,0,1) (0,0,0)	20.0%/35.6 %	64.95	44.22	141.63	67.49

For peer review only - http://bmjopen.bmj.com/site/about/guidelines.xhtml

		of stay, surgical						

Our forecasts were fairly close to the actual values observed in the test data. Our prediction for admissions overall, for example, which can be seen in table 4, was within the moderate threshold (18.8), excepting 5 days on our 45-day horizon. 15 days were between the moderate and strict (9.4) thresholds. 25 days were under the strict accuracy threshold. The model was less accurate the farther forward in time from the beginning of the horizon. While the forecast sometimes underestimates the number of admissions, the forecasting models are reviewed by the clinical site team, and make adjustments to the predictor based on the particular day. Further, possibly due to the impact of the coronavirus pandemic, the latter part of the horizon may have different patterning with respect to hospital admissions. This can be seen in figure 2 (Model 1 predictions), with the actual value of admissions dropping at the end of the horizon. The 95% confidence intervals range from about 67 to 135 beds wide.

Figure 2: ARIMA forecast for overall admissions to NBT

Figure 2 goes here

Compared to NBT's in-house prediction modelling for overall admissions, the ARIMA model outperformed them in terms of accuracy apart from two days, one which the NBT model was closer by just 1.5 beds, and another where the NBT model was closer by 10 beds. The ARIMA forecast is closer in value to the true admissions count 95.6% of the time. Figure 3 shows the forecast for overall admissions and its 95% confidence interval, compared to the actual value and NBT model. The mean difference between the ARIMA models and the actual admissions value was 7.3 beds, with a standard deviation of 9.9 beds. The median difference for the ARIMA models was 6 beds. For the NBT prediction models, the mean difference from the actual value was 21 beds, with a standard deviation of 11.1 beds, and a median difference of 21 beds. Finally, the correlation between the ARIMA prediction and the actual values was 0.75, whereas for the NBT prediction, it was -0.45. Therefore, the ARIMA models are more accurate and more precise than the NBT models.

Figure 3: Forecast predictions for overall admissions (model 1) compared to the actual values and the NBT model

Figure 3 goes here

The sensitivity analyses for the other horizons (see Appendix 4) on the overall admissions data demonstrated that the modelling strategy was more accurate prior to the coronavirus pandemic. Compared to the horizon presented earlier, the December 2019 horizon predicted within a strict accuracy threshold (10% of the sample mean) 75.6% of the time compared to 60.0% for the study horizon, and within the moderate accuracy threshold (20% of the sample mean) 93.3% of the time, compared to 88.9% in the study horizon. The June 2020 forecast horizon performed worse than both study horizon and other sensitivity analysis. It only forecasted 42.2% of the time within the strict threshold, and 77.8% of the time within the moderate threshold. The coronavirus pandemic likely has an impact on forecasting models, so consideration should be taken when using data from the pandemic period.

Discussion

For peer review only - http://bmjopen.bmj.com/site/about/guidelines.xhtml

We have extended the literature by producing forecasts for a variety of combinations of specialty and length of stay, for both emergency admissions and bed occupancy, as well as for nonelective admissions beyond those for trauma[23 25 32]. We have found that these models work very well for some but not as well for other types of subsets, specifically shorter lengths of stay and for surgery. The models appear to work better in general for admissions rather than bed occupancy, as well as for shorter lengths of stay. The differences in accuracy can possibly be explained by the beginning of the coronavirus pandemic in the United Kingdom, as can be seen in the test data for several of the subsets, particularly for over 48 hour length of stay, and bed occupancy: a sharp drop can be seen at the beginning of February 2020. In the all data categories, and under 48 hour length of stay this sharp rise and fall can be seen around mid February 2020 (see appendix 1). This can be interpreted as a limitation to the models' usefulness while COVID-19 has an impact on hospital admissions. Indeed, the sensitivity analyses showed that the pandemic had an impact on the accuracy of the models. Back to recovery, similarly to the pre-pandemic sensitivity analysis, the model might be more applicable although it might need more refinements to take into account changes brought about by the pandemic.

Other limitations of this study include the poorer accuracy in subforecasts, which may require more complex methods to mitigate, and the use of only one NHS Trust context. Using only one Trust means that the models are not necessarily validated for different contexts and settings. Work by Shah et al[14] has done similar modelling techniques at the national level, with accurate results. However, models should be site-specific to maximise utility: this means that it is necessary to test them in other Trusts. Further, Ordu et al[8] emphasise the importance of testing different modelling techniques for varying specialties and services. patients. More complex modelling strategies could prove to be more accurate, like the combination of different modelling techniques, such as hybrid forecasting-simulation-optimisation models[17 18], which can be used for not only A&E but inpatient and outpatient services as well.

The strengths of this study are the use of ARIMA models, which are simple to set up using *R*, and can be used to improve short term forecasts given enough input data. The dataset used is the hospital level precursor to the Hospital Episode Statistics (HES), available through *NHS Digital* It is possible to improve short-term models from NBT with a relatively simple procedure, which should be transferable to other contexts, due to this similarity. The models can be incorporated easily into practice by sharing the *R* code on Github or a similar platform, and tested on data from other Trusts, which should follow a standard structure, again due to the reporting requirements of HES The code used to generate the models in this paper is available, with commentary, as a supplementary file (see appendix 2).

Abraham and colleagues found that admissions for patients spending at least one night were predominantly random and difficult to predict, using 3 years worth of training data. However, we found that, in general, over all subsets admissions forecasts specified better than bed occupancy ones, and that forecast horizons of over a week can be produced. It would appear that bed occupancy data are more volatile than admissions. Bed occupancy may be harder to forecast, in that it is driven by admissions, and then length of stay is at times difficult to predict[33]. It is likely that, for subsets of bed occupancy, due to the smaller amount of observations (for example, under 48 hour surgical bed occupancy has a mean of 21.00 compared to 849.71 on the whole), and higher variability of the subsets, that the models for bed occupancy produce less accurate forecasts with higher MAPEs. The MAPE for daily admissions found by Boyle and colleagues is 11%: several of the subforecasts, such as the medicine, surgery, and over 48 hour length of stay ones reach this threshold, but as the amount of

For peer review only - http://bmjopen.bmj.com/site/about/guidelines.xhtml

observations decreases, the inaccuracy seems to increase. A possible avenue of improvement would be to forecast bed occupancy by levels of severity.

Day of the week has been shown to have reasonably strong predictive capacity in previous work[23 24 26], though the inclusion of climate variables is debated, with no clear answer[9 10 16 24-26 32 34]. Calegari and colleagues found that SARIMA models including day of the week, but not climatic variables were the most accurate, especially in complex departments. However, the test period did not comprise the whole year, and the study was located in one hospital in southern Brazil, a different climate to Bristol, England[10]. Similarly, in Brazil, the ambient temperature effect was not found to improve daily visit forecasts, though again, the data only were for one season. Sun et al[16] also found that admissions were not predicted by weather, though caution that Singapore, the study setting, has little variation in its weather throughout the year. Atherton et al[25] found that adult trauma admissions were not influenced by the weather, but only used temperature in 5 Celsius increments over a single year, whereas this study uses temperature in Celsius to a single decimal point over a longer time period. In contrast, Macgregor[34] found an association between daily temperature and trauma admissions in Aberdeen, and Rising et al[32] found similar results for temperature and precipitation on trauma admissions in an American hospital. Note that these solely examine trauma admissions, rather than other types of nonelective secondary admissions, which our study includes. Finally, Sahu et al[26] developed a Bayesian model for forecasting hospital admissions in Cardiff and Southampton, two nearby localities to Bristol, the setting of this study, and emphasised the importance and relevance of including weather data. Therefore context is important, as in climatic data marginally improved our models, similar to other UK studies, and we suggest its inclusion be considered in future work.

By improving short term forecasts, care can be better planned, limiting cancellations due to capacity or patient flow issues. Better care can be provided as hospitals will be less crowded. In future, other methods more appropriate for longer horizons can be tested, and medium to long term forecasts can be undertaken with more appropriate methods. We have developed ARIMA models that can forecast emergency admissions over a six week horizon with very good accuracy, which are more precise and more accurate than the current models used by the NBT. More work is needed to inform further model development, as the accuracy of subforecasts for length of stay and specialty varies significantly. This is important work because improved modelling capability will have a direct impact on the business planning of acute NHS Trusts. Improving patient flow through the hospitals greatly improves the working and care environment, reduces A&E crowding and enables hospitals to provide the most appropriate care for patients.

Acknowledgements

This research was supported by the National Institute for Health Research (NIHR) Applied Research Collaboration West (NIHR ARC West). The views expressed in this article are those of the author(s) and not necessarily those of the NIHR or the Department of Health and Social Care. The authors are grateful to the members of the public who contributed through the patient and public involvement process.

Author Statement: EE drafted the manuscript. EE and TJ analysed the data. MTR and TK supervised the project. TJ managed the data. MP conceptualised the methodology and conducted preliminary analysis of the data. MTR is the guarantor of this paper. The public contributed through the patient and public involvement process in the development of the study.

For peer review only - http://bmjopen.bmj.com/site/about/guidelines.xhtml

Copyright: I, the Submitting Author has the right to grant and does grant on behalf of all authors of the Work (as defined in the author licence), an exclusive licence and/or a non-exclusive licence for contributions from authors who are: i) UK Crown employees; ii) where BMJ has agreed a CC-BY licence shall apply, and/or iii) in accordance with the terms applicable for US Federal Government officers or employees acting as part of their official duties; on a worldwide, perpetual, irrevocable, royalty-free basis to BMJ Publishing Group Ltd ("BMJ") its licensees.

The Submitting Author accepts and understands that any supply made under these terms is made by BMJ to the Submitting Author unless you are acting as an employee on behalf of your employer or a postgraduate student of an affiliated institution which is paying any applicable article publishing charge ("APC") for Open Access articles. Where the Submitting Author wishes to make the Work available on an Open Access basis (and intends to pay the relevant APC), the terms of reuse of such Open Access shall be governed by a Creative Commons licence – details of these licences and which licence will apply to this Work are set out in our licence referred to above.

Competing interest statement: All authors have completed the ICMJE uniform disclosure form at www.icmje.org/coi_disclosure.pdf and declare: support from HDRUK Better Care Partnership and NIHR ARC West for the submitted work; no financial relationships with any organisations that might have an interest in the submitted work in the previous three years; no other relationships or activities that could appear to have influenced the submitted work.

Transparency declaration: The lead author affirms that this manuscript is an honest, accurate, and transparent account of the study being reported; that no important aspects of the study have been omitted; and that any discrepancies from the study as planned (and, if relevant, registered) have been explained[35].

Ethical approval: University of Bristol Faculty Research Ethics Approval was obtained. (ID #109,024)

Funding statement: This study was funded by the HDRUK Better Care Partnership (#6.12). This research was supported by the National Institute for Health Research (NIHR) Applied Research Collaboration West (NIHR ARC West). The views expressed in this article are those of the author(s) and not necessarily those of the NIHR or the Department of Health and Social Care.

Role of sponsor/ Statement of independence of researchers from funders: The funding source and sponsors had no role in the design of this study and will not have any role during its execution, analyses, interpretation of the data, or decision to submit results.

Dissemination declaration: The results of the study are to be shared to the North Bristol NHS Trust.

Data sharing statement: The data used in the study is collected by the NBT as part of their care and support. Sharing of anonymised data with the University of Bristol was underpinned by a data sharing agreement and solely covers the purposes of this study. Data requests can be made through the NBT. Statistical code is available as a supplementary file.

References

1. Steventon A, Deeny S, Friebel R, et al. Briefing: Emergency hospital admissions in England: which may be avoidable and how? London: The Health Foundation, 2018.
2. Abraham G, Byrnes GB, Bain CA. Short-Term Forecasting of Emergency Inpatient Flow. *IEEE Transactions on Information Technology in Biomedicine* 2009;13(3):380-88.

For peer review only - http://bmjopen.bmj.com/site/about/guidelines.xhtml

3. Association BM. NHS pressures – winter analysis. London: British Medical Association, 2018.
4. Office NA. Report by the Comptroller and Auditor General: Reducing emergency admissions. London: National Audit Office, 2018.
5. Falconer C, Keen T. Forecasting and demand in the North Bristol Trust, 2018.
6. Bilings J, Georghiou T, Blunt I, et al. Choosing a model to predict hospital admission: an observational study of new variants of predictive models for case finding. *BMJ Open* 2013;3:e003352.
7. Boyle J, Wallis M, Jessup M, et al. Regression Forecasting of Patient Admission Data. *30th Annual International Conference of the IEEE Engineering in Medicine and Biology Society* 2008:3819-22.
8. Ordu M, Demir E, Tofallis C. A comprehensive modelling framework to forecast the demand for all hospital services. *The International Journal of Health Planning and Management* 2019;34(2):e1257-e71.
9. Marcilio I, Hajat S, Gouveia N. Forecasting Daily Emergency Department Visits Using Calendar Variables and Ambient Temperature Readings. *Academic Emergency Medicine* 2013;20(8):769-77.
10. Calegari R, Fogliatto FS, Lucini FR, et al. Forecasting Daily Volume and Acuity of Patients in the Emergency Department. *Computational and Mathematical Methods in Medicine* 2016;2016:1-8.
11. Wargon M, Guidet B, Hoang TD, et al. A systematic review of models for forecasting the number of emergency department visits. *Emerg Med J* 2009;26:395-99.
12. Schweigler LM, Desmond JS, McCarthy ML, et al. Forecasting Models of Emergency Department Crowding. *Academic Emergency Medicine* 2009;16(4):301-08.
13. Earnest A, Chen MI, Ng D, et al. Using autoregressive integrated moving average (ARIMA) models to predict and monitor the number of beds occupied during a SARS outbreak in a tertiary hospital in Singapore. *BMC Health Services Research* 2005;5:36.
14. Shah K, Sharma A, Moulton C, et al. Forecasting the Requirement for Nonelective Hospital Beds in the National Health Service of the United Kingdom: Model Development Study. *JMIR Med Inform* 2021;9:e21990.
15. England N. The NHS Long Term Plan. London: NHS, 2019.
16. Sun Y, Heng BH, Seow YT, et al. Forecasting daily attendances at an emergency department to aid resource planning. *BMC Emergency Medicine* 2009;9:1.
17. Ordu M, Demir E, Davari S. A hybrid analytical model for an entire hospital resource optimisation. *Soft Computing* 2021;25:11673-90.
18. Ordu M, Demir E, Tofallis C, et al. A novel healthcare resource allocation decision support tool: A forecasting-simulation-optimization approach. *Journal of the Operational Research Society* 2021;72(3):484-500.
19. Digital N. Bed availability and occupancy data - overnight London: NHS; 2021 [Available from: https://www.england.nhs.uk/statistics/statistical-work-areas/bed-availability-and-occupancy/bed-data-overnight/ accessed 28/01 2022.
20. Digital N. Data Access Request Service London: NHS; 2022 [Available from: https://digital.nhs.uk/services/data-access-request-service-dars
21. Office M. Daily Southwest England & Wales Precipitation (mm) In: Office M, ed. London, 2020.
22. England N, Improvement N. Advanced forecasting techniques: How to use advanced forecasting techniques for estimating demand of NHS Services. London: NHS, 2020.
23. Bhattacharyya T, Millham F. Relationship between Weather and Seasonal Factors and Trauma Admission Volume at a Level I Trauma Center. *The Journal of Trauma and Acute Care Surgery* 2001;51(1):118-22.
24. Holleman Jr DR, Bowling RL, Gathy C. Predicting daily visits to a walk-in clinic and emergency department using calendar and weather data. *J Gen Intern Med* 1996;11(4):237-39.

For peer review only - http://bmjopen.bmj.com/site/about/guidelines.xhtml

25. Atherton W, Harper W, Abrams K. A year's trauma admissions and the effect of the weather. *Injury* 2005;36(1):40-46.
26. Sahu S, Baffour B, Harper P, et al. A hierarchical Bayesian model for improving short-term forecasting of hospital demand by including meteorological information. *Journal of the Royal Statistical Society Series A (Statistics in Society)* 2014;177(1):39-61.
27. Jebb A, Tay L, Wang W, et al. Time series analysis for psychological research: Examining and forecasting change. *Frontiers in Psychology* 2015;6:727.
28. Leman P. Emergency care in the first 48 hours. *BMJ* 2007;334(7587):218-19.
29. NHS. Treatment Function Code 2021 [updated 25/11/2021. Available from: https://datadictionary.nhs.uk/attributes/treatment_function_code.html accessed 15/01 2022.
30. Hyndman RJ, Athanasopoulos G. Forecasting: principles and practice. Melbourne: OTexts, 2018.
31. Hyndman RJ. auto.arima 2020 [Available from: https://www.rdocumentation.org/packages/forecast/versions/8.12/topics/auto.arima accessed 20/08/2020 2020.
32. Rising W, O'Daniel J, Roberts C. Correlating Weather and Trauma Admissions at a Level I Trauma Center. *The Journal of Trauma and Acute Care Surgery* 2006;60(5):1096-100.
33. Predicting Hospital Length of Stay (PHLOS): A Multi-tiered Data Mining Approach. 2012 IEEE 12th International Conference on Data Mining Workshops; 2012 10-10 Dec. 2012.
34. Macgregor D. Effect of weather on attendance with injury at a paediatric emergency department. *Emerg Med J* 2003;20:204-05.
35. Altman DG, Moher D. Declaration of transparency for each research article. *BMJ : British Medical Journal* 2013;347

For peer review only

For peer review only - http://bmjopen.bmj.com/site/about/guidelines.xhtml

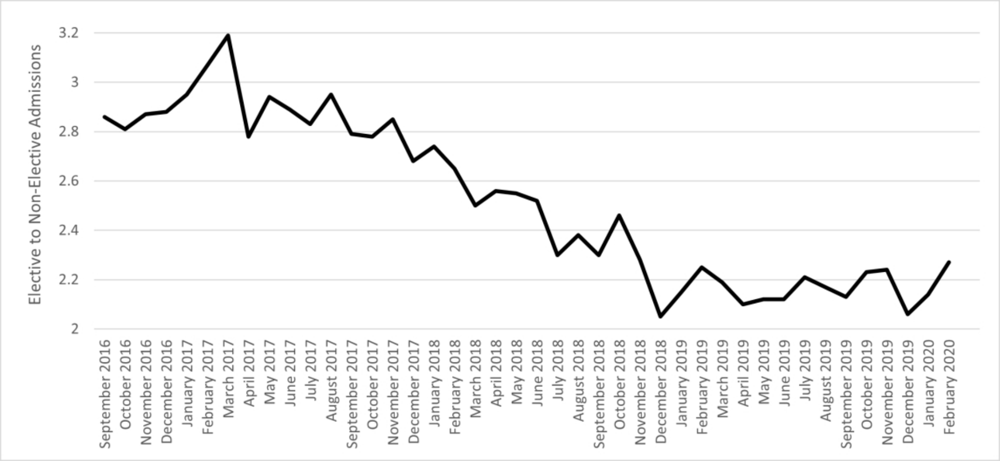

Figure 1: Monthly ratio of elective to non-elective admissions, September 2016 to February 2020

497x228mm (130 x 130 DPI)

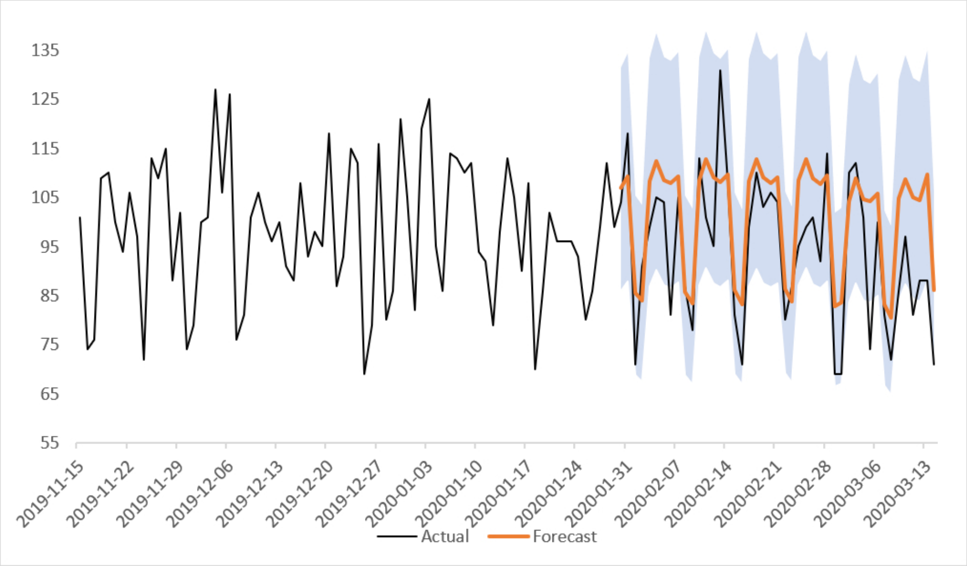

Figure 2: ARIMA forecast for overall admissions to NBT

566x330mm (47 x 47 DPI)

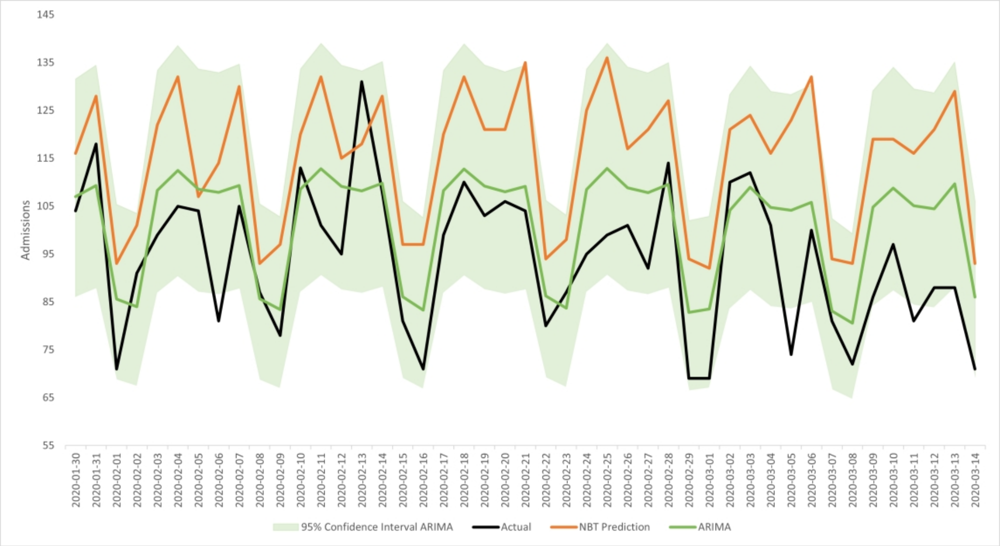

Figure 3: Forecast predictions for overall admissions (model 1) compared to the actual values and the NBT model

685x375mm (130 x 130 DPI)

Appendix 1: Subforecast figures

Figure A1: Model 2 (medicine admissions)

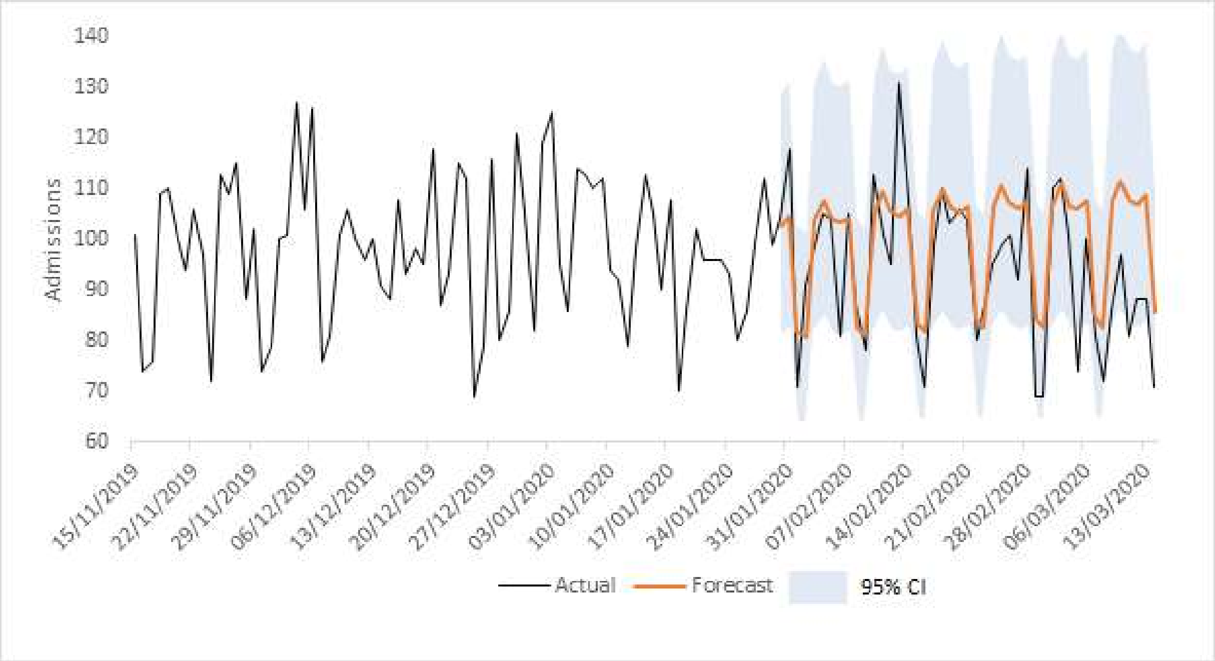

Figure A2: Model 3 (surgery admissions)

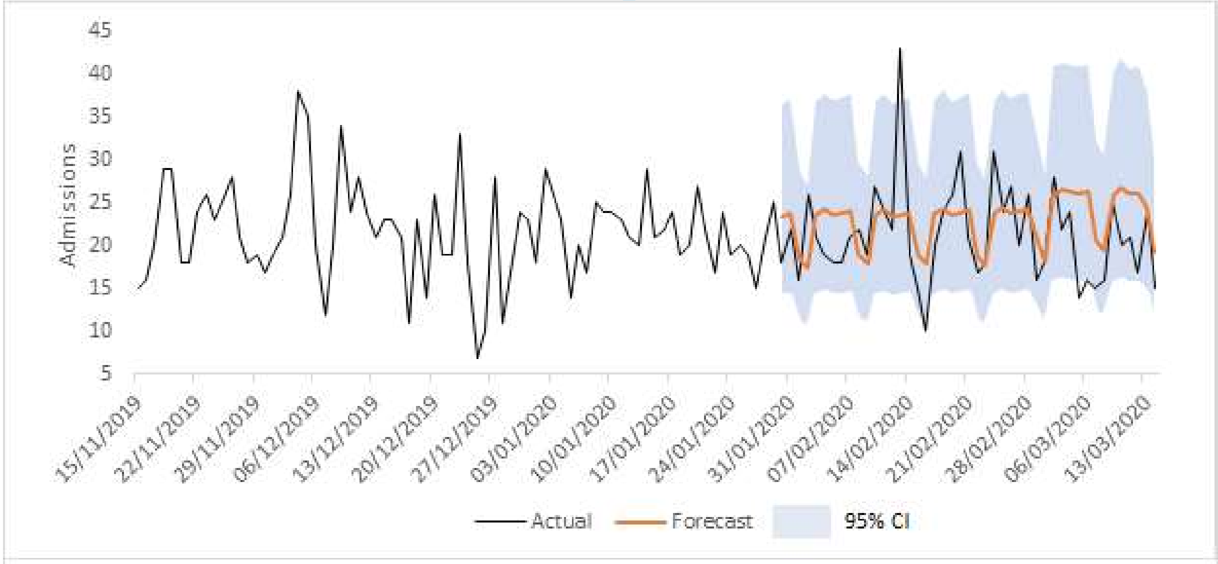

Figure A3: Model 4 (under 48 hour length of stay admissions)

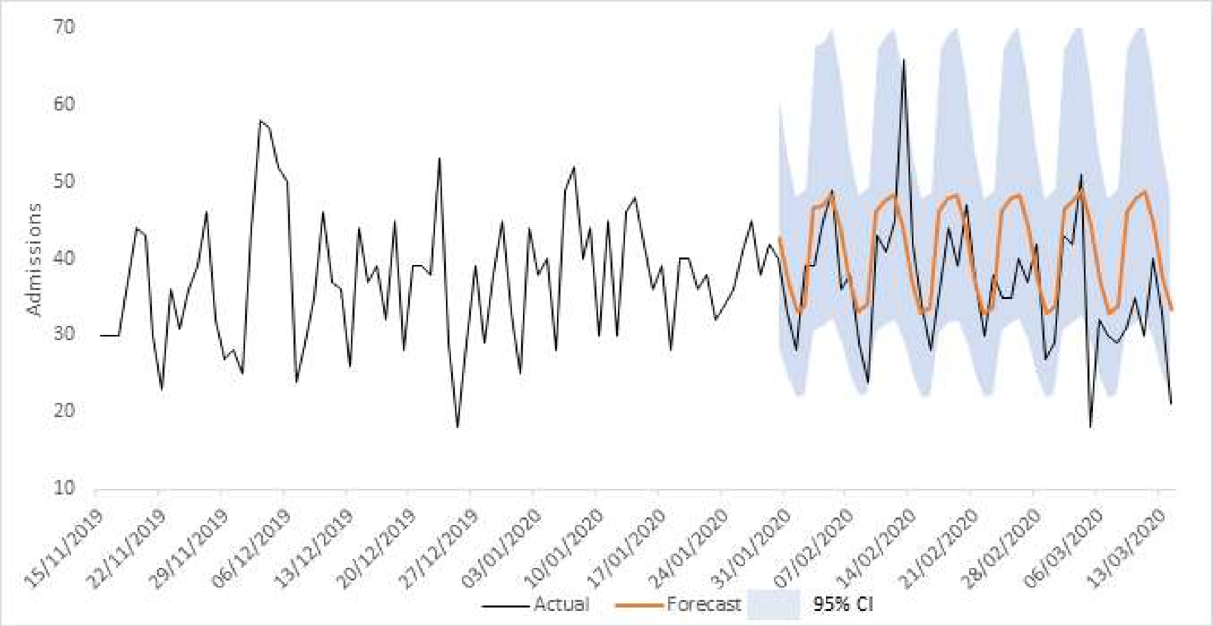

Figure A4: Model 5 (over 48 hour length of stay admissions)

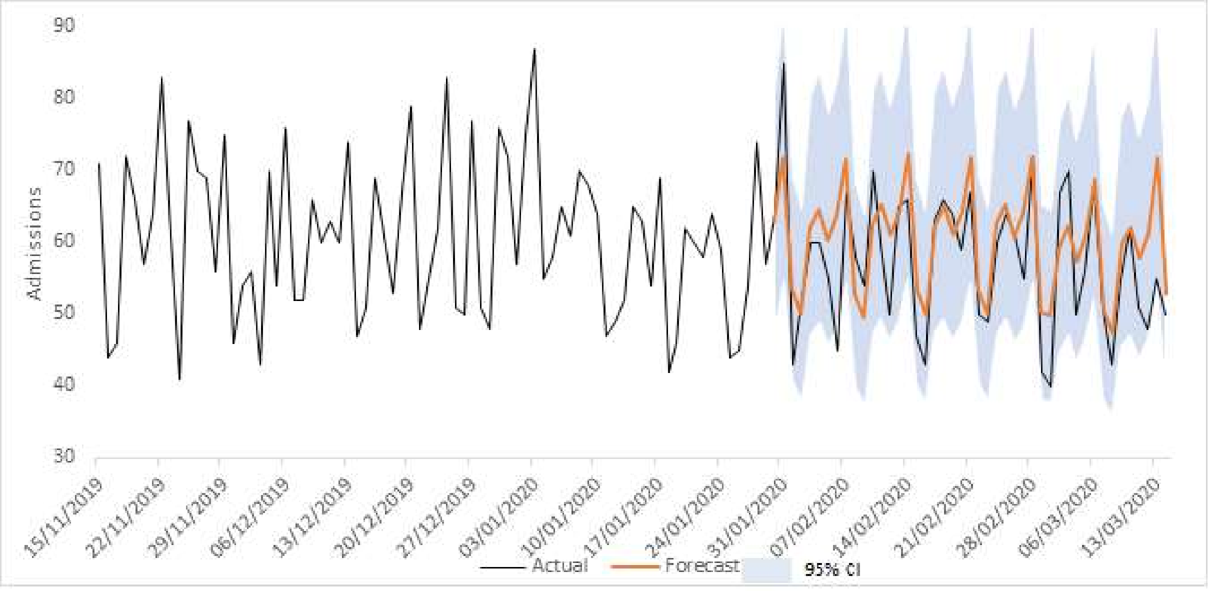

Figure A5: Model 6 (under 48 hour length of stay medicine admissions)

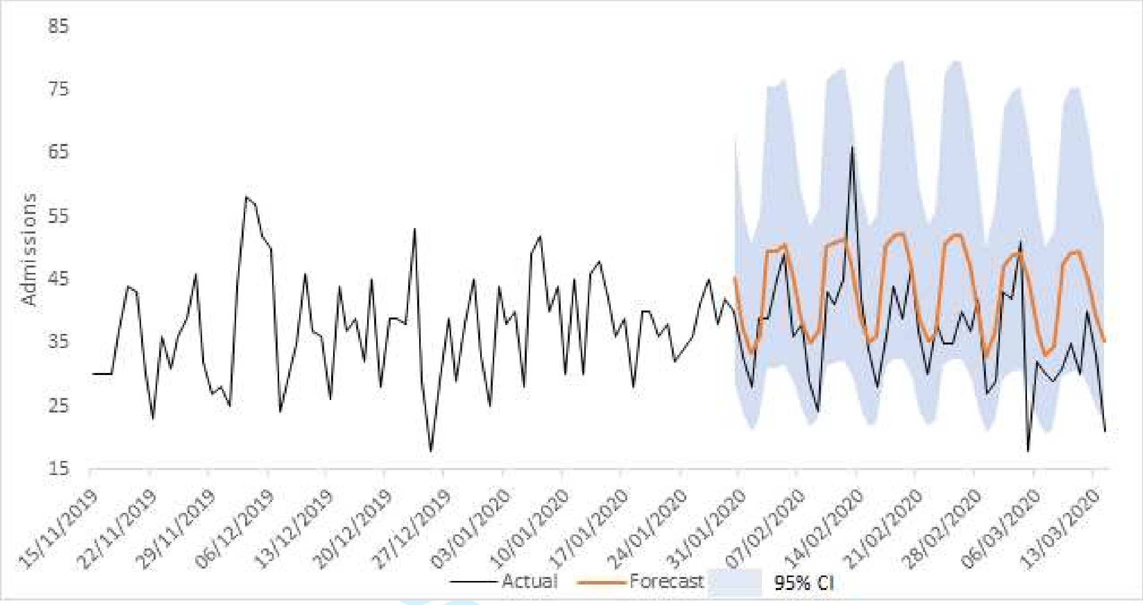

Figure A6: Model 7 (under 48 hour length of stay surgery admissions)

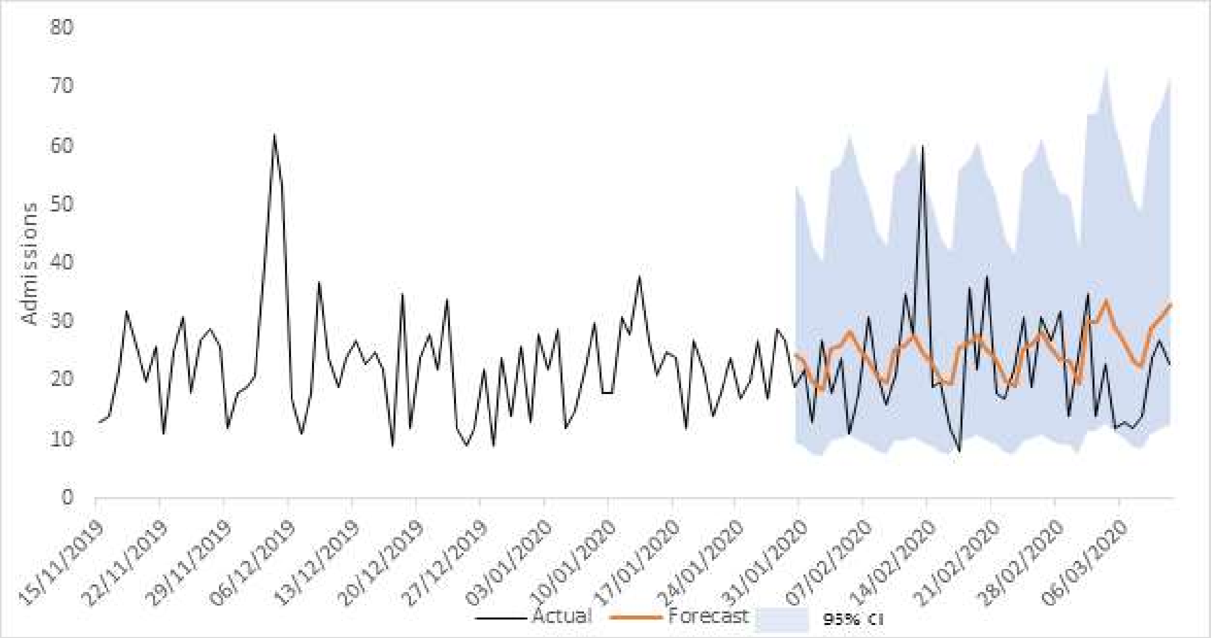

Figure A7: Model 8 (over 48 hour length of stay medicine admissions)

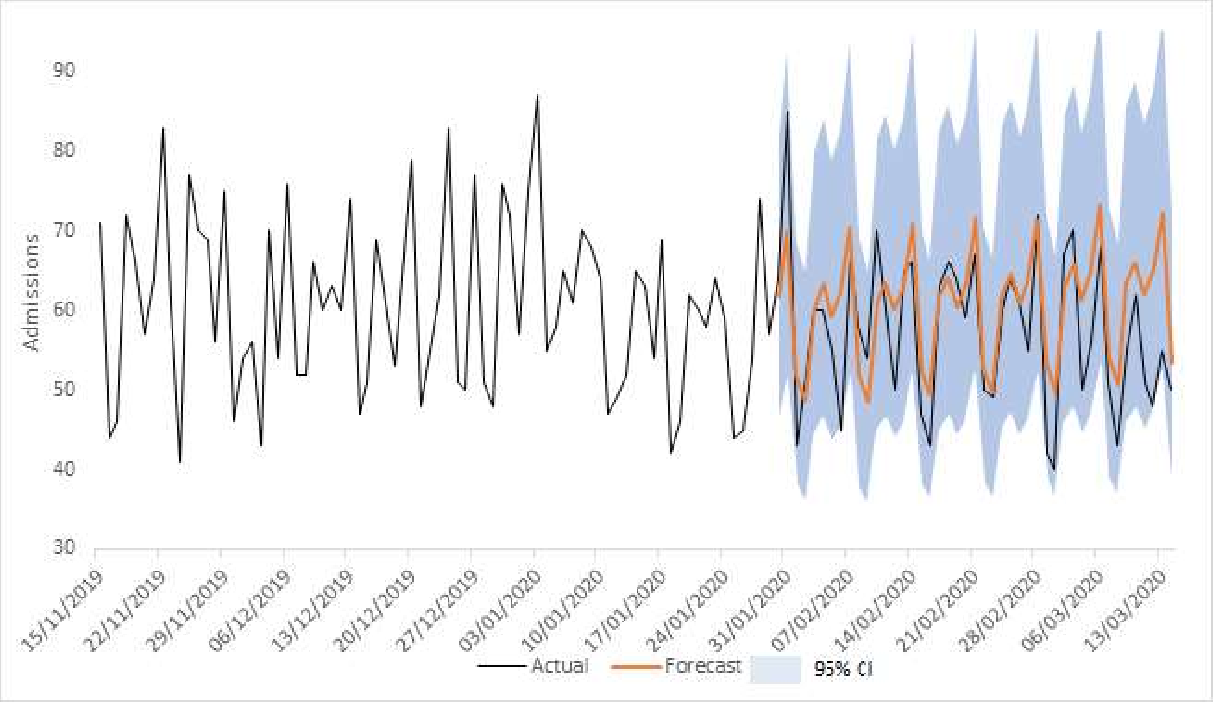

Figure A8: Model 9 (over 48 hour length of stay surgery admissions)

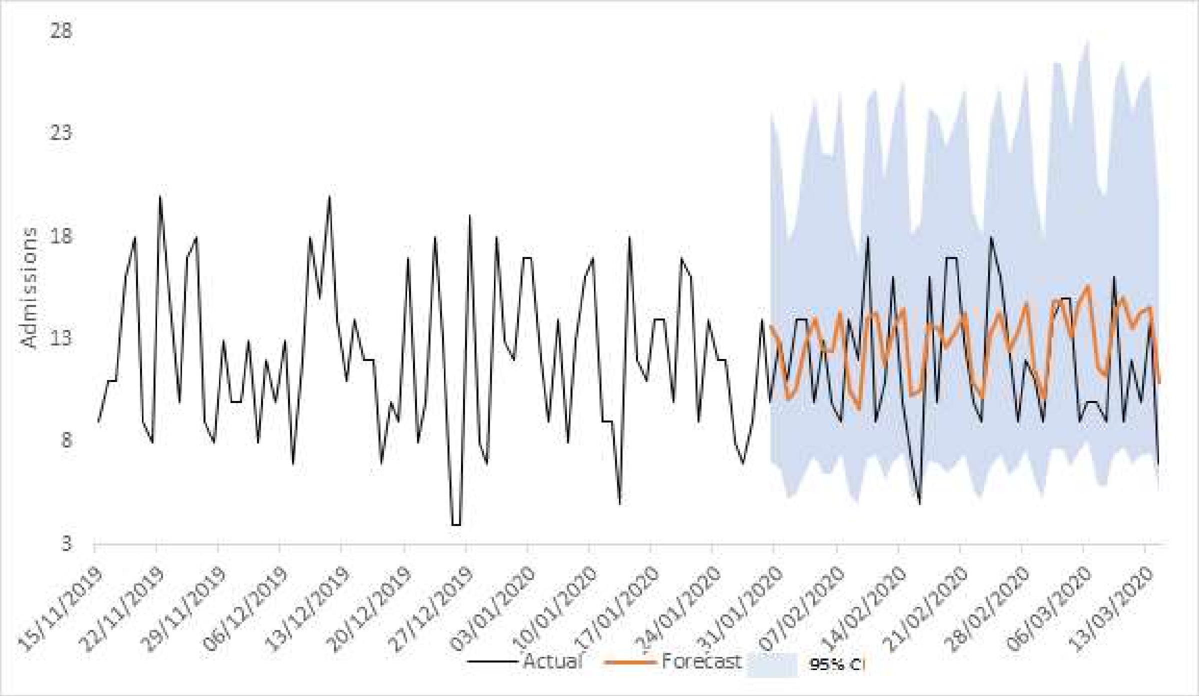

Figure A9: Model 10 (all bed occupancy)

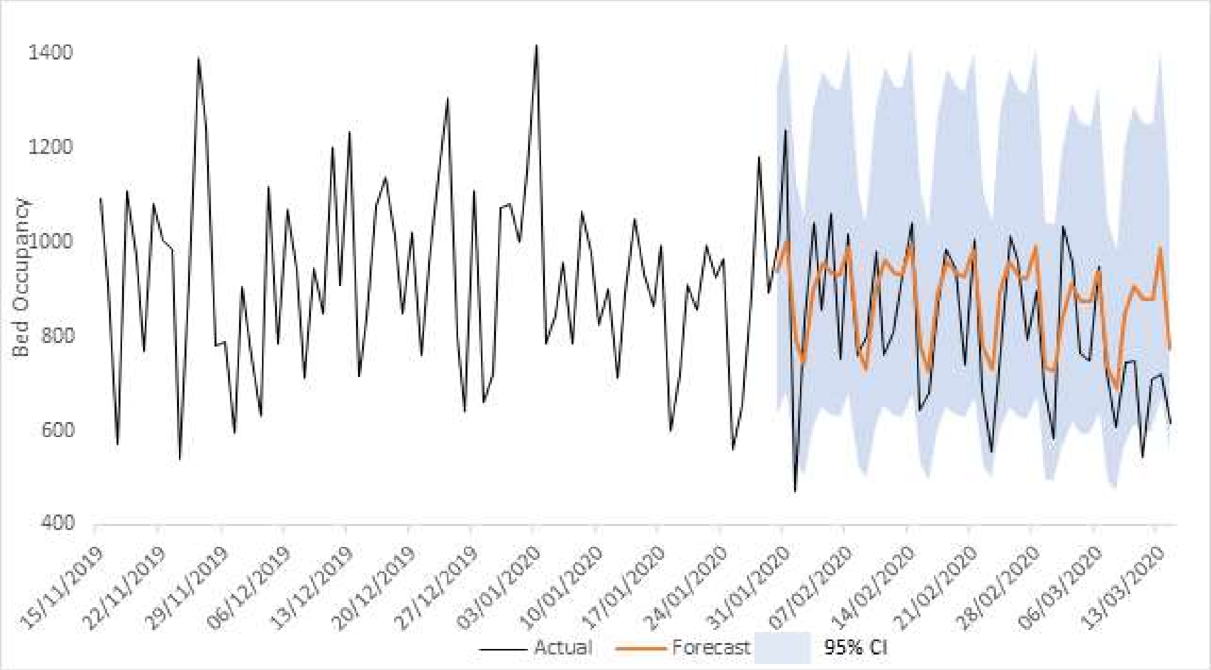

Figure A10: Model 11 (medicine bed occupancy)

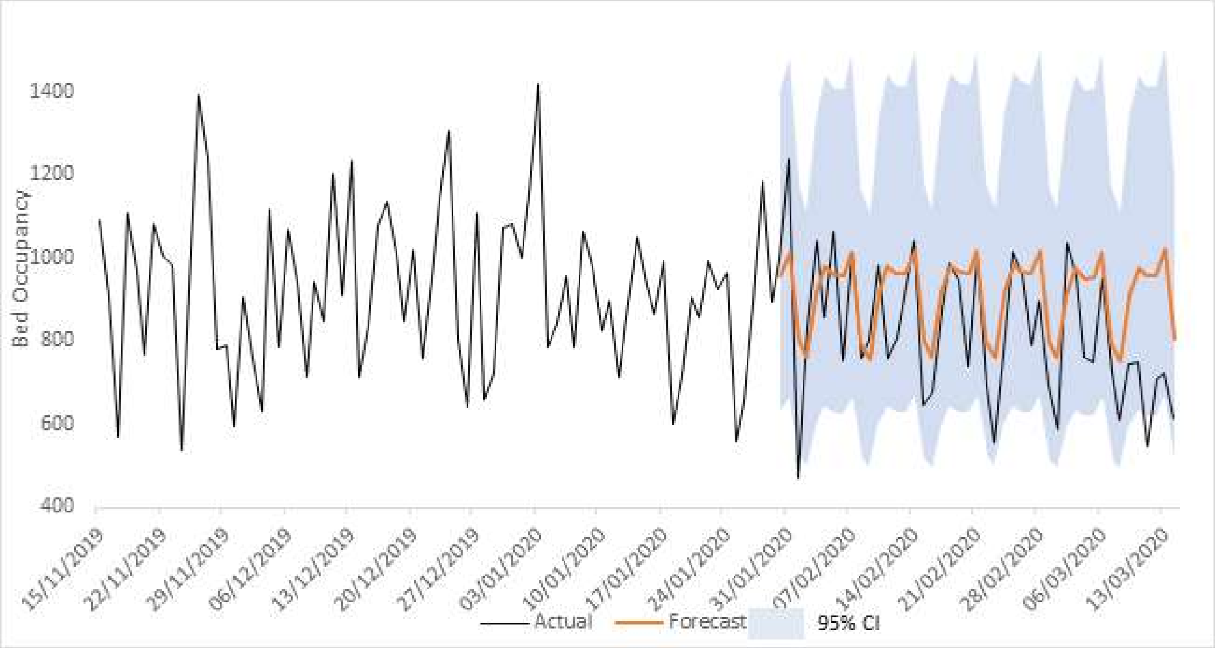

Figure A11: Model 12 (surgery bed occupancy)

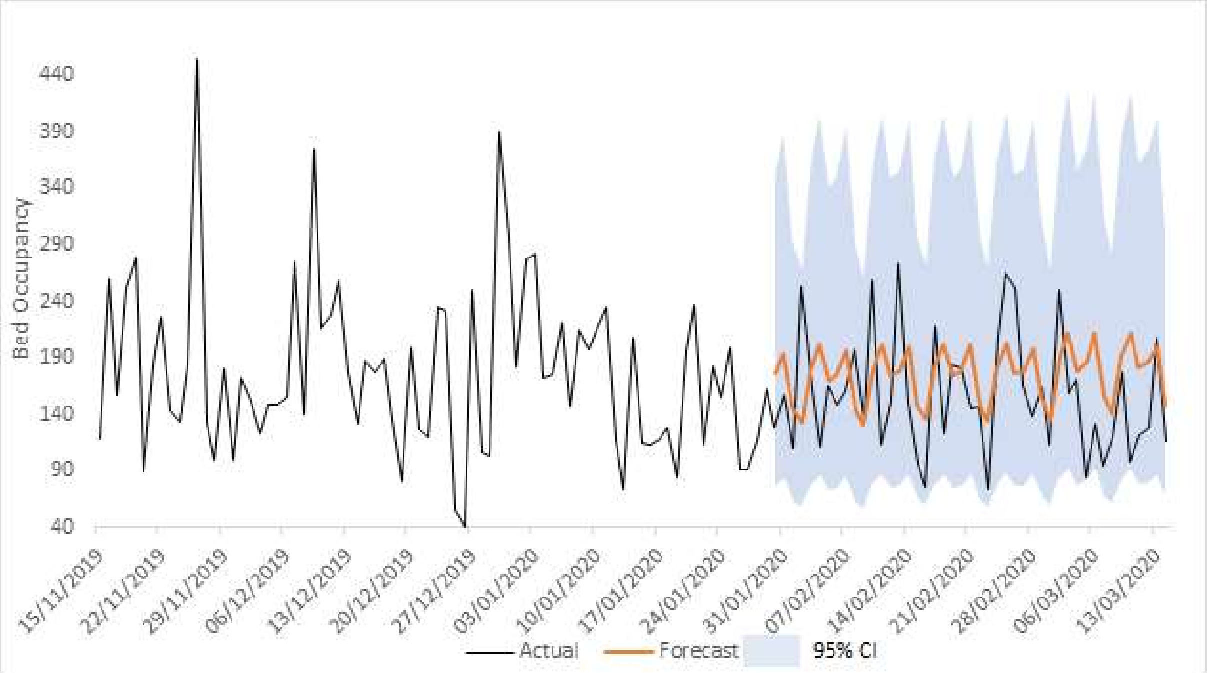

Figure A12: Model 13 (under 48 hour length of stay bed occupancy)

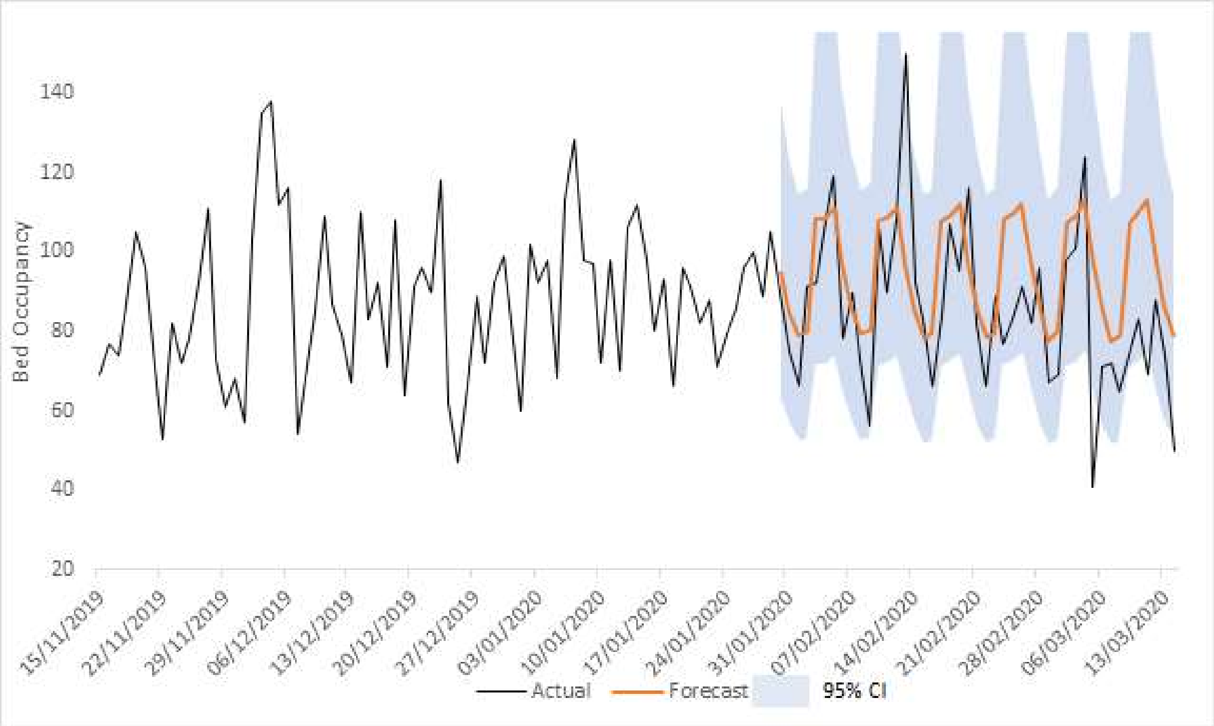

Figure A13: Model 14 (over 48 hour length of stay bed occupancy)

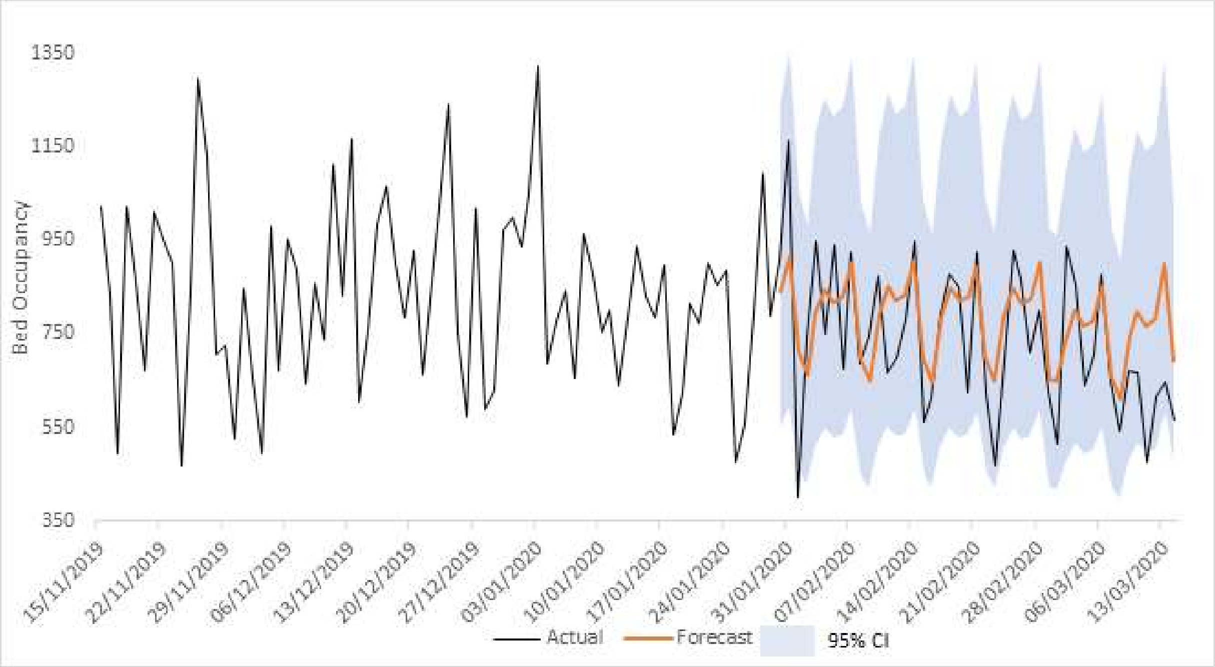

Figure A14: Model 15 (under 48 hour length of stay medicine bed occupancy)

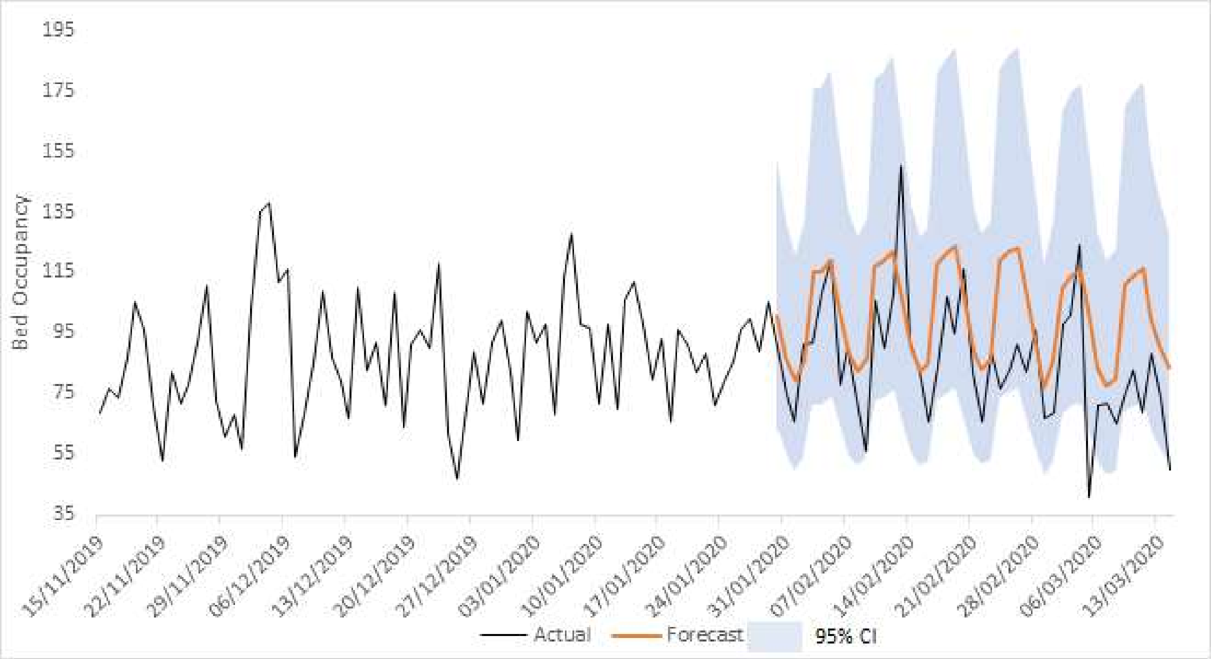

Figure A15: Model 16 (under 48 hour length of stay surgery bed occupancy)

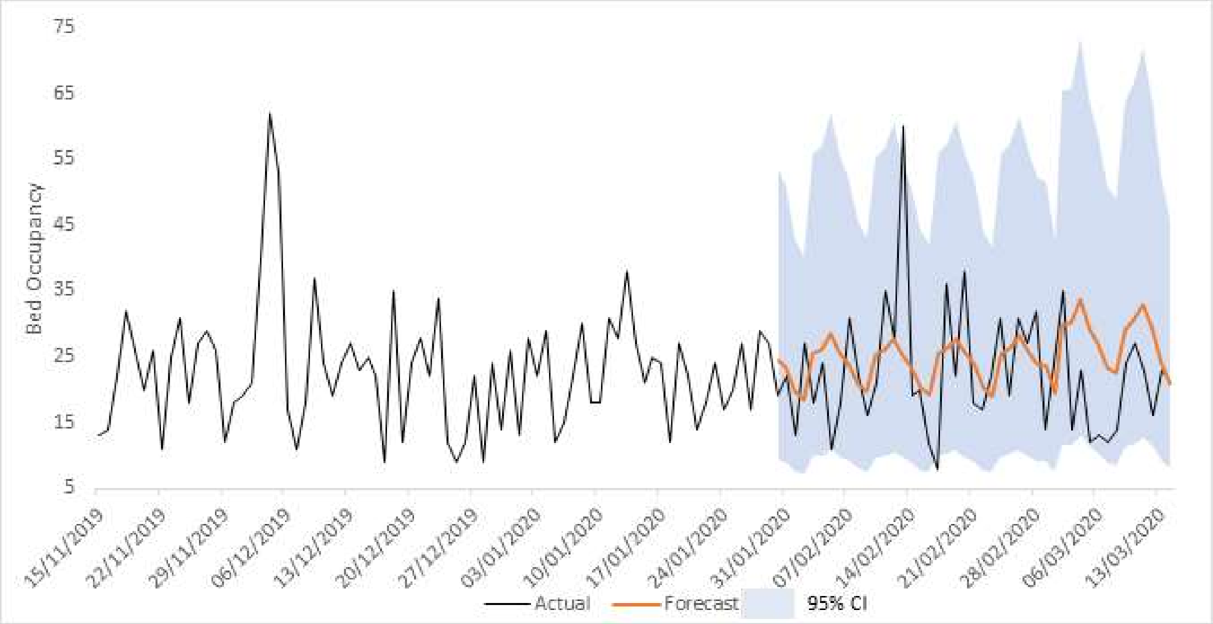

Figure A16: Model 17 (over 48 hour length of stay medicine bed occupancy)

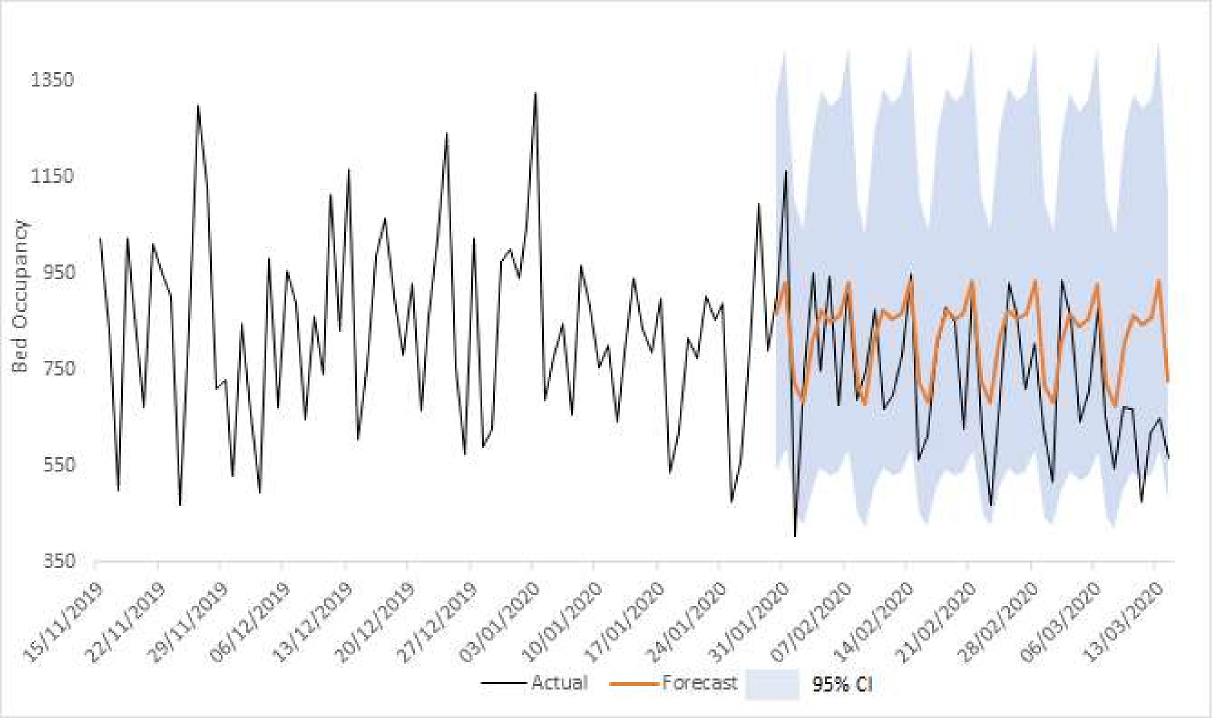

Figure A17: Model 18 (over 48 hour length of stay surgery bed occupancy)

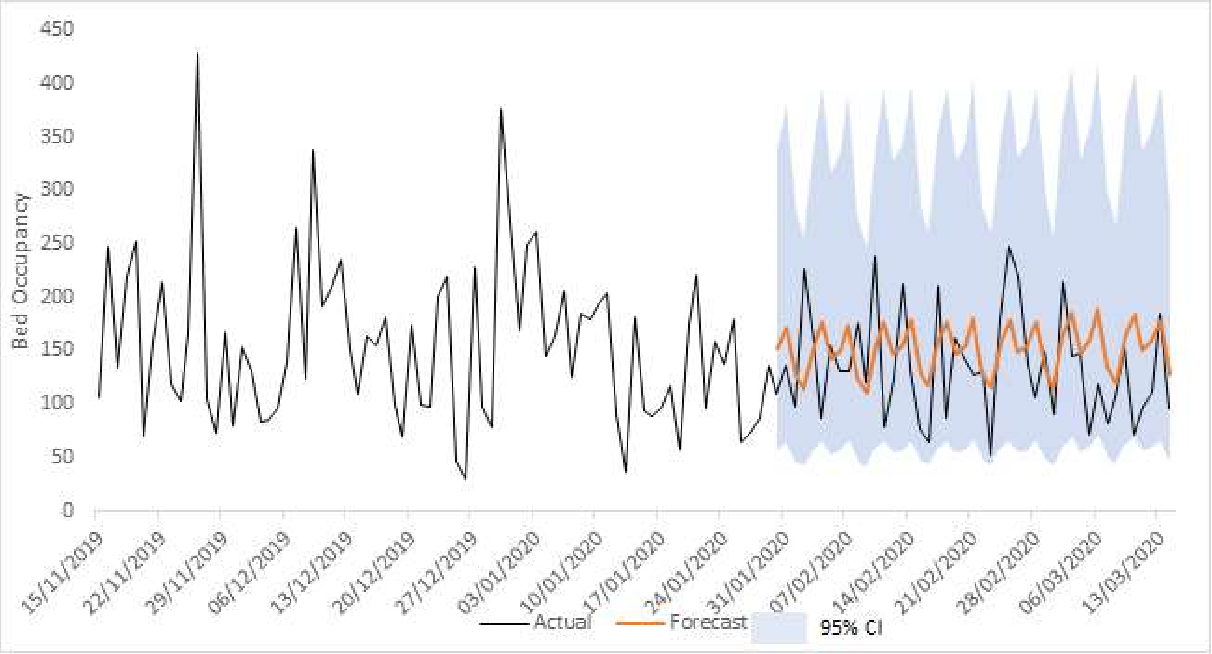

For peer review only

```
#clear the environment of all data
rm(list = ls())

## install necessary packages (Can skip this step if you have them)
install.packages("readstata13", "Hmisc", "DataCombine", "lmtest","tidyverse",
"scales", "forecast", "zoo", "tempdisagg", "imputeTS", "lubridate", "aTSA",
"tseries", "gridExtra", "broom", "knitr", "dplyr", "pixedust")


#tidy regression output libraries
library(broom)
library(knitr)
require(broom)
require(knitr)
library(dplyr) ## this is the library with 'pipes' %>%
library(pixiedust)



## Packages needed
## If you don't have a package, use 'install.packages("NAME OF PACKAGE HERE")
#bring in stata data
library(readstata13)

#lag function
library(Hmisc)

#Row insertion library for missing data problem
library(DataCombine)

#Some tests are in these libraries
library(lmtest)


#tidyverse includes many other useful libraries for formatting data, scales
includes date formatting
library(tidyverse)
library(scales)

#forecasting and time series libraries
library(forecast)
library(zoo)
library(tempdisagg)
library(imputeTS)
library(lubridate)
library(aTSA)
library(tseries)

#laying out graph library
library(gridExtra)
```

For peer review only

```
#tidy regression output libraries
library(broom)
library(knitr)
require(broom)
require(knitr)
library(dplyr) ## this is the library with 'pipes' %>%
library(pixiedust)



# Set file path for forecasting data

setwd("YOUR WORKING DIRECTORY HERE")


# Transfer Stata dataset into R datasets. Use the relevant dataset in 'hosp_data'
hosp_data = read.dta13("COLLAPSED HOSPITAL DATA HERE", convert.factors = TRUE,
generate.factors = TRUE, nonint.factors = TRUE)
temp = read.csv("TEMPERATURE WITH SUFFICIENT LAG (ONE YEAR BEFORE HOSPITAL DATA
START) HERE")
precip = read.dta13("PRECIPITATION WITH SUFFICIENT LAG (ONE YEAR BEFORE HOSPITAL
DATA START) HERE", convert.factors = TRUE, generate.factors = TRUE, nonint.factors
= TRUE)

#Use below if temperature/precipitation data is in dta format from stata, but new
code to combine in R is much more efficient.
#temp = read.dta13("temperatures_lag.dta", convert.factors = TRUE, generate.factors
= TRUE, nonint.factors = TRUE)
#precip = read.dta13("temperatures_lag.dta", convert.factors = TRUE,
generate.factors = TRUE, nonint.factors = TRUE)

#make a full date variable for the temp and precip data to match hosp_data
temp = temp %>%
  mutate(admidate=make_date(year, month, day))

precip = precip %>%
  mutate(admidate=make_date(admiyear, admimonth, admiday))


#check the plot to make sure it looks like you would expect (time series data, with
a count per day)

plot(hosp_data$id)


#pull out the length and the end date of the hospital data, for data manipulation
purposes

leng = nrow(hosp_data)
```

For peer review only

```
end_date=hosp_data$dateindex[leng]
init_date=hosp_data$dateindex[1]


#check the data if there's a huge outlier (often for some of the bed occupancy
subforecasts)

plot(hosp_data$id)

#find the position of the outlier, then make it an average of the previous week's
occupancy/admissions.
#CHECK: max admissions/max occupancy with NBT

outval = which.max(hosp_data$id) #if the outlier is higher
#outval = which.min(hosp_data$id) #if the outlier is lower (this one is commented
out)
hosp_data$id[outval] #displays the value
hosp_data$id[outval] = as.integer(sum(hosp_data$id[(outval-7):(outval-1)])/7)
#makes the outlier an average of the previous 7 days' occupancy/admissions


#sort out the lag temperature - the temperature and precipitation files should have
one year more data in the past, so that you can do this part
#if we didn't do it this way, if we did last week's temperature, we'd have to
predict the weather accurately if our forecast horizon exceeded one week
#in conclusion, since we're not meteorologists, it's probably better to just use
last year's, so we can use whatever horizon we'd like up to a year

temp$last_year = Lag(temp$avg_temp, 365)

#lag precipitation

precip$last_year = Lag(precip$rainfall, 365)

#overwrite the dataframes so that they're the same length as the hospital
admissions data
#the models won't work if the inputs have different lengths

precip= subset(precip, admidate>=init_date & admidate<=end_date)
temp = subset(temp, admidate>=init_date & admidate<=end_date)

#Looking at missingness in the temperature and precipitation data
#Some days may be missing, (NOT IN CURRENT TRANCHE) and missing values need to be
imputed
#If there are no missing values, it will say 'integer (empty)' in the environment

missing_t = which(is.na(temp$last_year))
missing_p = which(is.na(precip$last_year))

#A quick fix for NAs in the temperature and precipitation data, if there
```

For peer review only

```
#are missing values, but don't run if there aren't.

temp$last_year[missing_t] = na.spline(temp$last_year)
precip$last_year[missing_t] = na.spline(precip$last_year)

#Check for missing dates - for subforecasts this might be an issue for those with
small numbers
#Make sure to put 0s in when preparing subforecast data in Stata rather than
allowing missingness
#If there are missing dates, go back to the preparation stage.
#It will say 'Date' num(0) in the global environment if there are no missing dates

## THIS SECTION IS ONLY FOR IF THERE ARE MISSING COUNT DATA ON ANY DAY ##

missing_d = as.Date(setdiff(precip$admidate,hosp_data$dateindex))

#If they are missing, add them in as '0' values (missingness means there was
#no occupancy on those dates, as far as I understand).

#First create a new dataframe with the information that's missing
#Name the columns Add the new dataframe to the end of the old one, then sort
("arrange")

add_miss = data.frame(missing_d, 1, month(missing_d), year(missing_d),
day(missing_d), wday(missing_d), 0)
names(add_miss)= c("dateindex", "id", "month", "year", "day", "dow", "yearmonth")
hosp_data=rbind(hosp_data, add_miss)
hosp_data=hosp_data %>% arrange(dateindex)
rm(add_miss)


leng=nrow(hosp_data)

#tidy up a bit
rm(missing_d,missing_t,missing_p)

#check and record the mean and standard deviation of the data
mean(hosp_data$id)
sd(hosp_data$id)


#Transform the dataframes into time series objects, with a daily 'seasonality'

hospts <- msts(hosp_data, seasonal.periods = c(7,365.25), ts.frequency = 7)
tempts <- msts(temp, seasonal.periods = c(7,365.25), ts.frequency = 7)
precipts <- msts(precip, seasonal.periods = c(7,365.25), ts.frequency = 7)



#Check ACF plot of the count to confirm the presence of autocorrelation.
```

For peer review only

```
#If the values fall between the blue lines (p<0.05), then the data are not
#likely to be autocorrelated. If they do fall outside the lines, they are likely to
be.
#This uses a lag of 21 days
# "The ACF of stationary data drops to zero relatively quickly"
# "The ACF of non-starionary data decreases slowly"

acf(hospts[,"id"], lag = 21)

#Check Partial ACF plot to confirm that the autocorrelation is or isn't a result of
the
#autocorrelation of the first lag carrying through (if it is, the spikes on the
PACF will
#peak at the first lag, then be in between the blue lines (p <0.05))
# "A partial autocorrelation is the amount of correlation between a variable
#and a lag of itself that is not explained by correlations at all
lower-order-lags."
#This uses a lag of 21 days

pacf(hospts[,"id"], lag = 21)

#Fit a regression model to formally test for a lagged autocorrelation in the
residuals

#create a time variable
timef <- seq_along(hospts[,"id"])

#fit a linear regression to model the series as a function of time
lin_reg = tslm(hospts[,"id"] ~ timef)

#Durbin-Watson test - check for lag-1 autocorrelated residuals
#H0 = residuals do not have lag-1 auotocorrelation
#small p -> there is evidence for lag-1 autocorrelation

dwtest(lin_reg)

#tidy up
rm(timef, lin_reg)


#Determine stationarity of the data (it is non-stationary if it has a trend,
seasonal
#effect, or any systematic change in mean and variance


#Seasonal decomposition graphs (including weekly and annual seasonality)
#These are decomposed to the full sample and also financial years

## You may need to adjust these to the years of your data (the label), but the
actual end of the window
```

For peer review only

```
##is one year long##

hospts[,"id"] %>% mstl(lambda = 0) %>% autoplot() + xlab("week") +
  ggtitle("Estimated components (full sample)")

#2015-2016 (check the weeks of the year for these)

hospts[,"id"] %>% mstl(lambda = 0) %>% window(end = 52) %>% autoplot(facet=TRUE) +
xlab("week") +
  ggtitle("Estimated components (2015-2016)")

#2016-2017 (check the weeks of the year for these)

hospts[,"id"] %>% mstl(lambda = 0) %>% window(start = 53, end = 105) %>%
autoplot(facet=TRUE) +
  xlab("week") + ggtitle("Estimated components (2016-2017)")

#2017-2018 (check the weeks of the year for these)

hospts[,"id"] %>% mstl(lambda = 0) %>% window(start = 106, end = 158) %>%
autoplot(facet=TRUE) +
  xlab("week") + ggtitle("Estimated components (2017-2018)")

#2018-2019 (check the weeks of the year for these)

hospts[,"id"] %>% mstl(lambda = 0) %>% window(start = 159, end = 211) %>%
autoplot(facet=TRUE) +
  xlab("week") +ggtitle("Estimated components (2018-2019)")

#2019-'2020' (check the weeks of the year for these -
#keep in mind that we currently don't have all the data for this financial year

hospts[,"id"] %>% mstl(lambda = 0) %>% window(start = 212, end = 250) %>%
autoplot(facet=TRUE) +
  xlab("week") +ggtitle("Estimated components (2019-2020)")

# Check the seasonality of the data - annual seasonality often includes Christmas
(push to discharge before Christmas) and at times Easter
# There is also usually weekly seasonality. Check the trend of the data as well.

#Conduct an augmented Dickey-Fuller test (ADF) test to formally test for
stationarity
#H0 = non-stationary, H1 = stationary. low p value = reject the null

adf.test(hospts[,"id"])

#If the data are not stationary (i.e. high p), transform them to be stationary
(differencing)
#if the adf test rejects the null, then don't difference as they are stationary.
#if you difference once, and it still isn't stationary, consider differencing the
```

For peer review only

```
differenced data
#or, log the data (if the variance is unstable). Currently commented out.

#hospts = diff(hospts)

#Onto modelling, creating the data. Please note if you differenced the data, you
will need to change the variable names:

# Create deterministic trend and log deterministic trend

trend <- seq_along(hospts[,"id"])

# Create dummy daily variables

mon <- ifelse(hospts[,"dow"] == 1, 1, 0) # create dummy variable equal to 1 if
Monday and 0 otherwise
tue <- ifelse(hospts[,"dow"] == 2, 1, 0)
wed <- ifelse(hospts[,"dow"] == 3, 1, 0)
thu <- ifelse(hospts[,"dow"] == 4, 1, 0)
fri <- ifelse(hospts[,"dow"] == 5, 1, 0)
sat <- ifelse(hospts[,"dow"] == 6, 1, 0)

# Create dummmy(ies) for Christmas effect

chris <- ifelse(hospts[,"day"] == 25 & hospts[,"month"] == 12, 1, 0)
chrisweek <- ifelse(hospts[,"day"] %in% c(23, 24, 25, 26, 27) & hospts[,"month"] ==
12, 1, 0)
easter2015 <- ifelse(hospts[,"day"] %in% c(3,4,5,6) & hospts[,"month"] == 4, 1, 0
& hospts[,"year"] == 2015)
easter2016 <- ifelse(hospts[,"day"] %in% c(25,26,27,28) & hospts[,"month"] == 3, 1,
0  & hospts[,"year"] == 2016)
easter2017 <- ifelse(hospts[,"day"] %in% c(14,15,16,17) & hospts[,"month"] == 4, 1,
0  & hospts[,"year"] == 2017)
easter2018a <- ifelse(hospts[,"day"] %in% c(30,31) & hospts[,"month"] == 3, 1, 0  &
hospts[,"year"] == 2018)
easter2018b <- ifelse(hospts[,"day"] %in% c(1,2) & hospts[,"month"] == 4, 1, 0  &
hospts[,"year"] == 2018)
easter2018 <- easter2018a + easter2018b
easter2019 <- ifelse(hospts[,"day"] %in% c(19,20,21,22) & hospts[,"month"] == 4, 1,
0  & hospts[,"year"] == 2019)

easterall <- easter2015 + easter2016 + easter2017 + easter2018 + easter2019

bhm2015 <- ifelse(hospts[,"day"] %in% c(4,25) & hospts[,"month"] == 5, 1, 0  &
hospts[,"year"] == 2015)
bha2015 <- ifelse(hospts[,"day"] %in% c(31) & hospts[,"month"] == 8, 1, 0  &
hospts[,"year"] == 2015)
bh2015 <- bhm2015 + bha2015
bhj2016 <- ifelse(hospts[,"day"] %in% c(1) & hospts[,"month"] == 1, 1, 0  &
hospts[,"year"] == 2016)
```

For peer review only

```
bhm2016 <- ifelse(hospts[,"day"] %in% c(2,30) & hospts[,"month"] == 5, 1, 0  &
hospts[,"year"] == 2016)
bha2016 <- ifelse(hospts[,"day"] %in% c(29) & hospts[,"month"] == 8, 1, 0  &
hospts[,"year"] == 2016)
bh2016 <- bhj2016 + bhm2016 + bha2016
bhj2017 <- ifelse(hospts[,"day"] %in% c(2) & hospts[,"month"] == 1, 1, 0  &
hospts[,"year"] == 2017)
bhm2017 <- ifelse(hospts[,"day"] %in% c(1,29) & hospts[,"month"] == 5, 1, 0  &
hospts[,"year"] == 2017)
bha2017 <- ifelse(hospts[,"day"] %in% c(28) & hospts[,"month"] == 8, 1, 0  &
hospts[,"year"] == 2017)
bh2017 <- bhj2017 + bhm2017 + bha2017
bhj2018 <- ifelse(hospts[,"day"] %in% c(1) & hospts[,"month"] == 1, 1, 0  &
hospts[,"year"] == 2018)
bhm2018 <- ifelse(hospts[,"day"] %in% c(7,28) & hospts[,"month"] == 5, 1, 0  &
hospts[,"year"] == 2018)
bha2018 <- ifelse(hospts[,"day"] %in% c(27) & hospts[,"month"] == 8, 1, 0  &
hospts[,"year"] == 2018)
bh2018 <- bhj2018 + bhm2018 + bha2018
bhj2019 <- ifelse(hospts[,"day"] %in% c(1) & hospts[,"month"] == 1, 1, 0  &
hospts[,"year"] == 2019)
bhm2019 <- ifelse(hospts[,"day"] %in% c(6,27) & hospts[,"month"] == 5, 1, 0  &
hospts[,"year"] == 2019)
bha2019 <- ifelse(hospts[,"day"] %in% c(26) & hospts[,"month"] == 8, 1, 0  &
hospts[,"year"] == 2019)
bh2019 <- bhj2019 + bhm2019 + bha2019

bhall <- bh2015 + bh2016 + bh2017 + bh2018 + bh2019

hollall <- easterall + bhall +chrisweek

#clean up the extra variables we don't need

rm(bhm2015,bha2015,bh2015,bhj2016,bhm2016,bha2016,bhj2017,bhm2017,bha2017,bhj2018,b
hm2018,bha2018,bhj2019,bhm2019,bha2019,chris)
rm(easter2015,easter2016,easter2017,easter2018,easter2018a,easter2018b,easter2019)


# Create a matrix of regressors for trend and daily dummies

trend_days <- tibble(trend, mon, tue, wed, thu, fri, sat, chrisweek, easterall,
bhall, tempts[,"last_year"], precipts[,"last_year"]) # create matrix of regressors:
trend + daily dummies
trend_days_noCLIM <- tibble(trend, mon, tue, wed, thu, fri, sat, chrisweek,
hollall) # create matrix of regressors: trend + daily dummies


#create the time series with the regressors included

seasonality <- msts(trend_days, seasonal.periods = c(7,365), ts.frequency = 7)
```

For peer review only

```
seasonality_noCLIM <-  msts(trend_days_noCLIM, seasonal.periods = c(7,365),
ts.frequency = 7)


# Estimate models with and without climatic data - other model types can be found
in the code graveyard

fit_trend <- auto.arima(hospts[,"id"], d=0, xreg=cbind(seasonality), lambda=0,
biasadj=TRUE)
fit_trend_noCLIM <- auto.arima(hospts[,"id"], d=0, xreg=cbind(seasonality_noCLIM),
lambda=0, biasadj=TRUE)

#Checks for the deterministic trend

summary(fit_trend) #to get model fit parameters manually
res_trend <- residuals(fit_trend)
checkresiduals(fit_trend, points = FALSE)
ggPacf(res_trend, lag=70)
adf.test(res_trend)
trend_nonseas = c(fit_trend$arma[1], fit_trend$arma[6], fit_trend$arma[2]) #pull
out the nonseasonal (first part) ARIMA parameters
trend_seas = c(fit_trend$arma[3], fit_trend$arma[7], fit_trend$arma[4]) #pull out
the seasonal (second part) ARIMA parameters

#Checks for the deterministic trend without climate data
summary(fit_trend_noCLIM) #to get model fit parameters manually
res_trend_noCLIM <- residuals(fit_trend_noCLIM)
checkresiduals(fit_trend_noCLIM, points = FALSE)
ggPacf(res_trend_noCLIM, lag=70)
adf.test(res_trend)
trend_noCLIM_nonseas = c(fit_trend_noCLIM$arma[1], fit_trend_noCLIM$arma[6],
fit_trend_noCLIM$arma[2]) #pull out the nonseasonal (first part) ARIMA parameters
trend_noCLIM_seas = c(fit_trend_noCLIM$arma[3], fit_trend_noCLIM$arma[7],
fit_trend_noCLIM$arma[4]) #pull out the seasonal (second part) ARIMA parameters


# Forecasting on training and test sets - 6 week horizon

train.hospts <- subset(hospts[,"id"], end = length(hospts[,"id"]) - 45)
test.hospts <- subset(hospts[,"id"], start=length(hospts[,"id"])-44)

lentrain=length(train.hospts)


#xlim=c(230,241) to examine the training/test split
train.hospts %>% autoplot()+
  autolayer(test.hospts, color = "red")


#deterministic trend
```

For peer review only

```
trend_model <- Arima(train.hospts, order=trend_nonseas, seasonal=trend_seas, 
include.constant = TRUE,
                     xreg = cbind(seasonality[1:lentrain,]),
                     lambda=0, biasadj=TRUE)

trend_fcast <- forecast(trend_model, level = c(95), xreg = 
cbind(seasonality[(lentrain+1):leng,]))

trend_fcast %>% autoplot() +
  autolayer(test.hospts, series="Actual value", color = 'darkorange') +
  guides(colour=guide_legend(title="")) +
  ggtitle("Deterministic trend model") +
  ylab("Daily admissions")


#deterministic trend no climate data

trend_model_noCLIM <- Arima(train.hospts, order=trend_noCLIM_nonseas, 
seasonal=trend_noCLIM_seas, include.constant = TRUE,
                     xreg = cbind(seasonality_noCLIM[1:lentrain,]),
                     lambda=0, biasadj=TRUE)

trend_fcast_noCLIM <- forecast(trend_model_noCLIM, level = c(95), xreg = 
cbind(seasonality_noCLIM[(lentrain+1):leng,]))

trend_fcast_noCLIM %>% autoplot() +
  autolayer(test.hospts, series="Actual value", color = 'darkorange') +
  guides(colour=guide_legend(title="")) +
  ggtitle("Deterministic trend model no climate") +
  ylab("Daily admissions")




# assess the accuracy - for AIC/BIC check model summaries - summary(trend_model)
etc

accuracy(trend_model$fitted, hospts[,"id"])
accuracy(trend_model_noCLIM$fitted, hospts[,"id"])


#residual differences tables
diff_trend = hospts[(lentrain+1):leng,"id"] - trend_fcast$mean
date = as.character(paste(hospts[(lentrain+1):leng,"year"], 
hospts[(lentrain+1):leng,"month"],  hospts[(lentrain+1):leng,"day"], sep="/"))
dow = factor(as.character(hospts[(lentrain+1):leng,"dow"]), levels = 
c(0,1,2,3,4,5,6), labels = c("Sun", "Mon", "Tues", "Weds", "Thurs", "Fri", "Sat"))

#admissions values
```

For peer review only

```
acc_trend = abs(diff_trend) <= (0.2*mean(hosp_data$id))
acc_trend_s = abs(diff_trend) < (0.1*mean(hosp_data$id))


##put it into a nice format
diff_trend_df=data.frame("date" = date, "dow" = dow , "actual" =
hospts[(lentrain+1):leng,"id"], "predicted" = trend_fcast$mean, "difference"=
diff_trend, "lower95" = trend_fcast$lower, "upper95" = trend_fcast$upper,
"accuracybig" = acc_trend,  "accuracybetter" = acc_trend_s)

##write to a csv (easier to copy)- when you open it in Excel the date column will
look weird - just make sure to format it as a date in Excel and pick a format that
shows up properly - also you can reduce the df in excel
write.csv(diff_trend_df, file="Trend_diff.csv", row.names=FALSE)

##write all the data to a csv for making graphs
write.csv(hosp_data, file="all_data.csv", row.names=FALSE)
```

For peer review only

Appendix 3: Model specifications and AICs for models with and without climatic data

Table A3.1: Model specifications. Guide to acronyms: a- admissions, bo- bed occupancy, o48 – over 48 hour length of stay, u48 – under 48 hour length of stay, med – medicine, surg - surgery

A/BO	Time	Climate	Speci alty	SARIMA	RMSE	MAPE	mean	sd	AIC
a	all	y	all	(3,0,0)(1,0,0)	9.59	8.66	90.52	15.02	-2069.49
a	all	n	all	(2,0,2)(1,0,0)	9.50	8.59	90.52	15.02	-2043.83
a	all	y	med	(3,0,2)(2,0,2)	8.98	9.44	79.14	19.32	-1817.45
a	all	n	med	(3,0,2)(2,0,2)	8.98	9.43	79.14	19.32	-1813.09
a	o48	y	all	(3,0,1)(1,0,1)	7.22	10.78	55.14	10.04	-1496.64
a	o48	n	all	(2,0,3)(1,0,0)	7.25	10.83	55.14	10.04	-1495.55
a	o48	y	med	(5,0,1)(2,0,0)	6.88	11.97	48.55	12.06	-1242.32
a	o48	n	med	(5,0,1)(2,0,0)	6.88	11.97	48.55	12.06	-1237.50
a	o48	y	surg	(3,0,3)(1,0,2)	3.40	27.45	11.87	3.74	678.12
a	o48	n	surg	(4,0,4)(0,0,2)	3.41	27.47	11.87	3.74	681.20
a	all	y	surg	(1,0,2)(0,0,0)	4.59	19.86	20.65	5.31	-7.37
a	all	n	surg	(2,0,1)(2,0,0)	4.58	19.87	20.65	5.31	-1.96
a	u48	y	all	(1,0,0)(1,0,0)	6.81	16.53	35.38	9.06	-471.22
a	u48	n	all	(2,0,3)(1,0,0)	6.78	16.47	35.38	9.06	-463.39
a	u48	y	med	(4,0,0)(2,0,0)	6.45	18.64	30.59	10.21	-177.19
a	u48	n	med	(4,0,0)(2,0,0)	6.46	18.67	30.59	10.21	-176.50
a	u48	y	surg	(0,0,1)(0,0,0)	7.82	44.19	20.99	8.39	1583.52
a	u48	n	surg	(0,0,2)(0,0,0)	7.85	44.51	20.99	8.39	1583.97
bo	all	y	all	(0,0,1)(1,0,0)	158.24	15.57	849.71	179.77	-620.10
bo	all	n	all	(1,0,3)(1,0,0)	158.17	15.57	849.71	179.77	-618.59
bo	all	y	med	(2,0,1)(0,0,0)	151.69	17.06	760.03	189.22	-423.18
bo	all	n	med	(2,0,1)(0,0,0)	151.75	17.06	760.03	189.22	-418.86
bo	o48	y	all	(0,0,1)(1,0,0)	159.55	17.60	767.41	177.20	-330.94
bo	o48	n	all	(1,0,3)(1,0,0)	159.42	17.62	767.41	177.20	-330.48
bo	o48	y	med	(2,0,1)(0,0,0)	152.70	19.15	689.20	181.24	-153.63
bo	o48	n	med	(2,0,1)(0,0,0)	152.76	19.15	689.20	181.24	-149.13
bo	o48	y	surg	(0,0,1)(0,0,0)	64.95	44.22	141.63	67.49	1661.21
bo	o48	n	surg	(0,0,1)(0,0,0)	65.09	44.36	141.63	67.49	1662.07
bo	all	y	surg	(0,0,1)(0,0,0)	64.50	35.40	162.62	67.71	1248.15
bo	all	n	surg	(0,0,1)(0,0,0)	64.63	35.45	162.62	67.71	1248.44
bo	u48	y	all	(3,0,0)(2,0,1)	15.72	16.41	82.30	21.06	-468.39
bo	u48	n	all	(3,0,0)(2,0,1)	15.73	16.42	82.30	21.06	-465.24
bo	u48	y	med	(4,0,0)(2,0,0)	14.95	18.63	70.83	23.95	-170.75
bo	u48	n	med	(4,0,0)(2,0,1)	14.88	18.61	70.83	23.95	-164.97
bo	u48	y	surg	(0,0,1)(0,0,0)	7.82	44.19	21.00	8.38	1583.52
bo	u48	n	surg	(0,0,2)(0,0,0)	7.85	44.51	21.00	8.38	1583.97

For peer review only

Appendix 4: Sensitivity Analyses

The results of the sensitivity analyses are presented here. Two other horizons were tested: One from November to December of 2019, and one from May to June of 2020. These analyses were performed on the overall admissions data.

Table A4.1: Model specifications and accuracy

Model	SARIMA (p,d,q)(P, D,Q)	Model accuracy (strict/moderate thresholds)	MAPE	RMSE	Mean of Outcome	SD of outcome	AIC
Dec 2019	(2,0,2) (1,0,0)	75.6%/93.3%	8.57	9.43	90.09	14.97	-1933.76
June 2020	(3,0,1) (1,0,1)	42.2%/77.8%	8.88	9.67	89.28396	15.79	-2063.21

Figure A4.1: Forecast versus Actual values for December 2019 Horizon.

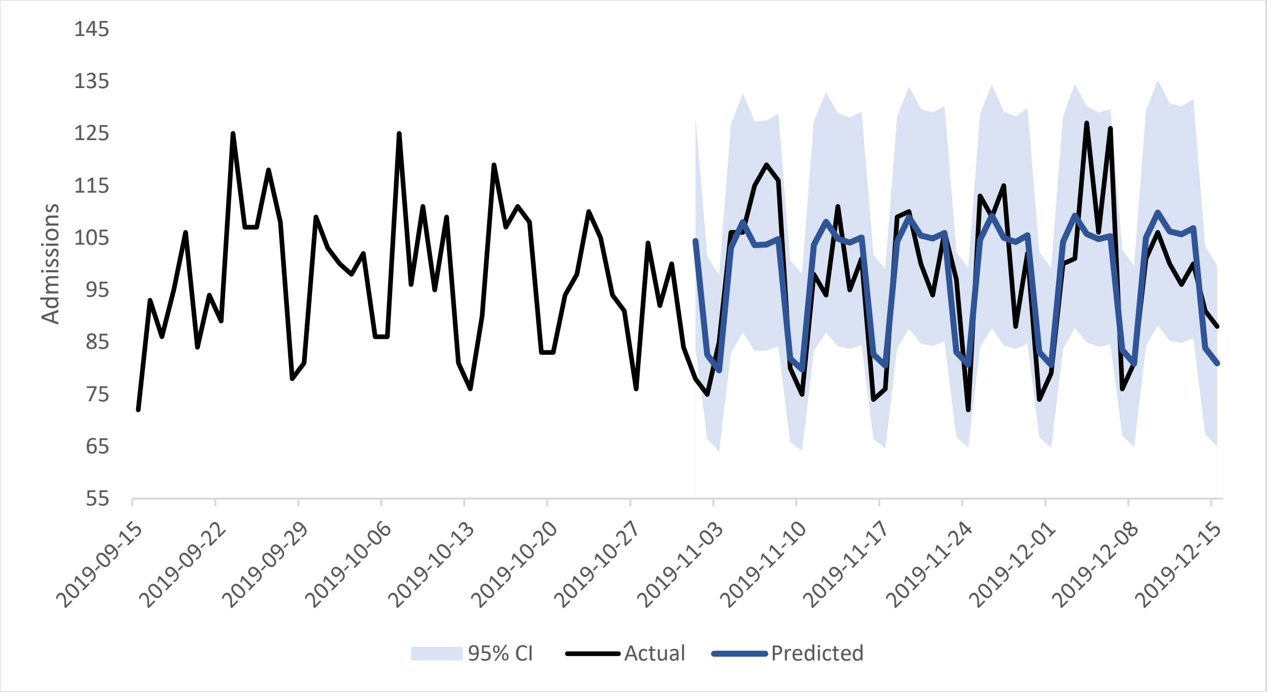

Figure A4.2: Forecast versus Actual values for June 2020 Horizon

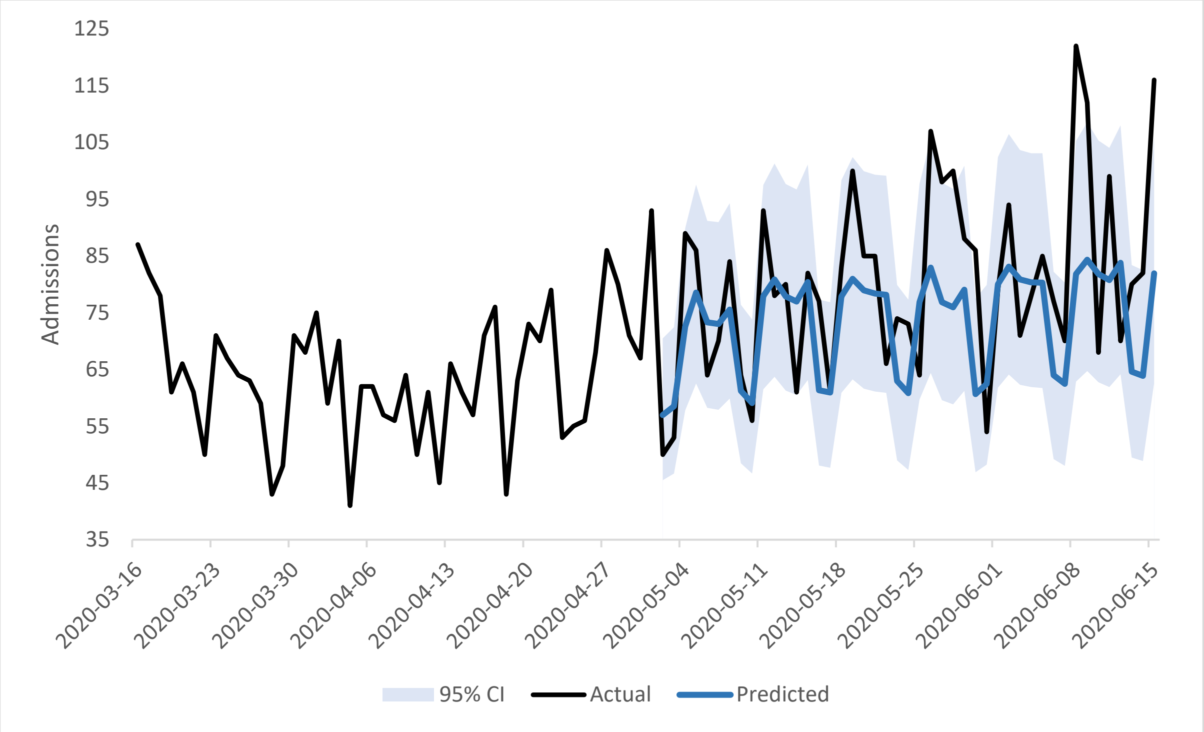
125
115
105
95
85
75
65
55
45
35
Admissions
2020-03-16
2020-03-23
2020-03-30
2020-04-06
2020-04-13
2020-04-20
2020-04-27
2020-05-04
2020-05-11
2020-05-18
2020-05-25
2020-06-01
2020-06-08
2020-06-15
95% CI
Actual
Predicted

TRIPOD Checklist: Prediction Model Development

1Section/Topic	Item	Checklist Item	Page
**Title and abstract**			
Title	1	Identify the study as developing and/or validating a multivariable prediction model, the target population, and the outcome to be predicted.	1
Abstract	2	Provide a summary of objectives, study design, setting, participants, sample size, predictors, outcome, statistical analysis, results, and conclusions.	2
**Introduction**			
Background and objectives	3a	Explain the medical context (including whether diagnostic or prognostic) and rationale for developing or validating the multivariable prediction model, including references to existing models.	2-4
	3b	Specify the objectives, including whether the study describes the development or validation of the model or both.	3-4
**Methods**			
Source of data	4a	Describe the study design or source of data (e.g., randomized trial, cohort, or registry data), separately for the development and validation data sets, if applicable.	4
	4b	Specify the key study dates, including start of accrual; end of accrual; and, if applicable, end of follow-up.	4
Participants	5a	Specify key elements of the study setting (e.g., primary care, secondary care, general population) including number and location of centres.	4
	5b	Describe eligibility criteria for participants.	4
	5c	Give details of treatments received, if relevant.	N/A
Outcome	6a	Clearly define the outcome that is predicted by the prediction model, including how and when assessed.	4
	6b	Report any actions to blind assessment of the outcome to be predicted.	N/A
Predictors	7a	Clearly define all predictors used in developing or validating the multivariable prediction model, including how and when they were measured.	4,5
	7b	Report any actions to blind assessment of predictors for the outcome and other predictors.	N/A
Sample size	8	Explain how the study size was arrived at.	4
Missing data	9	Describe how missing data were handled (e.g., complete-case analysis, single imputation, multiple imputation) with details of any imputation method.	4
Statistical analysis methods	10a	Describe how predictors were handled in the analyses.	5,6
	10b	Specify type of model, all model-building procedures (including any predictor selection), and method for internal validation.	6,7
	10d	Specify all measures used to assess model performance and, if relevant, to compare multiple models.	7
Risk groups	11	Provide details on how risk groups were created, if done.	N/A
**Results**			
Participants	13a	Describe the flow of participants through the study, including the number of participants with and without the outcome and, if applicable, a summary of the follow-up time. A diagram may be helpful.	N/A
	13b	Describe the characteristics of the participants (basic demographics, clinical features, available predictors), including the number of participants with missing data for predictors and outcome.	4, 8-9
Model development	14a	Specify the number of participants and outcome events in each analysis.	8-9
	14b	If done, report the unadjusted association between each candidate predictor and outcome.	N/A
Model specification	15a	Present the full prediction model to allow predictions for individuals (i.e., all regression coefficients, and model intercept or baseline survival at a given time point).	N/A
	15b	Explain how to the use the prediction model.	N/A
Model performance	16	Report performance measures (with CIs) for the prediction model.	8-9,
**Discussion**			
Limitations	18	Discuss any limitations of the study (such as nonrepresentative sample, few events per predictor, missing data).	2, 11
Interpretation	19b	Give an overall interpretation of the results, considering objectives, limitations, and results from similar studies, and other relevant evidence.	11, 12
Implications	20	Discuss the potential clinical use of the model and implications for future research.	11, 12
**Other information**			
Supplementary information	21	Provide information about the availability of supplementary resources, such as study protocol, Web calculator, and data sets.	2,13
Funding	22	Give the source of funding and the role of the funders for the present study.	13

We recommend using the TRIPOD Checklist in conjunction with the TRIPOD Explanation and Elaboration document.

Can we accurately forecast non-elective bed occupancy and admissions in the NHS? A time-series MSARIMA analysis of longitudinal data from an NHS Trust.

Journal:	*BMJ Open*
Manuscript ID	bmjopen-2021-056523.R2
Article Type:	Original research
Date Submitted by the Author:	01-Apr-2022

Complete List of Authors:	Eyles, Emily; NIHR ARC West; University of Bristol School of Medical Sciences, Population Health Sciences<br>Redaniel, Maria Theresa; NIHR ARC West; University of Bristol School of Medical Sciences, Population Health Sciences<br>Jones, Tim; NIHR ARC West, NIHR ARC West; University of Bristol School of Medical Sciences, Population Health Sciences<br>Prat, Marion; University of Bristol Faculty of Social Sciences and Law, School of Economics<br>Keen, Tim; North Bristol NHS Trust

**Primary Subject Heading**:	Epidemiology
Secondary Subject Heading:	Emergency medicine, Health services research
Keywords:	STATISTICS & RESEARCH METHODS, HEALTH SERVICES ADMINISTRATION & MANAGEMENT, EPIDEMIOLOGY, ACCIDENT & EMERGENCY MEDICINE

I, the Submitting Author has the right to grant and does grant on behalf of all authors of the Work (as defined in the below author licence), an exclusive licence and/or a non-exclusive licence for contributions from authors who are: i) UK Crown employees; ii) where BMJ has agreed a CC-BY licence shall apply, and/or iii) in accordance with the terms applicable for US Federal Government officers or employees acting as part of their official duties; on a worldwide, perpetual, irrevocable, royalty-free basis to BMJ Publishing Group Ltd ("BMJ") its licensees and where the relevant Journal is co-owned by BMJ to the co-owners of the Journal, to publish the Work in this journal and any other BMJ products and to exploit all rights, as set out in our licence.

The Submitting Author accepts and understands that any supply made under these terms is made by BMJ to the Submitting Author unless you are acting as an employee on behalf of your employer or a postgraduate student of an affiliated institution which is paying any applicable article publishing charge ("APC") for Open Access articles. Where the Submitting Author wishes to make the Work available on an Open Access basis (and intends to pay the relevant APC), the terms of reuse of such Open Access shall be governed by a Creative Commons licence – details of these licences and which Creative Commons licence will apply to this Work are set out in our licence referred to above.

Other than as permitted in any relevant BMJ Author's Self Archiving Policies, I confirm this Work has not been accepted for publication elsewhere, is not being considered for publication elsewhere and does not duplicate material already published. I confirm all authors consent to publication of this Work and authorise the granting of this licence.

For peer review only

Can we accurately forecast non-elective bed occupancy and admissions in the NHS? A time-series MSARIMA analysis of longitudinal data from an NHS Trust.

Emily Eyles [1,2*] (emily.eyles@bristol.ac.uk) ; Maria Theresa Redaniel [1,2] (theresa.redaniel@bristol.ac.uk); Tim Jones [1,2] (timothy.jones@bristol.ac.uk); Marion Prat [3] (marion.prat@bristol.ac.uk); Tim Keen [4] (tim.keen@nbt.nhs.uk)

1. The National Institute for Health Research Applied Research Collaboration West (NIHR ARC West) at University Hospitals Bristol and Weston NHS Foundation Trust, UK
2. Population Health Sciences, Bristol Medical School, University of Bristol, UK
3. School of Economics, University of Bristol, UK
4. North Bristol NHS Trust, UK

*corresponding author: 9th Floor, Whitefriars, Lewins Mead, Bristol BS1 2NT. emily.eyles@bristol.ac.uk ; 0117 342 1262

For peer review only

Abstract

Objectives: The main objective of the study was to develop more accurate and precise short-term forecasting models for admissions and bed occupancy for an NHS Trust located in Bristol, England. Subforecasts for the medical and surgical specialties, and for different lengths of stay were realised

Design: Autoregressive integrated moving average models were specified on a training dataset of daily count data, then tested on a six-week forecast horizon. Explanatory variables were included in the models: day of the week, holiday days, lagged temperature and precipitation.

Setting: A secondary care hospital in an NHS Trust in South West England.

Participants: Hospital admissions between September 2016 and March 2020, comprising 1291 days.

Primary and secondary outcome measures: The accuracy of the forecasts was assessed through standard measures, as well as compared to the actual data using accuracy thresholds of 10% and 20% of the mean number of admissions or occupied beds.

Results: The overall ARIMA admissions forecast was compared to the Trust's forecast, and found to be more accurate, namely, being closer to the actual value 95.6% of the time. Furthermore, it was more precise than the Trust's. The subforecasts, as well as those for bed occupancy, tended to be less accurate compared to the overall forecasts. All of the explanatory variables improved the forecasts.

Conclusions: ARIMA models can forecast nonelective admissions in an NHS Trust accurately on a 6-week horizon, which is an improvement on the current predictive modelling in the Trust. These models can be readily applied to other contexts, improving patient flow.

Strengths and limitations of this study:

- The use of ARIMA models, which are simple to set up using *R*, and can be used to improve short term forecasts given enough input data are a strength of this study. Limitations include poorer accuracy in subforecasts, possibly due to using a simpler method.
- The coronavirus pandemic has an impact on the accuracy of forecasts, as shown by the sensitivity analysis
- Data from only one NHS Trust was used, so the models are not validated for other contexts.

Word Count: 4742

Introduction

Hospitals are increasingly busy, and there is more demand for resources such as hospital beds than can be easily met. Resource management is a challenge for many hospitals, and with an increase in the number of unplanned admissions, the issue of efficient resource allocation has become more urgent. The annual growth rate in England of emergency admissions is 3.2% on average over the last ten years [1]. Inaccurate estimates of unplanned admissions, and therefore, unpredictable emergency admissions, can lead to the cancellation of planned or routine operations[2]. This leads to subsequent need for more capacity to cope with routine operation backlog. [3,4]. Granular models with daily and

For peer review only

weekly forecasts predicted accurately can help strategically plan short term and long term resource management, particularly in order to cope with surges in demand.

In the past three and a half years, emergency admissions in the North Bristol NHS Trust (NBT) increased by 26% while average length of stay was reduced from 7.4 days to 6.1 days over the same period[5]. The North Bristol Trust is situated in Bristol and South Gloucestershire, England. The non-elective activity in NBT, particularly emergency admissions and bed occupancy, is higher than in other hospitals in the Bristol, North Somerset, South Gloucestershire Clinical Commissioning Group area. The consequences of this increase in emergency activity are a difficulty in meeting planned care, and, for the Trust, receiving less pay than expected, as unplanned procedures are paid less than planned ones[5]. As the increase in demand is rising faster than demographic growth, the need to efficiently forecast non-elective activity and understand its predictors has been identified. If the Trust is better able to forecast emergency admissions and understand the factors driving their increase, then it will be able to confidently plan over the longer term. Understanding demand allows the Trust to plan what capacity it will need to meet that demand, including the beds required, the workforce and the theatre time. This in turn influences what capacity is then available for elective work.

The NBT used two main methods to forecast non-elective admissions: a scenario-based model, combining the previous year's growth in demand and demographic growth, and a crude average growth model, which only includes demographic growth[5]. These techniques have been found to be insufficient for fine-grained, accurate operational planning, especially at the specialty level[5]. Several techniques have been used to predict unplanned admissions, including multiple linear regression[6-8], generalised estimating equations[9], exponential smoothing[8 10], and the widely-used family of Autoregressive Integrated Moving Average (ARIMA) models[8 11]. Other work has used hybrid models, including forecasting-simulation-optimisation SARIMA and ARIMA models have previously been used to forecast emergency department admissions and occupancy[2 8 10], emergency department crowding (hourly forecasting)[12], and infectious disease bed occupancy[13].

Previous work has mostly used horizons of up to one to two weeks, as forecasting benefit was seen to be limited for longer horizons [2]. Reliable hourly models have been established[12], but the daily horizon over a period of several weeks has been largely underexplored. Shah et al[14] developed a national model of daily midnight bed occupancy using 121 NHS Trusts, across a six week horizon, but this approach may not reflect local context, a priority in the NHS Long Term Plan[15].Our study will explore the six week horizon in a site-specific context. Further, most previous research did not divide by length of stay and specialty, but rather by urgency, or how severe a case is[10 16]. Medicine and Surgery may have different drivers in terms of admission to hospital, and longer lengths of stay tend to have different characteristics. Other work[17 18] has modelled not only beds but nurse and physician availability in A&E, inpatient and outpatient services.

In this paper, we aim to present a method of forecasting admissions and bed occupancy in a local context, using a 45 day (6 week) horizon. We also present subforecasts, divided by length of stay and specialty. The daily horizon over several weeks and the separation by length of stay and specialty are unique to this study, and we further use climatic information to inform the forecast as well. The subforecasts will be assessed using several measures of accuracy, including the mean absolute percentage error, and accuracy thresholds determined by the mean of that particular subforecast. This

For peer review only - http://bmjopen.bmj.com/site/about/guidelines.xhtml

will demonstrate the possibility of conducting more detailed, specific forecasts, which will allow for better, more granular planning.

Methods

NBT provided operational data on non-elective, i.e. unplanned, admissions to Southmead Hospital, the largest of the three hospitals in the Trust, from September 2016 to March 2020. These data are the precursor to what NBT provides to NHS Digital as part of the Hospital Episode Statistics (HES). On average, NBT reported having 868 General and Acute beds available[19]. Figure 1 shows the ratio of monthly elective to non-elective admissions up to February 2020, which was generated from HES data. The data from Hospital Episode Statistics (HES) was obtained under licence (DARS-NIC-17875-X7K1V) from NHS Digital (previously the Health and Social Care Information Centre); Copyright © 2021, re-used with the permission of The Health & Social Care Information Centre. All rights reserved. The data are provided by patients and collected by the NHS as part of their care and support. HES data can be accessed via NHS Digital[20].

The NBT data were split into two: a training dataset of 1246 days to develop the model, and a test dataset of 45 days to test the model. The six week forecast horizon was from the 29 January 2020 to the 14 March 2020. All days had at least one admission or occupied bed, so there were no missing values. The forecast horizon was decided by operational demand. Temperature data were obtained from the Horfield and Filton weather station, and precipitation data were obtained from the Met Office daily values for South West England[21].

Figure 1 goes here

We made forecasts for admissions and bed occupancy (see table 1). Admissions are the start of a hospital spell[22]. Admissions therefore are a count of the number of patients admitted to hospital on a particular day. Bed occupancy was calculated from the admissions data by counting a bed as being occupied by a patient between their admission and discharge dates. Bed occupancy is thus a cumulative measure of how many patients are in hospital on a particular day, even if they were admitted on a previous day. Zero length of stay is defined operationally as a stay of under 24 hours, which does not overlap midnight. Patients who stay over midnight, but for under 24 hours are counted as a non zero length of stay: e.g. if the patient was admitted at 9pm and was discharged at 3am the next day, this would be recorded as a length of stay of one rather than zero. Zero length of stay patients, i.e. patients who did not stay over at least one midnight, were excluded from the analysis[2]. This is because zero length of stay patients are managed through a separate bed-base at NBT. The acuity of these patients means they are rarely admitted into the main bed-base and may not occupy a physical bed (e.g., they may be under observation in a chair for a few hours).

For peer review only - http://bmjopen.bmj.com/site/about/guidelines.xhtml

We controlled for temperature and precipitation (both lagged, the same day last year) the day of the week, holidays (bank, Christmas week, and the Easter holiday weekend). The temperature and precipitation were lagged to the previous year, as weather prediction is not sufficiently accurate for longer forecast horizons. Weather data have been included in other forecasting models and contexts, to mixed results[10 16 23 24].

Day of the week, as well as holidays, inclusive of bank holidays, were found across several studies to be highly relevant in predicting secondary care admissions[23-26]. Finally, a simple trend variable was generated, to account for the upward direction of admissions and bed occupancy over time, calculated as the days since the start of the dataset [27]. Table 1 details the variables included in the models. Separate subforecasts were also conducted divided by specialty (Surgery or Medicine) and length of stay (over or under 48 hours), detailed in table 2. The threshold of 48 hours was chosen as it is the acute medicine phase[28]. In the UK, as a part of good clinical care leading to successful outcomes, many patients can and should be diagnosed, treated, and even discharged within the 48 hour timeline[28]. Specialty was determined by a classification of Treatment Function Codes[29] provided by the Trust.

Table 1: Outcome variables and explanatory variables

**Variable**	**Definition**
**Outcomes**	
Admissions	Count of admissions to hospital, for a non-zero length of stay, i.e. overnight stays
Bed Occupancy	Count of hospital beds occupied, calculated as a count between admission and discharge dates, for non-zero lengths of stay
**Explanatory variables**	
Day of the week	Sunday, Monday, Tuesday, Wednesday, Thursday, Friday, Saturday
Temperature last year	The average temperature in Celsius on the same day last year
Precipitation last year	The precipitation in millimetres on the same day last year
Holiday	Whether a day was a bank holiday, over Christmas week, or during the Easter holiday weekend

Table 2: Model specifications

For peer review only - http://bmjopen.bmj.com/site/about/guidelines.xhtml

Outcome	Treatment Specialty	Length of Stay
**Admissions**	All	All
		<= 48 hours
		> 48 hours
	Medicine	All
		<= 48 hours
		> 48 hours
	Surgery	All
		<= 48 hours
		> 48 hours
**Bed Occupancy**	All	All
		<= 48 hours
		> 48 hours
	Medicine	All
		<= 48 hours
		> 48 hours
	Surgery	All
		<= 48 hours
		> 48 hours

We used Multivariate Seasonal AutoRegressive Integrated Moving Average (MSARIMA) models to forecast emergency admissions and bed occupancy, following the ARIMA forecasting procedure detailed by Jebb et al[27]. MSARIMA models allow for seasonality, or regularly repeating cycles or trends in the data, and takes into account the autoregressive order p, d is the differencing order, and q is the moving average order. The second set of brackets represents the seasonal versions of these components[9]. The autoregressive part of the model regresses the forecast variable against its own past values, to the order of the number of time lags (p)[30]. The moving average part of the model uses the past regression errors terms to the order of the time lag of the error (q)[30]. The differencing part of the model (d) refers to how many times lagged values have been subtracted from the data [30].

The modelling component consists first of an examination of the time series to check whether it is stationary, ie whether its mean, variance and autocorrelation do not vary over time, a necessary requirement if we want to use information about the past behaviour of the series to learn something about its future values. Unlike other time series modelling techniques, the ARIMA family of models allow for nonstationary data and the inclusion of explanatory variables[27]. Augmented Dickey-Fuller tests indicated that all data series were stationary or integrated of order 0 (d=0). MSARIMA models are a special type of regression for time series, which use information from previous days to inform them, while adjusting for other factors in the model, such as the weather, or day of the week[10]. MSARIMA modelling techniques were chosen as they are one of the recommended advanced forecasting techniques by NHS England[22], due to the complexity and seasonality of A&E settings. Further, according to NHS England[22], these techniques provided the most consistent estimate of daily A&E patient volumes; other methods such as artificial neural network models were said to provide less accurate forecasts of these volumes.

For peer review only - http://bmjopen.bmj.com/site/about/guidelines.xhtml

The mean absolute percentage error (MAPE) and the root mean square error (RMSE) are presented for each model [2 10-13 16 30]. MAPE measures model fit with closer to 0% indicating better fit[16]. For the RMSE, lower values indicate better model performance, though its value is dependent on the mean value of the variable, therefore comparisons of this metric between models with widely varying means is hindered[11] . The subforecast means are presented in table 3 along with the standard deviations; more detailed results can be found in Appendix 1. The model was tested against the test dataset, providing the actual out-of-sample accuracy. The model accuracy was determined by whether the residual of the forecast vs the observed was over or under a given threshold. We used 2 thresholds, representing moderate and strict accuracy. This was defined as 20% of the sample mean for moderate accurate and 10% of the sample mean for strict accuracy. These thresholds match the ad-hoc thresholds determined in meetings with NBT, namely a 10/20 bed threshold for all admissions, which is matched by a 9.4/18.8 bed threshold as mean percentages. Accuracy is thus represented as a percentage of accurate daily forecasts within those thresholds (see table 3). A short-term 6 week (45 day) forecast horizon was chosen, as long term daily forecasts require different methods, as eventually the autoregressive component of the model will converge to the mean [30].

The data were prepared using Stata 15.1, and analysed using the *forecast* package in R. Following the data checks, the *auto.arima* function in R was used to determine the model specifications[31]. To determine the relevance of the explanatory variables, once the model specifications were determined, the models were specified with and without the explanatory variables. Models with the explanatory variable of interest were compared to null models and models containing the other explanatory variables on the basis of AIC, similar to how *auto.arima* determines model specifications[31]. Admissions and bed occupancy were modelled separately. The first two models were all admissions and all bed occupancy data. Then, subforecasts were modelled, split by specialty or length of stay, and finally specialty and length of stay (see table 2). The code for the models is provided in Appendix 2.

Sensitivity analyses were performed for two other time periods on the overall admissions dataset. The first was using the data from September 2016 to mid December 2019, for a horizon of six weeks between the 1 November 2019 and the 15 December 2019. The second sensitivity analysis used the data from September 2016 to mid June 2020, for a horizon between the 2 May 2020 and the 15 June 2020. These horizons were selected to either omit the impact of the coronavirus pandemic (the earlier horizon) or to examine the forecast within the coronavirus pandemic (the later horizon). A further sensitivity analysis was performed by excluding the climatic data from the models (see Appendix 3).

Patient and public involvement statement: Members of the public were consulted in the development of this study. The research idea was presented to them in a workshop and suggestions and comments were incorporated in the protocol. Feedback during the workshop was positive, with participants agreeing with the research objectives and the identified need for forecasting models. The use of generic and generalisable models were highlighted in the discussion, and that the audience and end users perspectives should be considered in the development of the model and tool.

Results

18 separate ARIMA models were forecast for the variable specifications described in Table 1, and the subsets described in table 2. The final ARIMA specification, accuracy, RMSE, and MAPE of the models can be seen in table 3. Day of the week, holiday days, temperature, and precipitation were all found to improve the accuracy of the forecasts and subforecasts. The model AICs and the AICs of

For peer review only - http://bmjopen.bmj.com/site/about/guidelines.xhtml

comparable models without temperature and precipitation can be found in Appendix 3. The subsets' mean and standard deviation are also provided. Models with more training data, i.e. higher sample size, were more accurate.

The overall admissions model was accurate at the moderate threshold 88.9% of the time, and at the strict threshold 60.0% of the time. Bar the surgical and under 48 hour length of stay subforecasts, 82.2% of daily predictions of admissions were within the moderate admission threshold; 51.1% were within the strict accuracy admission threshold. Models for surgical admissions performed less well, with the under 48 hour length of stay surgical admissions having accuracy of 31.1% on the moderate level, and 11.1% on the strict level. This is likely due to sample size, and high variation in daily admissions.

For bed occupancy, the models generally performed more poorly. For the moderate bed threshold, all models were at least 28.9% accurate, and for the strict bed threshold, the lowest accuracy was 15.6%. However, this, similar to admissions, was dependent on specialty and length of stay. The bed occupancy model overall (model 10) performed rather poorly, and was accurate within the moderate threshold77.8% of the time, and within the strict threshold 40.0 % of the time. Surgery performed better as a specialty, and under 48 hour length of stay tended to outperform over 48 hour length of stay as well. Appendix 1 shows the subforecasts for each model in table 3, with 95% confidence intervals.

Table 3: Model specifications and accuracy

Model	Outcome	(Sub)for-ecast	SARIMA (p,d,q)(P,D,Q)	Model accuracy (strict/moderate thresholds)	RMSE	MAPE	Mean of Outcome	SD of outcome
1	Admissions	All data overall	(3,0,0) (1,0,0)	60.0%/88.9 %	9.59	8.66	94.04	14.98
2	Admissions	All medical	(3,0,2) (2,0,2)	51.1%/84.4 %	8.98	9.44	79.14	19.32
3	Admissions	All surgical	(1,0,2) (0,0,0)	28.9%/57.8 %	4.59	19.86	20.65	5.31
4	Admissions	All under 48 hour length of stay	(1,0,0) (1,0,0)	28.9%/71.1 %	6.81	16.53	35.38	9.06
5	Admissions	All over 48 hour length of stay	(3,0,1) (1,0,1)	62.2%/88.9 %	7.22	10.78	55.14	10.04
6	Admissions	Under 48 hour length of stay, medical	(4,0,0) (2,0,0)	24.4%/48.9 %	6.45	18.64	30.59	10.21
7	Admissions	Under 48 hour	(0,0,1) (0,0,0)	11.1%/31.1 %	7.82	44.19	20.99	8.39

For peer review only - http://bmjopen.bmj.com/site/about/guidelines.xhtml

		length of stay, surgical						
8	Admissions	Over 48 hour length of stay, medical	(5,0,1) (2,0,0)	53.3%/82.2 %	6.88	11.97	48.55	12.06
9	Admissions	Over 48 hour length of stay, surgical	(3,0,3) (1,0,2)	25.8%/46.7 %	3.40	27.45	11.87	3.74
10	Bed Occupancy	All data overall	(0,0,1) (1,0,0)	40.0%/77.8 %	158.24	15.57	823.36	165.22
11	Bed Occupancy	All medical	(4,0,0) (2,0,0)	37.8%/60.0 %	151.69	17.06	760.03	189.22
12	Bed Occupancy	All surgical	(0,0,1) (0,0,0)	24.4%/37.8 %	64.50	35.40	162.62	67.71
13	Bed Occupancy	All under 48 hour length of stay	(3,0,0) (2,0,1)	28.9%/66.7 %	15.72	16.41	82.3	21.06
14	Bed Occupancy	All over 48 hour length of stay	(0,0,1) (1,0,0)	40.0%/75.6 %	159.55	17.6	767.41	177.2
15	Bed Occupancy	Under 48 hour length of stay, medical	(4,0,0) (2,0,0)	22.2%/48.9 %	14.95	18.63	70.83	23.95
16	Bed Occupancy	Under 48 hour length of stay, surgical	(0,0,1) (0,0,0)	15.6%/28.9 %	7.82	44.19	21	8.38
17	Bed Occupancy	Over 48 hour length of stay, medical	(2,0,1) (0,0,0)	35.6%/57.8 %	152.7	19.15	673.24	181.24
18	Bed Occupancy	Over 48 hour length	(0,0,1) (0,0,0)	20.0%/35.6 %	64.95	44.22	141.63	67.49

For peer review only - http://bmjopen.bmj.com/site/about/guidelines.xhtml

		of stay, surgical						

Our forecasts were fairly close to the actual values observed in the test data. Our prediction for admissions overall, for example, was within the moderate threshold (18.8), excepting 5 days on our 45-day horizon. 15 days were between the moderate and strict (9.4) thresholds. 25 days were under the strict accuracy threshold. The model was less accurate the farther forward in time from the beginning of the horizon. While the forecast sometimes underestimates the number of admissions, the forecasting models are reviewed by the clinical site team, and adjustments are made to the predictor based on the particular day. Further, possibly due to the impact of the coronavirus pandemic, the latter part of the horizon may have different patterning with respect to hospital admissions. This can be seen in figure 2 (Model 1 predictions), with the actual value of admissions dropping at the end of the horizon. The 95% confidence intervals range from about 67 to 135 beds wide.

Figure 2 goes here

Compared to NBT's in-house prediction modelling for overall admissions, the ARIMA model outperformed them in terms of accuracy apart from two days, one which the NBT model was closer by just 1.5 beds, and another where the NBT model was closer by 10 beds. The ARIMA forecast is closer in value to the true admissions count 95.6% of the time. Figure 3 shows the forecast for overall admissions and its 95% confidence interval, compared to the actual value and NBT model. The mean difference between the ARIMA models and the actual admissions value was 7.3 beds, with a standard deviation of 9.9 beds. The median difference for the ARIMA models was 6 beds. For the NBT prediction models, the mean difference from the actual value was 21 beds, with a standard deviation of 11.1 beds, and a median difference of 21 beds. Finally, the correlation between the ARIMA prediction and the actual values was 0.75, whereas for the NBT prediction, it was -0.45. Therefore, the ARIMA models are more accurate and more precise than the NBT models.

Figure 3 goes here

The sensitivity analyses for the other horizons (see Appendix 4) on the overall admissions data demonstrated that the modelling strategy was more accurate prior to the coronavirus pandemic. Compared to the horizon presented earlier, the December 2019 horizon predicted within a strict accuracy threshold (10% of the sample mean) 75.6% of the time compared to 60.0% for the study horizon, and within the moderate accuracy threshold (20% of the sample mean) 93.3% of the time, compared to 88.9% in the study horizon. The June 2020 forecast horizon performed worse than both study horizon and other sensitivity analysis. It only forecasted 42.2% of the time within the strict threshold, and 77.8% of the time within the moderate threshold. The coronavirus pandemic likely has an impact on forecasting models, so consideration should be taken when using data from the pandemic period.

Discussion

We have extended the literature by producing forecasts for a variety of combinations of specialty and length of stay, for both emergency admissions and bed occupancy, as well as for nonelective admissions beyond those for trauma[23 25 32]. We have found that these models work very well for some but not as well for other types of subsets, specifically shorter lengths of stay and for surgery.

For peer review only - http://bmjopen.bmj.com/site/about/guidelines.xhtml

The models appear to work better in general for admissions rather than bed occupancy, as well as for shorter lengths of stay. The differences in accuracy can possibly be explained by the beginning of the coronavirus pandemic in the United Kingdom, as can be seen in the test data for several of the subsets, particularly for over 48 hour length of stay, and bed occupancy: a sharp drop can be seen at the beginning of February 2020. In the all data categories, and under 48 hour length of stay this sharp rise and fall can be seen around mid February 2020 (see appendix 1). This can be interpreted as a limitation to the models' usefulness while COVID-19 has an impact on hospital admissions. Indeed, the sensitivity analyses showed that the pandemic had an impact on the accuracy of the models. Back to recovery, similarly to the pre-pandemic sensitivity analysis, the model might be more applicable although it might need more refinements to take into account changes brought about by the pandemic.

Other limitations of this study include the poorer accuracy in subforecasts, which may require more complex methods to mitigate, and the use of only one NHS Trust context. Using only one Trust means that the models are not necessarily validated for different contexts and settings. Work by Shah et al[14] has done similar modelling techniques at the national level, with accurate results. However, models should be site-specific to maximise utility: this means that it is necessary to test them in other Trusts. Further, Ordu et al[8] emphasise the importance of testing different modelling techniques for varying specialties, services, and patients. More complex modelling strategies could prove to be more accurate, like the combination of different modelling techniques, such as hybrid forecasting-simulation-optimisation models[17 18], which can be used for not only A&E but inpatient and outpatient services as well.

The strengths of this study are the use of ARIMA models, which are simple to set up using *R*, and can be used to improve short term forecasts given enough input data. The dataset used is the hospital level precursor to the Hospital Episode Statistics (HES), available through *NHS Digital.* It is possible to improve short-term models from NBT with a relatively simple procedure, which should be transferable to other contexts, due to this similarity. The models can be incorporated easily into practice by sharing the *R* code on Github or a similar platform, and tested on data from other Trusts, which should follow a standard structure, again due to the reporting requirements of HES. The code used to generate the models in this paper is available, with commentary, as a supplementary file (see appendix 2).

Abraham and colleagues[2] found that admissions for patients spending at least one night were predominantly random and difficult to predict, using 3 years worth of training data. However, we found that, in general, over all subsets admissions forecasts specified better than bed occupancy ones, and that forecast horizons of over a week can be produced. It would appear that bed occupancy data are more volatile than admissions. Bed occupancy may be harder to forecast, in that it is driven by admissions, and then length of stay is at times difficult to predict[33]. It is likely that, for subsets of bed occupancy, due to the smaller amount of observations (for example, under 48 hour surgical bed occupancy has a mean of 21.00 compared to 849.71 on the whole), and higher variability of the subsets, that the models for bed occupancy produce less accurate forecasts with higher MAPEs. The MAPE for daily admissions found by Boyle and colleagues is 11%: several of the subforecasts, such as the medicine, surgery, and over 48 hour length of stay ones reach this threshold, but as the amount of observations decreases, the inaccuracy seems to increase. A possible avenue of improvement would be to forecast bed occupancy by levels of severity.

For peer review only - http://bmjopen.bmj.com/site/about/guidelines.xhtml

Day of the week has been shown to have reasonably strong predictive capacity in previous work[23 24 26], though the inclusion of climate variables is debated, with no clear answer[9 10 16 24-26 32 34]. Calegari and colleagues found that SARIMA models including day of the week, but not climatic variables were the most accurate, especially in complex departments. However, the test period did not comprise the whole year, and the study was located in one hospital in southern Brazil, a different climate to Bristol, England[10]. Similarly, in Brazil, the ambient temperature effect was not found to improve daily visit forecasts, though again, the data only were for one season. Sun et al[16] also found that admissions were not predicted by weather, though caution that Singapore, the study setting, has little variation in its weather throughout the year. Atherton et al[25] found that adult trauma admissions were not influenced by the weather, but only used temperature in 5 Celsius increments over a single year, whereas this study uses temperature in Celsius to a single decimal point over a longer time period. In contrast, Macgregor[34] found an association between daily temperature and trauma admissions in Aberdeen, and Rising et al[32] found similar results for temperature and precipitation on trauma admissions in an American hospital. Note that these solely examine trauma admissions, rather than other types of nonelective secondary admissions, which our study includes. Finally, Sahu et al[26] developed a Bayesian model for forecasting hospital admissions in Cardiff and Southampton, two nearby localities to Bristol, the setting of this study, and emphasised the importance and relevance of including weather data. Therefore context is important, as in climatic data marginally improved our models, similar to other UK studies, and we suggest its inclusion be considered in future work.

By improving short term forecasts, care can be better planned, limiting cancellations due to capacity or patient flow issues. Better care can be provided as hospitals will be less crowded. In future, other methods more appropriate for longer horizons can be tested, and medium to long term forecasts can be undertaken with more appropriate methods. We have developed ARIMA models that can forecast emergency admissions over a six week horizon with very good accuracy, which are more precise and more accurate than the current models used by the NBT. More work is needed to inform further model development, as the accuracy of subforecasts for length of stay and specialty varies significantly. This is important work because improved modelling capability will have a direct impact on the business planning of acute NHS Trusts. Improving patient flow through the hospitals greatly improves the working and care environment, reduces A&E crowding and enables hospitals to provide the most appropriate care for patients.

Acknowledgements

This research was supported by the National Institute for Health Research (NIHR) Applied Research Collaboration West (NIHR ARC West). The views expressed in this article are those of the author(s) and not necessarily those of the NIHR or the Department of Health and Social Care. The authors are grateful to the members of the public who contributed through the patient and public involvement process.

Author Statement: EE drafted the manuscript. EE and TJ analysed the data. MTR and TK supervised the project. TJ managed the data. MP conceptualised the methodology and conducted preliminary analysis of the data. MTR is the guarantor of this paper. The public contributed through the patient and public involvement process in the development of the study.

Copyright: I, the Submitting Author has the right to grant and does grant on behalf of all authors of the Work (as defined in the author licence), an exclusive licence and/or a non-exclusive licence for contributions from authors who are: i) UK Crown employees; ii) where BMJ has agreed a CC-BY licence

For peer review only - http://bmjopen.bmj.com/site/about/guidelines.xhtml

shall apply, and/or iii) in accordance with the terms applicable for US Federal Government officers or employees acting as part of their official duties; on a worldwide, perpetual, irrevocable, royalty-free basis to BMJ Publishing Group Ltd ("BMJ") its licensees.

The Submitting Author accepts and understands that any supply made under these terms is made by BMJ to the Submitting Author unless you are acting as an employee on behalf of your employer or a postgraduate student of an affiliated institution which is paying any applicable article publishing charge ("APC") for Open Access articles. Where the Submitting Author wishes to make the Work available on an Open Access basis (and intends to pay the relevant APC), the terms of reuse of such Open Access shall be governed by a Creative Commons licence – details of these licences and which licence will apply to this Work are set out in our licence referred to above.

Competing interest statement: All authors have completed the ICMJE uniform disclosure form at www.icmje.org/coi_disclosure.pdf and declare: support from HDRUK Better Care Partnership and NIHR ARC West for the submitted work; no financial relationships with any organisations that might have an interest in the submitted work in the previous three years; no other relationships or activities that could appear to have influenced the submitted work.

Transparency declaration: The lead author affirms that this manuscript is an honest, accurate, and transparent account of the study being reported; that no important aspects of the study have been omitted; and that any discrepancies from the study as planned (and, if relevant, registered) have been explained.

Ethical approval: University of Bristol Faculty Research Ethics Approval was obtained. (ID #109,024)

Funding statement: This study was funded by the HDRUK Better Care Partnership (#6.12). This research was supported by the National Institute for Health Research (NIHR) Applied Research Collaboration West (NIHR ARC West). The views expressed in this article are those of the author(s) and not necessarily those of the NIHR or the Department of Health and Social Care.

Role of sponsor/ Statement of independence of researchers from funders: The funding source and sponsors had no role in the design of this study and will not have any role during its execution, analyses, interpretation of the data, or decision to submit results.

Dissemination declaration: The results of the study are to be shared to the North Bristol NHS Trust.

Data sharing statement: The data used in the study is collected by the NBT as part of their care and support. Sharing of anonymised data with the University of Bristol was underpinned by a data sharing agreement and solely covers the purposes of this study. Data requests can be made through the NBT. Statistical code is available as a supplementary file.

Figure legends:

Figure 1: Monthly ratio of elective to non-elective admissions, September 2016 to February 2020

Figure 2: ARIMA forecast for overall admissions to NBT

Figure 3: Forecast predictions for overall admissions (model 1) compared to the actual values and the NBT model

References

For peer review only - http://bmjopen.bmj.com/site/about/guidelines.xhtml

1. Steventon A, Deeny S, Friebel R, et al. Briefing: Emergency hospital admissions in England: which may be avoidable and how? London: The Health Foundation, 2018.
2. Abraham G, Byrnes GB, Bain CA. Short-Term Forecasting of Emergency Inpatient Flow. *IEEE Transactions on Information Technology in Biomedicine* 2009;13(3):380-88.
3. Association BM. NHS pressures – winter analysis. London: British Medical Association, 2018.
4. Office NA. Report by the Comptroller and Auditor General: Reducing emergency admissions. London: National Audit Office, 2018.
5. Falconer C, Keen T. Forecasting and demand in the North Bristol Trust, 2018.
6. Bilings J, Georghiou T, Blunt I, et al. Choosing a model to predict hospital admission: an observational study of new variants of predictive models for case finding. *BMJ Open* 2013;3:e003352.
7. Boyle J, Wallis M, Jessup M, et al. Regression Forecasting of Patient Admission Data. *30th Annual International Conference of the IEEE Engineering in Medicine and Biology Society* 2008:3819-22.
8. Ordu M, Demir E, Tofallis C. A comprehensive modelling framework to forecast the demand for all hospital services. *The International Journal of Health Planning and Management* 2019;34(2):e1257-e71.
9. Marcilio I, Hajat S, Gouveia N. Forecasting Daily Emergency Department Visits Using Calendar Variables and Ambient Temperature Readings. *Academic Emergency Medicine* 2013;20(8):769-77.
10. Calegari R, Fogliatto FS, Lucini FR, et al. Forecasting Daily Volume and Acuity of Patients in the Emergency Department. *Computational and Mathematical Methods in Medicine* 2016;2016:1-8.
11. Wargon M, Guidet B, Hoang TD, et al. A systematic review of models for forecasting the number of emergency department visits. *Emerg Med J* 2009;26:395-99.
12. Schweigler LM, Desmond JS, McCarthy ML, et al. Forecasting Models of Emergency Department Crowding. *Academic Emergency Medicine* 2009;16(4):301-08.
13. Earnest A, Chen MI, Ng D, et al. Using autoregressive integrated moving average (ARIMA) models to predict and monitor the number of beds occupied during a SARS outbreak in a tertiary hospital in Singapore. *BMC Health Services Research* 2005;5:36.
14. Shah K, Sharma A, Moulton C, et al. Forecasting the Requirement for Nonelective Hospital Beds in the National Health Service of the United Kingdom: Model Development Study. *JMIR Med Inform* 2021;9:e21990.
15. England N. The NHS Long Term Plan. London: NHS, 2019.
16. Sun Y, Heng BH, Seow YT, et al. Forecasting daily attendances at an emergency department to aid resource planning. *BMC Emergency Medicine* 2009;9:1.
17. Ordu M, Demir E, Davari S. A hybrid analytical model for an entire hospital resource optimisation. *Soft Computing* 2021;25:11673-90.
18. Ordu M, Demir E, Tofallis C, et al. A novel healthcare resource allocation decision support tool: A forecasting-simulation-optimization approach. *Journal of the Operational Research Society* 2021;72(3):484-500.
19. Digital N. Bed availability and occupancy data - overnight London: NHS; 2021 [Available from: https://www.england.nhs.uk/statistics/statistical-work-areas/bed-availability-and-occupancy/bed-data-overnight/ accessed 28/01 2022.
20. Digital N. Data Access Request Service London: NHS; 2022 [Available from: https://digital.nhs.uk/services/data-access-request-service-dars
21. Office M. Daily Southwest England & Wales Precipitation (mm) In: Office M, ed. London, 2020.
22. England N, Improvement N. Advanced forecasting techniques: How to use advanced forecasting techniques for estimating demand of NHS Services. London: NHS, 2020.

For peer review only - http://bmjopen.bmj.com/site/about/guidelines.xhtml

23. Bhattacharyya T, Millham F. Relationship between Weather and Seasonal Factors and Trauma Admission Volume at a Level I Trauma Center. *The Journal of Trauma and Acute Care Surgery* 2001;51(1):118-22.
24. Holleman Jr DR, Bowling RL, Gathy C. Predicting daily visits to a walk-in clinic and emergency department using calendar and weather data. *J Gen Intern Med* 1996;11(4):237-39.
25. Atherton W, Harper W, Abrams K. A year's trauma admissions and the effect of the weather. *Injury* 2005;36(1):40-46.
26. Sahu S, Baffour B, Harper P, et al. A hierarchical Bayesian model for improving short-term forecasting of hospital demand by including meteorological information. *Journal of the Royal Statistical Society Series A (Statistics in Society)* 2014;177(1):39-61.
27. Jebb A, Tay L, Wang W, et al. Time series analysis for psychological research: Examining and forecasting change. *Frontiers in Psychology* 2015;6:727.
28. Leman P. Emergency care in the first 48 hours. *BMJ* 2007;334(7587):218-19.
29. NHS. Treatment Function Code 2021 [updated 25/11/2021. Available from: https://datadictionary.nhs.uk/attributes/treatment_function_code.html accessed 15/01 2022.
30. Hyndman RJ, Athanasopoulos G. Forecasting: principles and practice. Melbourne: OTexts, 2018.
31. Hyndman RJ. auto.arima 2020 [Available from: https://www.rdocumentation.org/packages/forecast/versions/8.12/topics/auto.arima accessed 20/08/2020 2020.
32. Rising W, O'Daniel J, Roberts C. Correlating Weather and Trauma Admissions at a Level I Trauma Center. *The Journal of Trauma and Acute Care Surgery* 2006;60(5):1096-100.
33. Predicting Hospital Length of Stay (PHLOS): A Multi-tiered Data Mining Approach. 2012 IEEE 12th International Conference on Data Mining Workshops; 2012 10-10 Dec. 2012.
34. Macgregor D. Effect of weather on attendance with injury at a paediatric emergency department. *Emerg Med J* 2003;20:204-05.

For peer review only - http://bmjopen.bmj.com/site/about/guidelines.xhtml

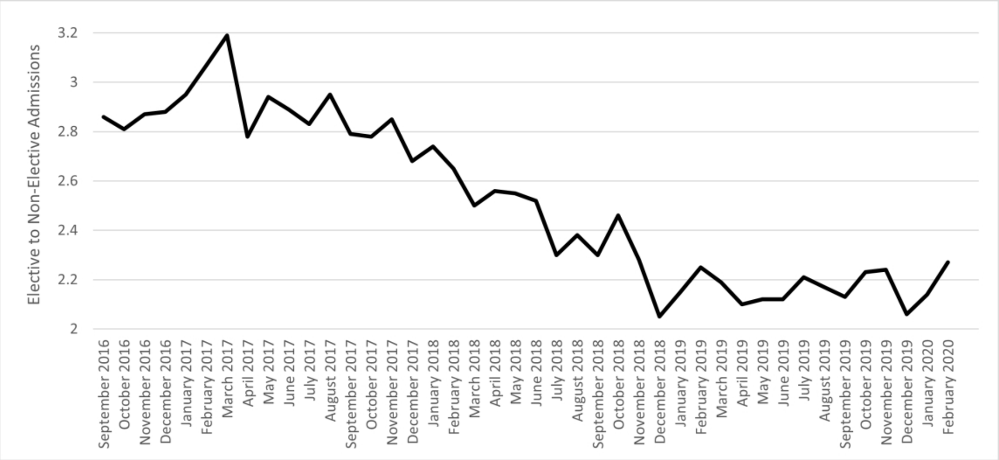

Figure 1: Monthly ratio of elective to non-elective admissions, September 2016 to February 2020

497x228mm (130 x 130 DPI)

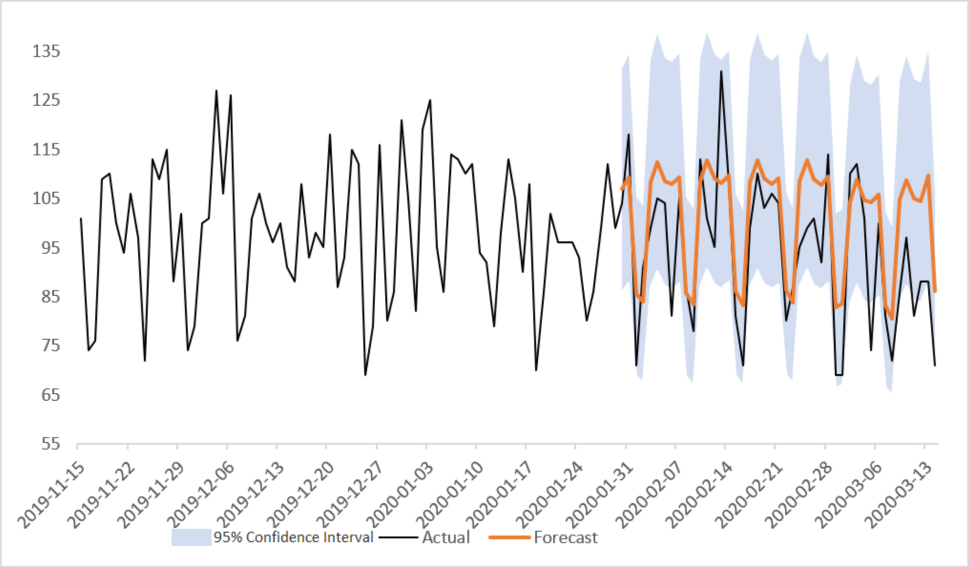

Figure 2: ARIMA forecast for overall admissions to NBT

221x129mm (120 x 120 DPI)

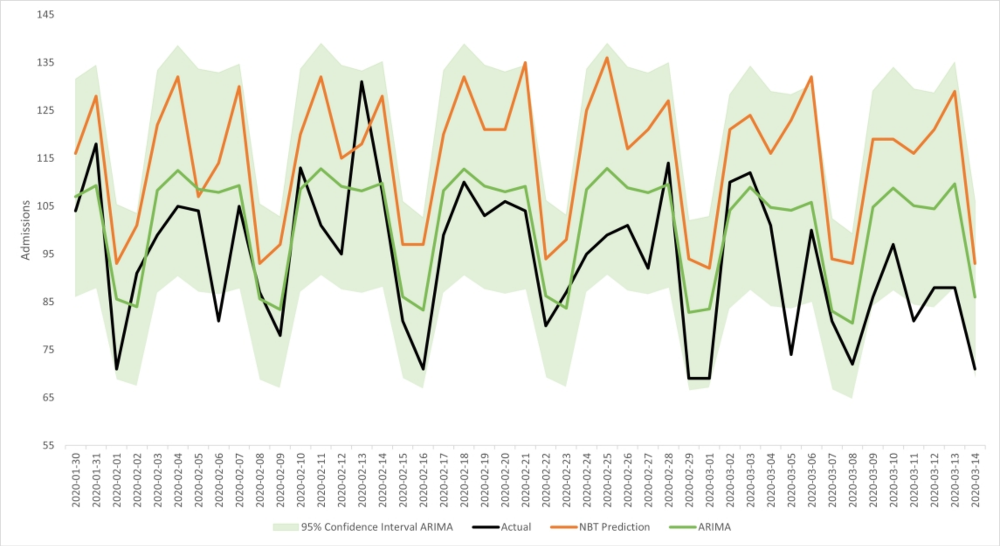

Figure 3: Forecast predictions for overall admissions (model 1) compared to the actual values and the NBT model

685x375mm (130 x 130 DPI)

Appendix 1: Subforecast figures

Figure A1: Model 2 (medicine admissions)

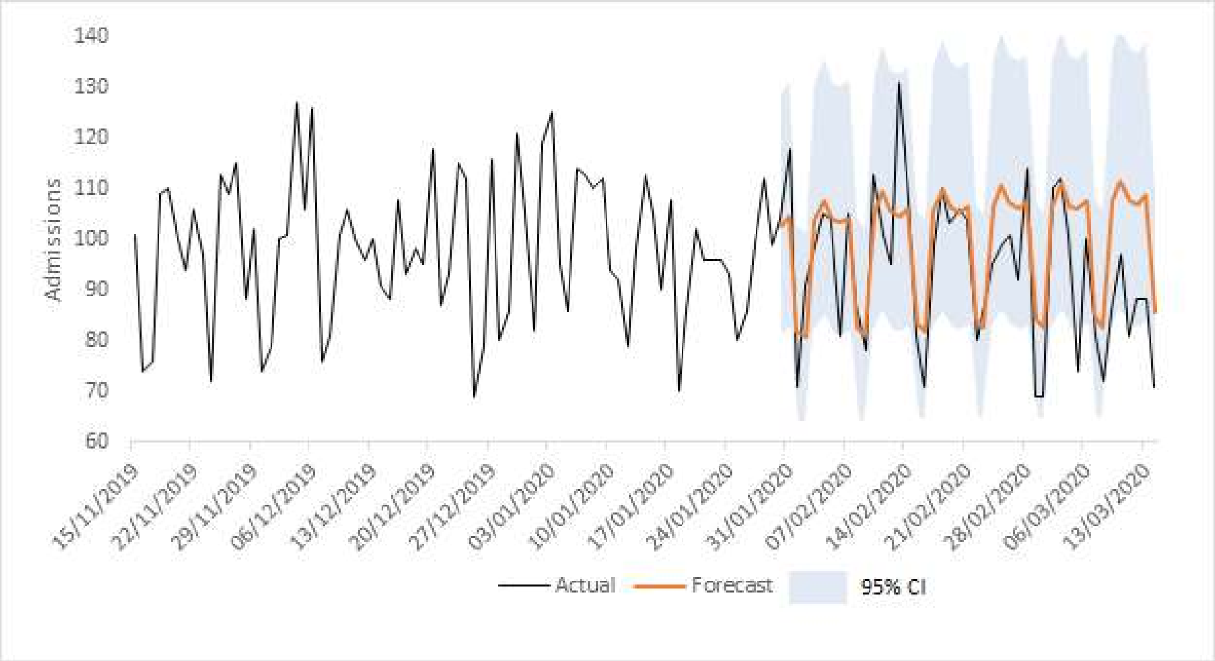

Figure A2: Model 3 (surgery admissions)

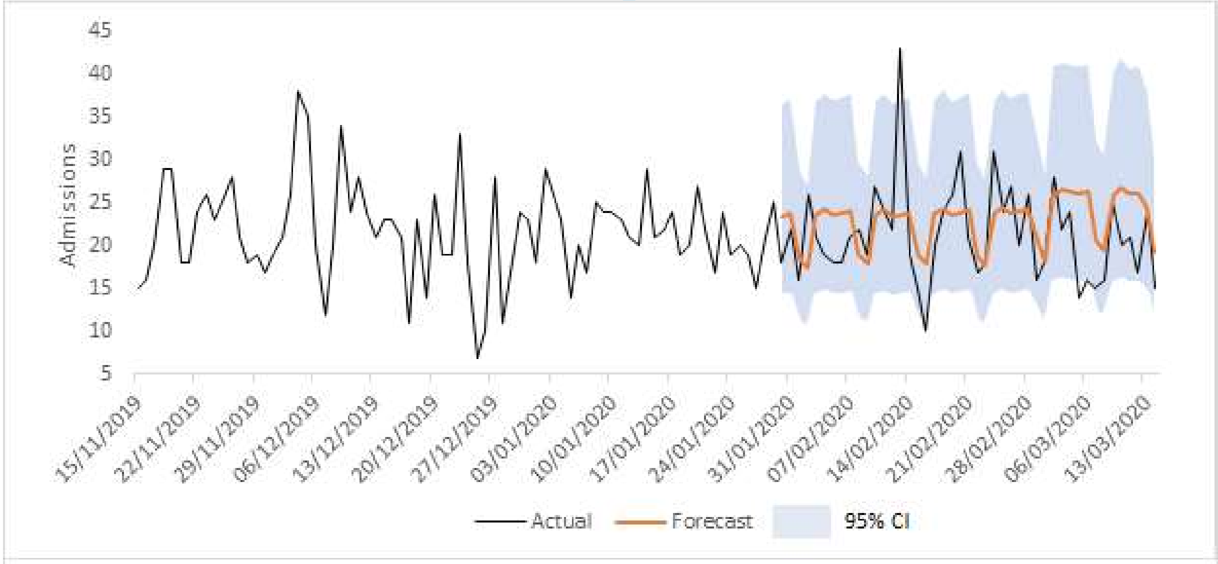

Figure A3: Model 4 (under 48 hour length of stay admissions)

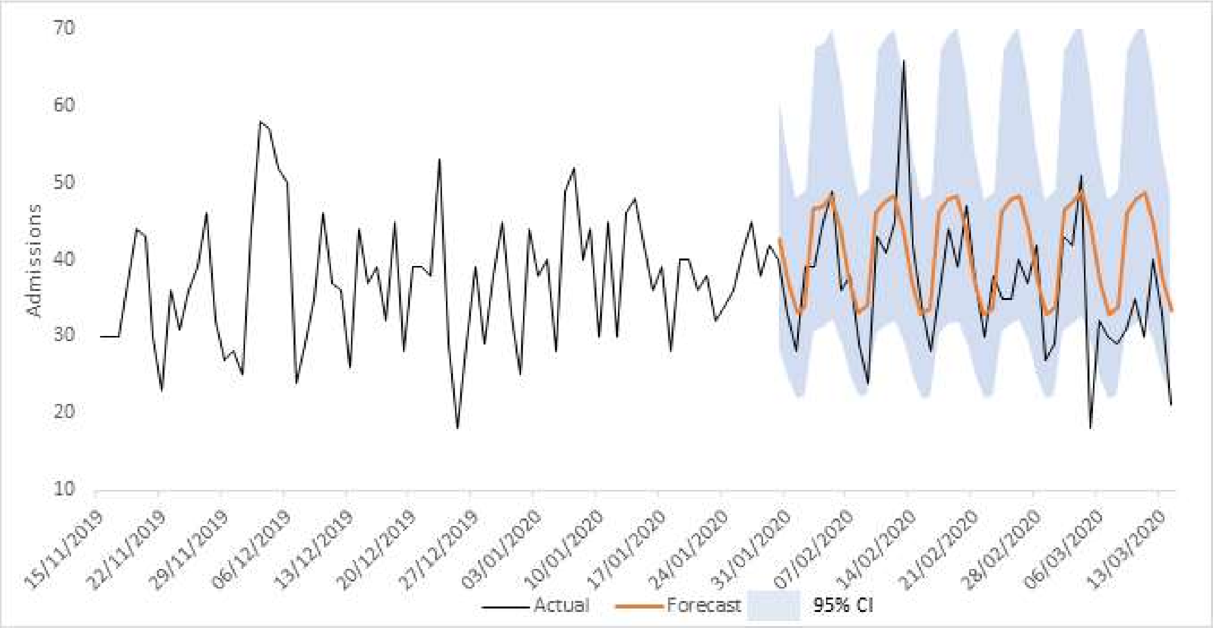

Figure A4: Model 5 (over 48 hour length of stay admissions)

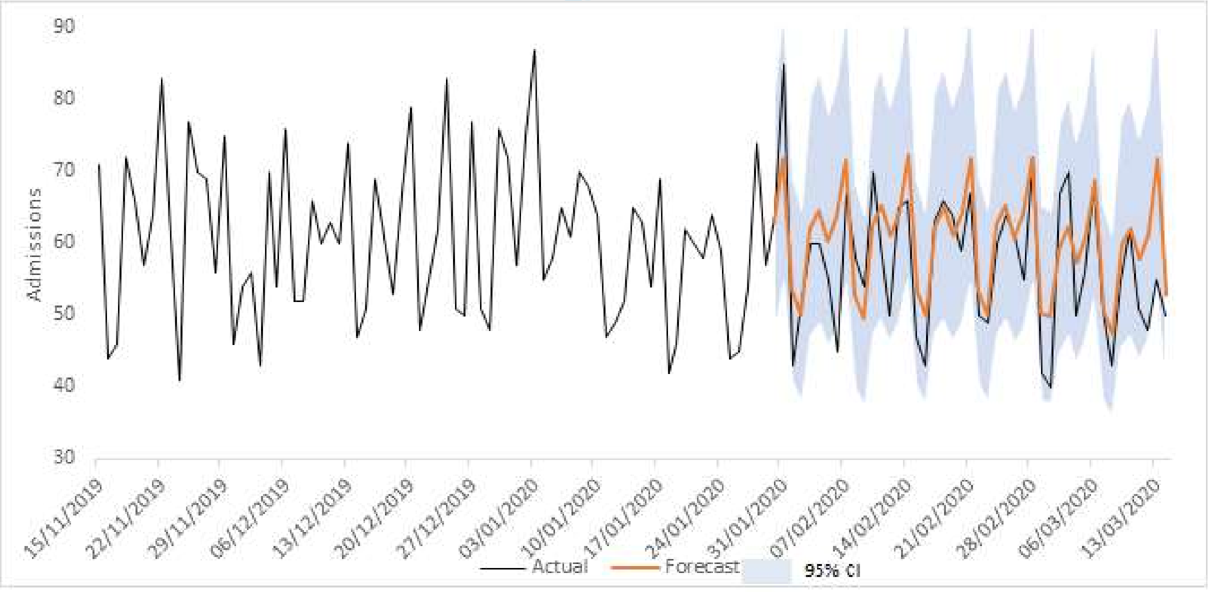

Figure A5: Model 6 (under 48 hour length of stay medicine admissions)

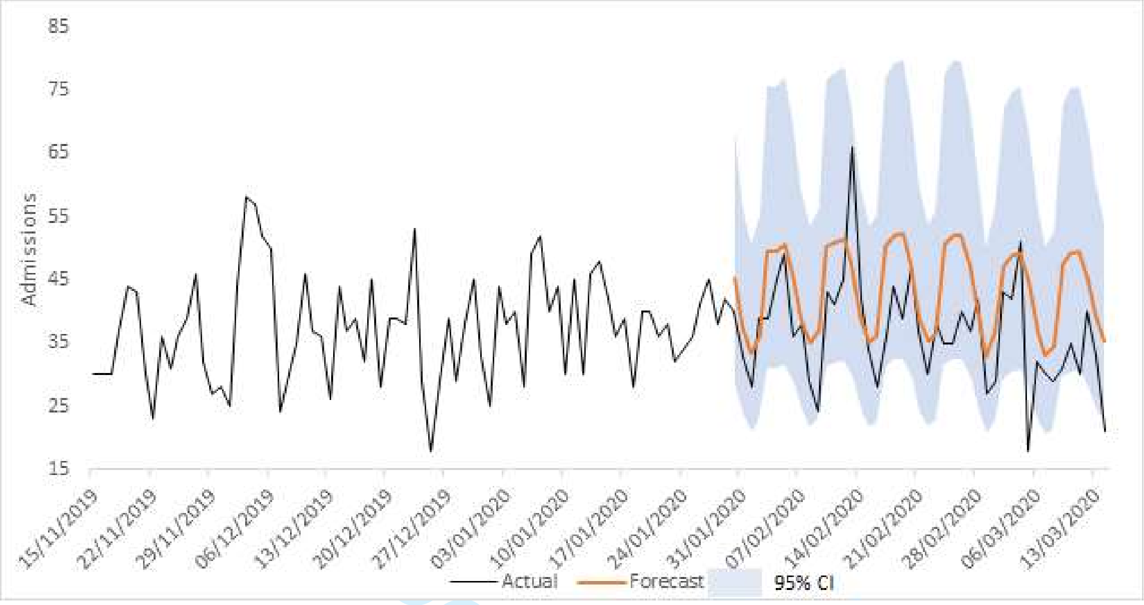

Figure A6: Model 7 (under 48 hour length of stay surgery admissions)

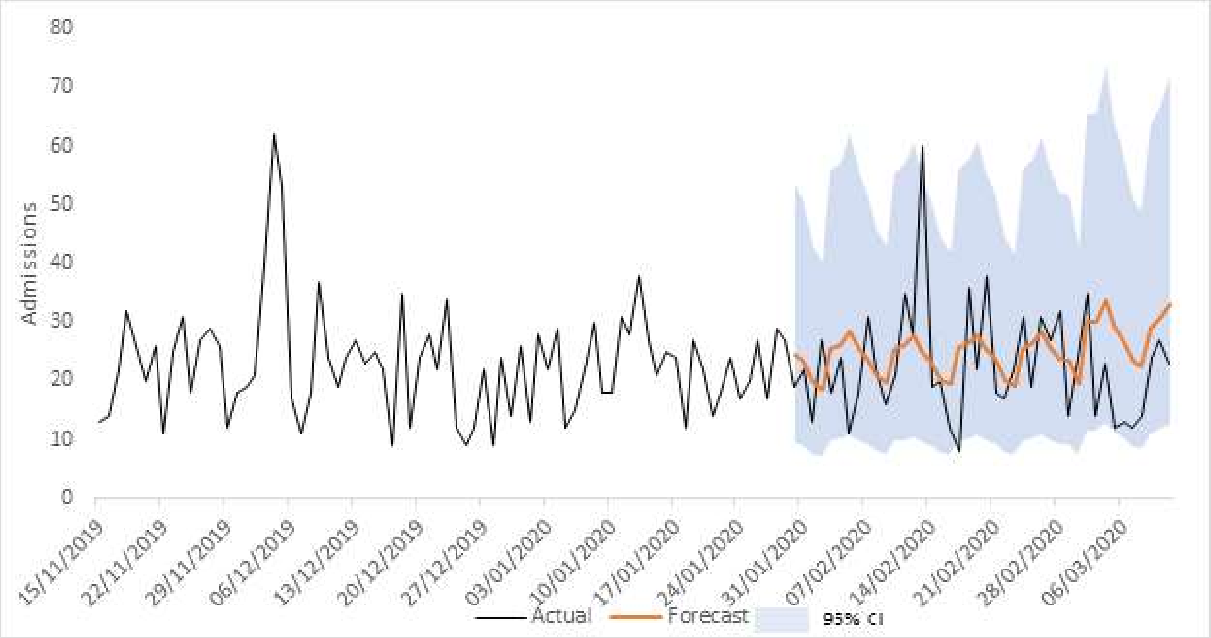

Figure A7: Model 8 (over 48 hour length of stay medicine admissions)

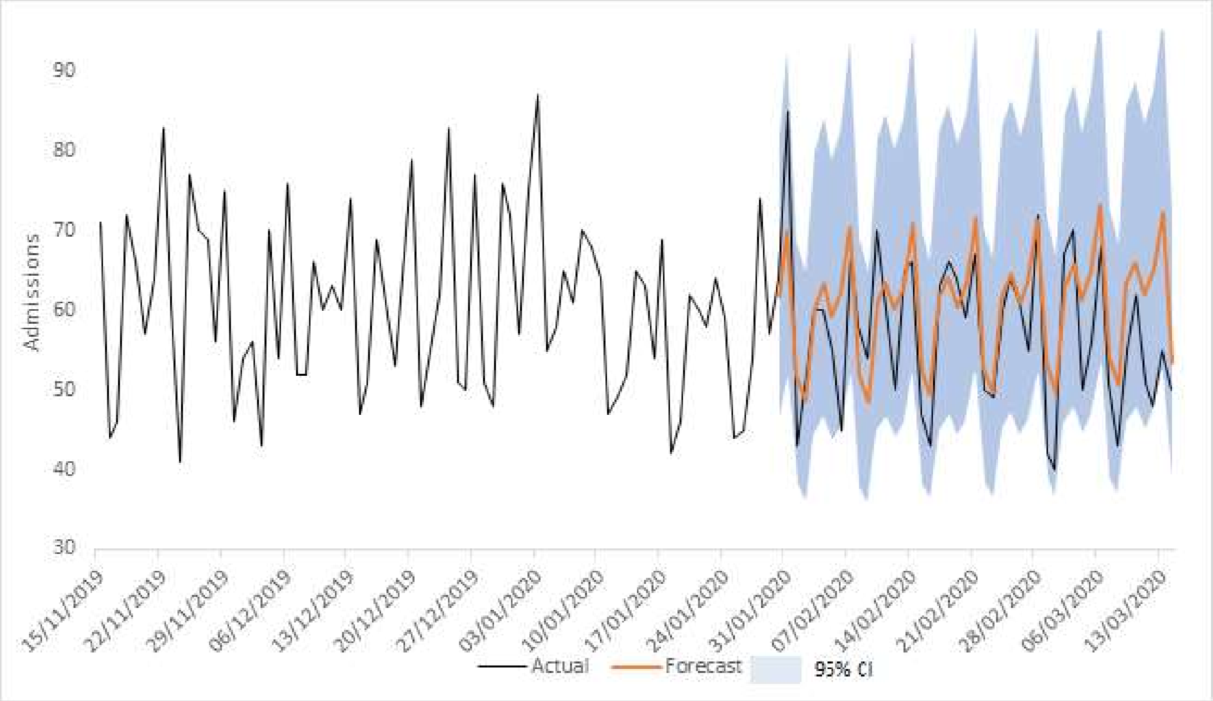

Figure A8: Model 9 (over 48 hour length of stay surgery admissions)

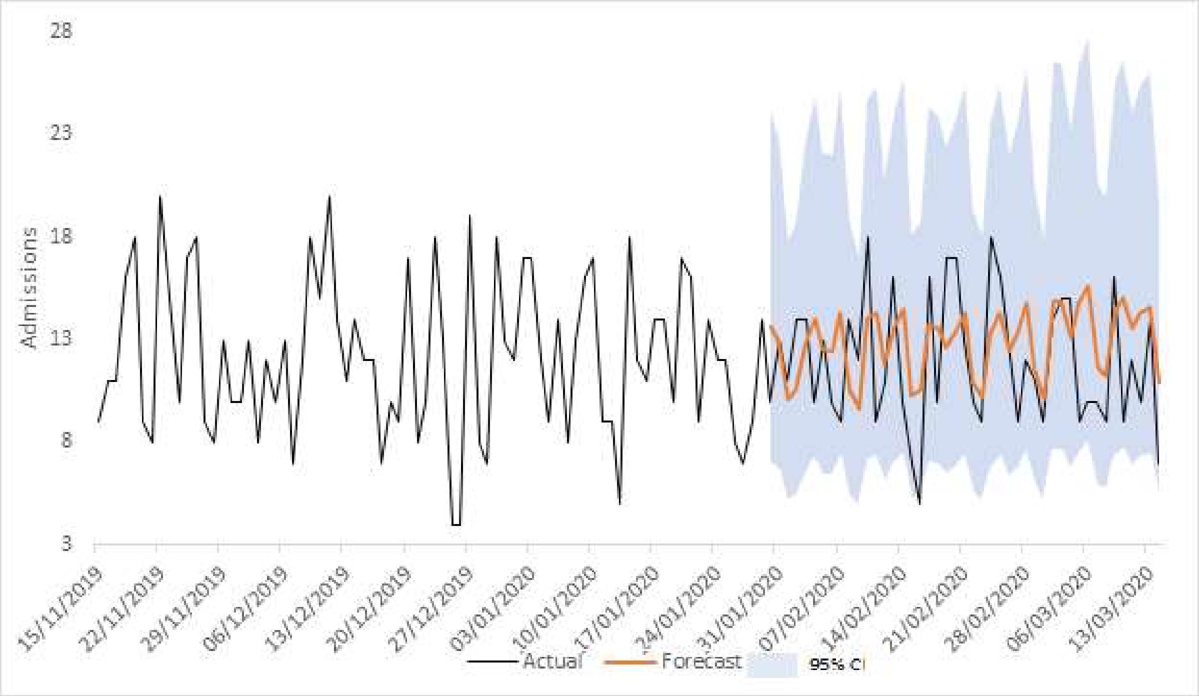

Figure A9: Model 10 (all bed occupancy)

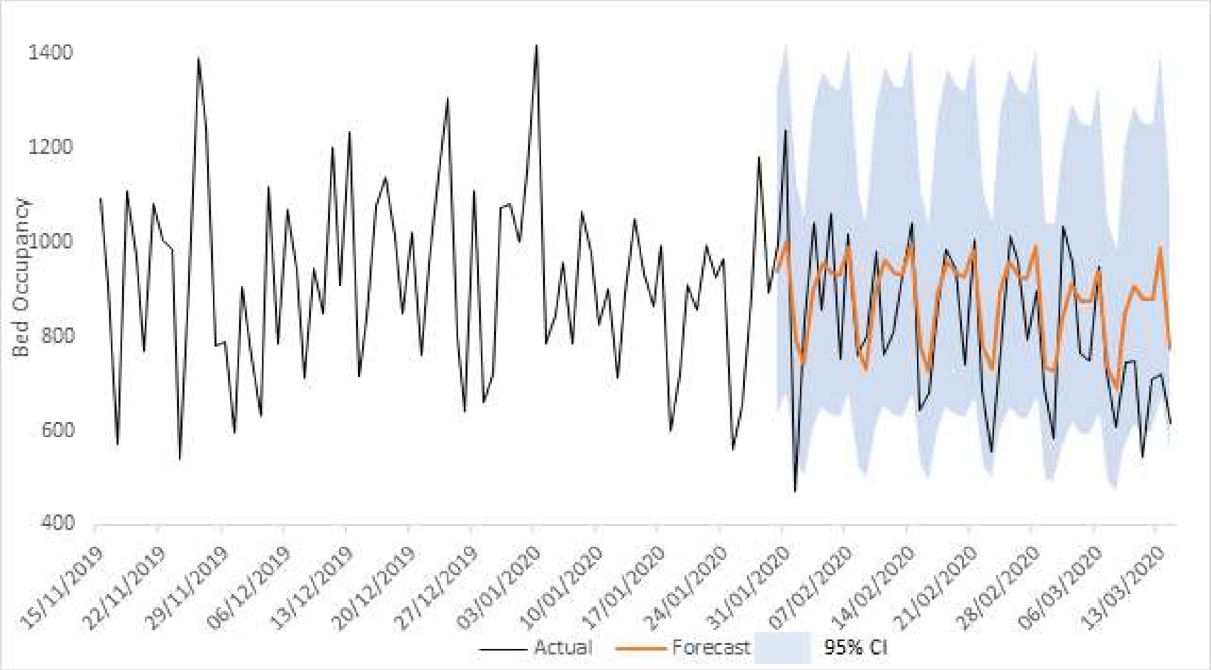

Figure A10: Model 11 (medicine bed occupancy)

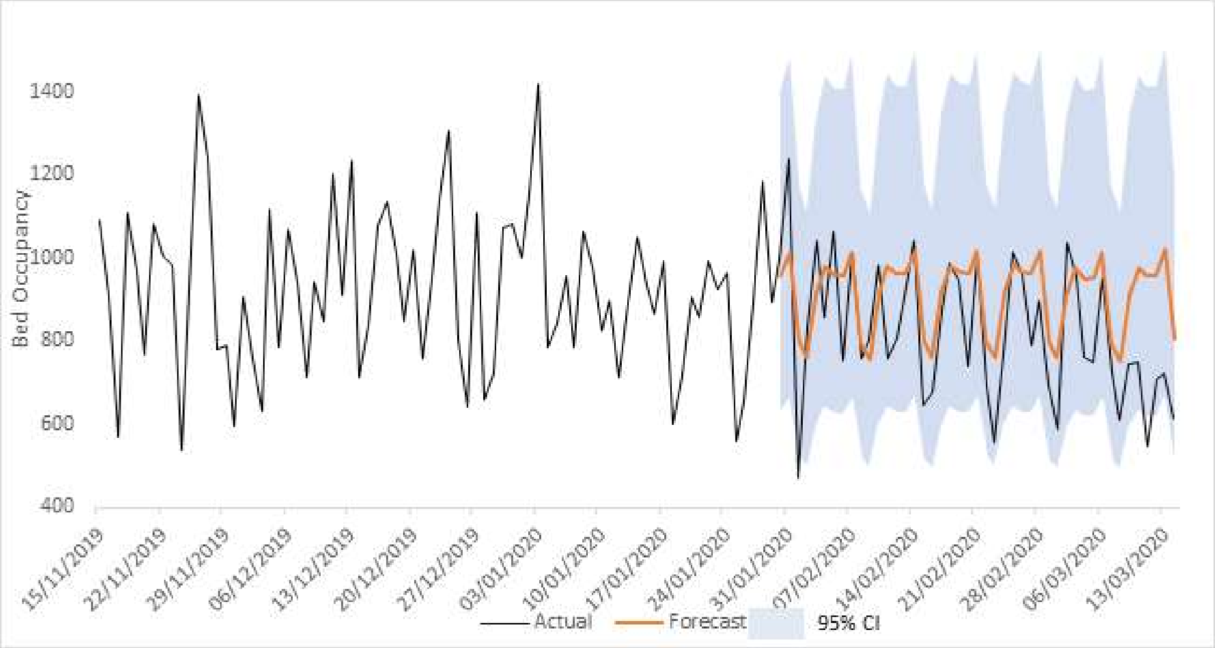

Figure A11: Model 12 (surgery bed occupancy)

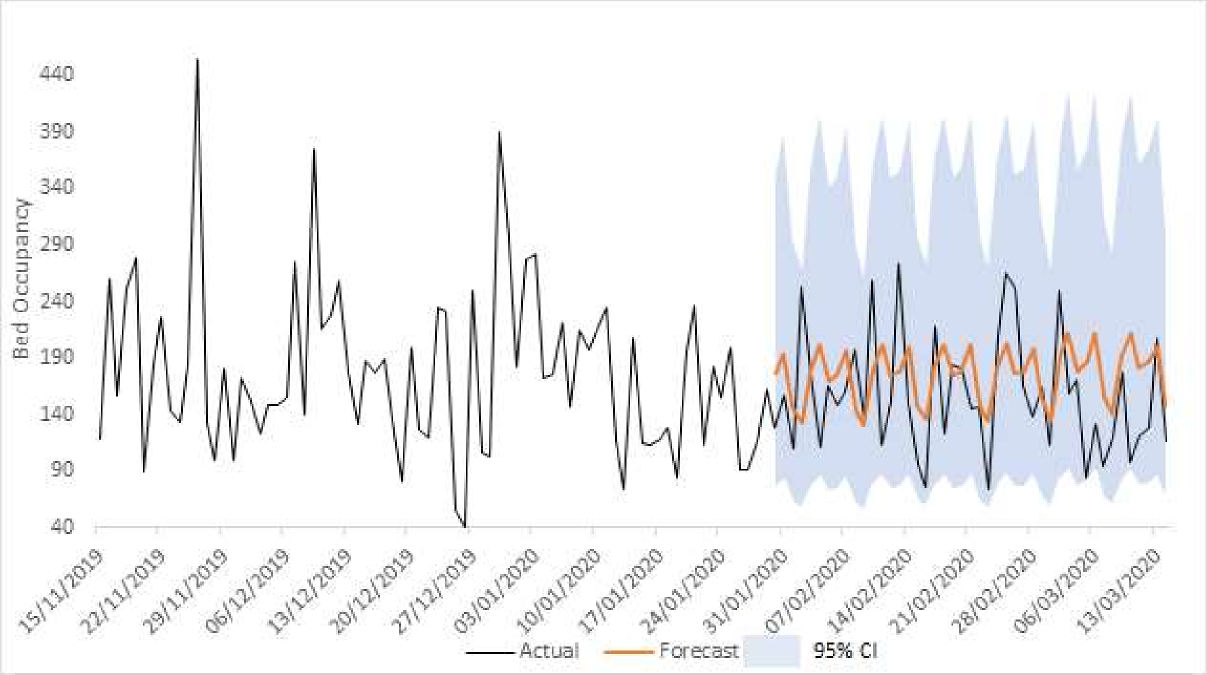

Figure A12: Model 13 (under 48 hour length of stay bed occupancy)

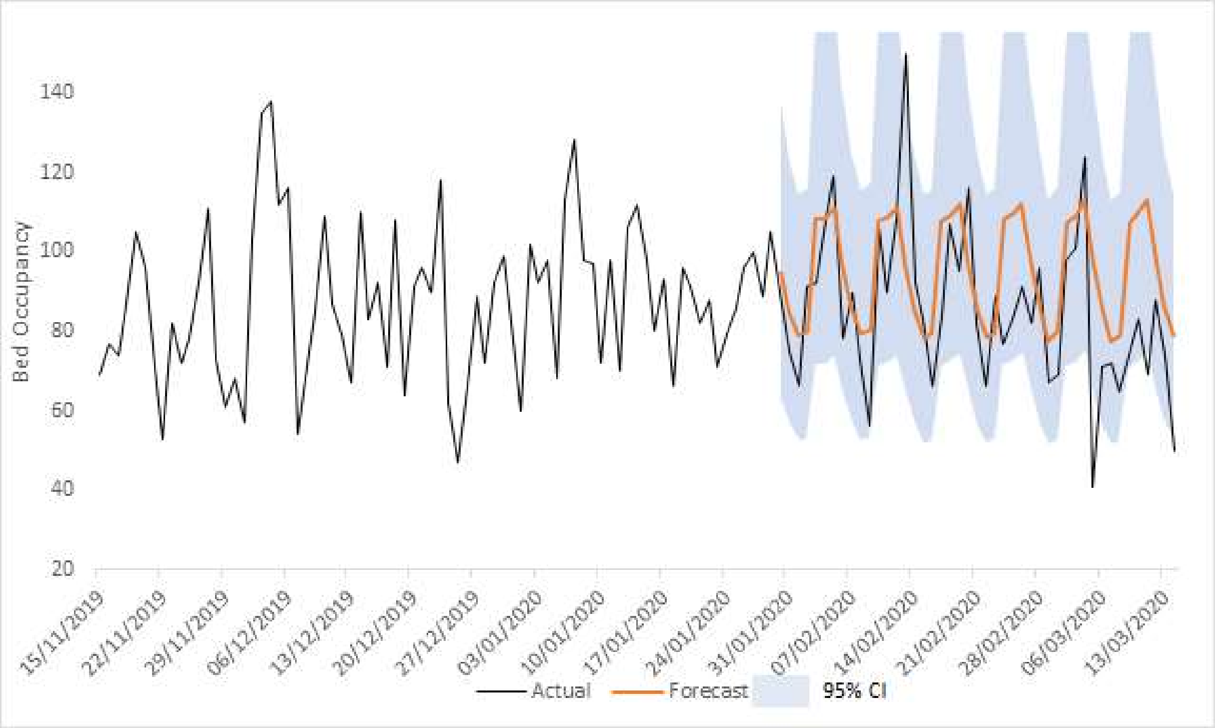

Figure A13: Model 14 (over 48 hour length of stay bed occupancy)

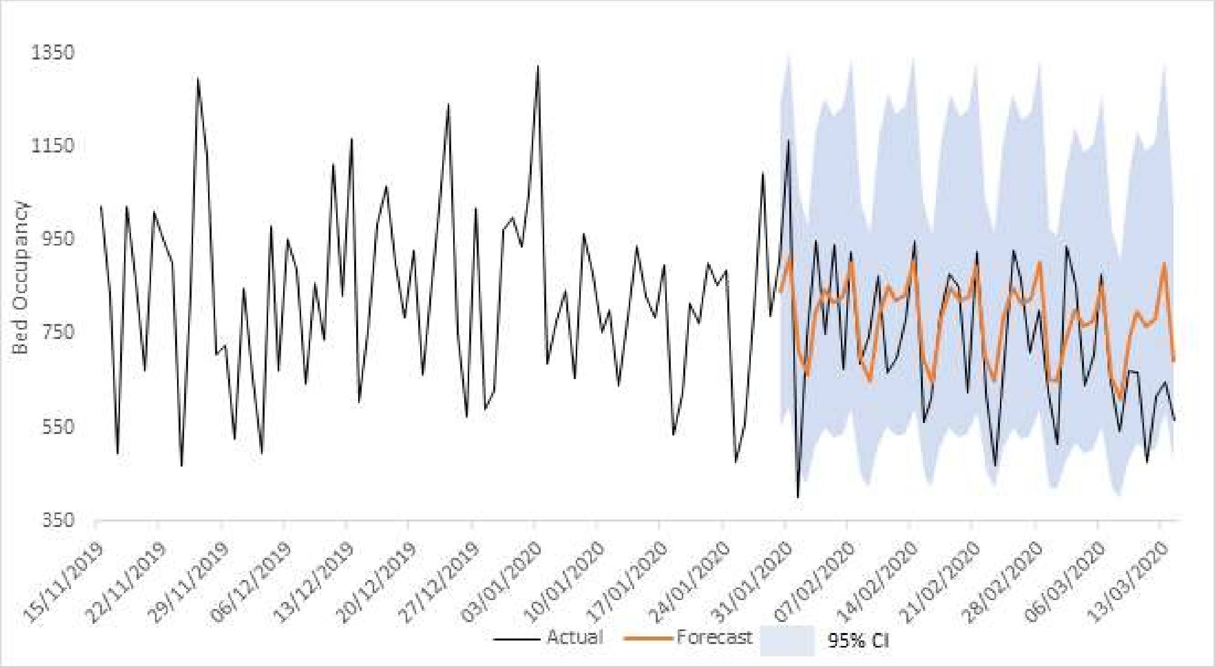

Figure A14: Model 15 (under 48 hour length of stay medicine bed occupancy)

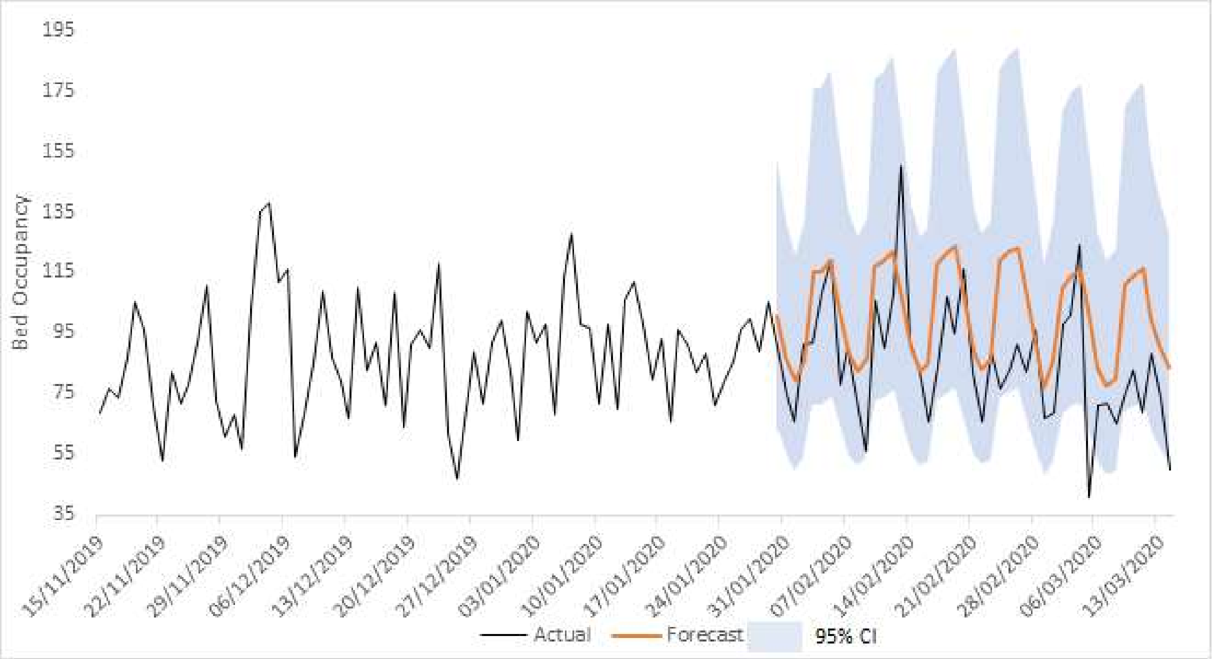

Figure A15: Model 16 (under 48 hour length of stay surgery bed occupancy)

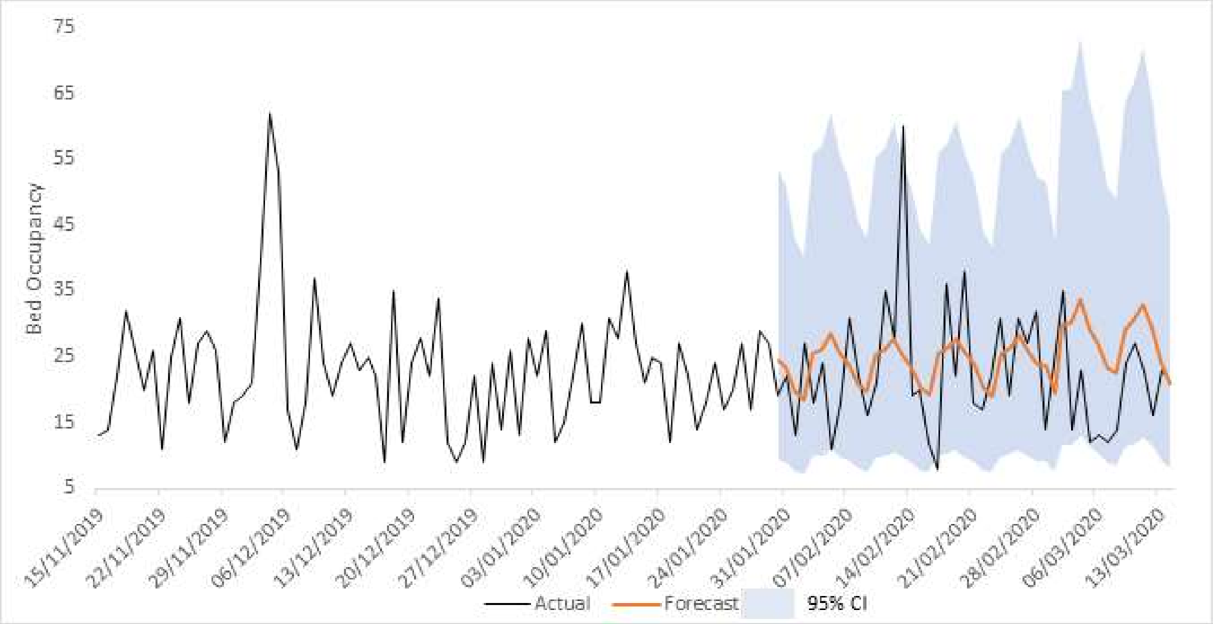

Figure A16: Model 17 (over 48 hour length of stay medicine bed occupancy)

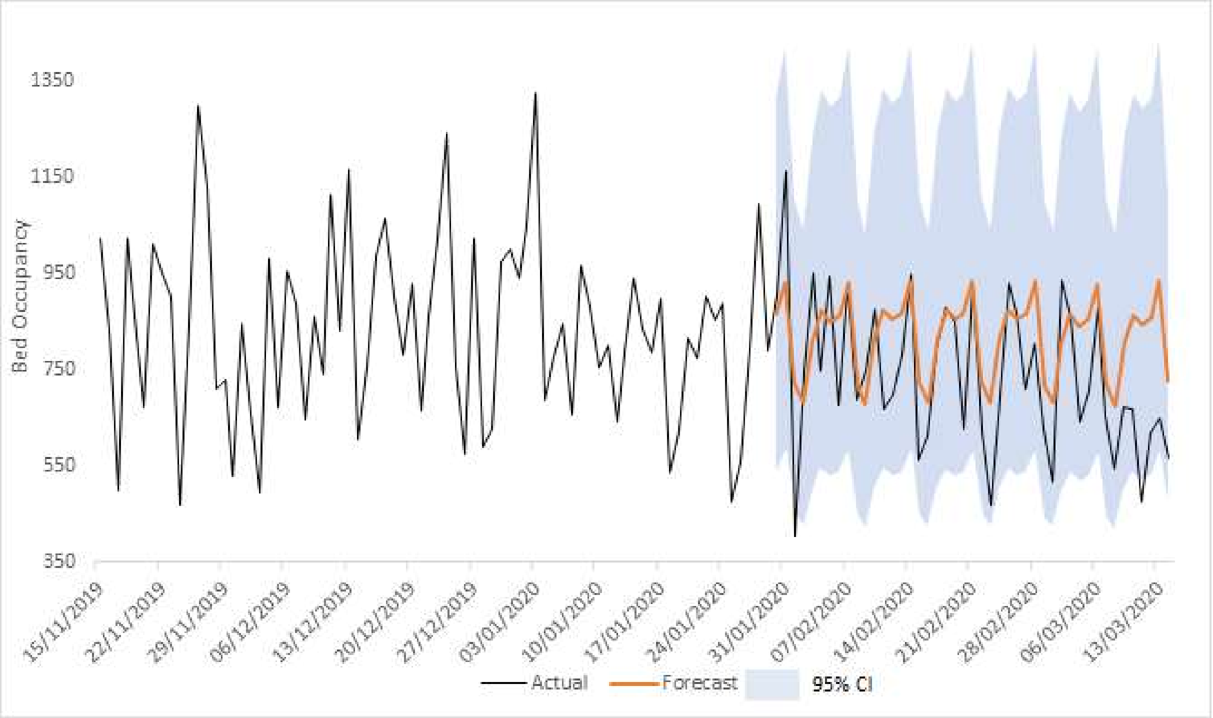

Figure A17: Model 18 (over 48 hour length of stay surgery bed occupancy)

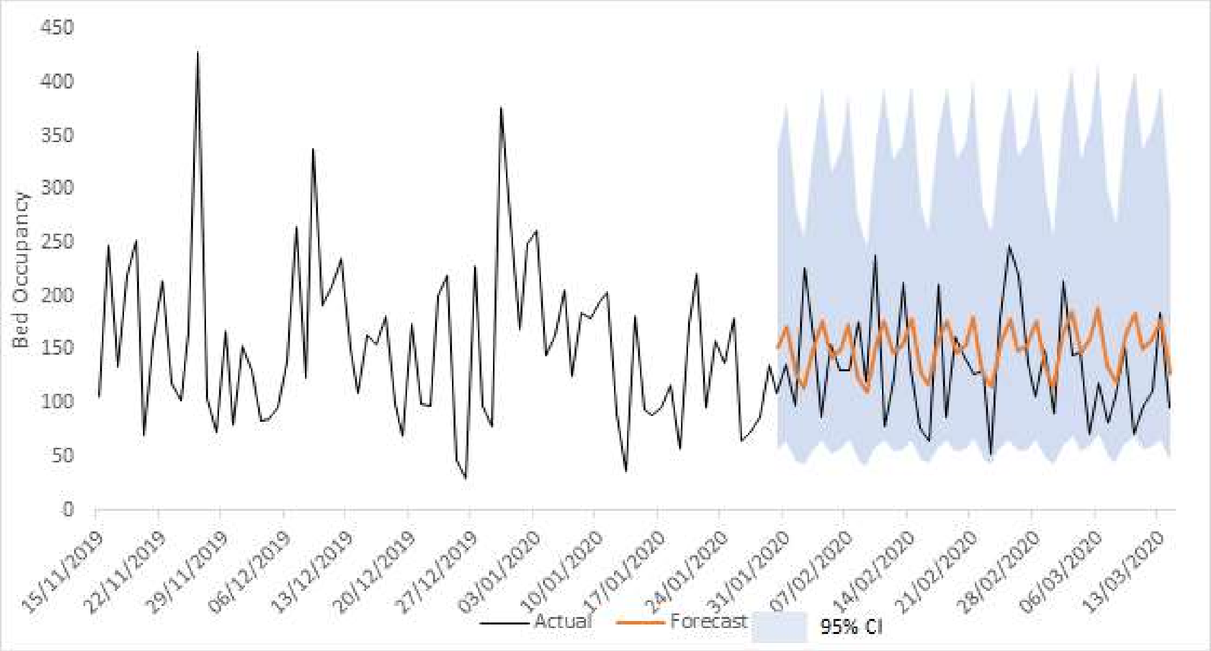

For peer review only

```
#clear the environment of all data
rm(list = ls())

## install necessary packages (Can skip this step if you have them)
install.packages("readstata13", "Hmisc", "DataCombine", "lmtest","tidyverse",
"scales", "forecast", "zoo", "tempdisagg", "imputeTS", "lubridate", "aTSA",
"tseries", "gridExtra", "broom", "knitr", "dplyr", "pixedust")


#tidy regression output libraries
library(broom)
library(knitr)
require(broom)
require(knitr)
library(dplyr) ## this is the library with 'pipes' %>%
library(pixiedust)



## Packages needed
## If you don't have a package, use 'install.packages("NAME OF PACKAGE HERE")
#bring in stata data
library(readstata13)

#lag function
library(Hmisc)

#Row insertion library for missing data problem
library(DataCombine)

#Some tests are in these libraries
library(lmtest)


#tidyverse includes many other useful libraries for formatting data, scales
includes date formatting
library(tidyverse)
library(scales)

#forecasting and time series libraries
library(forecast)
library(zoo)
library(tempdisagg)
library(imputeTS)
library(lubridate)
library(aTSA)
library(tseries)

#laying out graph library
library(gridExtra)
```

For peer review only

```
#tidy regression output libraries
library(broom)
library(knitr)
require(broom)
require(knitr)
library(dplyr) ## this is the library with 'pipes' %>%
library(pixiedust)



# Set file path for forecasting data

setwd("YOUR WORKING DIRECTORY HERE")


# Transfer Stata dataset into R datasets. Use the relevant dataset in 'hosp_data'
hosp_data = read.dta13("COLLAPSED HOSPITAL DATA HERE", convert.factors = TRUE,
generate.factors = TRUE, nonint.factors = TRUE)
temp = read.csv("TEMPERATURE WITH SUFFICIENT LAG (ONE YEAR BEFORE HOSPITAL DATA
START) HERE")
precip = read.dta13("PRECIPITATION WITH SUFFICIENT LAG (ONE YEAR BEFORE HOSPITAL
DATA START) HERE", convert.factors = TRUE, generate.factors = TRUE, nonint.factors
= TRUE)

#Use below if temperature/precipitation data is in dta format from stata, but new
code to combine in R is much more efficient.
#temp = read.dta13("temperatures_lag.dta", convert.factors = TRUE, generate.factors
= TRUE, nonint.factors = TRUE)
#precip = read.dta13("temperatures_lag.dta", convert.factors = TRUE,
generate.factors = TRUE, nonint.factors = TRUE)

#make a full date variable for the temp and precip data to match hosp_data
temp = temp %>%
  mutate(admidate=make_date(year, month, day))

precip = precip %>%
  mutate(admidate=make_date(admiyear, admimonth, admiday))


#check the plot to make sure it looks like you would expect (time series data, with
a count per day)

plot(hosp_data$id)


#pull out the length and the end date of the hospital data, for data manipulation
purposes

leng = nrow(hosp_data)
```

For peer review only

```
end_date=hosp_data$dateindex[leng]
init_date=hosp_data$dateindex[1]


#check the data if there's a huge outlier (often for some of the bed occupancy
subforecasts)

plot(hosp_data$id)

#find the position of the outlier, then make it an average of the previous week's
occupancy/admissions.
#CHECK: max admissions/max occupancy with NBT

outval = which.max(hosp_data$id) #if the outlier is higher
#outval = which.min(hosp_data$id) #if the outlier is lower (this one is commented
out)
hosp_data$id[outval] #displays the value
hosp_data$id[outval] = as.integer(sum(hosp_data$id[(outval-7):(outval-1)])/7)
#makes the outlier an average of the previous 7 days' occupancy/admissions


#sort out the lag temperature - the temperature and precipitation files should have
one year more data in the past, so that you can do this part
#if we didn't do it this way, if we did last week's temperature, we'd have to
predict the weather accurately if our forecast horizon exceeded one week
#in conclusion, since we're not meteorologists, it's probably better to just use
last year's, so we can use whatever horizon we'd like up to a year

temp$last_year = Lag(temp$avg_temp, 365)

#lag precipitation

precip$last_year = Lag(precip$rainfall, 365)

#overwrite the dataframes so that they're the same length as the hospital
admissions data
#the models won't work if the inputs have different lengths

precip= subset(precip, admidate>=init_date & admidate<=end_date)
temp = subset(temp, admidate>=init_date & admidate<=end_date)

#Looking at missingness in the temperature and precipitation data
#Some days may be missing, (NOT IN CURRENT TRANCHE) and missing values need to be
imputed
#If there are no missing values, it will say 'integer (empty)' in the environment

missing_t = which(is.na(temp$last_year))
missing_p = which(is.na(precip$last_year))

#A quick fix for NAs in the temperature and precipitation data, if there
```

For peer review only

```
#are missing values, but don't run if there aren't.

temp$last_year[missing_t] = na.spline(temp$last_year)
precip$last_year[missing_t] = na.spline(precip$last_year)

#Check for missing dates - for subforecasts this might be an issue for those with
small numbers
#Make sure to put 0s in when preparing subforecast data in Stata rather than
allowing missingness
#If there are missing dates, go back to the preparation stage.
#It will say 'Date' num(0) in the global environment if there are no missing dates

## THIS SECTION IS ONLY FOR IF THERE ARE MISSING COUNT DATA ON ANY DAY ##

missing_d = as.Date(setdiff(precip$admidate,hosp_data$dateindex))

#If they are missing, add them in as '0' values (missingness means there was
#no occupancy on those dates, as far as I understand).

#First create a new dataframe with the information that's missing
#Name the columns Add the new dataframe to the end of the old one, then sort
("arrange")

add_miss = data.frame(missing_d, 1, month(missing_d), year(missing_d),
day(missing_d), wday(missing_d), 0)
names(add_miss)= c("dateindex", "id", "month", "year", "day", "dow", "yearmonth")
hosp_data=rbind(hosp_data, add_miss)
hosp_data=hosp_data %>% arrange(dateindex)
rm(add_miss)


leng=nrow(hosp_data)

#tidy up a bit
rm(missing_d,missing_t,missing_p)

#check and record the mean and standard deviation of the data
mean(hosp_data$id)
sd(hosp_data$id)


#Transform the dataframes into time series objects, with a daily 'seasonality'

hospts <- msts(hosp_data, seasonal.periods = c(7,365.25), ts.frequency = 7)
tempts <- msts(temp, seasonal.periods = c(7,365.25), ts.frequency = 7)
precipts <- msts(precip, seasonal.periods = c(7,365.25), ts.frequency = 7)



#Check ACF plot of the count to confirm the presence of autocorrelation.
```

For peer review only

```
#If the values fall between the blue lines (p<0.05), then the data are not
#likely to be autocorrelated. If they do fall outside the lines, they are likely to
be.
#This uses a lag of 21 days
# "The ACF of stationary data drops to zero relatively quickly"
# "The ACF of non-starionary data decreases slowly"

acf(hospts[,"id"], lag = 21)

#Check Partial ACF plot to confirm that the autocorrelation is or isn't a result of
the
#autocorrelation of the first lag carrying through (if it is, the spikes on the
PACF will
#peak at the first lag, then be in between the blue lines (p <0.05))
# "A partial autocorrelation is the amount of correlation between a variable
#and a lag of itself that is not explained by correlations at all
lower-order-lags."
#This uses a lag of 21 days

pacf(hospts[,"id"], lag = 21)

#Fit a regression model to formally test for a lagged autocorrelation in the
residuals

#create a time variable
timef <- seq_along(hospts[,"id"])

#fit a linear regression to model the series as a function of time
lin_reg = tslm(hospts[,"id"] ~ timef)

#Durbin-Watson test - check for lag-1 autocorrelated residuals
#H0 = residuals do not have lag-1 auotocorrelation
#small p -> there is evidence for lag-1 autocorrelation

dwtest(lin_reg)

#tidy up
rm(timef, lin_reg)


#Determine stationarity of the data (it is non-stationary if it has a trend,
seasonal
#effect, or any systematic change in mean and variance


#Seasonal decomposition graphs (including weekly and annual seasonality)
#These are decomposed to the full sample and also financial years

## You may need to adjust these to the years of your data (the label), but the
actual end of the window
```

For peer review only

```
##is one year long##

hospts[,"id"] %>% mstl(lambda = 0) %>% autoplot() + xlab("week") +
  ggtitle("Estimated components (full sample)")

#2015-2016 (check the weeks of the year for these)

hospts[,"id"] %>% mstl(lambda = 0) %>% window(end = 52) %>% autoplot(facet=TRUE) +
xlab("week") +
  ggtitle("Estimated components (2015-2016)")

#2016-2017 (check the weeks of the year for these)

hospts[,"id"] %>% mstl(lambda = 0) %>% window(start = 53, end = 105) %>%
autoplot(facet=TRUE) +
  xlab("week") + ggtitle("Estimated components (2016-2017)")

#2017-2018 (check the weeks of the year for these)

hospts[,"id"] %>% mstl(lambda = 0) %>% window(start = 106, end = 158) %>%
autoplot(facet=TRUE) +
  xlab("week") + ggtitle("Estimated components (2017-2018)")

#2018-2019 (check the weeks of the year for these)

hospts[,"id"] %>% mstl(lambda = 0) %>% window(start = 159, end = 211) %>%
autoplot(facet=TRUE) +
  xlab("week") +ggtitle("Estimated components (2018-2019)")

#2019-'2020' (check the weeks of the year for these -
#keep in mind that we currently don't have all the data for this financial year

hospts[,"id"] %>% mstl(lambda = 0) %>% window(start = 212, end = 250) %>%
autoplot(facet=TRUE) +
  xlab("week") +ggtitle("Estimated components (2019-2020)")

# Check the seasonality of the data - annual seasonality often includes Christmas
(push to discharge before Christmas) and at times Easter
# There is also usually weekly seasonality. Check the trend of the data as well.

#Conduct an augmented Dickey-Fuller test (ADF) test to formally test for
stationarity
#H0 = non-stationary, H1 = stationary. low p value = reject the null

adf.test(hospts[,"id"])

#If the data are not stationary (i.e. high p), transform them to be stationary
(differencing)
#if the adf test rejects the null, then don't difference as they are stationary.
#if you difference once, and it still isn't stationary, consider differencing the
```

For peer review only

```
differenced data
#or, log the data (if the variance is unstable). Currently commented out.

#hospts = diff(hospts)

#Onto modelling, creating the data. Please note if you differenced the data, you
will need to change the variable names:

# Create deterministic trend and log deterministic trend

trend <- seq_along(hospts[,"id"])

# Create dummy daily variables

mon <- ifelse(hospts[,"dow"] == 1, 1, 0) # create dummy variable equal to 1 if
Monday and 0 otherwise
tue <- ifelse(hospts[,"dow"] == 2, 1, 0)
wed <- ifelse(hospts[,"dow"] == 3, 1, 0)
thu <- ifelse(hospts[,"dow"] == 4, 1, 0)
fri <- ifelse(hospts[,"dow"] == 5, 1, 0)
sat <- ifelse(hospts[,"dow"] == 6, 1, 0)

# Create dummmy(ies) for Christmas effect

chris <- ifelse(hospts[,"day"] == 25 & hospts[,"month"] == 12, 1, 0)
chrisweek <- ifelse(hospts[,"day"] %in% c(23, 24, 25, 26, 27) & hospts[,"month"] ==
12, 1, 0)
easter2015 <- ifelse(hospts[,"day"] %in% c(3,4,5,6) & hospts[,"month"] == 4, 1, 0
& hospts[,"year"] == 2015)
easter2016 <- ifelse(hospts[,"day"] %in% c(25,26,27,28) & hospts[,"month"] == 3, 1,
0  & hospts[,"year"] == 2016)
easter2017 <- ifelse(hospts[,"day"] %in% c(14,15,16,17) & hospts[,"month"] == 4, 1,
0  & hospts[,"year"] == 2017)
easter2018a <- ifelse(hospts[,"day"] %in% c(30,31) & hospts[,"month"] == 3, 1, 0  &
hospts[,"year"] == 2018)
easter2018b <- ifelse(hospts[,"day"] %in% c(1,2) & hospts[,"month"] == 4, 1, 0  &
hospts[,"year"] == 2018)
easter2018 <- easter2018a + easter2018b
easter2019 <- ifelse(hospts[,"day"] %in% c(19,20,21,22) & hospts[,"month"] == 4, 1,
0  & hospts[,"year"] == 2019)

easterall <- easter2015 + easter2016 + easter2017 + easter2018 + easter2019

bhm2015 <- ifelse(hospts[,"day"] %in% c(4,25) & hospts[,"month"] == 5, 1, 0  &
hospts[,"year"] == 2015)
bha2015 <- ifelse(hospts[,"day"] %in% c(31) & hospts[,"month"] == 8, 1, 0  &
hospts[,"year"] == 2015)
bh2015 <- bhm2015 + bha2015
bhj2016 <- ifelse(hospts[,"day"] %in% c(1) & hospts[,"month"] == 1, 1, 0  &
hospts[,"year"] == 2016)
```

For peer review only

```
bhm2016 <- ifelse(hospts[,"day"] %in% c(2,30) & hospts[,"month"] == 5, 1, 0  &
hospts[,"year"] == 2016)
bha2016 <- ifelse(hospts[,"day"] %in% c(29) & hospts[,"month"] == 8, 1, 0  &
hospts[,"year"] == 2016)
bh2016 <- bhj2016 + bhm2016 + bha2016
bhj2017 <- ifelse(hospts[,"day"] %in% c(2) & hospts[,"month"] == 1, 1, 0  &
hospts[,"year"] == 2017)
bhm2017 <- ifelse(hospts[,"day"] %in% c(1,29) & hospts[,"month"] == 5, 1, 0  &
hospts[,"year"] == 2017)
bha2017 <- ifelse(hospts[,"day"] %in% c(28) & hospts[,"month"] == 8, 1, 0  &
hospts[,"year"] == 2017)
bh2017 <- bhj2017 + bhm2017 + bha2017
bhj2018 <- ifelse(hospts[,"day"] %in% c(1) & hospts[,"month"] == 1, 1, 0  &
hospts[,"year"] == 2018)
bhm2018 <- ifelse(hospts[,"day"] %in% c(7,28) & hospts[,"month"] == 5, 1, 0  &
hospts[,"year"] == 2018)
bha2018 <- ifelse(hospts[,"day"] %in% c(27) & hospts[,"month"] == 8, 1, 0  &
hospts[,"year"] == 2018)
bh2018 <- bhj2018 + bhm2018 + bha2018
bhj2019 <- ifelse(hospts[,"day"] %in% c(1) & hospts[,"month"] == 1, 1, 0  &
hospts[,"year"] == 2019)
bhm2019 <- ifelse(hospts[,"day"] %in% c(6,27) & hospts[,"month"] == 5, 1, 0  &
hospts[,"year"] == 2019)
bha2019 <- ifelse(hospts[,"day"] %in% c(26) & hospts[,"month"] == 8, 1, 0  &
hospts[,"year"] == 2019)
bh2019 <- bhj2019 + bhm2019 + bha2019

bhall <- bh2015 + bh2016 + bh2017 + bh2018 + bh2019

hollall <- easterall + bhall +chrisweek

#clean up the extra variables we don't need

rm(bhm2015,bha2015,bh2015,bhj2016,bhm2016,bha2016,bhj2017,bhm2017,bha2017,bhj2018,b
hm2018,bha2018,bhj2019,bhm2019,bha2019,chris)
rm(easter2015,easter2016,easter2017,easter2018,easter2018a,easter2018b,easter2019)


# Create a matrix of regressors for trend and daily dummies

trend_days <- tibble(trend, mon, tue, wed, thu, fri, sat, chrisweek, easterall,
bhall, tempts[,"last_year"], precipts[,"last_year"]) # create matrix of regressors:
trend + daily dummies
trend_days_noCLIM <- tibble(trend, mon, tue, wed, thu, fri, sat, chrisweek,
hollall) # create matrix of regressors: trend + daily dummies


#create the time series with the regressors included

seasonality <- msts(trend_days, seasonal.periods = c(7,365), ts.frequency = 7)
```

For peer review only

```
seasonality_noCLIM <-  msts(trend_days_noCLIM, seasonal.periods = c(7,365),
ts.frequency = 7)


# Estimate models with and without climatic data - other model types can be found
in the code graveyard

fit_trend <- auto.arima(hospts[,"id"], d=0, xreg=cbind(seasonality), lambda=0,
biasadj=TRUE)
fit_trend_noCLIM <- auto.arima(hospts[,"id"], d=0, xreg=cbind(seasonality_noCLIM),
lambda=0, biasadj=TRUE)

#Checks for the deterministic trend

summary(fit_trend) #to get model fit parameters manually
res_trend <- residuals(fit_trend)
checkresiduals(fit_trend, points = FALSE)
ggPacf(res_trend, lag=70)
adf.test(res_trend)
trend_nonseas = c(fit_trend$arma[1], fit_trend$arma[6], fit_trend$arma[2]) #pull
out the nonseasonal (first part) ARIMA parameters
trend_seas = c(fit_trend$arma[3], fit_trend$arma[7], fit_trend$arma[4]) #pull out
the seasonal (second part) ARIMA parameters

#Checks for the deterministic trend without climate data
summary(fit_trend_noCLIM) #to get model fit parameters manually
res_trend_noCLIM <- residuals(fit_trend_noCLIM)
checkresiduals(fit_trend_noCLIM, points = FALSE)
ggPacf(res_trend_noCLIM, lag=70)
adf.test(res_trend)
trend_noCLIM_nonseas = c(fit_trend_noCLIM$arma[1], fit_trend_noCLIM$arma[6],
fit_trend_noCLIM$arma[2]) #pull out the nonseasonal (first part) ARIMA parameters
trend_noCLIM_seas = c(fit_trend_noCLIM$arma[3], fit_trend_noCLIM$arma[7],
fit_trend_noCLIM$arma[4]) #pull out the seasonal (second part) ARIMA parameters


# Forecasting on training and test sets - 6 week horizon

train.hospts <- subset(hospts[,"id"], end = length(hospts[,"id"]) - 45)
test.hospts <- subset(hospts[,"id"], start=length(hospts[,"id"])-44)

lentrain=length(train.hospts)


#xlim=c(230,241) to examine the training/test split
train.hospts %>% autoplot()+
  autolayer(test.hospts, color = "red")


#deterministic trend
```

For peer review only

```
trend_model <- Arima(train.hospts, order=trend_nonseas, seasonal=trend_seas,
include.constant = TRUE,
                     xreg = cbind(seasonality[1:lentrain,]),
                     lambda=0, biasadj=TRUE)

trend_fcast <- forecast(trend_model, level = c(95), xreg =
cbind(seasonality[(lentrain+1):leng,]))

trend_fcast %>% autoplot() +
  autolayer(test.hospts, series="Actual value", color = 'darkorange') +
  guides(colour=guide_legend(title="")) +
  ggtitle("Deterministic trend model") +
  ylab("Daily admissions")


#deterministic trend no climate data

trend_model_noCLIM <- Arima(train.hospts, order=trend_noCLIM_nonseas,
seasonal=trend_noCLIM_seas, include.constant = TRUE,
                     xreg = cbind(seasonality_noCLIM[1:lentrain,]),
                     lambda=0, biasadj=TRUE)

trend_fcast_noCLIM <- forecast(trend_model_noCLIM, level = c(95), xreg =
cbind(seasonality_noCLIM[(lentrain+1):leng,]))

trend_fcast_noCLIM %>% autoplot() +
  autolayer(test.hospts, series="Actual value", color = 'darkorange') +
  guides(colour=guide_legend(title="")) +
  ggtitle("Deterministic trend model no climate") +
  ylab("Daily admissions")




# assess the accuracy - for AIC/BIC check model summaries - summary(trend_model)
etc

accuracy(trend_model$fitted, hospts[,"id"])
accuracy(trend_model_noCLIM$fitted, hospts[,"id"])


#residual differences tables
diff_trend = hospts[(lentrain+1):leng,"id"] - trend_fcast$mean
date = as.character(paste(hospts[(lentrain+1):leng,"year"],
hospts[(lentrain+1):leng,"month"],  hospts[(lentrain+1):leng,"day"], sep="/"))
dow = factor(as.character(hospts[(lentrain+1):leng,"dow"]), levels =
c(0,1,2,3,4,5,6), labels = c("Sun", "Mon", "Tues", "Weds", "Thurs", "Fri", "Sat"))

#admissions values
```

For peer review only

```
acc_trend = abs(diff_trend) <= (0.2*mean(hosp_data$id))
acc_trend_s = abs(diff_trend) < (0.1*mean(hosp_data$id))


##put it into a nice format
diff_trend_df=data.frame("date" = date, "dow" = dow , "actual" =
hospts[(lentrain+1):leng,"id"], "predicted" = trend_fcast$mean, "difference"=
diff_trend, "lower95" = trend_fcast$lower, "upper95" = trend_fcast$upper,
"accuracybig" = acc_trend,  "accuracybetter" = acc_trend_s)

##write to a csv (easier to copy)- when you open it in Excel the date column will
look weird - just make sure to format it as a date in Excel and pick a format that
shows up properly - also you can reduce the df in excel
write.csv(diff_trend_df, file="Trend_diff.csv", row.names=FALSE)

##write all the data to a csv for making graphs
write.csv(hosp_data, file="all_data.csv", row.names=FALSE)
```

For peer review only

Appendix 3: Model specifications and AICs for models with and without climatic data

Table A3.1: Model specifications. Guide to acronyms: a- admissions, bo- bed occupancy, o48 – over 48 hour length of stay, u48 – under 48 hour length of stay, med – medicine, surg - surgery

A/BO	Time	Climate	Speci alty	SARIMA	RMSE	MAPE	mean	sd	AIC
a	all	y	all	(3,0,0)(1,0,0)	9.59	8.66	90.52	15.02	-2069.49
a	all	n	all	(2,0,2)(1,0,0)	9.50	8.59	90.52	15.02	-2043.83
a	all	y	med	(3,0,2)(2,0,2)	8.98	9.44	79.14	19.32	-1817.45
a	all	n	med	(3,0,2)(2,0,2)	8.98	9.43	79.14	19.32	-1813.09
a	o48	y	all	(3,0,1)(1,0,1)	7.22	10.78	55.14	10.04	-1496.64
a	o48	n	all	(2,0,3)(1,0,0)	7.25	10.83	55.14	10.04	-1495.55
a	o48	y	med	(5,0,1)(2,0,0)	6.88	11.97	48.55	12.06	-1242.32
a	o48	n	med	(5,0,1)(2,0,0)	6.88	11.97	48.55	12.06	-1237.50
a	o48	y	surg	(3,0,3)(1,0,2)	3.40	27.45	11.87	3.74	678.12
a	o48	n	surg	(4,0,4)(0,0,2)	3.41	27.47	11.87	3.74	681.20
a	all	y	surg	(1,0,2)(0,0,0)	4.59	19.86	20.65	5.31	-7.37
a	all	n	surg	(2,0,1)(2,0,0)	4.58	19.87	20.65	5.31	-1.96
a	u48	y	all	(1,0,0)(1,0,0)	6.81	16.53	35.38	9.06	-471.22
a	u48	n	all	(2,0,3)(1,0,0)	6.78	16.47	35.38	9.06	-463.39
a	u48	y	med	(4,0,0)(2,0,0)	6.45	18.64	30.59	10.21	-177.19
a	u48	n	med	(4,0,0)(2,0,0)	6.46	18.67	30.59	10.21	-176.50
a	u48	y	surg	(0,0,1)(0,0,0)	7.82	44.19	20.99	8.39	1583.52
a	u48	n	surg	(0,0,2)(0,0,0)	7.85	44.51	20.99	8.39	1583.97
bo	all	y	all	(0,0,1)(1,0,0)	158.24	15.57	849.71	179.77	-620.10
bo	all	n	all	(1,0,3)(1,0,0)	158.17	15.57	849.71	179.77	-618.59
bo	all	y	med	(2,0,1)(0,0,0)	151.69	17.06	760.03	189.22	-423.18
bo	all	n	med	(2,0,1)(0,0,0)	151.75	17.06	760.03	189.22	-418.86
bo	o48	y	all	(0,0,1)(1,0,0)	159.55	17.60	767.41	177.20	-330.94
bo	o48	n	all	(1,0,3)(1,0,0)	159.42	17.62	767.41	177.20	-330.48
bo	o48	y	med	(2,0,1)(0,0,0)	152.70	19.15	689.20	181.24	-153.63
bo	o48	n	med	(2,0,1)(0,0,0)	152.76	19.15	689.20	181.24	-149.13
bo	o48	y	surg	(0,0,1)(0,0,0)	64.95	44.22	141.63	67.49	1661.21
bo	o48	n	surg	(0,0,1)(0,0,0)	65.09	44.36	141.63	67.49	1662.07
bo	all	y	surg	(0,0,1)(0,0,0)	64.50	35.40	162.62	67.71	1248.15
bo	all	n	surg	(0,0,1)(0,0,0)	64.63	35.45	162.62	67.71	1248.44
bo	u48	y	all	(3,0,0)(2,0,1)	15.72	16.41	82.30	21.06	-468.39
bo	u48	n	all	(3,0,0)(2,0,1)	15.73	16.42	82.30	21.06	-465.24
bo	u48	y	med	(4,0,0)(2,0,0)	14.95	18.63	70.83	23.95	-170.75
bo	u48	n	med	(4,0,0)(2,0,1)	14.88	18.61	70.83	23.95	-164.97
bo	u48	y	surg	(0,0,1)(0,0,0)	7.82	44.19	21.00	8.38	1583.52
bo	u48	n	surg	(0,0,2)(0,0,0)	7.85	44.51	21.00	8.38	1583.97

For peer review only

Appendix 4: Sensitivity Analyses

The results of the sensitivity analyses are presented here. Two other horizons were tested: One from November to December of 2019, and one from May to June of 2020. These analyses were performed on the overall admissions data.

Table A4.1: Model specifications and accuracy

Model	SARIMA (p,d,q)(P, D,Q)	Model accuracy (strict/moderate thresholds)	MAPE	RMSE	Mean of Outcome	SD of outcome	AIC
Dec 2019	(2,0,2) (1,0,0)	75.6%/93.3%	8.57	9.43	90.09	14.97	-1933.76
June 2020	(3,0,1) (1,0,1)	42.2%/77.8%	8.88	9.67	89.28396	15.79	-2063.21

Figure A4.1: Forecast versus Actual values for December 2019 Horizon.

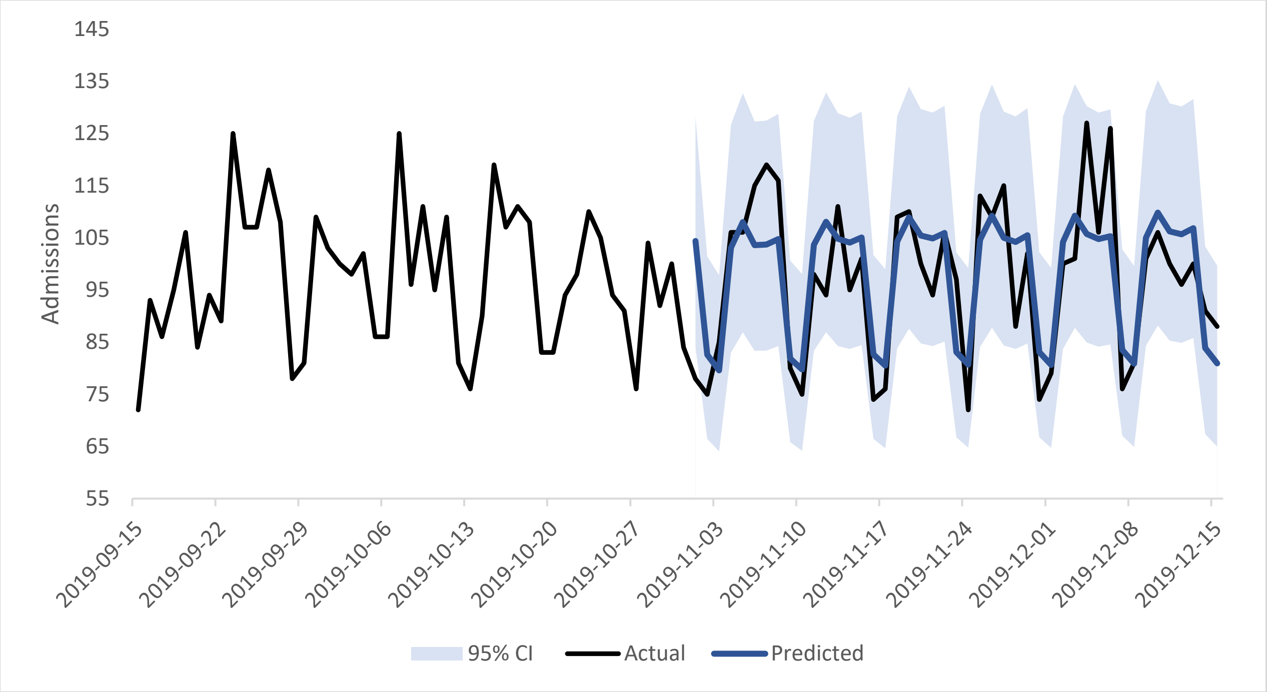

Figure A4.2: Forecast versus Actual values for June 2020 Horizon

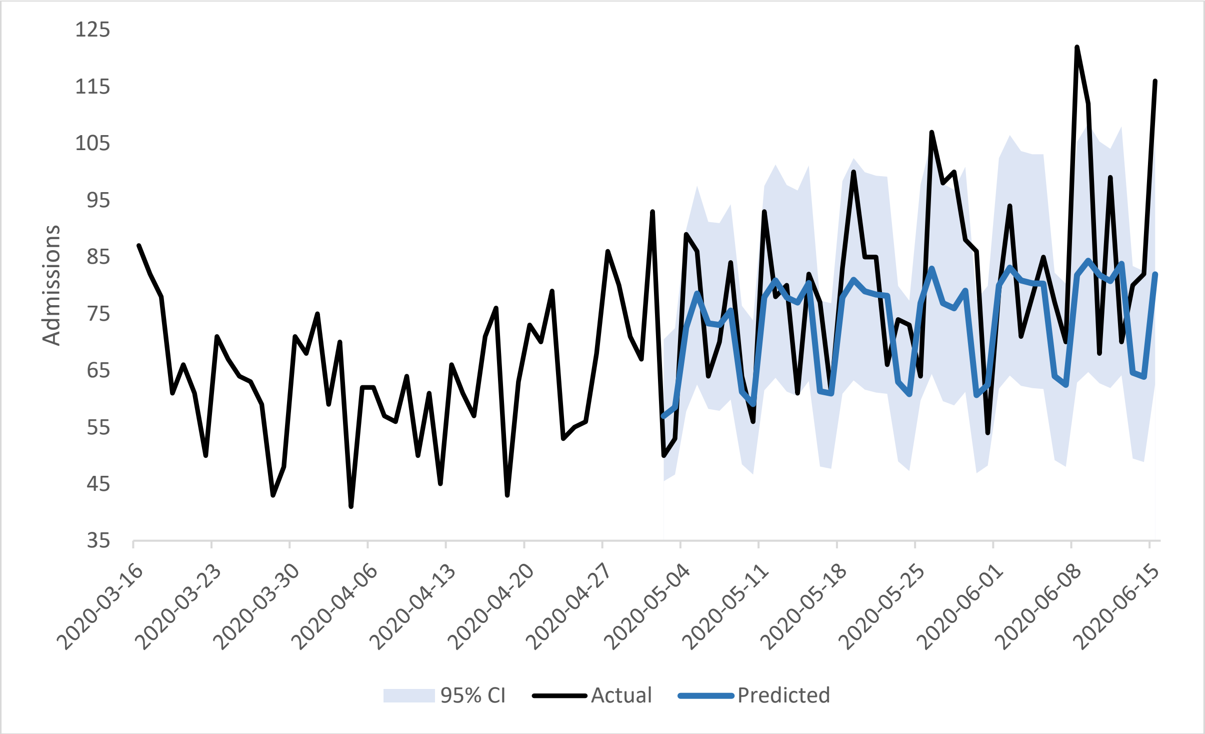

For peer review only

TRIPOD Checklist: Prediction Model Development

1Section/Topic	Item	Checklist Item	Page
**Title and abstract**			
Title	1	Identify the study as developing and/or validating a multivariable prediction model, the target population, and the outcome to be predicted.	1
Abstract	2	Provide a summary of objectives, study design, setting, participants, sample size, predictors, outcome, statistical analysis, results, and conclusions.	2
**Introduction**			
Background and objectives	3a	Explain the medical context (including whether diagnostic or prognostic) and rationale for developing or validating the multivariable prediction model, including references to existing models.	2-4
	3b	Specify the objectives, including whether the study describes the development or validation of the model or both.	3-4
**Methods**			
Source of data	4a	Describe the study design or source of data (e.g., randomized trial, cohort, or registry data), separately for the development and validation data sets, if applicable.	4
	4b	Specify the key study dates, including start of accrual; end of accrual; and, if applicable, end of follow-up.	4
Participants	5a	Specify key elements of the study setting (e.g., primary care, secondary care, general population) including number and location of centres.	4
	5b	Describe eligibility criteria for participants.	4
	5c	Give details of treatments received, if relevant.	N/A
Outcome	6a	Clearly define the outcome that is predicted by the prediction model, including how and when assessed.	4
	6b	Report any actions to blind assessment of the outcome to be predicted.	N/A
Predictors	7a	Clearly define all predictors used in developing or validating the multivariable prediction model, including how and when they were measured.	4,5
	7b	Report any actions to blind assessment of predictors for the outcome and other predictors.	N/A
Sample size	8	Explain how the study size was arrived at.	4
Missing data	9	Describe how missing data were handled (e.g., complete-case analysis, single imputation, multiple imputation) with details of any imputation method.	4
Statistical analysis methods	10a	Describe how predictors were handled in the analyses.	5,6
	10b	Specify type of model, all model-building procedures (including any predictor selection), and method for internal validation.	6,7
	10d	Specify all measures used to assess model performance and, if relevant, to compare multiple models.	7
Risk groups	11	Provide details on how risk groups were created, if done.	N/A
**Results**			
Participants	13a	Describe the flow of participants through the study, including the number of participants with and without the outcome and, if applicable, a summary of the follow-up time. A diagram may be helpful.	N/A
	13b	Describe the characteristics of the participants (basic demographics, clinical features, available predictors), including the number of participants with missing data for predictors and outcome.	4, 8-9
Model development	14a	Specify the number of participants and outcome events in each analysis.	8-9
	14b	If done, report the unadjusted association between each candidate predictor and outcome.	N/A
Model specification	15a	Present the full prediction model to allow predictions for individuals (i.e., all regression coefficients, and model intercept or baseline survival at a given time point).	N/A
	15b	Explain how to the use the prediction model.	N/A
Model performance	16	Report performance measures (with CIs) for the prediction model.	8-9,
**Discussion**			
Limitations	18	Discuss any limitations of the study (such as nonrepresentative sample, few events per predictor, missing data).	2, 11
Interpretation	19b	Give an overall interpretation of the results, considering objectives, limitations, and results from similar studies, and other relevant evidence.	11, 12
Implications	20	Discuss the potential clinical use of the model and implications for future research.	11, 12
**Other information**			
Supplementary information	21	Provide information about the availability of supplementary resources, such as study protocol, Web calculator, and data sets.	2,13
Funding	22	Give the source of funding and the role of the funders for the present study.	13

We recommend using the TRIPOD Checklist in conjunction with the TRIPOD Explanation and Elaboration document.
